# Manganese(I)-Catalyzed Asymmetric Hydrophosphination of α,β-Unsaturated Carbonyl Derivatives

**DOI:** 10.1021/acs.orglett.2c04256

**Published:** 2023-03-09

**Authors:** Roxana Postolache, Juana M. Pérez, Marta Castiñeira Reis, Luo Ge, Esther G. Sinnema, Syuzanna R. Harutyunyan

**Affiliations:** Stratingh Institute for Chemistry, University of Groningen, 9747 AG Groningen, The Netherlands

## Abstract

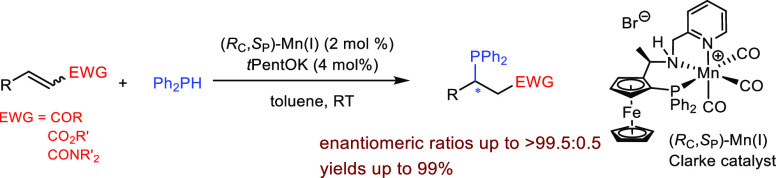

Here we report catalytic asymmetric hydrophosphination of α,β-unsaturated carbonyl derivatives using a chiral Mn(I) complex as a catalyst. Through H–P bond activation, various phosphine-containing chiral products can be accessed via hydrophosphination of various ketone-, ester-, and carboxamide-based Michael acceptors.

Phosphines containing carbon stereocenters make up an important class of compounds that have found applications in asymmetric catalysis,^1^ both as ligands, in combination with various transition metals, and in their own right as chiral organocatalysts.^2,3^ These phosphines are often difficult to access, due to their laborious, multistep synthesis, and therefore are expensive. In addition, their preparation commonly requires stoichiometric amounts of chiral auxiliaries or performing optical resolutions of preformed racemates.^4^ These aspects have hindered ready access to the library of chiral derivatives of phosphine ligands.^5^

Stereoselective hydrophosphination reactions offer an attractive strategy for accessing chiral compounds bearing phosphine moieties that can be further modified into chiral phosphine ligands. Consequently, the development of synthetic procedures for the formation of C–P bonds^6^ stereoselectively using metal-catalyzed hydrophosphination has been an active field of research in the past two decades.^7,8^ In 2001, the group of Glueck demonstrated the potential of catalytic asymmetric hydrophosphination reactions by obtaining moderate enantioselectivity in the Pt(0)-catalyzed hydrophosphination reaction of methacrylonitrile.^9^ Following this report, the use of noble metals such as Pt and Pd has been extensively explored for the synthesis of enantioenriched phosphines with stereogenic carbon.^10^ However, in recent years, the focus of the catalysis community has gradually shifted to the development of new competitive catalysts based on earth-abundant, readily available metals. In this context, a small number of examples of enantioselective hydrophosphinations have also been reported, all of which make use of chiral earth-abundant metal-based complexes of Ni^8e,8f,11^ and Cu.^8g,12^ Apart from organometallic catalysts, a few examples of asymmetric organocatalytic hydrophosphinations have also been reported.^13^ Recently, our group has reported the first case of asymmetric hydrophosphination of α,β-unsaturated nitriles using a readily available chiral manganese(I) complex.^14^ In addition to offering an attractive catalytic route to a variety of phosphines, this method stands out as the first example of catalytic H–P bond activation via metal–ligand cooperation.

Encouraged by these initial results, we decided to investigate the potential of Mn(I) catalysis for hydrophosphination of other Michael acceptors, namely *α,β*-unsaturated carbonyl derivatives of ketones, esters, and carboxamides.

We started our studies by testing hydrophosphination of *α,β-*unsaturated ketones catalyzed by the Clarke catalyst,^15^ (*R*_C_,*S*_P_)-Mn(I) (Scheme 1).

**Scheme 1:**
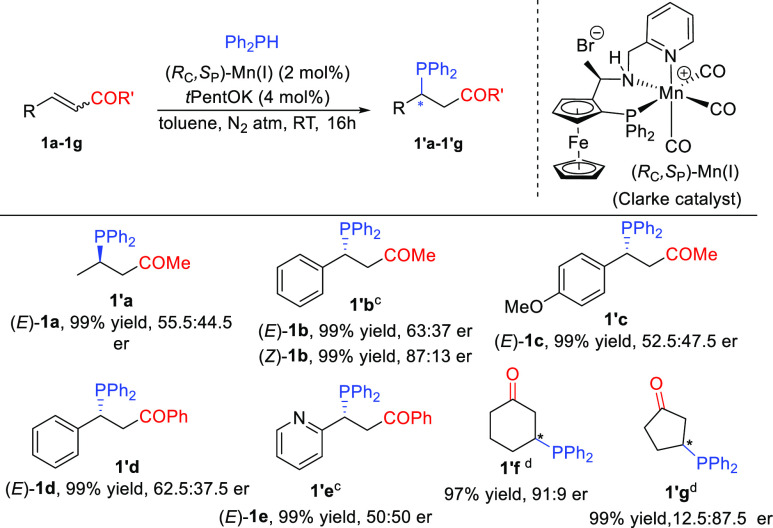
Catalytic Asymmetric Hydrophosphination of α,β-Unsaturated Ketones, Reaction conditions: 0.1 M **1** in toluene, (*R*_C_,*S*_P_)-Mn(I) (2 mol %), *t*PentOK (4 mol %), and Ph_2_PH (1.0 equiv) at RT under a N_2_ atmosphere. Isolated yields reported. For the assignment of the absolute configurations of **1′a**–**1′e**, see the Supporting Information. The absolute configuration of **1′f** and **1′g** was not determined. Reaction quenched with H_2_O_2_. The oxidized product was isolated and characterized (see the Supporting Information). Reaction carried out at 0 °C.

Mn(I) catalysis requires the use of a base to activate the catalyst via deprotonation of the benzylic amine moiety. We anticipated that the background base-catalyzed hydrophosphination might be a problem with *α,β*-unsaturated ketones, because of their enhanced reactivity compared to that of the *α,β*-unsaturated nitriles previously explored in this reaction.^14a^

The initial reaction was carried out between (*E*)-pent-3-en-2-one **1a** and diphenylphosphine, using 2 mol % (*R*_C_,*S*_P_)-Mn(I), 4 mol % *t*PentOK, and toluene as the solvent at room temperature (RT) under a nitrogen atmosphere (Scheme 1). While full conversion to the desired product was obtained, the enantiomeric ratio (er) was only 55.5:44.5. Similarly, a low er was found when using β-*p*-OMePh- and β-Ph-substituted substrates (*E*)*-***1b** and (*E*)-**1c**, respectively. Interestingly, (*Z*)-**1b** resulted in hydrophosphinated product **1′b** with an er higher than that obtained for (*E*)-**1b**. This was unexpected, because in our previous studies, (*Z*)- and (*E*)*-*configured β-alkyl-substituted aliphatic nitriles resulted in products with similarly high er values, while lower er’s were obtained from (*Z*)**-**β-aryl-substituted nitrile substrates. Hydrophosphination of chalcone (*E*)**-1d** afforded the corresponding product with a moderate er, similar to that obtained with (*E*)**-1b**. A racemic product was obtained with heteroaromatic substrate (*E*)-**1e**. Decreasing the temperature to 0 °C did not improve these results. Interestingly, in the case of cyclic aliphatic ketones (*E*)**-1f** and (*E*)**-1g**, this decrease in temperature did prove beneficial, yielding enantioselectivities of ≤91:9 (Scheme 1) at 0 °C. The initial conclusion from these results is that the stereochemical outcome of the reaction strongly depends on the configuration of the alkene. More specifically, (*Z*)-configured substrates are better suited for enantiodiscrimination by the chiral catalyst.

Next, we moved to study another class of Michael acceptors, namely the less reactive *α,β-*unsaturated esters (Scheme 2).

**Scheme 2:**
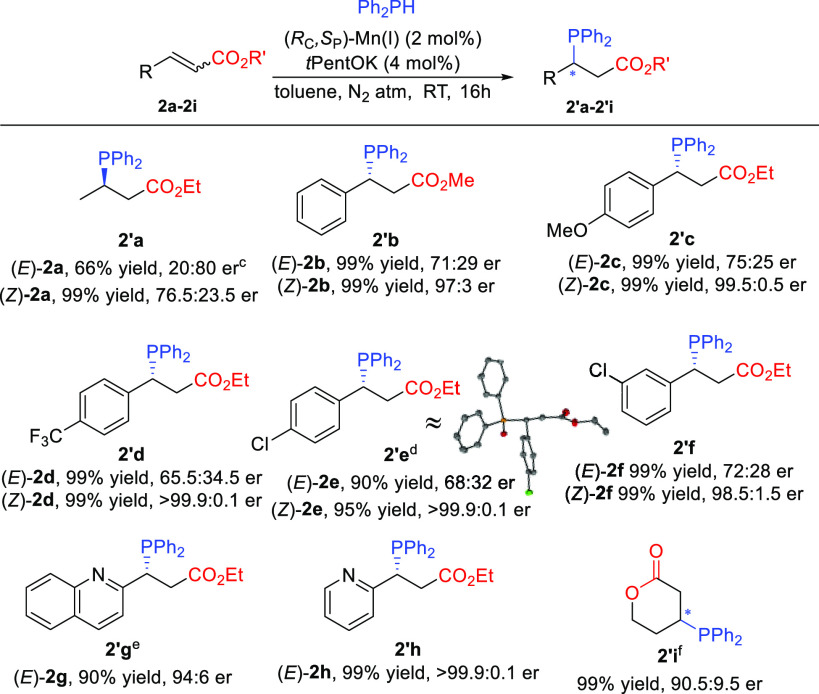
Scope of α,β-Unsaturated Esters, Reaction conditions: 0.1 M **2** in toluene, (*R*_C_,*S*_P_)-Mn(I) (2 mol %), *t*PentOK (4 mol %), and Ph_2_PH (1.0 equiv) at RT under a N_2_ atmosphere. Isolated yields reported. For the assignment of the absolute configurations of **2′a**–**2′h**, see the Supporting Information. The absolute configuration of **2′i** was not determined. *i*PrOH was used as a solvent in this case because of its slightly better er. This also resulted in a change in product configuration. The oxidized compound was used for X-ray crystallography. Reaction quenched with H_2_O_2_. The oxidized product was isolated and characterized (see the Supporting Information). Reaction carried out at 0 °C.

As with the ketones, we initially tested aliphatic (*E*)**-**enoate **2a**, obtaining the corresponding product with a promising 20:80 er. A similarly moderate er was obtained when the reaction was performed using (*Z*)*-***2a**. In contrast, greatly improved er’s were observed for products **2′b**–**2′f** derived from the corresponding (*Z*)-configured β-aryl-substituted *α,β-*unsaturated esters compared to the results with their (*E*)**-**counterparts. Importantly, both electron-donating (**2′c**) and electron-withdrawing functionalities (**2′d**–**2′f**) in the aromatic ring were tolerated. Unexpectedly, high enantioselectivities were obtained for (*E*)*-*configured substrates with heteroaryl-substituted esters (*E*)*-***2g** and (*E*)*-***2h**. We were pleased with these results because the corresponding (*Z*)-substrates are difficult to access and the chiral products obtained have potential for applications as precursors for chiral ligands in asymmetric catalysis.

As the carboxamide functional group is a common moiety in pharmaceuticals and biologically active compounds,^16^ we embarked on evaluating the scope of hydrophosphination of α,β-unsaturated carboxamides (Scheme 3). Highly enantioselective hydrophosphination of similar substrates was reported recently by the group of Yin using copper catalysis.^8g^ Given the low reactivity of these substrates, we wondered whether Mn(I) catalysis will also be competitive and how it would compare with the results obtained with nitriles, ketones, and esters.

**Scheme 3:**
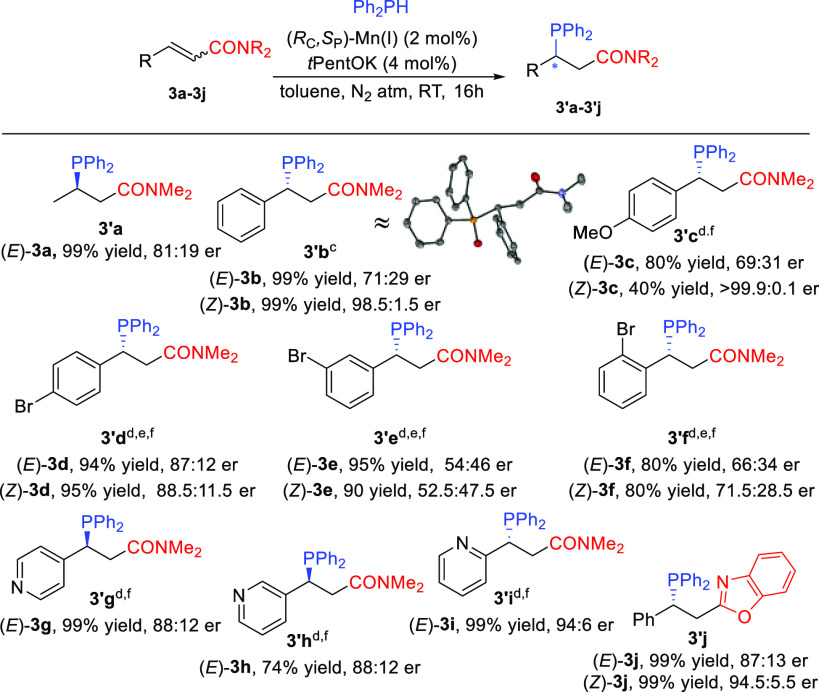
Scope of α,β-Unsaturated Amides, Reaction conditions: 0.1 M **3** in toluene, (*R*_C_,*S*_P_)-Mn(I) (2 mol %), *t*PentOK (4 mol %), and Ph_2_PH (1.0 equiv) at RT under a N_2_ atmosphere. Isolated yields reported. For the assignment of the absolute configurations of **3′a**–**3′j**, see the Supporting Information. The oxidized compound was used for X-ray crystallography. Here, 4 mol % (*R*_C_,*S*_P_)-Mn(I) and 8 mol % *t*PentOK were used. Reaction quenched with H_2_O_2_. The oxidized product was isolated and characterized (see the Supporting Information). Reaction time of 3 days.

The results were similar to those obtained with enoates, except that the difference in the stereochemical outcome between (*Z*)- and (*E*)-configured substrates was less pronounced. The (*Z*)-configured *α,β-*unsaturated carboxamides provided hydrophosphinated products with improved enantiomeric purities, but the stereochemical outcome of the reaction was to a larger extent dependent on the nature of the substituent at the β-position, albeit without a clear trend. Keeping in mind the possible application of the phosphine products derived from carboxamides as potential chiral ligands in asymmetric catalysis, we were grateful to see high er’s and yields for products **3′i** and **3′j** when using carboxamide substrates with a heteroaromatic group.

The ability of the Mn(I) complex to catalyze the hydrophosphination of various classes of Michael acceptors, together with several stereochemical observations made in this process, is intriguing. Among these observations are (i) the alkene class-dependent (*E*)- or (*Z*)-configuration required to achieve high er’s for aromatic substrates (nitriles vs other alkenes studied in this work) and (ii) the same sense of asymmetric induction for all of the (*E*)- and (*Z*)-configured substrates but the opposite sense of asymmetric induction when switching from aliphatic to aromatic substrates. While not aiming to rationalize all of these observations in this work, we undertook additional experimental studies to gain more insight. The fact that the same sense of asymmetric induction is observed when using an (*R*_C_,*S*_P_)-Mn(I) catalyst for hydrophosphination of (*E*)- and (*Z*)-configured alkenes could be indicative of alkene double bond isomerization during the reaction. However, the higher er’s obtained with the (*Z*)-alkenes investigated in this work are convincing proof against isomerization happening during the reaction catalyzed by manganese. This is further supported by the lack of base- or base/Mn(I)-catalyzed (*Z*)- and (*E*)-isomerization of enoate **2b**, as confirmed by ^1^H NMR monitoring of the reaction (see the Supporting Information).

The outcome of the hydrophosphination reaction depends critically on the relative rates of the desired Mn(I)-catalyzed and the undesired base-catalyzed pathways. Control experiments with nitriles^14a^ showed that there is base-catalyzed conversion of alkene substrates in the presence of only 4 mol % base at RT. On the contrary, a slight excess of base with respect to the Mn(I) catalyst is required as using equimolar amounts with base resulted in poor substrate conversion.^14^ Therefore, a delicate balance between the amount of catalyst and base is critical to ensure a minimal amount of free base present in the reaction mixture. To understand the role of the base-catalyzed reactions for the substrates studied in this work, we selected for every class of alkene (*E*)*-* and (*Z*)*-*configured substrate (**1b**–**3b**) to carry out the base- and Mn-catalyzed reactions. The substrate conversion and er of the product were analyzed after a 5 min reaction time (Scheme 4). We found that the base-catalyzed reactions with 3 mol % *t*PentOK proceed to full conversion toward the racemic phosphine products in all cases, with the exception of ketone **1b**. This ketone undergoes a side reaction due to base-promoted enolization. To avoid the enolization, we used non-enolizable ketone **1d** instead, and also in this case, full conversion to the product was obtained under base catalysis. These results show that base catalysis is very efficient and therefore can cause some erosion of er’s during the Mn(I)-catalyzed reaction. However, this is not always the case, as evidenced from the different enantioselectivities obtained for (*E*)- and (*Z*)-**1b**, while no hydrophosphination product is being formed under base catalysis. Therefore, the obtained data are not conclusive and do not by themselves evidence a direct correlation between the relative rate of the base-catalyzed reaction and the obtained er’s for various substrates. Most likely, both the detrimental effect of the free base and the level of enantiodiscrimination offered by the chiral catalyst are dependent on the specific structure of the alkene. More detailed mechanistic studies are required to elucidate this further.

**Scheme 4:**
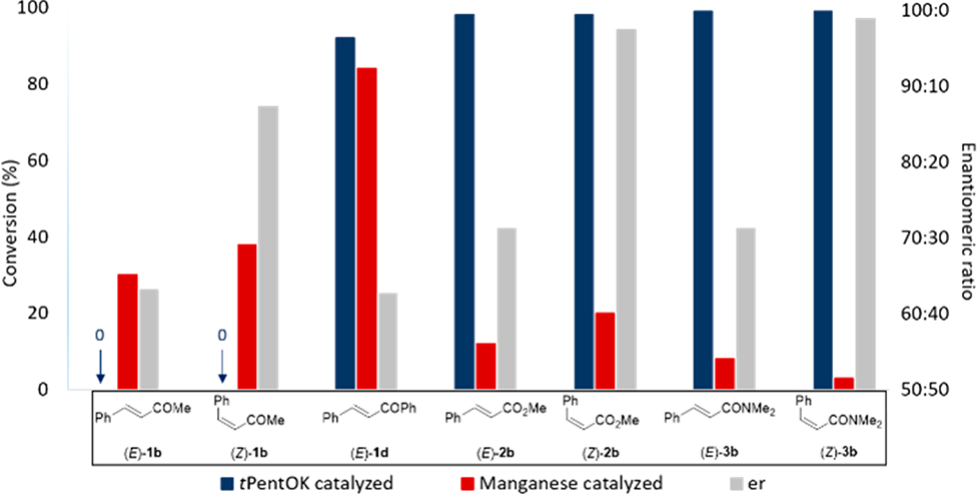
Outcome of the Base- and (*R*_C_,*S*_P_)/Mn(I)-Catalyzed Reactions Reaction conditions: 0.1 M (*E*)-**1b**, (*Z*)-**1b**, (*E*)-**1d**, (*E*)-**2b**, (*Z*)-**2b**, (*E*)-**3b**, or (*Z*)-**3b** and Ph_2_PH (1.0 equiv) in toluene at RT under a N_2_ atmosphere. For the base-catalyzed reaction, *t*PentOK (3 mol %) was used. For the Mn(I)-catalyzed reaction, (*R*_C_,*S*_P_)-Mn(I) (2 mol %) and *t*PentOK (4 mol %) were used. Conversions and er’s were measured after 5 min.

In conclusion, we have demonstrated that Mn(I)-catalyzed hydrophosphination can be applied to various classes of Michael acceptors. Currently, the portfolio of the Mn(I) catalysis includes nitriles, esters, carboxamides, and to a lesser extent ketones. Although the enantioselectivities are not homogeneously high across the substrate scope, this methodology shows the potential of Mn(I) catalysis for targeted synthesis of phosphine products and thus for providing readily available precursors for chiral ligands in asymmetric catalysis. Further structural tuning of the Mn catalyst to improve the enantioselectivities and to expand the scope is underway.

## Data Availability

The data underlying this study are available in the published article and its Supporting Information.

## Supporting Information Available

Experimental procedures, characterization data, and NMR spectra (PDF)
