# Supplementary material for: Manganese(I)-Catalyzed Asymmetric Hydrophosphination of α,β-Unsaturated Carbonyl Derivatives
Source: Org Lett. 2023 Mar 9;25(10):1611–5. doi: 10.1021/acs.orglett.2c04256 (PMC10028696; doi:10.1021/acs.orglett.2c04256)
Supplement: Supplementary file 1 — ol2c04256_si_001.pdf [file ol2c04256_si_001.pdf]

Supplementary Materials for

Manganese(I)-catalyzed asymmetric hydrophosphination of *α,β*-unsaturated carbonyl derivatives

Roxana Postolache,[‡] Juana M. Pérez,[‡] Marta Castiñeira Reis, Luo Ge, Esther G. Sinnema, Syuzanna R. Harutyunyan*

Stratingh Institute for Chemistry, University of Groningen, Nijenborgh 4, 9747 AG, Groningen, The Netherlands. [‡] These authors share the first authorship: Roxana Postolache, Juana M. Pérez. Correspondence and requests for materials should be addressed to S.R.H. (e-mail: s.harutyunyan@rug.nl)

This PDF file includes:

Materials and Methods

Figures S1 to S6

Tables S1, S2

NMR spectra

X-ray data

References

Table of Contents

1. General experimental information S3

2. Chemicals..S4

3. Determination of the absolute configuration S4

4. Manganese(I)-catalyzed enantioselective hydrophosphination reactions*.........................S5

4.1 General procedure 1..S5

4.2 General procedure 2: synthesis of racemic hydrophosphination products S6

5. Specific experimental details and characterization of the products of the catalytic enantioselective hydrophosphination reactions S7

5.1 Structures of starting materials and their corresponding products.....................................S7

5.2 Characterization of the products resulting from the manganese(I)-catalyzed hydrophosphination*..S9

6. (*Z*)/(*E*)-Isomerization experiments...S41

7. Synthesis of starting materials: general procedures and characterization of internal and terminal *α*,*β*-unsaturated nitriles, esters and amides S45

8. Characterization data of starting materials S47

9. NMR spectra S62

10. References...S107

1. General experimental information

All reactions using oxygen- and/or moisture-sensitive materials were carried out with dry and deoxygenated solvents (*vide infra*) under a nitrogen atmosphere using oven-dried glassware and standard Schlenk techniques. Manganese(I)-catalyzed hydrophosphination reactions were carried out in the glovebox. The reactions were monitored by ^{1}H NMR and ^{31}P NMR spectroscopy. Purification of the products was performed by flash column chromatography using Merck 60 Å 230-400 mesh silica gel or alumina when necessary. NMR data were collected on a Varian Mercury Plus (^{1}H at 400 MHz; ^{13}C at 101 MHz; ^{31}P at 162 MHz) equipped with a 5 mm PFG AutoSW probe and Bruker Avance NEO (^{1}H at 600 MHz; ^{13}C at 151 MHz; ^{31}P at 243 MHz), equipped with a 5 mm SmartProbe BBFO. Chemical shifts are reported in parts per million (ppm) relative to residual solvent peak ($CDCl_3$, ^{1}H: 7.26 ppm; ^{13}C: 77.2 ppm; CD_2Cl_2, ^{1}H: 5.32 ppm; ^{13}C: 54.0 ppm, toluene-d8, ^{1}H: 2.09, 6.98, 7.00, 7.09). Coupling constants are reported in Hertz (Hz). Multiplicity is reported with the usual abbreviations (s: singlet, br s: broad singlet, d: doublet, dd: doublet of doublets, dt: doublet of triplets, t: triplet, q: quartet, dq: doublet of quartets, m: multiplet). Please note: in the ^{13}C NMR spectra the symmetric carbons cannot be indicated and they are listed as one signal. If an apparent multiplicity is observed, the actual multiplicity will be noted in brackets. Exact mass spectra were recorded on a LTQ Orbitrap XL apparatus with ESI ionization. Enantiomeric ratios (er) were determined by Chiral HPLC analysis using a Shimadzu LC-10AD*VP* HPLC equipped with a Shimadzu SPD-M10A*VP* diode array detector or by a Thar SFC system equipped with chiralpak IC columns or Waters Acquity UPC2 system with PDA detector. To follow the reactivity, Shimadzu GCMS-QP2010 Ultra gas chromatograph mass spectrometer with FID detector (column: Agilent HP-5MS, dimensions: 30 m x 0.25 mm x 0.25 μm, phase: 5%-Phenyl-methylpolysiloxane) was used.

2. Chemicals

Unless otherwise indicated, reagents and substrates were purchased from commercial sources and used as received. Solvents that are not required to be dry were purchased as technical grade and used as received (pentane, EtOAc, Et_2O). Dry solvents were freshly collected from a dry solvent purification system (toluene, Et_2O, CH_2Cl_2, THF) or dried over molecular sieves (*i*PrOH) prior to use. Inert atmosphere experiments were performed with standard Schlenk techniques with dried (P_2O_5) nitrogen gas or inside the glovebox (the levels of O_2 and H_2O were maintained approximately under 0.1 ppm). Ph_2PH, (*E*)-**1a,** (*E*)-**1b,** (*E*)-**1c,** (*E*)-**1d,** (*E*)-**1e,** (*E*)-**1f,** (*E*)-**1g,** (*E*)-**2a**, (*E*)-**2b** and **2i** were purchased from Sigma-Aldrich, TCI and ABCR. All other substrates were prepared by literature reported methods (*vide infra*). All reported compounds and the manganese (R_C,S_P)-Clarke catalyst ((R_C,S_P)-Mn(I))[1] were characterized by ^1H, ^{13}C and ^{31}P NMR spectroscopy and compared with literature data. All new compounds were fully characterized by ^1H, ^{13}C and ^{31}P NMR, HPLC, SFC and HRMS techniques.

**High purity of dry solvents, reagents involved in the Clarke catalyst synthesis and alkene substrates is crucial to obtain reproducible results in the catalytic asymmetric hydrophosphination reactions (CAH).*

3. Determination of the absolute configuration

The absolute configuration of compounds **2'e** and **3'b** - derived from the hydrophosphination of the corresponding aromatic internal alkenes using (R_C,S_P)-Mn(I) was determined by X-ray crystallography analysis. The absolute configuration of other aromatic esters and amides were assigned by analogy. Taking into account that the configuration of aromatic nitrile products in our previous report (*J. Am. Chem. Soc*. **2021**, *143,* 20071) exhibits the same X-ray confirmed absolute configuration as that of the esters and amides **2'e** and **3'b** we believe the same configuration should be also obtained for acyclic aromatic ketone products **1'b-1'e** by analogy. Stereochemistry of cyclic products **1'f, 1'g** and **2'i** has not been determined**.**
Regarding aliphatic products: from the information above we know that that the aromatic nitrile, ester, amide and ketone products all giving the same product configuration. Therefore, we can conclude that between functional groups stereochemistry doesn't change and the phosphine attacks from the back side. The configuration of an aliphatic nitrile has been confirmed by X-ray, indicating phosphine attack from the front (*J. Am. Chem. Soc*. **2021**, *143*, 20071). Consequently we assume that for all the other aliphatic products with different functional groups the stereochemistry will e the same.

4. Manganese(I)-catalyzed enantioselective hydrophosphination reactions*

**Note:*
a) All the catalytic enantioselective reactions were carried out in duplo. In the cases when the enantiomeric ratios exceed 99:1 (see Scheme 2 and 3, Main text), *the corresponding reactions were carried out using both configurations of the chiral Mn-catalyst.*

b) High purity of all the starting materials, catalyst and solvents used in the hydrophosphination reaction is essential for the reaction to work.

4.1 General procedure 1: synthesis of the enantioselective hydrophosphination products

All the hydrophosphination reactions were performed inside the glovebox to ensure inert reaction conditions. In an oven-dried 4 mL vial equipped with a magnetic stirring bar, the (R_C,S_P)-Mn(I) (1.5 mg, 0.002 mmol, 2 mol%) was added and it was dissolved in the corresponding dry, deoxygenated solvent (1 mL of toluene or *i*PrOH) and stirred for 5 min. Then, a commercially available solution of *t*PentOK (0.042 M solution in toluene,* 95 µL, 0.004 mmol, 4 mol%) was added and the mixture was stirred for 5 min. When toluene is used as solvent and *t*PentOK as base, a color change of the solution from yellow to dark red is observed. This color change is indicative of the catalyst activation.** After 5 min stirring, the *α,β*-unsaturated substrate (0.1 mmol, 1.0 equiv) was added at once, followed by the addition of Ph_2PH (17 µL, 0.1 mmol, 1.0 equiv). The reaction was monitored by analyzing aliquots of the reaction mixture by ^{1}H NMR and ^{31}P NMR spectroscopy. Upon reaction completion, pentane (2 mL) was added to the reaction and the resulting mixture was filtered through a plug of alumina in order to remove the catalyst. EtOAc (1 mL) was added to flush the plug. The solvents were evaporated under reduced pressure and when necessary (if impurities are present) the crude was purified by column chromatography using SiO_2 as the solid phase and a mixture of pentane and EtOAc as eluent.***

When the reactions were carried out at 0 °C (to obtain products **1'f**, **** **1'g** and **2'i**), the reaction mixture, prior to the addition of Ph_2PH was prepared inside the glovebox in an oven-dried Schlenk equipped with a stirring bar and septum. The Schlenk was taken out of the glovebox and put under a nitrogen flow. The mixture was cooled and stirred at 0 °C for 10 min before adding the Ph_2PH through a siringe via the septum. The mixture was stirred at 0 °C under nitrogen atmosphere until full conversion to the product was observed. Upon reaction completion the work-up and purification were carried out as described above.

* *Note*: *A 1.7 M commercially available solution of tPentOK in toluene was diluted in order to obtain a 0.042 M solution which was used for the rection.*

*** Note: Absence of color change when toluene and tPentOK are used as the solvent and base, respectively, is indicative of either catalyst decomposition or insufficient amount of base. In such case no reaction will occur.*

**** Note: In the cases when the product was sensitive to oxidation, it was fully oxidized using H_2O_2 (30%, 0.25 mmol, 2.5 equiv) and the corresponding oxidized compound was characterized (**1'b**, **1'e**, **2'g**, **3'd**, **3'e** and **3'f**).*

**** The reaction with **1f** was carried in larger 1mm scale

4.2 General procedure 2: synthesis of the racemic hydrophosphination products

All the racemic hydrophosphination reactions were performed inside the glovebox to ensure inert reaction conditions. To an oven-dried 4 mL vial equipped with a magnetic stirring bar, the internal or terminal *α,β*-unsaturated alkene (1.0 mmol, 1.0 equiv) was added and dissolved in dry, deoxygenated toluene (1 mL). Then, a commercially available solution of *t*PentOK (1.7 M in toluene, 18 µL, 0.03 mmol, 0.3 equiv) was added followed by the addition of Ph_2PH (17 µL, 1.0 mmol, 1.0 equiv). After stirring overnight at RT, the reaction mixture was filtered through a plug of alumina and washed with EtOAc The solution was concentrated under reduced pressure to obtain the products which were used without further purification or purified by column chromatography when necesary.

5. Specific experimental details and characterization of the products of the catalytic enantioselective hydrophosphination reactions

5.1 Structures of starting materials and their corresponding products

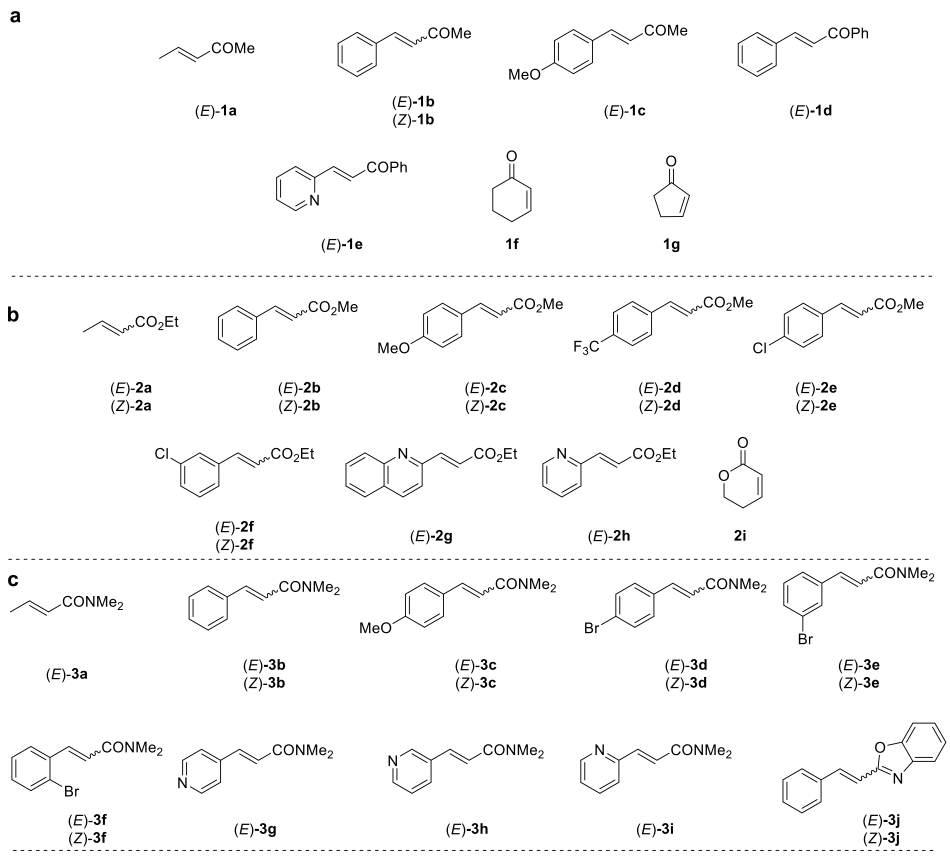

Figure S1. Structures of the starting materials used in the manganese(I)-catalyzed hydrophosphination. a. Structures of the internal *α,β*-unsaturated ketones. **b.** Structures of the internal *α,β*-unsaturated esters. **c.** Structures of the internal *α,β*-unsaturated amides.

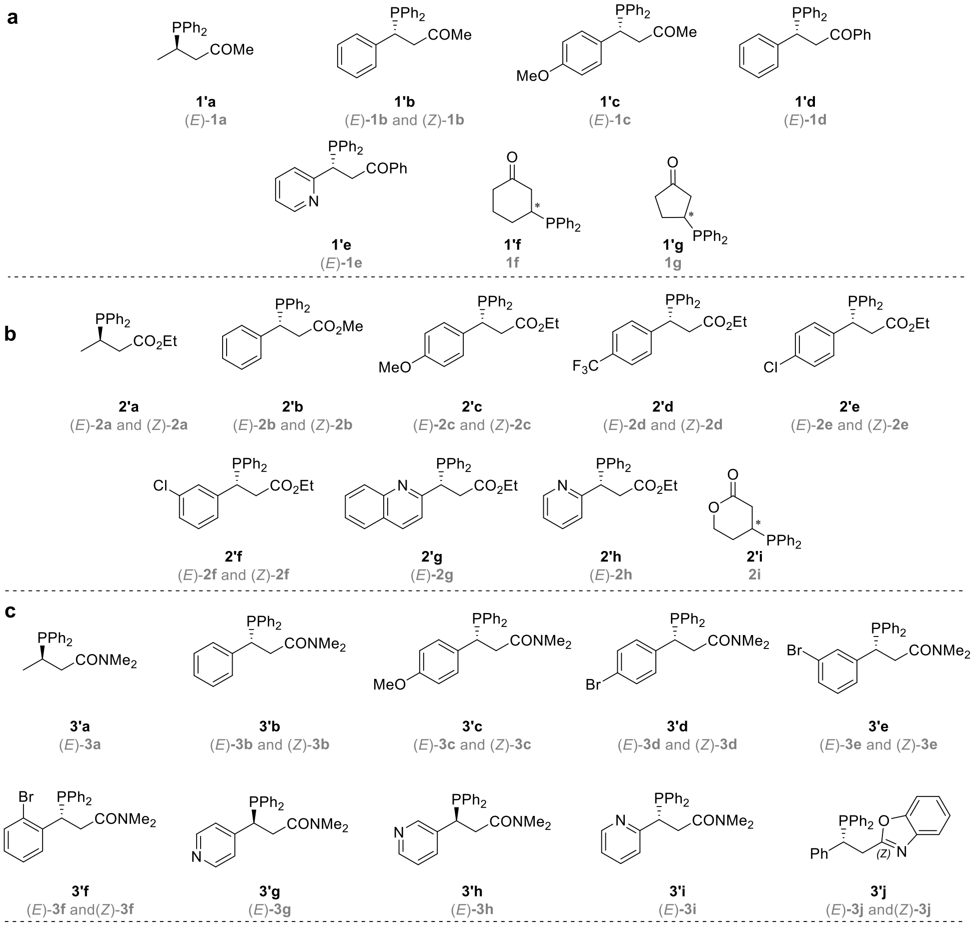

Figure S2. Structures of the products resulting from the manganese(I)-catalyzed hydrophosphination. a. Structures of the hydrophosphinated internal *α,β*-unsaturated ketones. **b.** Structures of the hydrophosphinated internal *α,β*-unsaturated esters. **b.** Structures of the hydrophosphinated internal *α,β*-unsaturated amides.

5.2 Characterization of the products resulting from the manganese(I)-catalyzed hydrophosphination*

** Note: When both the (E) and (Z)-configured substrates were used for the hydrophosphination reaction, the HPLC trace corresponding to the product obtained with the higher er is reported.*

(*R*)-3-(Diphenylphosphanyl)pentan-2-one (1’a):[2]

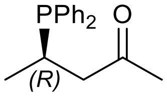

1’a

Compound **1’a** was synthesized following **general procedure 1** with (*E*)-**1a** (8 mg, 0.1 mmol, 1.0 equiv), Ph_2PH (17 µL, 0.1 mmol, 1.0 equiv), (R_C,S_P)-Mn(I) (1.5 mg, 0.002 mmol, 0.02 equiv), *t*PentOK (0.042 M, 95 µL, 0.004 mmol, 0.04 equiv), in dry, deoxigenated toluene (1 mL). Pentane (2 mL) was added and the resulting mixture was filtered through a plug of alumina in order to remove the catalyst. EtOAc (1 mL) was added to flush the plug. Product **1’a** was obtained as a colourless oil after evaporation of the solvent with reduced pressure. [(*E*)-**1a**, 99% yield, 55.5:45.5 er].

^{1}H NMR (400 MHz, $CDCl_3$): δ 7.52–7.49 (m, 4H), 7.36–7.30 (m, 6H), 3.02–2.92 (m, 1H), 2.52–2.44 (m, 1H), 2.41–2.33 (m, 1H), 2.07 (s, 3H), 1.03 (dd, *J* = 15.1, 6.8 Hz, 3H) ppm.

^{13}C NMR (151 MHz, $CDCl_3$): δ 207.5 (d, *J* = 12.8 Hz), 134.0, 133.9, 133.6 (d, *J* = 3.7 Hz), 133.4 (d, *J* = 3.6 Hz), 129.0 (2 x C), 128.5 (d, *J* = 7.2 Hz), 128.4 (d, *J* = 7.2 Hz), 47.1 (d, *J* = 17.1 Hz), 30.6 (d, *J* = 1.7 Hz), 25.4 (d, *J* = 8.8 Hz), 16.9 (d, *J* = 16.5 Hz) ppm.

^{31}P NMR (243 MHz, $CDCl_3$): δ -4.3 ppm.

CSP-HPLC: (254 nm, Chiralcel OJ-H, *n*-heptane:*i*PrOH = 99:1, 40 °C, 0.5 mL/min), t_R = 34.2 min (major), t_R = 39.7 min (minor).

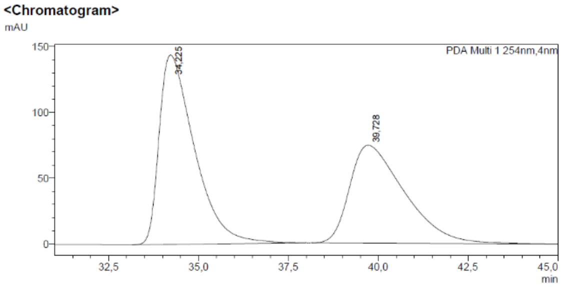

<Peak Table>

PDA Ch1 254nm

Peak#	Ret. Time	Area	Area%
1	34,225	9858152	55,382
2	39,728	7942140	44,618
Total		17800292	100,000

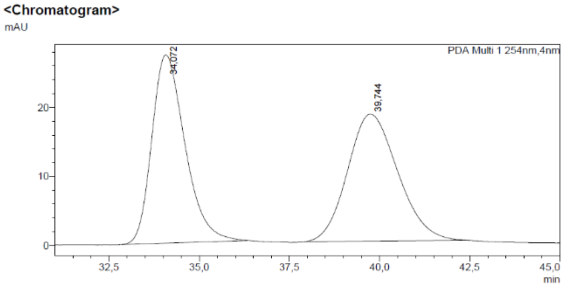

<Peak Table>

PDA Ch1 254nm

Peak#	Ret. Time	Area	Area%	Height%
1	34,072	1745180	49,439	59,701
2	39,744	1784753	50,561	40,299
Total		3529933	100,000	100,000

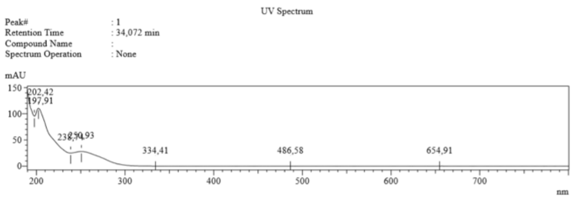

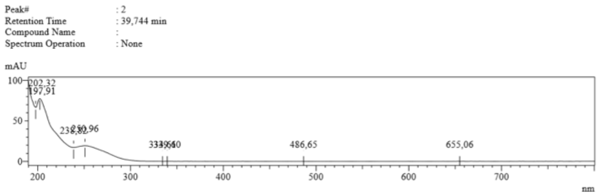

(*R*)-4-(Diphenylphosphoryl)-4-phenylbutan-2-one (1’b):[3]

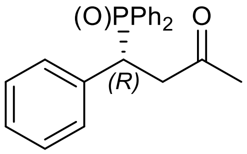

1’b

Compound **1’b** was synthesized following **general procedure 1** with (*E*)-**1b** or (*Z*)-**1b** (15 mg, 0.1 mmol, 1.0 equiv), Ph_2PH (17 µL, 0.1 mmol, 1.0 equiv), (R_C S_P)-Mn(I) (11.5 mg, 0.002 mmol, 0.02 equiv), *t*PentOK (0.042 M, 95 µL, 0.004 mmol, 0.04 equiv), in dry, deoxigenated toluene (1 mL). An aqueous solution of H_2O_2 (30%, 0.25 mmol, 2.5 equiv) was used to oxidize the product which was crashed out of the solution by additing of Et_2O. Product **1’b** was obtained after filtration and washing with Et_2O as a white solid. [(*E*)-**1’b**, 99% yield, 63:37 er], [(*Z*)-**1’b**, 99% yield, 87:13 er].

^{1}H NMR (400 MHz, $CDCl_3$): δ 7.95–7.91 (m, 2H), 7.57–7.51 (m, 3H), 7.45–7.41 (m, 2H), 7.35–7.22 (m, 6H), 7.18–7.11 (m, 2H), 4.24–4.19 (m, 1H), 3.37–3.28 (m, 1H), 2.98–2.90 (m, 1H), 1.95 (s, 3H) ppm.

^{13}C NMR (151 MHz, $CDCl_3$): δ 206.7 (d, *J* = 12.1 Hz), 140.5 (d, *J* = 8.8 Hz), 136.2 (d, *J* = 14.1 Hz), 135.9 (d, *J* = 15.8 Hz), 134.0 (d, *J* = 20.5 Hz), 133.1 (d, *J* = 18.4 Hz), 129.5, 128.9 (d, *J* = 7.7 Hz), 128.7 (d, *J* = 7.4 Hz), 128.5, 128.3, 127.9 (d, *J* = 6.9 Hz), 126.4 (d, *J* = 2.3 Hz), 47.1 (d, *J* = 20.6 Hz), 39.8 (d, *J* = 12.4 Hz), 30.8 ppm.

^{31}P NMR (162 MHz, $CDCl_3$): δ 33.6 ppm.

CSP-HPLC: (206 nm, Chiralcel AD-H, *n*-heptane:*i*PrOH = 80.20, 40 °C, 1 mL/min), t_R = 12.8 min (minor), t_R = 19.5 min (major).

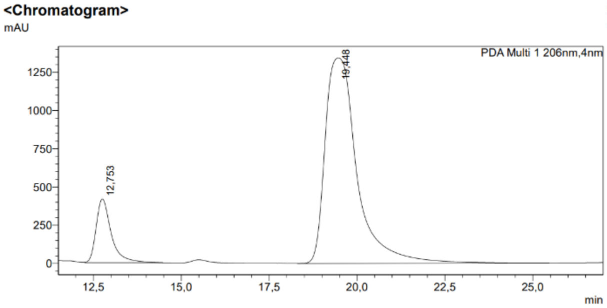

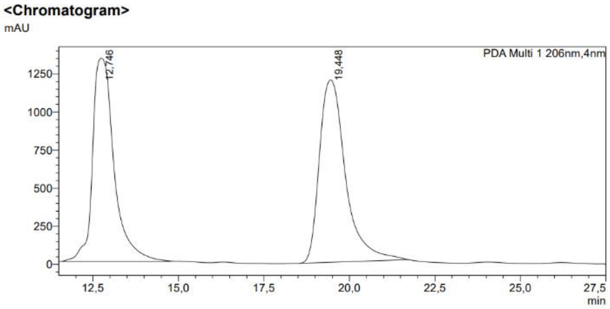

<Peak Table>
PDA Ch1 206nm

Peak#	Ret. Time	Area%
1	12,753	12,769
2	19,448	87,231
Total		100,000

<Peak Table>
PDA Ch1 206nm

Peak#	Ret. Time	Area%
1	12,746	47,879
2	19,448	52,121
Total		100,000

UV Spectrum

Peak# : 1
Retention Time : 12,753 min
Compound Name :
Spectrum Operation:

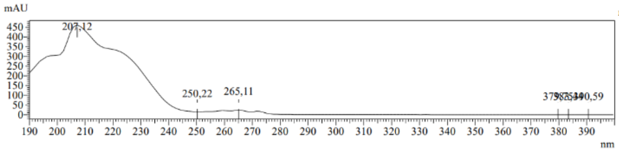

Peak# : 2
Retention Time : 19,448 min
Compound Name :
Spectrum Operation:

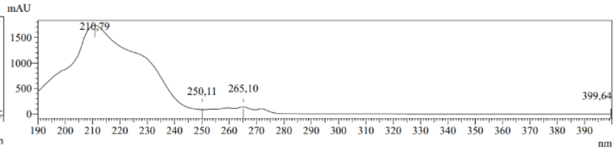

(*R*)-4-(Diphenylphosphaneyl)-4-(4-methoxyphenyl)butan-2-one (1'c):[4]

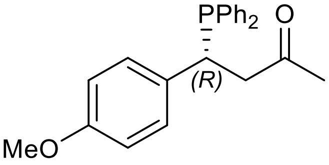

1'c

Compound **1'c** was synthesized following **general procedure 1** with (*E*)-**1c** (18 mg, 0.1 mmol, 1.0 equiv), Ph_2PH (17 µL, 0.1 mmol, 1.0 equiv), (R_C,S_P)-Mn(I) (1.5 mg, 0.002 mmol, 0.02 equiv), *t*PentOK (0.042M, 95 µL, 0.004 mmol, 0.04 equiv), in dry, deoxigenated toluene (1 mL). Pentane (2 mL) was added and the resulting mixture was filtered through a plug of alumina in order to remove the catalyst. EtOAc (1 mL) was added to flush the plug. Product **1'c** was obtained as a white solid after evaporation of the solvent with reduced pressure. [(*E*)-**1'c**, 99% yield, 52.5:47.5 er].

^{1}H-NMR (400 MHz, $CDCl_3$): δ 7.65–7.61 (m, 2H), 7.41–7.40 (m, 3H), 7.19–7.07 (m, 7H), 6.71 (d, *J* = 8.6 Hz, 2H), 4.07–4.02 (m, 1H), 3.73 (s, 3H), 3.04–2.96 (m, 1H), 2.64–2.57 (m, 1H), 1.89 (s, 3H) ppm.

^{13}C-NMR (101 MHz, $CDCl_3$): δ 206.8, 158.1, 136.4, 136.1 (d, *J* = 16.3 Hz), 134.1 (d, *J* = 20.0 Hz), 133.1 (d, *J* = 17.3 Hz), 132.4, 129.9, 129.5, 128.7, 128.5, 127.9, 113.8, 55.2, 47.3 (d, *J* = 20.1 Hz), 39.1, 30.9 ppm.

^{31}P-NMR (162 MHz, $CDCl_3$): δ –1.1 ppm.

CSP-HPLC: (254 nm, Chiralcel OD-H, *n*-heptane:*i*PrOH = 95.5, 40 °C, 1 mL/min), t_R = 80.8 min (minor), t_R = 83.6 min (major).

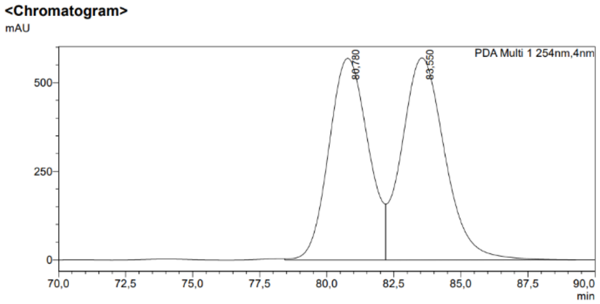

<Peak Table>
PDA Ch1 254nm

Peak#	Ret. Time	Area%
1	80,780	47,539
2	83,550	52,461
Total		100,000

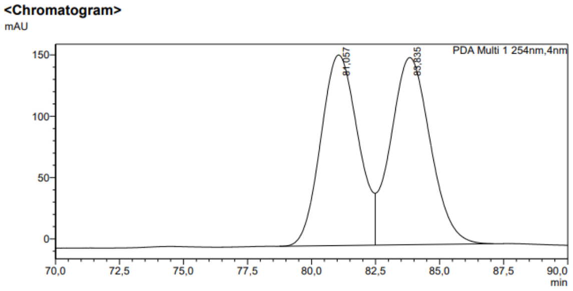

<Peak Table>
PDA Ch1 254nm

Peak#	Ret. Time	Area%
1	81,057	48,913
2	83,835	51,087
Total		100,000

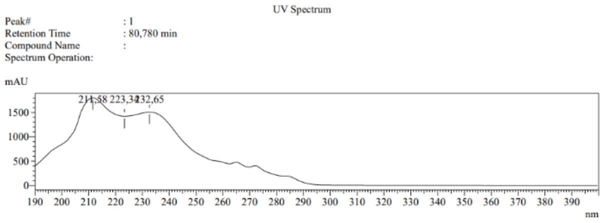

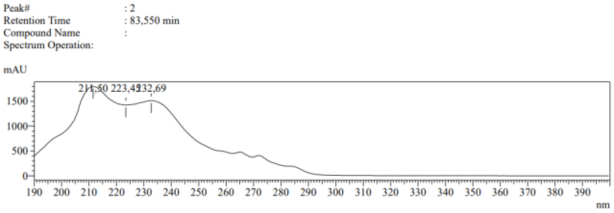

(*R*)-3-(Diphenylphosphanyl)-1,3-diphenylpropan-1-one (1'd):[5]

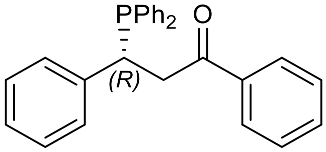

1'd

Compound **1'd** was synthesized following **general procedure 1** with (*E*)-**1d** (21 mg, 0.1 mmol, 1.0 equiv), Ph_2PH (17 µL, 0.1 mmol, 1.0 equiv), (R_C,S_P)-Mn(I) (1.5 mg, 0.002 mmol, 0.02 equiv), *t*PentOK (0.042 M, 95 µL, 0.004 mmol, 0.04 equiv), in dry, deoxigenated toluene (1 mL). Pentane (2 mL) was added and the resulting mixture was filtered through a plug of alumina in order to remove the catalyst. EtOAc (1 mL) was added to flush the plug. Product **1'd** was obtained as a white solid after evaporation of the solvent with reduced pressure. [99% yield, 62.5:37.5 er].

^{1}**H-NMR** (400 MHz, $CDCl_3$): δ 7.99–7.98 (m, 2H), 7.84 (d, *J* = 7.3 Hz, 2H), 7.52–7.48 (m, 7H), 7.39–7.32 (m, 5H), 7.25–7.21 (m, 1H), 7.14–7.07 (m, 3H), 4.49–4.46 (m, 1H), 4.06–3.98 (m, 1H), 3.43–3.35 (m 1H) ppm.

^{13}**C-NMR** (151 MHz, $CDCl_3$): δ 197.9 (d, *J* = 12.9 Hz), 140.7 (d, *J* = 8.6 Hz), 136.9, 136.4 (d, *J* = 14.3 Hz), 136.2 (d, *J* = 15.8 Hz), 134.1 (d, *J* = 20.4 Hz), 133.2 (d, *J* = 18.3 Hz), 133.0, 129.6,

129.1 (d, J = 7.6 Hz), 128.8 (d, J = 7.3 Hz), 128.6, 128.5, 128.2, 128.0, 127.9 (d, J = 6.9 Hz), 126.3, 42.4 (d, J = 22.2 Hz), 39.8 (d, J = 11.4 Hz) ppm.

^{31}P-NMR (162 MHz, $CDCl_3$): δ -0.5 ppm.

CSP-HPLC: (240 nm, Chiralcel OD-H, *n*-heptane:*i*PrOH = 98:2, 40 °C, 0.5 mL/min), t_R = 6.0 min (major), t_R = 6.9 min (minor).

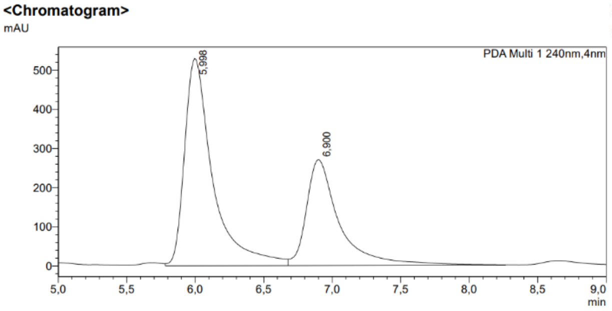

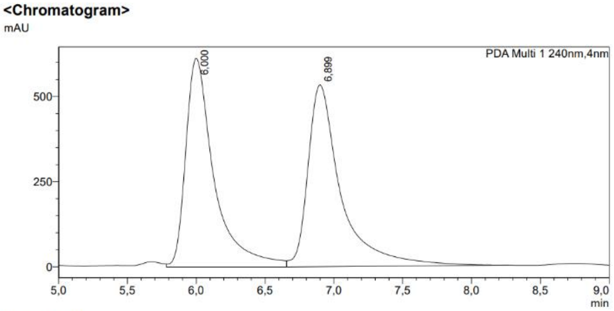

<Peak Table>
PDA Ch1 240nm

Peak#	Ret. Time	Area%
1	5,998	62,552
2	6,900	37,448
Total		100,000

<Peak Table>
PDA Ch1 240nm

Peak#	Ret. Time	Area%
1	6,000	49,690
2	6,899	50,310
Total		100,000

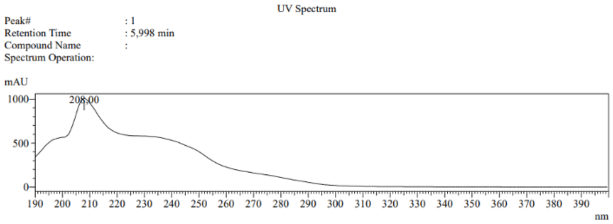

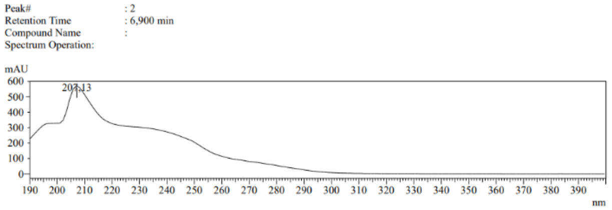

(*R*)-3-(Diphenylphosphaneyl)-1-phenyl-3-(pyridin-2-yl)propan-1-one (1'e):[6]

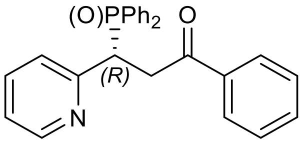

1'e

Compound **1'e** was synthesized following **general procedure 1** with (*E*)-**1e** (21 mg, 0.1 mmol, 1.0 equiv), Ph_2PH (17 µL, 0.1 mmol, 1.0 equiv), (R_C,S_P)-Mn(I) (1.5 mg, 0.002 mmol, 0.02 equiv), *t*PentOK (0.042 M, 95 µL, 0.004 mmol, 0.04 equiv), in dry, deoxigenated toluene (1 mL). An aqueous solution of H_2O_2 (30%, 0.25 mmol, 2.5 equiv) was used to oxidize the product which was crashed out of the solution by additing of Et_2O. Product **1'e** was obtained after filtration and washing with Et_2O as a white solid. [99% yield, 50:50 er].

^{1}H-NMR (400 MHz, $CDCl_3$): δ 8.61 (d, J = 4.3 Hz, 1H), 8.01–7.97 (m, 2H), 7.82 (d, J = 7.8 Hz, 1H), 7.73–7.69 (m, 1H), 7.51–7.46 (m, 5H), 7.41–7.24 (m, 6H), 7.14–7.08 (m, 3H), 4.48–4.34 (m, 2H), 3.61–3.54 (m 1H) ppm.

^{13}C-NMR (101 MHz, $CDCl_3$): δ 198.8 (d, *J* = 13.6 Hz), 152.7, 149.0, 136.6, 135.8 (d, *J* = 5.6 Hz), 132.3, 132.0, 131.9, 131.5 (d, *J* = 8.5 Hz), 131.3, 131.1 (d, *J* = 8.8 Hz), 130.0 (d, *J* = 5.4 Hz), 128.8, 128.7, 128.1 (2 x C), 128.0, 127.2, 127.0, 121.7, 41.5 (d, *J* = 68.6 Hz), 38.2 ppm.

^{31}P-NMR (162 MHz, $CDCl_3$): δ 33.7 ppm.

SFC: (254 nm, Waters Trefoil CEL1, MeOH, 40 °C, 1.8 mL/min), t_R = 3.3 min (minor), t_R = 3.6 min (major).

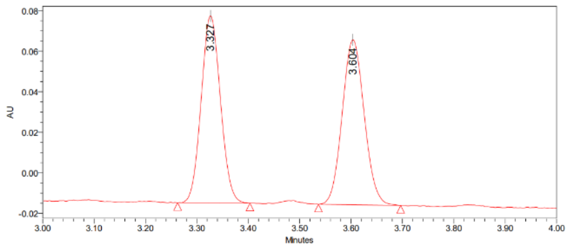

Peak Results

	Name	RT	Area	% Area	Height
1		3.327	240481	49.82	92163
2		3.604	242184	50.18	81334

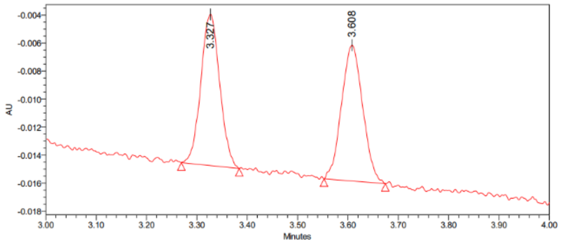

Peak Results

	Name	RT	Area	% Area	Height
1		3.327	28188	49.83	10804
2		3.608	28385	50.17	9704

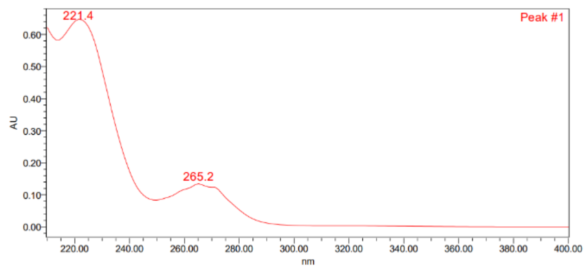

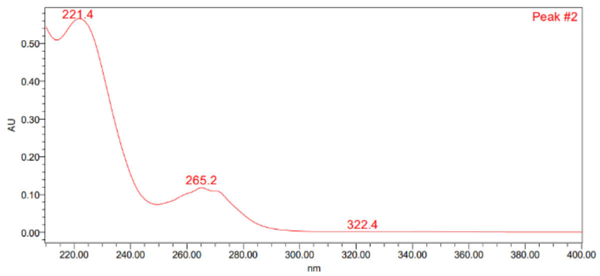

3-(Diphenylphosphaneyl)cyclohexan-1-one (1’f): 1mmol reaction

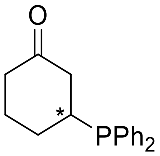

1’f

To an oven-dried Schlenk, located inside the glovebox and equipped with a septum and a magnetic stirring bar, the (R_C, S_P)-Mn(I) catalyst (15 mg, 0.02 mmol, 2 mol%) was added and it was dissolved in dry, deoxygenated toluene (10 mL). The mixture was stirred inside the glovebox for 5 min. Then, a commercially available solution of *t*PentOK (1.7 M in toluene, 4 mol%) was added and the mixture was stirred for 5 min to activate the catalyst. Upon activation, a change in the color of the solution from yellow to dark red was observed. The substrate **1f** (1.0 mmol, 1.0 equiv, 97 μL) was added at once. The Schlenk was taken out of the glovebox and put under a nitrogen flow. The mixture was cooled and stirred at 0 °C for 10 min before adding the Ph_2PH

(1.0 mmol, 1.0 equiv, 174 μL) through a siringe via the septum. Upon reaction completion (24h), the mixture was allowed to warm to room temperature. Pentane (5 mL) was added to the reaction and the resulting mixture was filtered through a plug of alumina in order to remove the catalyst. EtOAc (10 mL) was added to flush the plug and the solvents were evaporated under reduced pressure to afford **1’f** [97% yield, 91:9 er, 275 mg].

^{1}H NMR (400 MHz, $CDCl_3$): δ 7.51–7.44 (m, 4H), 7.36–7.33 (m, 6H), 2.69–2.59 (m, 1H), 2.42–2.12 (m, 5H), 1.92–1.86 (m, 1H), 1.80–1.68 (m, 1H), 1.63–1.51 (m, 1H) ppm.

^{13}C NMR (151 MHz, $CDCl_3$): δ 210.4 (d, J = 12.0 Hz), 135.9 (d, J = 13.5 Hz), 135.2 (d, J = 13.6 Hz), 133.7 (d, J = 19.9 Hz), 133.4 (d, J = 19.4 Hz), 129.3, 129.1, 128.6 (d, J = 5.4 Hz), 128.5 (d, J = 5.2 Hz), 44.1 (d, J = 14.2 Hz), 41.4, 36.0 (d, J = 12.6 Hz), 28.2 (d, J = 16.4 Hz), 26.8 (d, J = 12.6 Hz) ppm.

^{31}P NMR (243 MHz, $CDCl_3$): δ -3.6 ppm.

CSP-HPLC: (254 nm, Chiralcel AD-H, *n*-heptane:*i*PrOH = 99:1, 40 °C, 0.5 mL/min), t_R = 35.8 min (minor), t_R = 40.6 min (major).

<Chromatogram>

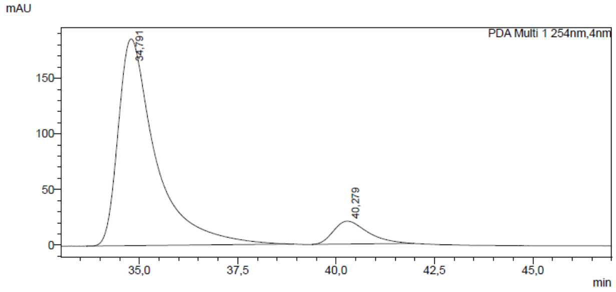

<Peak Table>

PDA Ch1 254nm

Peak#	Ret. Time	Area	Height	Area%
1	34,791	12374919	185913	90,813
2	40,279	1251851	20599	9,187
Total		13626771	206511	100,000

<Chromatogram>

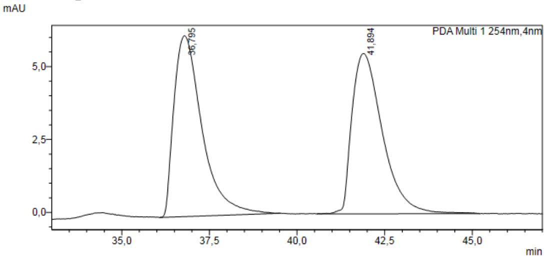

<Peak Table>

PDA Ch1 254nm

Peak#	Ret. Time	Area	Area%
1	36,795	347232	50,994
2	41,894	333699	49,006
Total		680931	100,000

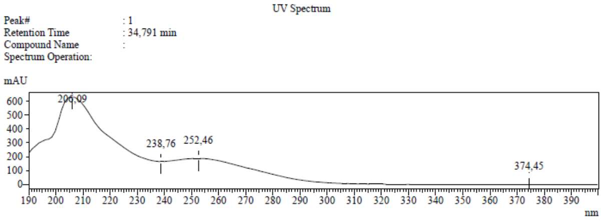

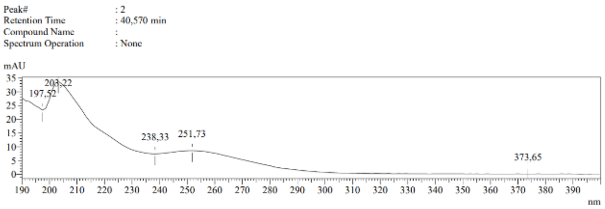

3-(Diphenylphosphanyl)cyclopentan-1-one (1g):

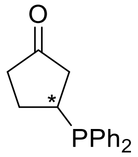

1’g

Compound **1’g** was synthesized following **general procedure 1** with **1g** (8 mg, 0.1 mmol, 1.0 equiv), Ph_2PH (17 µL, 0.1 mmol, 1.0 equiv), (R_C,S_P)-Mn(I) (1.5 mg, 0.002 mmol, 0.02 equiv), *t*PentOK (0.042 M, 95 µL 0.004 mmol, 0.04 equiv), in dry, deoxygenated toluene (1 mL) at 0 °C. Pentane (2 mL) was added and the resulting mixture was filtered through a plug of alumina in order to remove the catalyst. EtOAc (1 mL) was added to flush the plug. Product **1’g** was obtained as a colorless oil after short column chromatography (SiO_2, Pentane). [99% yield, 12.5:87.5 er].

^{1}H-NMR (600 MHz, $CDCl_3$): δ 7.51–7.48 (m, 2H), 7.46–7.44 (m, 2H), 7.37–7.34 (m, 6H), 2.97–2.91 (m, 1H), 2.42–2.34 (m, 2H), 2.23–2.08 (m, 3H), 1.91–1.83 (m, 1H) ppm.

^{13}C-NMR (151 MHz, $CDCl_3$): δ 218.1 (d, J = 10.4 Hz), 136.8 (d, J = 12.4 Hz), 136.6 (d, J = 12.5 Hz), 133.3 (d, J = 19.0 Hz), 133.1 (d, J = 18.8 Hz), 129.2, 129.1, 128.6 (2 x C), 42.9 (d, J = 18.0 Hz), 38.2 (d, J = 5.5 Hz), 33.9 (d, J = 9.3 Hz), 27.1 (d, J = 17.8 Hz) ppm.

^{31}P-NMR (243 MHz, $CDCl_3$): δ -4.9 ppm.

CSP-HPLC: (254 nm, Chiralcel AD-H, *n*-heptane:*i*PrOH = 99:1, 40 °C, 0.5 mL/min.), t_R = 36.8 min (minor), t_R = 38.9 min (major).

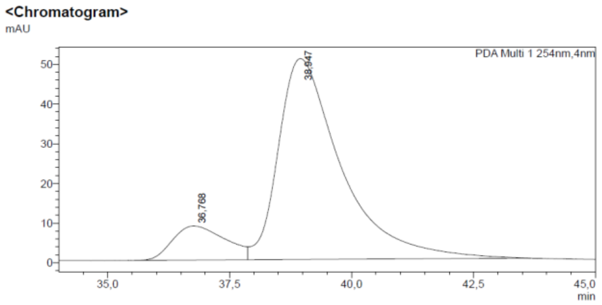

<Peak Table>
PDA Ch1 254nm

Peak#	Ret. Time	Area	Height	Area%
1	36,768	644825	8581	12,443
2	38,947	4537238	50595	87,557
Total		5182063	59176	100,000

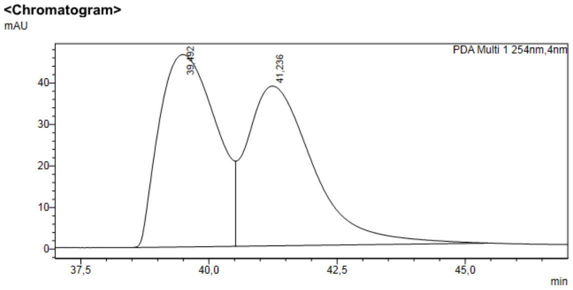

<Peak Table>
PDA Ch1 254nm

Peak#	Ret. Time	Area	Area%
1	39,492	3577762	50,763
2	41,236	3470278	49,237
Total		7048040	100,000

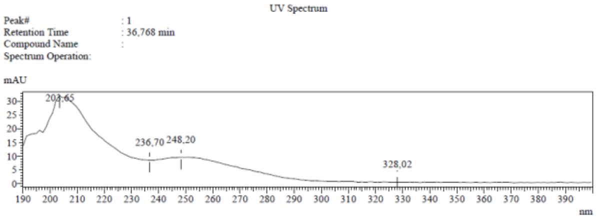

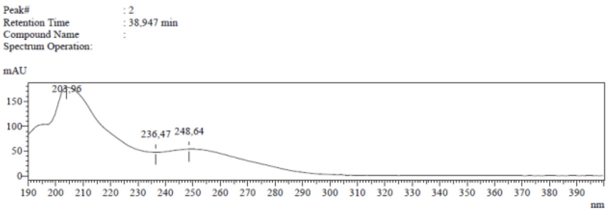

Ethyl (*R*)-3-(diphenylphosphaneyl)butanoate (2'a):

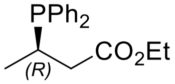

2'a

Compound **3'a** was synthesized following **general procedure 1** with (*E*)-**2a** or (*Z*)-**2a** (11 mg, 0.1 mmol, 1.0 equiv), Ph_2PH (17 µL, 0.1 mmol, 1.0 equiv), (R_C,S_P)-Mn(I) (1.5 mg, 0.002 mmol, 0.02 equiv), *t*PentOK (0.042 M, 95 µL, 0.004 mmol, 0.04 equiv), in dry, deoxygenated toluene or *i*PrOH (1 mL). Pentane (2 mL) was added and the resulting mixture was filtered through a plug of alumina in order to remove the catalyst. EtOAc (1 mL) was added to flush the plug. Product **2'a** was obtained as a colorless oil after evaporation of the solvent under reduced pressure. [(*E*)-**2a**, 66% yield, 20:80 er], [(*Z*)-**2a**, 99% yield, 76.5:23.5 er].

For this substrate the reaction was carried out in iPrOH with (E)-2a.** The resulting product was obtained major enantiomer with absolute configuration (S) as opposed to (R)-configurations typically obtained in toluene. When the reaction was performed in toluene, (Z)-**2a** was used as substrate yielding the (R)-enantiomer of **2'a**. The reported HPLC data correspond to the results obtained with substrate (Z)-**2a**.*

^{1}H NMR (400 MHz, $CDCl_3$): δ 7.53–7.48 (m, 4H), 7.36–7.31 (m, 6H), 4.12–4.06 (m, 2H), 2.92–2.82 (m, 1H), 2.47–2.41 (m, 1H), 2.19–2.11 (m, 1H), 1.23 (t, *J* = 7.1 Hz, 3H), 1.08 (dd, *J* = 14.9, 6.8 Hz, 3H) ppm.

^{13}C NMR (101 MHz, $CDCl_3$): δ 172.8 (d, *J* = 16.1 Hz), 136.5 (d, *J* = 3.1 Hz), 136.4 (d, *J* = 3.7 Hz), 133.8 (d, *J* = 5.5 Hz), 133.6 (d, *J* = 5.5 Hz), 129.1 (2 x C), 128.6 (d, *J* = 6.9 Hz), 128.5, 60.6, 38.6 (d, *J* = 19.0 Hz), 27.2 (d, *J* = 10.2 Hz), 16.9 (d, *J* = 16.6 Hz), 14.4 ppm.

^{31}P NMR (162 MHz, $CDCl_3$): δ -4.1 ppm.

HRMS (ESI+, m/z) calc. for $C_{18}H_{22}O_2P$: $[M+H]^+$ = 301.1352, found $[M+H]^+$ = 301.1358.

CSP-HPLC: (254 nm, Chiralcel OD-H, *n*-heptane:*i*PrOH = 95:5, 40 °C, 0.5 mL/min), t_R = 8.9 min (minor), t_R = 10.9 min (major).

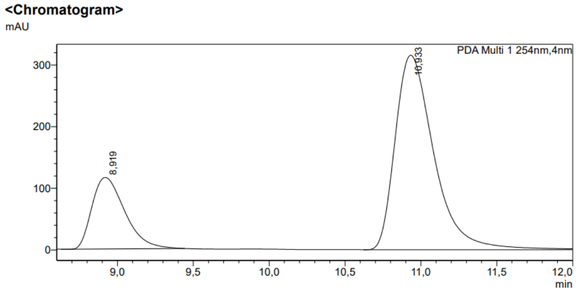

<Peak Table>
PDA Ch1 254nm

Peak#	Ret. Time	Area	Area%
1	8,919	1755691	23,599
2	10,933	5683958	76,401
Total		7439649	100,000

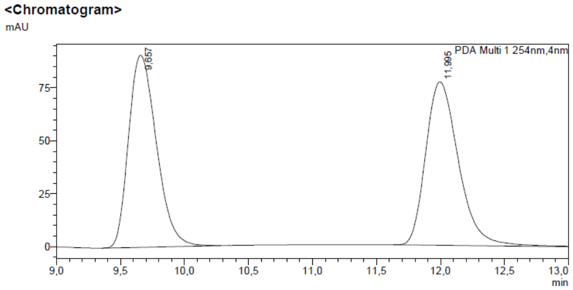

<Peak Table>
PDA Ch1 254nm

Peak#	Ret. Time	Area	Area%
1	9,657	1374151	49,877
2	11,995	1380955	50,123
Total		2755106	100,000

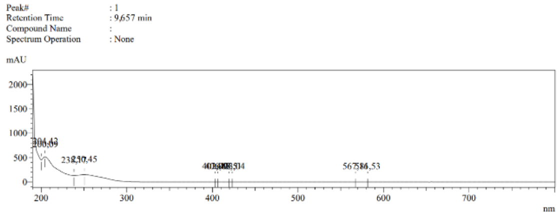

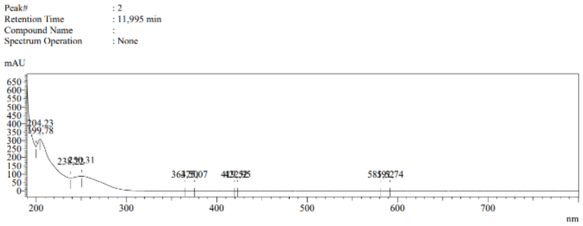

Methyl (*R*)-3-(diphenylphosphaneyl)-3-phenylpropanoate (2'b):

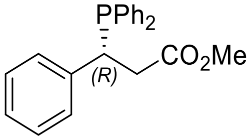

2'b

Compound **2'b** was synthesized following **general procedure 1** with (*E*)-**2b** or (*Z*)-**2b** (18 mg, 0.1 mmol, 1.0 equiv), Ph_2PH (17 µL, 0.1 mmol, 1.0 equiv), (R_C,S_P)-Mn(I) (1.5 mg, 0.002 mmol, 0.02 equiv), *t*PentOK (0.042 M, 95 µL, 0.004 mmol, 0.04 equiv), in dry, deoxygenated toluene (1 mL). Pentane (2 mL) was added and the resulting mixture was filtered through a plug of alumina in order to remove the catalyst. EtOAc (1 mL) was added to flush the plug. Product **2'b** was obtained as a white solid after evaporation of the solvent under reduced pressure. The NMR data are in agreement with literature precedents.[7] [(*E*)-**2b**, 99% yield, 71:29 er], [(*Z*)-**2b,** 99% yield, 97:3 er].

^{1}H NMR (400 MHz, $CDCl_3$): δ 7.97–7.92 (m, 2H), 7.56–7.54 (m, 3H), 7.47–7.42 (m, 2H), 7.36–7.33 (m, 1H), 7.26–7.15 (m, 7H), 4.09–4.05 (m, 1H), 3.47 (s, 3H), 3.15–3.07 (m, 1H), 2.94–2.87 (m, 1H) ppm.

^{13}C NMR (151 MHz, $CDCl_3$): δ 172.3 (d, *J* = 16.0 Hz), 140.1 (d, *J* = 8.6 Hz), 136.2 (d, *J* = 14.2 Hz), 135.9 (d, *J* = 16.0 Hz), 134.2 (d, *J* = 20.7 Hz), 133.2 (d, *J* = 18.3 Hz), 129.7, 129.0 (d, *J* = 7.3 Hz), 128.8 (d, *J* = 7.4 Hz), 128.7, 128.4, 128.0 (d, *J* = 6.7 Hz), 126.7 (d, *J* = 2.2 Hz), 51.7, 41.1 (d, *J* = 13.0 Hz), 38.4 (d, *J* = 23.0 Hz) ppm.

^{31}P NMR (243 MHz, $CDCl_3$): δ -3.2 ppm.

CSP-HPLC: (254 nm, Chiralcel AD-H, *n*-heptane:*i*PrOH = 99:1, 40 °C, 0.5 mL/min.), t_R = 23.2 min (minor), t_R = 26.3 min (major).

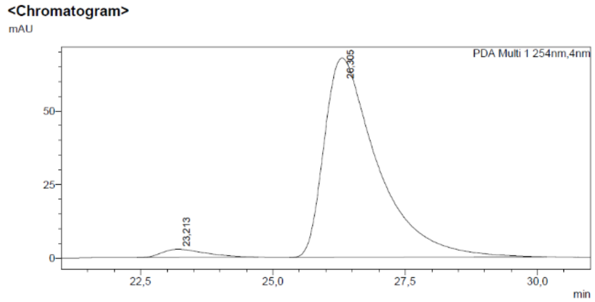

<Peak Table>
PDA Ch1 254nm

Peak#	Ret. Time	Area	Area%
1	23,213	159377	3,246
2	26,305	4750637	96,754
Total		4910014	100,000

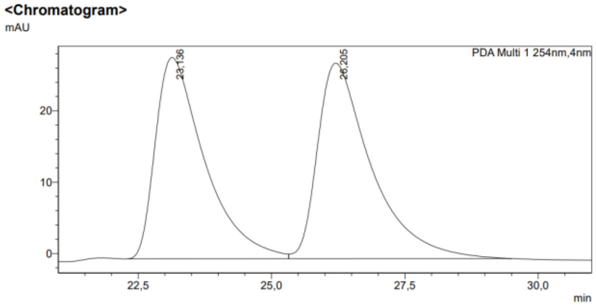

<Peak Table>
PDA Ch1 254nm

Peak#	Ret. Time	Area%
1	23,136	48,584
2	26,205	51,416
Total		100,000

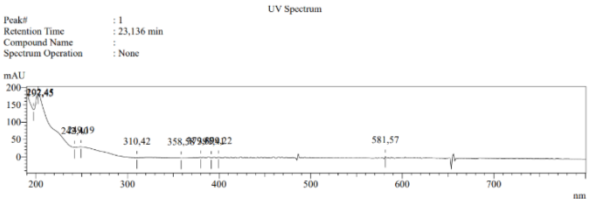

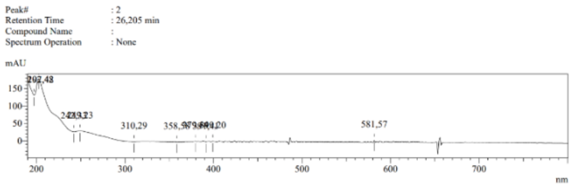

Ethyl (*R*)-3-(Diphenylphosphaneyl)-3-(4-methoxyphenyl)propanoate (2’c):

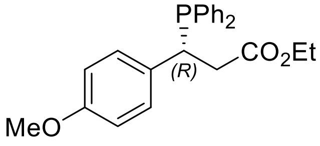

2’c

Compound **2’c** was synthesized following **general procedure 1** with (*E*)-**2c** or (*Z*)-**2c** (21 mg, 0.1 mmol, 1.0 equiv), Ph_2PH (17 µL, 0.1 mmol, 1.0 equiv), (R_C,S_P)-Mn(I) (1.5 mg, 0.002 mmol, 0.02 equiv), *t*PentOK (0.042 M, 95 µL, 0.004 mmol, 0.04 equiv), in dry, deoxygenated toluene (1 mL). Pentane (2 mL) was added and the resulting mixture was filtered through a plug of alumina in order to remove the catalyst. EtOAc (1 mL) was added to flush the plug. Product **2’c** was obtained as a white solid after evaporation of the solvent under reduced pressure. [(*E*)-**2c**, 99% yield, 75:25 er], [(*Z*)-**2c**, 99% yield, 99.5:0.5 er].

^{1}H NMR (600 MHz, $CDCl_3$): δ 7.68–7.66 (m, 2H), 7.44–7.43 (m, 3H), 7.21–7.16 (m, 5H), 7.11 (d, *J* = 8.5 Hz, 2H), 6.75 (d, *J* = 8.5 Hz, 2H), 4.01–3.97 (m, 1H), 3.96–3.92 (m, 2H), 3.76 (s, 3H), 2.80–2.74 (m, 1H), 2.67–2.63 (m, 1H), 1.07 (t, *J* = 7.1 Hz, 3H) ppm.

^{13}C NMR (151 MHz, $CDCl_3$): δ 171.8 (d, *J* = 16.4 Hz), 158.1 (d, *J* = 2.3 Hz), 136.3 (d, *J* = 14.0 Hz), 135.9 (d, *J* = 16.3 Hz), 134.1 (d, *J* = 20.7 Hz), 133.1 (d, *J* = 20.7 Hz), 131.7 (d, *J* = 8.9 Hz), 129.8 (d, *J* = 7.3 Hz), 129.4, 128.6 (d, *J* = 7.5 Hz), 128.4, 127.9 (d, *J* = 6.8 Hz), 113.6, 60.3, 55.1, 40.1 (d, *J* = 12.5 Hz), 38.6 (d, *J* = 23.4 Hz), 14.0 ppm.

^{31}P NMR (243 MHz, $CDCl_3$): δ -3.9 ppm.

HRMS (ESI+, m/z) calc. for $C_{24}H_{26}O_4P$ (oxidized product): $[M+H]^+$ = 409.1563, found $[M+H]^+$ = 409.1561.

CSP-HPLC: (254 nm, Chiralcel OD-H, *n*-heptane:*i*PrOH = 98:2, 40 °C, 1.0 mL/min), t_R = 7.5 min (minor), t_R = 8.6 min (major).

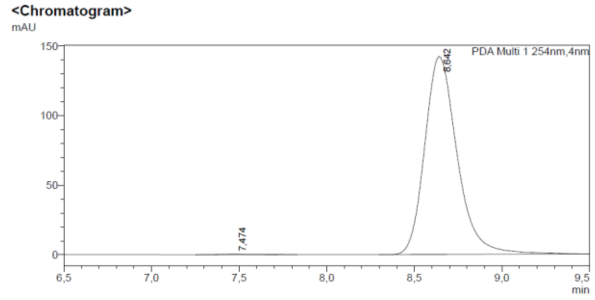

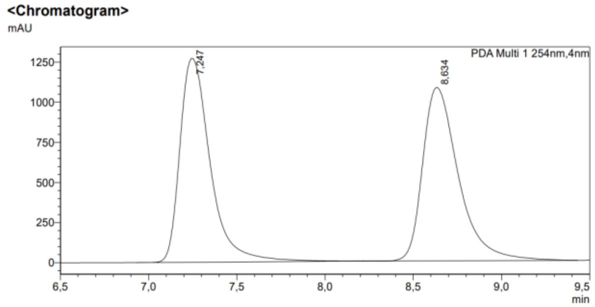

<Peak Table>

PDA Ch1 254nm

Peak#	Ret. Time	Area	Area%
1	7,474	7258	0,395
2	8,642	1831593	99,605
Total		1838852	100,000

<Peak Table>

PDA Ch1 254nm

Peak#	Ret. Time	Area	Area%
1	7,247	15210535	49,908
2	8,634	15266567	50,092
Total		30477103	100,000

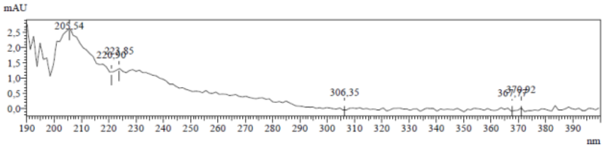

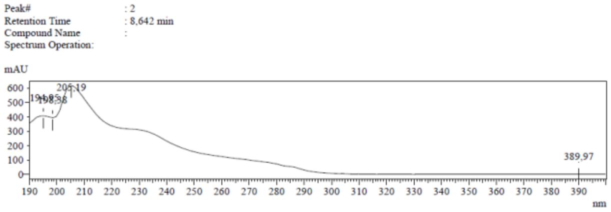

Ethyl (*R*)-3-(diphenylphosphaneyl)-3-(4-(trifluoromethyl)phenyl)propanoate (2’d):

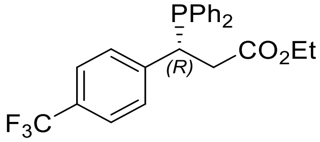

2’d

Compound **2’d** was synthesized following **general procedure 1** with (*E*)-**2d** or (*Z*)-**2d** (24 mg, 0.1 mmol, 1.0 equiv), Ph_2PH (17 µL, 0.1 mmol, 1.0 equiv), (R_C,S_P)-Mn(I) (1.5 mg, 0.002 mmol, 0.02 equiv), *t*PentOK (0.042 M, 95 µL, 0.004 mmol, 0.04 equiv), in dry, deoxygenated toluene (1 mL). Pentane (2 mL) was added and the resulting mixture was filtered through a plug of alumina in order to remove the catalyst. EtOAc (1 mL) was added to flush the plug. Product **2’d** was obtained as a white solid after evaporation of the solvent under reduced pressure. [(*E*)-**2d**, 99% yield, 65.5:34.5 er], [(*Z*)-**2d,** 99% yield, >99.9:0.1 er].

^{1}H NMR (600 MHz, $CDCl_3$): δ 7.87–7.80 (m, 2H), 7.47–7.45 (m, 4H), 7.35–7.26 (m, 6H), 7.16–7.15 (m, 2H), 4.07–3.97 (1H), 3.83–3.79 (m, 2H), 3.04–2.92 (m, 1H), 2.81–2.73 (m, 1H), 0.92 (t, *J* = 6.6 Hz, 3H) ppm.

^{13}C NMR (151 MHz, $CDCl_3$): δ 171.5, 171.4, 137.9, 135.4, 135.3, 135.2, 135.1, 134.1, 133.9, 133.2, 133.1, 129.8, 129.2 (2 x C), 129.1, 129.0, 128.9 (2 x C), 128.3, 128.2, 128.1, 125.3, 125.1 (2 x C), 61.0, 60.7, 41.1, 41.0, 38.1, 38.0, 21.5, 14.0 ppm. Due to complex splitting caused by the CF_3, peaks are reported only by their chemical shift.

^{31}P NMR (243 MHz, $CDCl_3$): δ -2.2 ppm.

HRMS (ESI+, m/z) calc. for $C_{24}H_{23}F_3O_3P$: $[M+H]^+$ (oxidized product) = 447.1331, found $[M+H]^+$ = 447.1338.

CSP-HPLC: (225 nm, Chiralcel OD-H, *n*-heptane:*i*PrOH = 99:1, 40 °C, 0.5 mL/min), t_R = 10.8 min (minor), t_R = 18.4 min (major). **The peak at 12.5 min present in the left chromatogram has a different UV/Vis spectrum than that of **2’d**.*

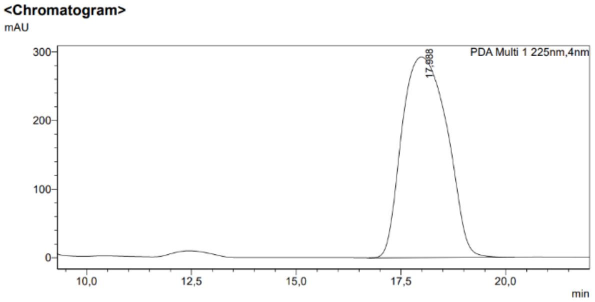

<Peak Table>
PDA Ch1 225nm

Peak#	Ret. Time	Area	Area%
1	17,988	21972973	100,000
Total		21972973	100,000

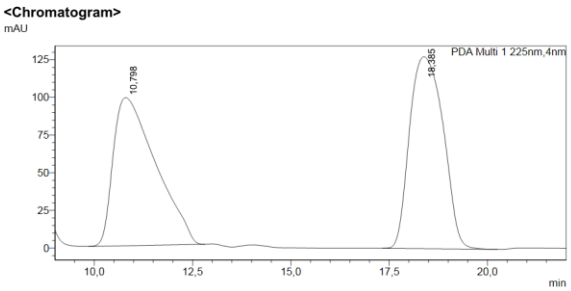

<Peak Table>
PDA Ch1 225nm

Peak#	Ret. Time	Area	Area%
1	10,798	7416426	49,422
2	18,385	7589820	50,578
Total		15006247	100,000

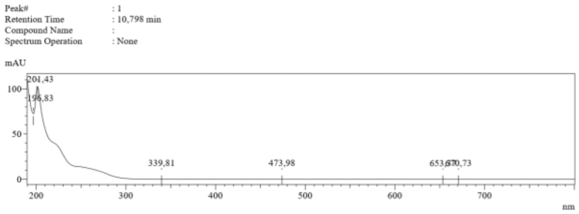

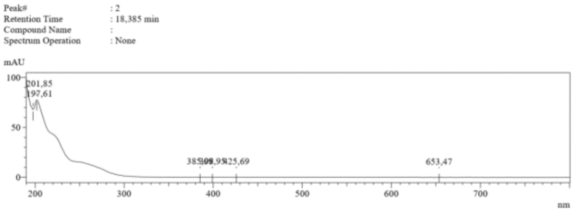

Ethyl (*R*)-3-(4-chlorophenyl)-3-(diphenylphosphaneyl)propanoate (2'e):

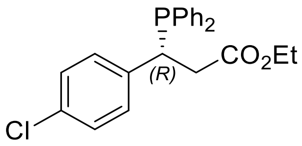

2'e

Compound **2'e** was synthesized following **general procedure 1** with (*E*)-**2e** or (*Z*)-**2e** (21 mg, 0.1 mmol, 1.0 equiv), Ph_2PH (17 µL, 0.1 mmol, 1.0 equiv), (R_C,S_P)-Mn(I) (1.5 mg, 0.002 mmol, 0.02 equiv), *t*PentOK (0.042 M, 95 µL, 0.004 mmol, 0.04 equiv), in dry, deoxygenated toluene (1 mL). Pentane (2 mL) was added and the resulting mixture was filtered through a plug of alumina in order to remove the catalyst. EtOAc (1 mL) was added to flush the plug. Product **2'e** was obtained as a white solid after evaporation of the solvent under reduced pressure. [(*E*)-**2e**, 90% yield, 68:32 er], [(*Z*)-**2e**, 95% yield, >99.9:0.1 er].

^{1}H NMR (400 MHz, $CDCl_3$): δ 7.68–7.65 (m, 2H), 7.49–7.44 (m, 4H), 7.19–7.09 (m, 8H), 4.04–3.92 (m, 3H), 2.82–2.74 (m, 1H), 2.71–2.65 (m, 1H), 1.08 (t, *J* = 7.1 Hz, 3H) ppm.

^{13}C NMR (151 MHz, $CDCl_3$): δ 171.5 (d, *J* = 16.2 Hz), 143.1, 138.6 (d, *J* = 8.7 Hz), 135.6 (d, *J* = 14.0 Hz), 135.3 (d, *J* = 16.2 Hz), 134.0 (d, *J* = 20.9 Hz), 133.1 (d, *J* = 18.7 Hz), 130.1 (d, *J* = 7.2 Hz), 129.6, 129.1 (d, *J* = 5.7 Hz), 128.8, 128.7, 128.3, 128.1 (d, *J* = 7.0 Hz), 118.8, 60.5, 40.5 (d, *J* = 13.3 Hz), 38.2 (d, *J* = 22.7 Hz), 14.0 ppm.

^{31}P NMR (243 MHz, $CDCl_3$): δ -0.1 ppm.

HRMS (ESI+, m/z) calc. for $C_{23}H_{23}ClO_3P$ (oxidized product): $[M+H]^+$ = 413.1068, found $[M+H]^+$ = 413.1064.

SFC: (209 nm, Waters Trefoil CEL1, MeOH, 40 °C, 1.8 mL/min), t_R = 7.1 min (minor), t_R = 7.2 min (major). **The peak at 7.2 min present in the left chromatogram has a different UV/Vis spectrum than that of **2'e**.*

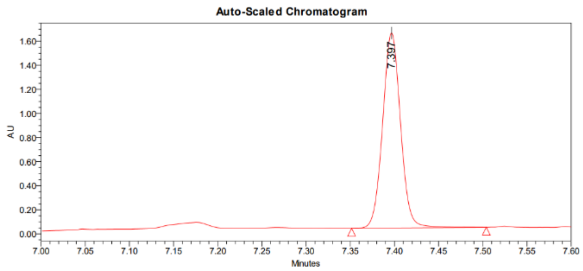

Peak Results

	Name	RT	Area	% Area	Height
1		7.397	2286400	100.00	1620278

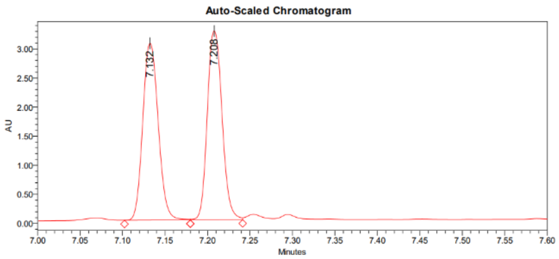

Peak Results

	Name	RT	Area	% Area	Height
1		7.132	3842444	49.53	3041651
2		7.208	3914836	50.47	3246110

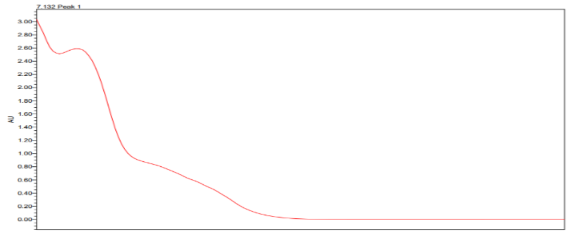

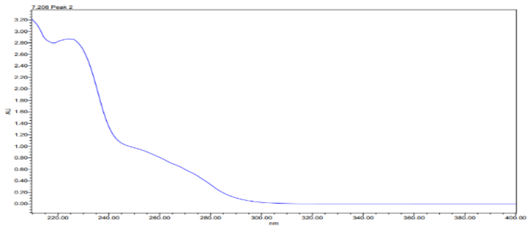

Ethyl (*R*)-3-(3-chlorophenyl)-3-(diphenylphosphaneyl)propanoate (2'f):

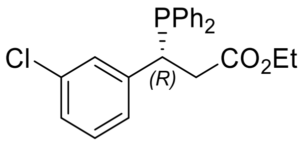

2'f

Compound **2'f** was synthesized following **general procedure 1** with (*E*)-**2f** or (*Z*)-**2f** (21 mg, 0.1 mmol, 1.0 equiv), Ph_2PH (17 µL, 0.1 mmol, 1.0 equiv), (R_C,S_P)-Mn(I) (1.5 mg, 0.002 mmol, 0.02 equiv), *t*PentOK (0.042 M, 95 µL, 0.004 mmol, 0.04 equiv), in dry, deoxygenated toluene (1 mL). Pentane (2 mL) was added and the resulting mixture was filtered through a plug of alumina in order to remove the catalyst. EtOAc (1 mL) was added to flush the plug. Product **2'f** was obtained as a white solid after evaporation of the solvent under reduced pressure. [(*E*)-**2f**, 99% yield, 72:28 er], [(*Z*)-**2f**, 99% yield, 98.5:1.5 er].

^{1}H NMR (600 MHz, $CDCl_3$): δ 7.63–7.49 (m, 2H), 7.41–7.27 (m, 3H), 7.19–6.95 (m, 9H), 3.96–3.82 (m, 3H), 2.76–2.65 (m, 1H), 2.63–2.52 (m, 1H), 0.99–0.97 (m, 3H) ppm.

^{13}C NMR (151 MHz, $CDCl_3$): δ 171.4 (d, *J* = 15.9 Hz), 142.1 (d, *J* = 8.7 Hz), 135.5 (d, *J* = 14.2 Hz), 135.2 (d, *J* = 16.0 Hz), 134.0 (d, *J* = 20.7 Hz), 133.9, 133.1 (d, *J* = 18.6 Hz), 129.6, 129.3, 128.9 (d, *J* = 7.5 Hz), 128.8, 128.7 (d, *J* = 7.6 Hz), 128.0 (d, *J* = 6.8 Hz), 127.1 (d, *J* = 7.1 Hz), 126.6, 60.5, 40.8 (d, *J* = 14.2 Hz), 38.1 (d, *J* = 22.4 Hz), 14.0 ppm.

^{31}P NMR (243 MHz, $CDCl_3$): δ 0.3 ppm.

HRMS (ESI+, m/z) calc. for $C_{23}H_{23}ClO_3P$ (oxidized product): $[M+H]^+$ = 413.1068, found $[M+H]^+$ = 413.1067.

SFC: (209 nm, Waters Trefoil CEL1, MeOH, 40 °C, 1.8 mL/min), t_R = 11.8 min (minor), t_R = 11.9 min (major).

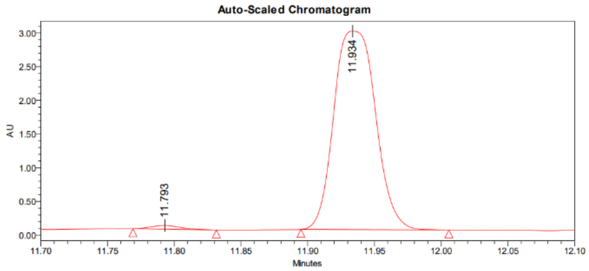

Peak Results

	Name	RT	Area	% Area	Height
1		11.793	83967	1.30	54849
2		11.934	6399410	98.70	2944044

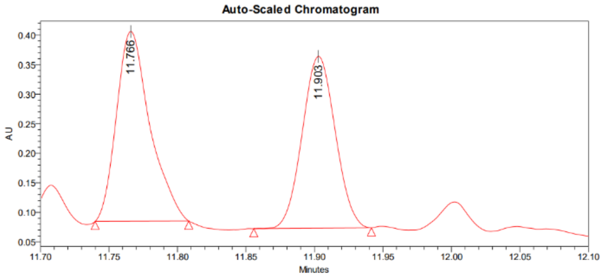

Peak Results

	Name	RT	Area	% Area	Height
1		11.766	546366	52.80	321215
2		11.903	488438	47.20	290845

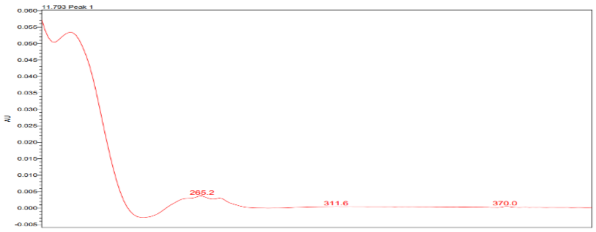

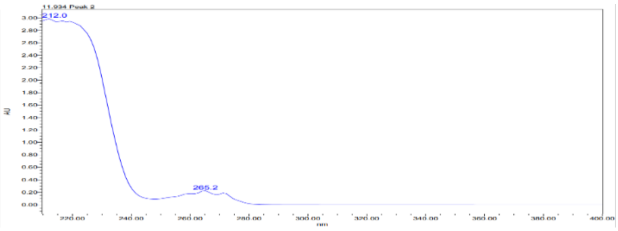

Ethyl (*R*)-3-(diphenylphosphoryl)-3-(quinolin-2-yl)propanoate (2’g):

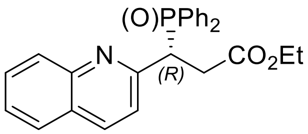

2’g

Compound **2’g** was synthesized following **general procedure 1** with (*E*)-**2g** (23 mg, 0.1 mmol, 1.0 equiv), Ph_2PH (17 µL, 0.1 mmol, 1.0 equiv), (R_C,S_P)-Mn(I) (1.5 mg, 0.002 mmol, 0.02 equiv), *t*PentOK (0.042 M, 95 µL, 0.004 mmol, 0.04 equiv), in dry, deoxygenated toluene (1 mL). An aqueous solution of H_2O_2 (30%, 0.25 mmol, 2.5 equiv) was used to oxidize the product which was crashed out of the solution by additing Et_2O. Product **2’g** was obtained after filtration and washing with Et_2O as a brown solid. [(*E*)-**2g**, 90% yield, 94:6 er].

^{1}H NMR (600 MHz, $CDCl_3$): δ 7.94–7.91 (m, 2H), 7.87–7.82 (m, 2H), 7.73–7.68 (m, 3H), 7.65–7.60 (m, 1H), 7.54–7.50 (m, 1H), 7.47–7.43 (m, 3H), 7.38–7.34 (m, 2H), 7.30–7.26 (m, 2H), 4.65–4.58 (m, 1H), 3.94–3.86 (m, 2H), 3.57–3.48 (m, 1H), 3.06– 2.98 (m, 1H), 0.99 (t, J = 7.1 Hz, 3H) ppm.

^{13}C NMR (151 MHz, $CDCl_3$): δ 171.4, 171.2, 156.0, 155.9, 147.6, 136.0, 132.0 (2 x C), 131.8 (2 x C), 131.7, 131.6, 131.5, 131.4, 129.3, 129.1, 128.7, 128.6, 128.2, 128.1, 127.4 (2 x C), 126.3, 122.2 (2 x C), 60.7, 47.2, 46.6, 33.4, 14.0 ppm. Due to complex splitting of the carbon signals in the aromatic region, peaks are reported only by their chemical shift.

^{31}P NMR (243 MHz, $CDCl_3$): δ 32.5 ppm.

HRMS (ESI+, m/z) calc. for $C_{26}H_{24}NO_3PNa$: $[M+Na]^+$ = 452.1386, found $[M+Na]^+$ = 452.1388.

CSP-HPLC: (254 nm, Chiralcel OD-H, *n*-heptane:*i*PrOH = 80:20, 40 °C, 0.5 mL/min), t_R = 8.5 min (minor), t_R = 8.9 min (major).

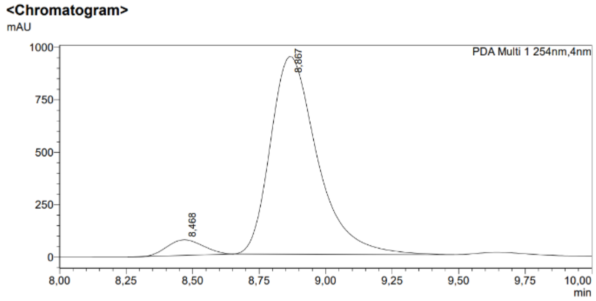

<Peak Table>
PDA Ch1 254nm

Peak#	Ret. Time	Area	Height	Area%
1	8,468	704051	74179	5,736
2	8,867	11569168	942515	94,264
Total		12273219	1016694	100,000

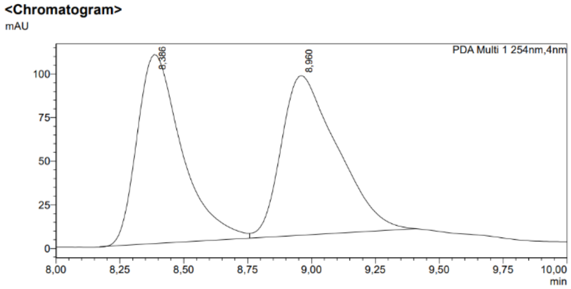

<Peak Table>
PDA Ch1 254nm

Peak#	Ret. Time	Area%
1	8,386	48,697
2	8,960	51,303
Total		100,000

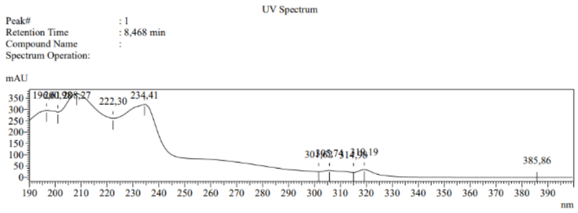

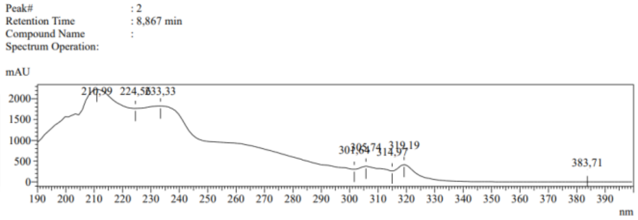

Ethyl (*R*)-3-(diphenylphosphaneyl)-3-(pyridin-2-yl)propanoate (2’h):

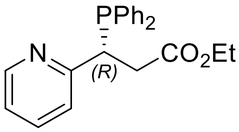

2’h

Compound **2’h** was synthesized following **general procedure 1** with (*E*)-**2h** (18 mg, 0.1 mmol, 1.0 equiv), Ph_2PH (17 µL, 0.1 mmol, 1.0 equiv), (R_C,S_P)-Mn(I) (1.5 mg, 0.002 mmol, 0.02 equiv), *t*PentOK (0.042 M, 95 µL, 0.004 mmol, 0.04 equiv), in toluene (1 mL). Pentane (2 mL) was added and the resulting mixture was filtered through a plug of alumina in order to remove the

catalyst. EtOAc (1 mL) was added to flush the plug. Product **2’h** was obtained as a white solid after evaporation of the solvent under reduced pressure. [(*E*)-**2h**, 99% yield, >99.9:0.1 er].

^{1}H NMR (600 MHz, $CDCl_3$): δ 8.52–8.52 (m, 1H), 7.68–7.64 (m, 2H), 7.44–7.33 (m, 4H), 7.19–7.13 (m, 5H), 7.02–7.00 (m, 1H), 6.90 (d, *J* = 6.7 Hz, 1H), 4.20–4.19 (m, 1H), 3.99–3.89 (m, 2H), 3.28–3.22 (m, 1H), 2.68–2.63 (m, 1H), 1.07 (t, *J* = 7.1 Hz, 3H) ppm.

^{13}C NMR (151 MHz, $CDCl_3$): δ 172.1 (d, *J* = 16.4 Hz), 159.8 (d, *J* = 5.8 Hz), 149.1, 136.0 (d, *J* = 18.1 Hz), 135.6 (d, *J* = 17.5 Hz), 133.8 (d, *J* = 18.1 Hz), 133.3 (d, *J* = 19.4 Hz), 129.4, 128.7, 128.6 (d, *J* = 7.2 Hz), 128.5 (d, *J* = 11.7 Hz), 128.0 (d, *J* = 6.9 Hz), 124.4 (d, *J* = 4.4 Hz), 121.2, 60.4, 43.0 (d, *J* = 13.5 Hz), 36.7 (d, *J* = 21.9 Hz), 14.0 ppm.

^{31}P NMR (243 MHz, $CDCl_3$): δ 0.4 ppm.

HRMS (ESI+, m/z) calc. for $C_{22}H_{23}NO_2P$: $[M+H]^+$ = 364.1466, found $[M+H]^+$ = 364.1463.

CSP-HPLC: (254 nm, Chiralcel OD-H, *n*-heptane:*i*PrOH = 99:1, 40 °C, 0.5 mL/min), t_R = 12.1 min (major), t_R = 15.8 min (minor).

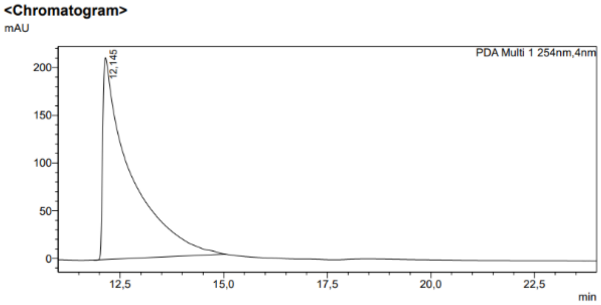

<Peak Table>
PDA Ch1 254nm

Peak#	Ret. Time	Area%
1	12,145	100,000
Total		100,000

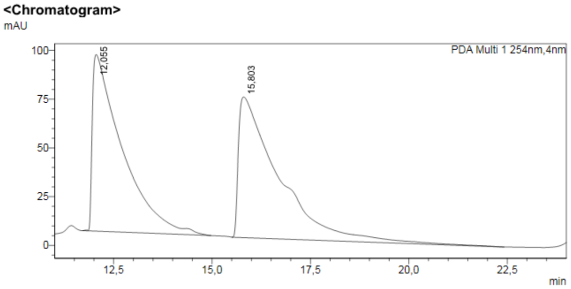

<Peak Table>
PDA Ch1 254nm

Peak#	Ret. Time	Area	Area%
1	12,055	4747894	48,128
2	15,803	5117144	51,872
Total		9865037	100,000

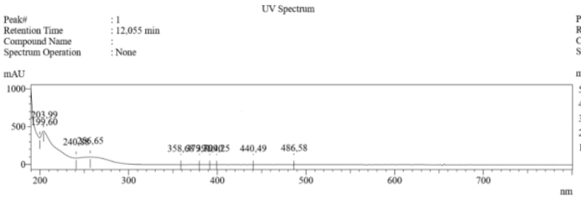

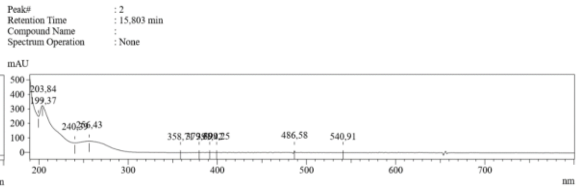

4-(Diphenylphosphaneyl)tetrahydro-2*H*-pyran-2-one (2’i):

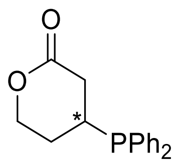

2’i

Compound **2’i** was synthesized following **general procedure 1** with **2i** (10 mg, 0.1 mmol, 1.0 equiv), Ph_2PH (17 µL, 0.1 mmol, 1.0 equiv), (R_C,S_P)-Mn(I) (1.5 mg, 0.002 mmol, 0.02 equiv), *t*PentOK (0.042 M, 95 µL, 0.004 mmol, 0.04 equiv), in dry, deoxygenated toluene (1 mL) at 0 °C. Pentane (2 mL) was added and the resulting mixture was filtered through a plug of alumina in order to remove the catalyst. EtOAc (1 mL) was added to flush the plug. Product **2’i** was obtained as a colorless oil after evaporation of the solvent under reduced pressure. [99% yield, 90.5:9.5 er].

^{1}**H NMR** (600 MHz, $CDCl_3$): δ 7.51–7.46 (m, 4H), 7.38–7.35 (m, 6H), 4.44–4.40 (m, 1H), 4.32–4.28 (m, 1H), 2.82–2.76 (m, 1H), 2.56 (dt, J = 17.3, 5.3 Hz, 1H), 2.44–2.37 (m, 1H), 2.00–1.94 (m, 1H), 1.82–1.74 (m, 1H) ppm.

^{13}**C NMR** (151 MHz, $CDCl_3$): δ 170.5 (d, *J* = 12.4 Hz), 135.0 (d, *J* = 13.1 Hz), 134.5 (d, *J* = 13.3 Hz), 133.6, 133.4 (d, *J* = 19.7 Hz), 133.2, 129.5 (d, *J* = 19.5 Hz), 128.8 (d, *J* = 5.7 Hz), 128.7 (d, *J* = 5.5 Hz), 68.4 (d, *J* = 8.7 Hz), 32.9 (d, *J* = 18.5 Hz), 28.3 (d, *J* = 11.6 Hz), 26.1 (d, *J* = 16.3 Hz) ppm.

^{31}**P NMR** (243 MHz, $CDCl_3$): δ -5.4 ppm.

HRMS (ESI+, m/z) calc. for $C_{17}H_{18}O_2P$: $[M+H]^+$ = 285.1039, found $[M+H]^+$ = 285.1036.

SFC: (221 nm, chiralpak IC column, MeOH, 40 °C, 4 mL/min), t_R = 6.1 min (major), t_R = 11.4 min (minor).

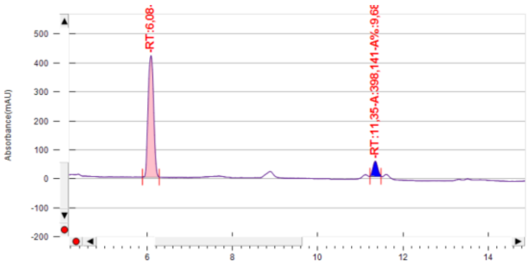

Peak Information

Peak No	% Area	Area	Ret. Time
1	90,3153	3712,8801	6,08 min
2	9,6847	398,141	11,35 min

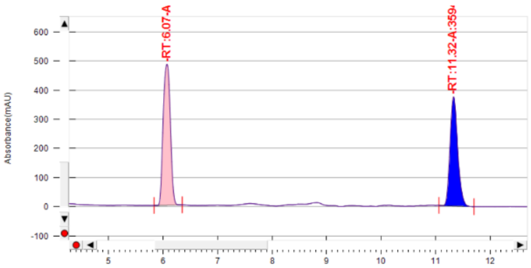

Peak Information

Peak No	% Area	Area	Ret. Time
1	54.8388	4364.9062	6.07 min
2	45.1612	3594.6188	11.32 min

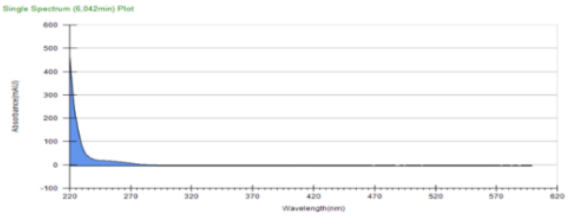

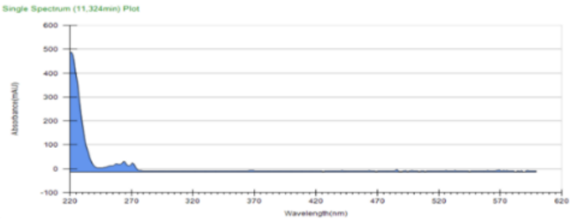

(*R*)-3-(Diphenylphosphaneyl)-*N*,*N*-dimethylbutanamide (3'a):

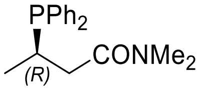

3'a

Compound **3'a** was synthesized following **general procedure 1** with (*E*)-**3a** (11 mg, 0.1 mmol, 1.0 equiv), Ph_2PH (17 µL, 0.1 mmol, 1.0 equiv), (R_C,S_P)-Mn(I) (1.5 mg, 0.002 mmol, 0.02 equiv), *t*PentOK (0.042 M, 95 µL, 0.004 mmol, 0.04 equiv), in dry, deoxygenated toluene (1 mL). Pentane (2 mL) was added and the resulting mixture was filtered through a plug of alumina in order to remove the catalyst. EtOAc (1 mL) was added to flush the plug. Product **3'a** was obtained as a colorless oil after evaporation of the solvent under reduced pressure. [(*E*)-**3a**, 99% yield, 81:19 er].

^{1}H NMR (600 MHz, $CDCl_3$): δ 7.56–7.52 (m, 4H), 7.35–7.31 (m, 6H), 3.05–3.02 (m, 1H), 2.91 (s, 3H), 2.87 (s, 3H), 2.37–2.33 (m, 1H), 2.31–2.25 (m, 1H), 1.07 (dd, *J* = 15.2, 6.6 Hz, 3H) ppm.

^{13}C NMR (151 MHz, $CDCl_3$): δ 171.5 (d, *J* = 15.0 Hz), 136.6 (d, *J* = 11.7 Hz), 136.5, 133.7 (d, *J* = 12.3 Hz), 133.5 (d, *J* = 12.2 Hz), 128.9 (2 x C), 128.5 (d, *J* = 7.1 Hz), 128.4 (d, *J* = 7.1 Hz), 37.2, 36.7 (d, *J* = 20.1 Hz), 35.5, 26.7 (d, *J* = 7.5 Hz), 17.1 (d, *J* = 16.5 Hz) ppm.

^{31}P NMR (243 MHz, $CDCl_3$): δ -1.2 ppm.

HRMS (ESI+, m/z) calc. for $C_{18}H_{23}NOP$: $[M+H]^+$ = 300.1512, found $[M+H]^+$ = 300.1517.

CSP-HPLC: (254 nm, Chiralcel OJ-H, *n*-heptane:*i*PrOH = 90.10, 40 °C, 0.5 mL/min), t_R = 17.1 min (minor), t_R = 23.3 min (major).

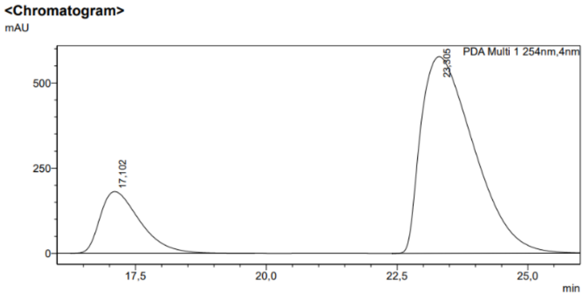

<Peak Table>

PDA Ch1 254nm

Peak#	Ret. Time	Area%
1	17,102	19,144
2	23,305	80,856
Total		100,000

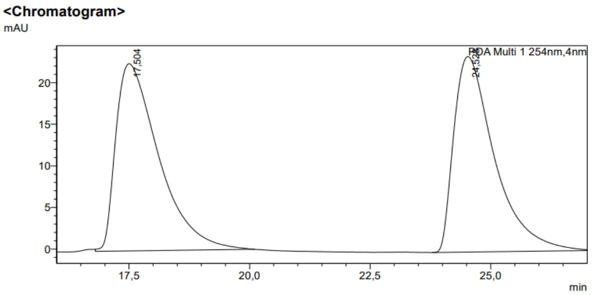

<Peak Table>

PDA Ch1 254nm

Peak#	Ret. Time	Area%
1	17,504	50,285
2	24,528	49,715
Total		100,000

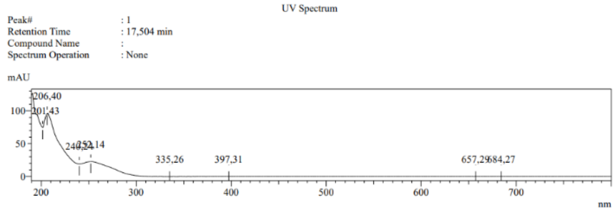

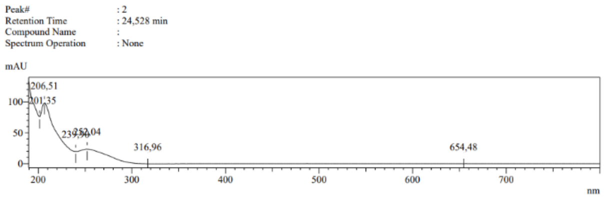

(*R*)-3-(Diphenylphosphaneyl)-*N*,*N*-dimethyl-3-phenylpropanamide (3'b):

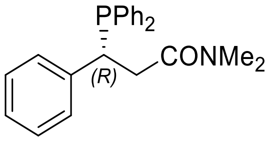

3'b

Compound **3'b** was synthesized following **general procedure 1** with (*E*)-**3b** or (*Z*)-3**b** (18 mg, 0.1 mmol, 1.0 equiv), Ph_2PH (17 µL, 0.1 mmol, 1.0 equiv), (R_C,S_P)-Mn(I) (1.5 mg, 0.002 mmol, 0.02 equiv), *t*PentOK (0.042 M, 95 µL, 0.004 mmol, 0.04 equiv), in dry, deoxygenated toluene (1 mL). Pentane (2 mL) was added and the resulting mixture was filtered through a plug of alumina in order to remove the catalyst. EtOAc (1 mL) was added to flush the plug. Product **3'b** was obtained as a white solid after evaporation of the solvent under reduced pressure. [(*E*)-**3b**, 99% yield, 71:29 er], [(*Z*)-**3b**, 99% yield, 98.5:1.5 er].

^{1}H NMR (600 MHz, $CDCl_3$): δ 7.73–7.67 (m, 2H), 7.42–7.38 (m, 3H), 7.22–7.21 (m, 2H), 7.16–7.14 (m, 3H), 7.10–7.08 (m, 5H), 4.26–4.21 (m, 1H), 3.02– 2.96 (m, 1H), 2.77 (s, 6H), 2.58–2.52 (m, 1H) ppm.

^{13}C NMR (151 MHz, $CDCl_3$): δ 140.9 (d, *J* = 8.6 Hz), 136.6 (d, *J* = 13.6 Hz), 136.2 (d, *J* = 15.3 Hz), 134.0 (d, *J* = 20.3 Hz), 133.2 (d, *J* = 18.2 Hz), 129.3, 129.1, 129.0, 128.7 (d, *J* = 7.3 Hz), 128.4, 128.2, 127.8 (d, *J* = 7.0 Hz), 126.2, 40.9 (d, *J* = 10.2 Hz), 37.1, 36.9 (d, *J* = 23.0 Hz), 35.4 ppm.

^{31}P NMR (243 MHz, $CDCl_3$): δ -1.1 ppm.

HRMS (ESI+, m/z) calc. for $C_{23}H_{24}NO_2PNa$ (oxidized product): $[M+Na]^+$ = 400.1437, found $[M+Na]^+$ = 400.1437.

CSP-HPLC: (254 nm, Chiralcel AD-H, *n*-heptane:*i*PrOH = 98:2, 40 °C, 0.5 mL/min), t_R = 21.3 min (minor), t_R = 24.1 min (major).

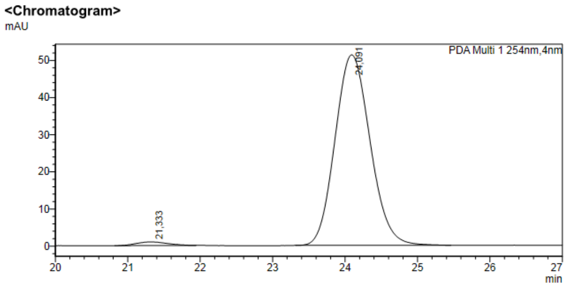

<Peak Table>
PDA Ch1 254nm

Peak#	Ret. Time	Area	Area%
1	21,333	28526	1,625
2	24,091	1726718	98,375
Total		1755244	100,000

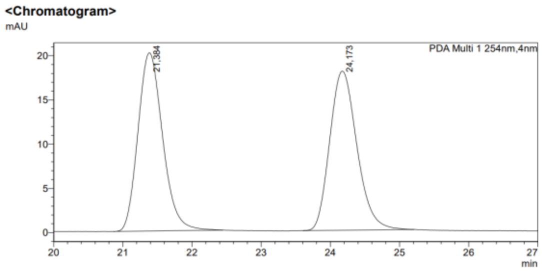

<Peak Table>
PDA Ch1 254nm

Peak#	Ret. Time	Area	Area%
1	21,384	500743	50,394
2	24,173	492917	49,606
Total		993660	100,000

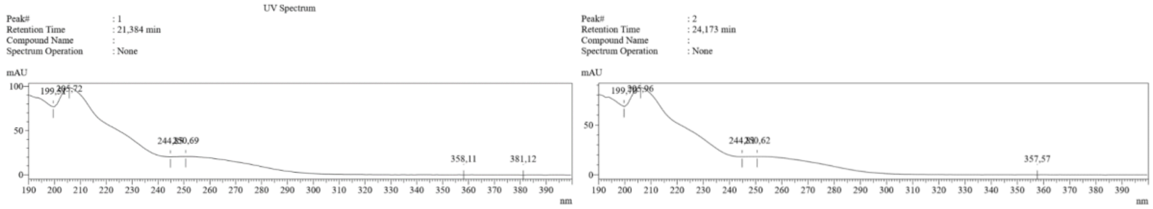

(*R*)-3-(Diphenylphosphaneyl)-3-(4-methoxyphenyl)-*N*,*N*-dimethylpropanamide (3’c):

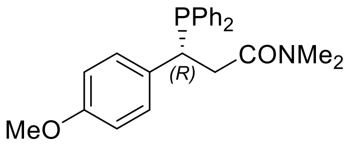

3’c

Compound **3’c** was synthesized following **general procedure 1** with (*E*)-**3c** or (*Z*)-**3c** (21 mg, 0.1 mmol, 1.0 equiv), Ph_2PH (17 µL, 0.1 mmol, 1.0 equiv), (R_C,S_P)-Mn(I) (3.0 mg, 0.004 mmol, 0.04 equiv), *t*PentOK (0.042 M, 190 µL, 0.008 mmol, 0.08 equiv), in dry, deoxygenated toluene (1 mL). Pentane (2 mL) was added and the resulting mixture was filtered through a plug of alumina in order to remove the catalyst. EtOAc (1 mL) was added to flush the plug. Product **3’g** was obtained as a white solid after short column chromatography (SiO_2, Pentane). [(*E*)**-3c**, 80% yield, 69:31 er], [(*Z*)**-3c,** 40% yield, >99.9:0.1 er].

^{1}H NMR (600 MHz, $CDCl_3$): δ 7.76–7.66 (m, 2H), 7.47–7.37 (m, 3H), 7.21–7.13 (m, 7H), 6.73 (d, *J* = 5.9 Hz, 2H), 4.24–4.17 (m, 1H), 3.76 (s, 3H), 3.00– 2.71 (m, 7H), 2.58–2.47 (m, 1H) ppm.

^{13}C NMR (151 MHz, $CDCl_3$): δ 158.0, 136.8 (d, *J* = 13.8 Hz), 136.4 (d, *J* = 16.2 Hz), 134.0 (d, *J* = 20.2 Hz), 133.2 (d, *J* = 18.1 Hz), 132.9 (d, *J* = 9.1 Hz), 130.0 (d, *J* = 7.6 Hz), 129.3, 129.0, 128.7 (d, *J* = 7.0 Hz), 128.4, 127.8 (d, *J* = 6.6 Hz), 113.6, 55.1, 40.0, 37.2, 35.4 ppm.

^{31}P NMR (243 MHz, $CDCl_3$): δ -1.9 ppm.

HRMS (ESI+, m/z) calc. for $C_{24}H_{26}NO_3PNa$ (oxidized product): $[M+Na]^+$ = 430.1542, found $[M+Na]^+$ = 430.1548.

CSP-HPLC: (254 nm, Chiralcel AD-H, *n*-heptane:*i*PrOH = 90:10, 40 °C, 0.5 mL/min), t_R = 28.0 min (minor), t_R = 39.6 min (major).

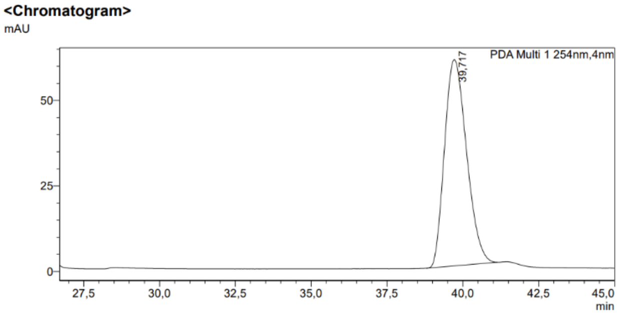

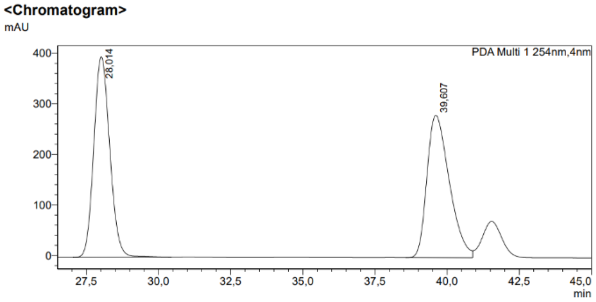

<Peak Table>

PDA Ch1 254nm

Peak#	Ret. Time	Area	Area%
1	39,717	3063146	100,000
Total		3063146	100,000

<Peak Table>

PDA Ch1 254nm

Peak#	Ret. Time	Area%
1	28,014	50,128
2	39,607	49,872
Total		100,000

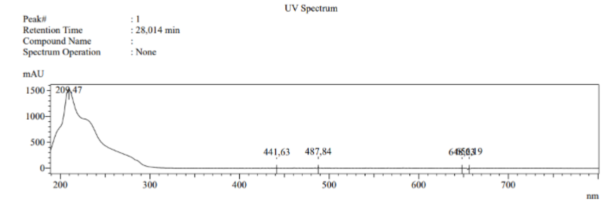

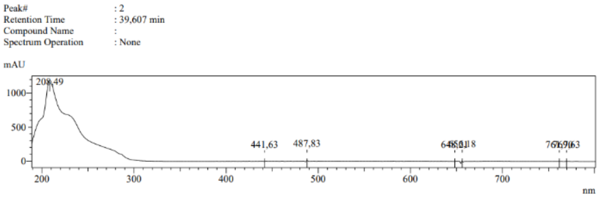

(*R*)-3-(4-Bromophenyl)-3-(diphenylphosphaneyl)-*N*,*N*-dimethylpropanamide (3’d):

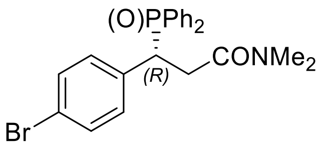

3’d

Compound **3’d** was synthesized following **general procedure 1** with (*E*)-**3d** or (*Z*)-**3d** (25 mg, 0.1 mmol, 1.0 equiv), Ph_2PH (17 µL, 0.1 mmol, 1.0 equiv), (R_C,S_P)-Mn(I) (3.0 mg, 0.004 mmol,

0.04 equiv), *t*PentOK (0.042 M, 190 µL, 0.008 mmol, 0.08 equiv), in dry, deoxygenated toluene (1 mL). An aqueous solution of H_2O_2 (30%, 0.25 mmol, 2.5 equiv) was used to oxidize the product which was crashed out of the solution by additing Et_2O. Product **3'd** was obtained after filtration and washing with Et_2O as a white solid. [(*E*)**-3d**, 99% yield, 87:12 er], [(*Z*)**-3d,** 99% yield, 88.5:11.5 er].

^{1}H NMR (400 MHz, $CDCl_3$): δ 7.99–7.94 (m, 2H), 7.57–7.51 (m, 3H), 7.47–7.43 (m, 2H), 7.35–7.32 (m, 1H), 7.29–7.24 (m, 6H), 4.36–4.31 (m, 1H), 3.18–3.11 (m, 1H), 2.82 (s, 3H), 2.76–2.71 (m, 4H) ppm.

^{13}C NMR (101 MHz, $CDCl_3$): δ 169.6 (d, *J* = 14.2 Hz), 135.4 (d, *J* = 4.6 Hz), 132.1, 131.7, 131.5 (2 x C), 131.4, 131.3, 131.2, 131.0, 130.8, 130.7, 129.0 (d, *J* = 11.2 Hz), 128.2 (d, *J* = 11.8 Hz), 121.1, 41.7 (d, *J* = 69.0 Hz), 37.1, 35.7, 33.6 ppm.

^{31}P NMR (162 MHz, $CDCl_3$): δ 34.1ppm.

HRMS (ESI+, m/z) calc. for $C_{23}H_{23}BrNO_2PNa$ (oxidized product): $[M+Na]^+$ = 478.0525, found $[M+Na]^+$ = 478.0542.

CSP-HPLC: (207 nm, Chiralcel AD-H, *n*-heptane:*i*PrOH = 80:20, 40 °C, 1 mL/min), t_R = 17.7 min (minor), t_R = 29.3 min (major).

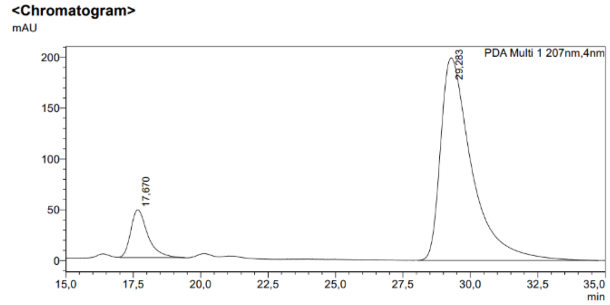

<Peak Table>
PDA Ch1 207nm

Peak#	Ret. Time	Area%
1	17,670	11,367
2	29,283	88,633
Total		100,000

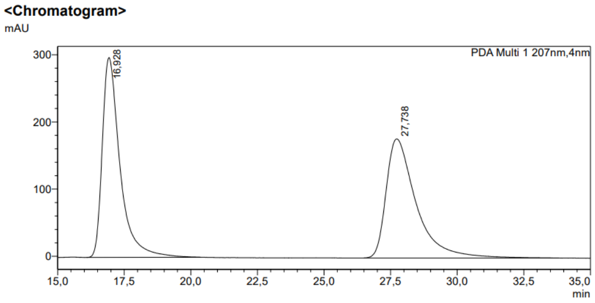

<Peak Table>
PDA Ch1 207nm

Peak#	Ret. Time	Area%
1	16,928	49,723
2	27,738	50,277
Total		100,000

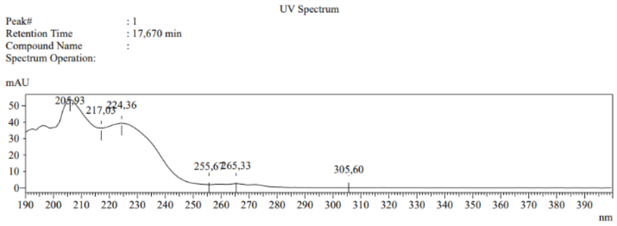

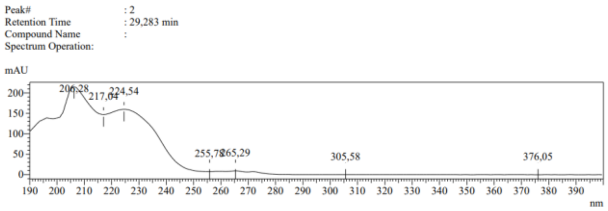

(*R*)-3-(3-Bromophenyl)-3-(diphenylphosphaneyl)-*N*,*N*-dimethylpropanamide (3'e):

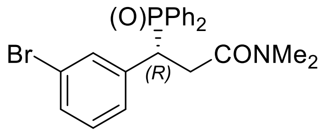

3'e

Compound **3'e** was synthesized following **general procedure 1** with (*E*)-**3e** or (*Z*)-**3e** (25 mg, 0.1 mmol, 1.0 equiv), Ph_2PH (17 µL, 0.1 mmol, 1.0 equiv), (R_C,S_P)-Mn(I) (3.0 mg, 0.004 mmol, 0.04 equiv), *t*PentOK (0.042 M, 190 µL, 0.008 mmol, 0.08 equiv), in dry, deoxygenated toluene (1 mL). An aqueous solution of H_2O_2 (30%, 0.25 mmol, 2.5 equiv) was used to oxidize the product which was crashed out of the solution by additing Et_2O. Product **3'e** was obtained after filtration and washing with Et_2O as a white solid. [(*E*)**-3e**, 99% yield, 54:46 er], [(*Z*)**-3e,** 99% yield, 52.5:47.5 er].

^{1}H NMR (400 MHz, $CDCl_3$): δ 7.99–7.95 (m, 2H), 7.55 (br s [m], 3H), 7.48–7.41 (m, 3H), 7.36–7.24 (m, 5H), 7.04–7.00 (m, 1H), 4.34–4.31 (m, 1H), 3.18–3.11 (m, 1H), 2.84 (s, 3H), 2.81–2.74 (m, 4H) ppm.

^{13}C NMR (101 MHz, $CDCl_3$): δ 169.5 (d, *J* = 14.0 Hz), 138.7 (d, *J* = 4.5 Hz), 132.7 (d, *J* = 4.8 Hz), 132.1, 131.6, 131.3 (d, *J* = 8.1 Hz), 130.8 (d, *J* = 8.6 Hz), 130.2, 129.7, 129.0, 128.9, 128.5 (d, *J* = 4.6 Hz), 128.2, 128.1, 122.2, 42.1 (d, *J* = 68.3 Hz), 37.1, 35.7, 33.5 ppm.

^{31}P NMR (162 MHz, $CDCl_3$): δ 34.1 ppm.

HRMS (ESI+, m/z) calc. for $C_{23}H_{24}BrNO_2P$ (oxidized product): $[M+H]^+$ = 456.0723, found $[M+H]^+$ = 456.0717.

CSP-HPLC: (207 nm, Chiralcel OD-H, *n*-heptane:*i*PrOH = 80.20, 40 °C, 1 mL/min), t_R = 5.1 min (minor), t_R = 5.6 min (major).

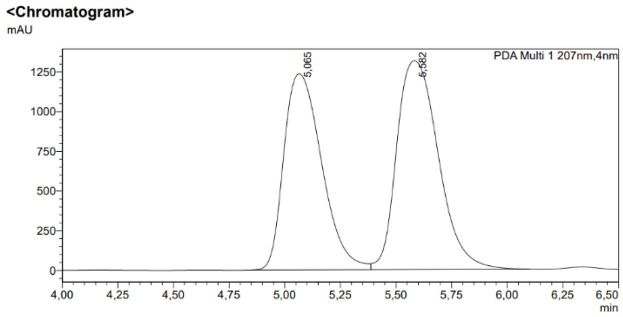

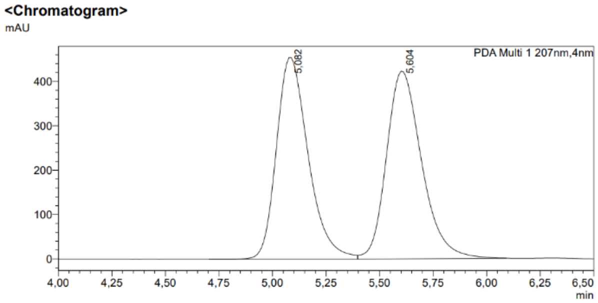

<Peak Table>

PDA Ch1 207nm

Peak#	Ret. Time	Area%
1	5,065	46,127
2	5,582	53,873
Total		100,000

<Peak Table>

PDA Ch1 207nm

Peak#	Ret. Time	Area%
1	5,082	49,351
2	5,604	50,649
Total		100,000

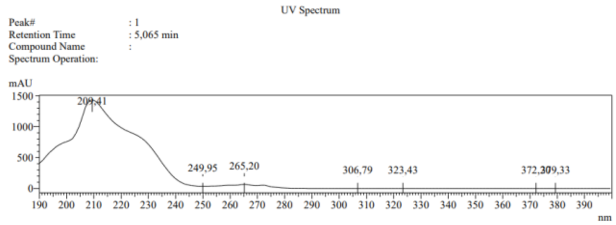

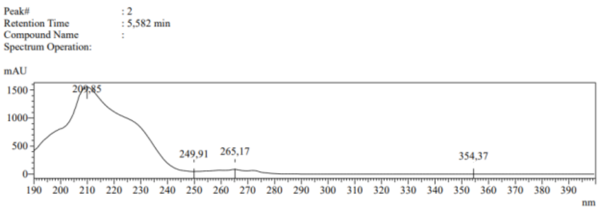

(*R*)-3-(2-Bromophenyl)-3-(diphenylphosphaneyl)-*N*,*N*-dimethylpropanamide (3’f):

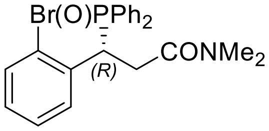

3’f

Compound **3’f** was synthesized following **general procedure 1** with (*E*)-**3f** or (*Z*)-**3f** (25 mg, 0.1 mmol, 1.0 equiv), Ph_2PH (17 µL, 0.1 mmol, 1.0 equiv), (R_C,S_P)-Mn(I) (3.0 mg, 0.004 mmol, 0.04 equiv), *t*PentOK (0.042 M, 190 µL, 0.008 mmol, 0.08 equiv), in dry, deoxygenated toluene (1 mL). An aqueous solution of H_2O_2 (30%, 0.25 mmol, 2.5 equiv) was used to oxidize the product which was crashed out of the solution by additing Et_2O. Product **3’f** was obtained after filtration and washing with Et_2O as a white solid. [(*E*)**-3f**, 80% yield, 66:34 er], [(*Z*)**-3f**, 80% yield, 71.5:28.5 er].

^{1}H NMR (400 MHz, $CDCl_3$): δ 8.09–8.04 (m, 2H), 7.82 (d, *J* = 7.6 Hz, 1H), 7.62–7.59 (m, 3H), 7.36–7.28 (m, 5H), 7.19–7.15 (m, 2H), 7.01–6.98 (m, 1H), 5.03–5.01 (m, 1H), 3.28–3.17 (m, 1H), 2.90–2.85 (m, 4H), 2.75 (s, 3H) ppm.

^{13}C NMR (101 MHz, $CDCl_3$): δ 169.5 (d, *J* = 13.7 Hz), 136.1 (d, *J* = 4.1 Hz), 132.8, 132.2, 131.6, 131.5, 131.1 (d, *J* = 9.5 Hz), 130.1, 130.0, 129.6, 128.9 (d, *J* = 11.2 Hz), 128.4, 128.3, 127.8 (d, *J* = 12.0 Hz), 127.5, 40.9 (d, *J* = 67.6 Hz), 36.5 (d, *J* = 158.6 Hz), 33.9, 30.3 ppm.

^{31}P NMR (162 MHz, $CDCl_3$): δ 34.0 ppm.

HRMS (ESI+, m/z) calc. for $C_{23}H_{24}BrNO_2P$ (oxidized product): $[M+H]^+$ = 456.0723, found $[M+H]^+$ = 456.0721.

CSP-HPLC: (207 nm, Chiralcel OD-H, *n*-heptane:*i*PrOH = 90:10, 40 °C, 1 mL/min), t_R = 12.8 min (minor), t_R = 18.5 min (major).

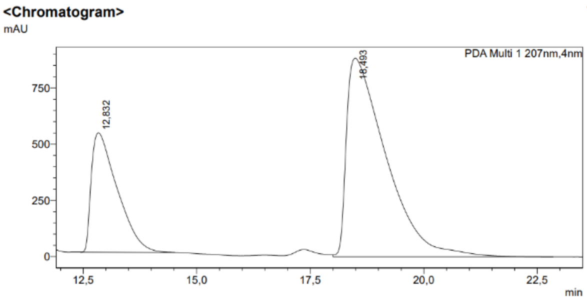

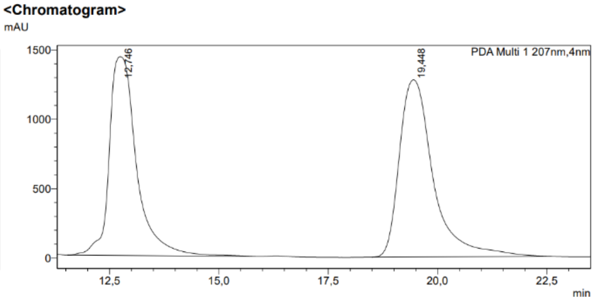

<Peak Table>
PDA Ch1 207nm

Peak#	Ret. Time	Area%
1	12,832	28,294
2	18,493	71,706
Total		100,000

<Peak Table>
PDA Ch1 207nm

Peak#	Ret. Time	Area%
1	12,746	49,477
2	19,448	50,523
Total		100,000

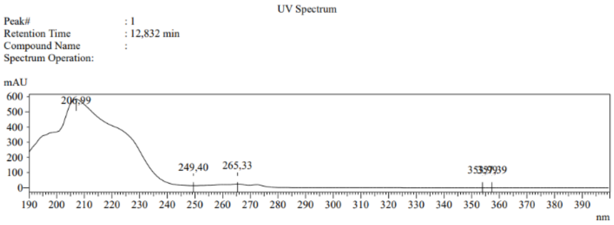

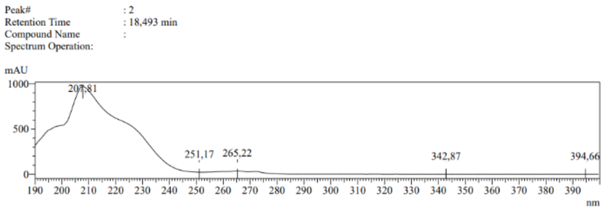

(*S*)-3-(Diphenylphosphaneyl)-*N*,*N*-dimethyl-3-(pyridin-4-yl)propanamide (3’g):

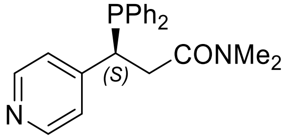

3’g

Compound **3’g** was synthesized following **general procedure 1** with (*E*)-**3g** (18 mg, 0.1 mmol, 1.0 equiv), Ph_2PH (17 µL, 0.1 mmol, 1.0 equiv), (S_C,R_P)-Mn(I) (3.0 mg, 0.004 mmol, 0.04 equiv), *t*PentOK (0.042 M, 190 µL, 0.008 mmol, 0.08 equiv), in dry, deoxygenated toluene (1 mL). Pentane (2 mL) was added and the resulting mixture was filtered through a plug of alumina in order to remove the catalyst. EtOAc (1 mL) was added to flush the plug. Product **3’g** was obtained as a white solid after evaporation of the solvent under reduced pressure. [(*E*)-**3g**, 99% yield, 88:12 er].

^{1}H NMR (600 MHz, $CDCl_3$): δ 8.41–8.28 (m, 2H), 7.73–7.61 (m, 2H), 7.48–7.37 (m, 3H), 7.19–7.08 (m, 7H), 4.28–4.19 (m, 1H), 3.01–2.91 (m, 1H), 2.83 (s, 3H), 2.79 (s, 3H), 2.61–2.59 (m, 1H) ppm.

^{13}C NMR (151 MHz, $CDCl_3$): δ 170.0 (d, J = 17.8 Hz), 150.6, 149.5, 135.4, 135.3 (d, J = 15.7 Hz), 133.7 (d, J = 20.6 Hz), 133.3 (d, J = 19.2 Hz), 129.6, 129.0, 128.8 (d, J = 7.5 Hz), 128.1 (d, J = 7.4 Hz), 124.2, 40.4 (d, J = 12.1 Hz), 37.0, 36.0 (d, J = 22.9 Hz), 35.5 ppm.

^{31}P NMR (243 MHz, $CDCl_3$): δ 0.1 ppm.

HRMS (ESI+, m/z) calc. for $C_{22}H_{24}N_2OP$: $[M+H]^+$ = 363.1626, found $[M+H]^+$ = 363.1625.

CSP-HPLC: (254 nm, Chiralcel AD-H, *n*-heptane:*i*PrOH = 80:20, 40 °C, 0.5 mL/min), t_R = 18.0 min (minor), t_R = 21.4 min (major).

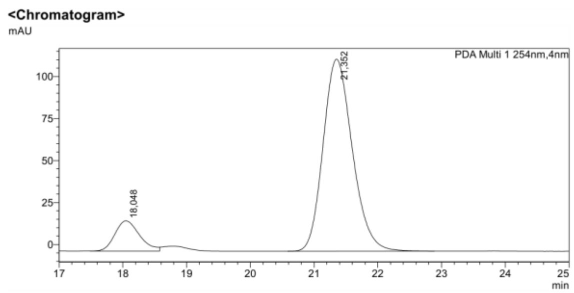

<Peak Table>
PDA Ch1 254nm

Peak#	Ret. Time	Area	Area%
1	18,048	485812	11,761
2	21,352	3644980	88,239
Total		4130792	100,000

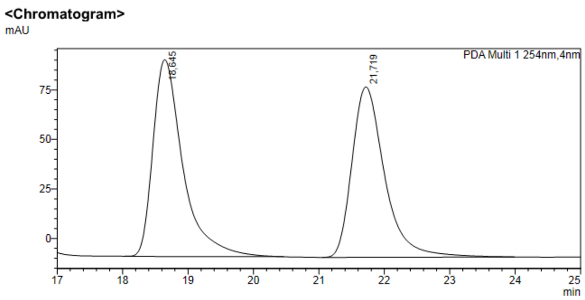

<Peak Table>
PDA Ch1 254nm

Peak#	Ret. Time	Area	Area%
1	18,645	3163794	51,465
2	21,719	2983625	48,535
Total		6147419	100,000

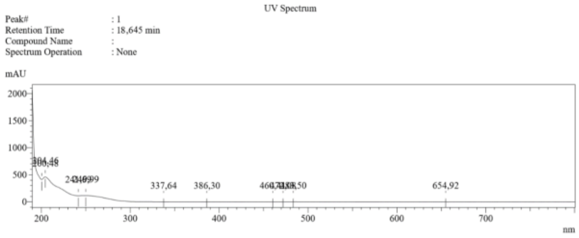

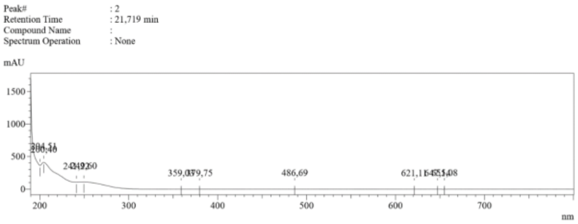

(*S*)-3-(Diphenylphosphaneyl)-*N*,*N*-dimethyl-3-(pyridin-3-yl)propanamide (3’h):

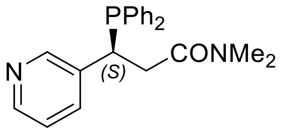

3’h

Compound **3’h** was synthesized following **general procedure 1** with (*E*)-**3h** (18 mg, 0.1 mmol, 1.0 equiv), Ph_2PH (17 µL, 0.1 mmol, 1.0 equiv), (S_C,R_P)-Mn(I) (3.0 mg, 0.004 mmol, 0.04 equiv), *t*PentOK (0.042 M, 190 µL, 0.008 mmol, 0.08 equiv), in dry, deoxygenated toluene (1 mL).

Pentane (2 mL) was added and the resulting mixture was filtered through a plug of alumina in order to remove the catalyst. EtOAc (1 mL) was added to flush the plug. Product **3'h** was obtained as a white solid after evaporation of the solvent under reduced pressure. [(*E*)-**3h**, 74% yield, 88:12 er].

^{1}H NMR (600 MHz, $CDCl_3$): δ 8.47–8.35 (m, 2H), 7.70–7.66 (m, 2H), 7.45–7.41 (m, 4H), 7.19–7.10 (m, 6H), 4.27–4.23 (m, 1H), 2.99–2.94 (m, 1H), 2.81 (s, 3H), 2.77 (s, 3H), 2.64–2.58 (m, 1H) ppm.

^{13}C NMR (151 MHz, $CDCl_3$): δ 170.1 (d, *J* = 15.0 Hz), 150.2, 147.6, 136.7, 135.6 (d, *J* = 7.6 Hz), 135.5 (d, *J* = 9.4 Hz), 133.7 (d, *J* = 20.4 Hz), 133.4 (d, *J* = 19.2 Hz), 129.6, 129.0, 128.8 (d, *J* = 7.4 Hz), 128.1 (d, *J* = 7.2 Hz), 125.3, 123.0, 38.3 (d, *J* = 10.1 Hz), 37.0, 36.3 (d, *J* = 23.4 Hz), 35.5 ppm.

^{31}P NMR (243 MHz, $CDCl_3$): δ -0.8 ppm.

HRMS (ESI+, m/z) calc. for $C_{22}H_{24}N_2OP$: $[M+H]^+$ = 363.1626, found $[M+H]^+$ = 363.1632.

CSP-HPLC: (254 nm, Chiralcel AD-H, *n*-heptane:*i*PrOH = 90:10, 40 °C, 0.5 mL/min), t_R = 14.0 min (major), t_R = 15.1 min (minor).

<Chromatogram>

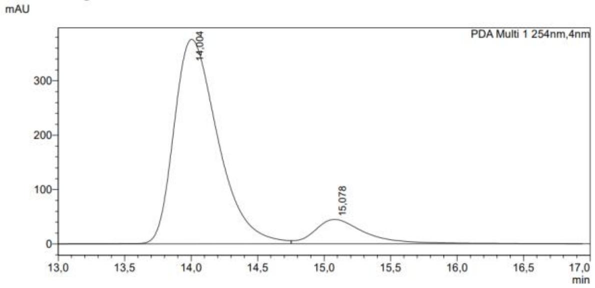

<Peak Table>

PDA Ch1 254nm

Peak#	Ret. Time	Area	Area%
1	14,004	8630400	87,812
2	15,078	1197828	12,188
Total		9828228	100,000

<Chromatogram>

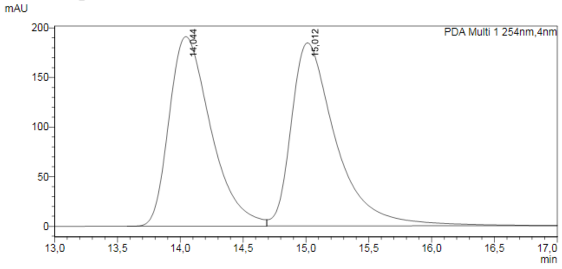

<Peak Table>

PDA Ch1 254nm

Peak#	Ret. Time	Area	Area%
1	14,044	4409987	49,143
2	15,012	4563866	50,857
Total		8973853	100,000

UV Spectrum

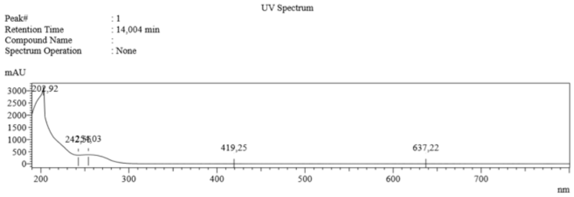

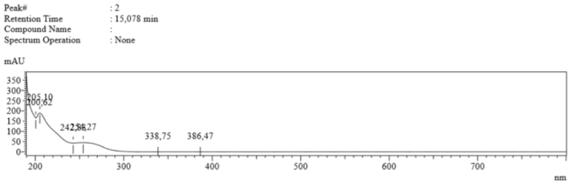

(*R*)-3-(Diphenylphosphaneyl)-*N*,*N*-dimethyl-3-(pyridin-2-yl)propanamide (3'i):

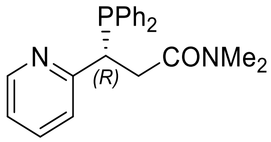

3'i

Compound **3'i** was synthesized following **general procedure 1** with (*E*)-**3i** (18 mg, 0.1 mmol, 1.0 equiv), Ph_2PH (17 µL, 0.1 mmol, 1.0 equiv), (R_C,S_P)-Mn(I) (3.0 mg, 0.004 mmol, 0.04 equiv), *t*PentOK (0.042 M, 190 µL, 0.008 mmol, 0.08 equiv), in dry, deoxygenated toluene (1 mL). Pentane (2 mL) was added and the resulting mixture was filtered through a plug of alumina in order to remove the catalyst. EtOAc (1 mL) was added to flush the plug. Product **3'i** was obtained as a white solid after evaporation of the solvent under reduced pressure. [(*E*)-**3i**, 99% yield, 94:6 er].

^{1}H NMR (600 MHz, $CDCl_3$): δ 8.45 (d, *J* = 4.3 Hz, 1H), 7.94–7.91 (m, 2H), 7.56–7.49 (m, 5H), 7.38–7.35 (m, 2H), 7.29–7.27 (m, 2H), 7.05–7.03 (m, 1H), 7.01–6.99 (m, 1H), 4.66–4.62 (m, 1H), 3.66–3.61 (m, 1H), 2.92 (s, 3H), 2.86–2.81 (m, 1H), 2.77 (s, 3H) ppm.

^{13}C NMR (151 MHz, $CDCl_3$): δ 170.2 (d, *J* = 14.2 Hz), 155.9 (d, *J* = 5.8 Hz), 149.1 (d, *J* = 1.6 Hz), 135.9 (d, *J* = 1.5 Hz), 132.0 (d, *J* = 2.5 Hz), 131.7 (d, *J* = 13.0 Hz), 131.6 (d, *J* = 2.6 Hz), 131.5 (d, *J* = 8.8 Hz), 131.3 (d, *J* = 9.1 Hz), 131.0 (d, *J* = 16.7 Hz), 128.8 (d, *J* = 11.5 Hz), 128.1 (d, *J* = 11.9 Hz), 125.0 (d, *J* = 4.1 Hz), 121.7 (d, *J* = 5.3 Hz), 45.2 (d, *J* = 16.2 Hz), 37.2, 35.6, 31.8 ppm.

^{31}P NMR (243 MHz, $CDCl_3$): δ -0.5 ppm.

HRMS (ESI+, m/z) calc. for $C_{22}H_{24}N_2OP$: $[M+H]^+$ = 363.1626, found $[M+H]^+$ = 363.1621.

CSP-HPLC: (254 nm, Chiralcel AD-H, *n*-heptane:*i*PrOH = 80:20, 40 °C, 0.5 mL/min), t_R = 13.7 min (minor), t_R = 21.4 min (major).

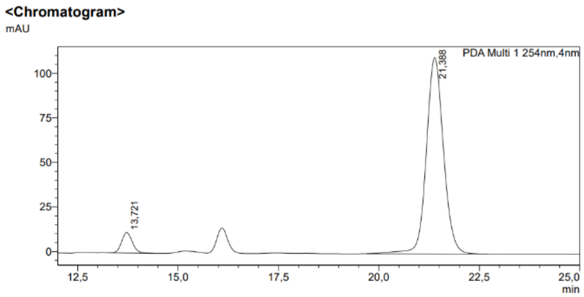

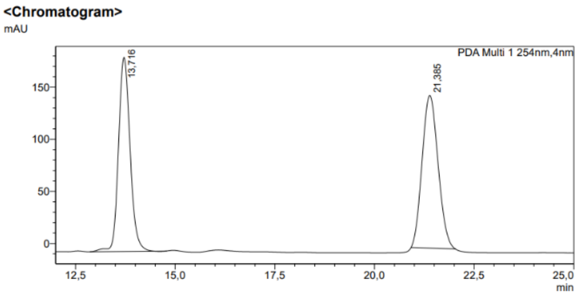

<Peak Table>
PDA Ch1 254nm

Peak#	Ret. Time	Area%
1	13,721	6,117
2	21,388	93,883
Total		100,000

<Peak Table>
PDA Ch1 254nm

Peak#	Ret. Time	Area%
1	13,716	47,930
2	21,385	52,070
Total		100,000

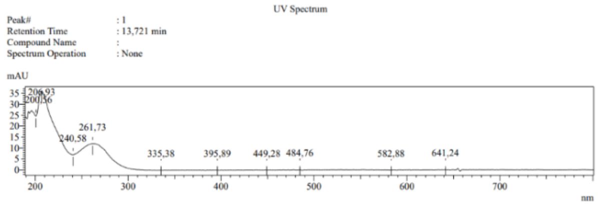

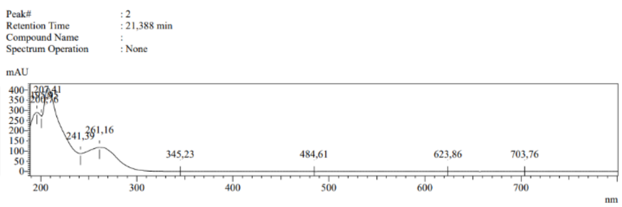

(*R*)-2-(2-(Diphenylphosphaneyl)-2-phenylethyl)benzo[*d*]oxazole (3’j):

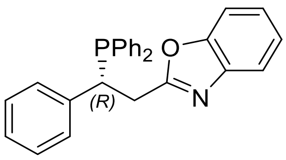

3’j

Compound **3’j** was synthesized following **general procedure 1** with (*E*)-**3j** or (*Z*)-**3j** (22 mg, 0.1 mmol, 1.0 equiv), Ph_2PH (17 µL, 0.1 mmol, 1.0 equiv), (R_C,S_P)-Mn(I) (1.5 mg, 0.002 mmol, 0.02 equiv), *t*PentOK (0.042 M, 95 µL, 0.004 mmol, 0.04 equiv), in dry, deoxygenated toluene (1 mL). Pentane (2 mL) was added and the resulting mixture was filtered through a plug of alumina in order to remove the catalyst. EtOAc (1 mL) was added to flush the plug. Product **3’j** was obtained as a white solid after evaporation of the solvent under reduced pressure. [(*E*)-**3j**, 99% yield, 87:13 er], [(*Z*)-**3j** 99% yield, 94.5:5.5 er].

^{1}H NMR (600 MHz, $CDCl_3$): δ 7.77–7.68 (m, 2H), 7.63–7.46 (m, 1H), 7.44–7.30 (m, 4H), 7.24–7.02 (m, 12H), 4.27–4.23 (m, 1H), 3.46–3.42 (m, 1H), 3.34–3.31 (m, 1H) ppm.

^{13}C NMR (151 MHz, $CDCl_3$): δ 165.0 (d, *J* = 15.6 Hz), 150.6, 141.2, 139.3 (d, *J* = 8.3 Hz), 136.0 (d, *J* = 14.0 Hz), 135.5 (d, *J* = 16.0 Hz), 134.1 (d, *J* = 21.0 Hz), 133.1 (d, *J* = 18.4 Hz), 129.6, 128.8 (d, *J* = 7.4 Hz), 128.7 (d, *J* = 7.5 Hz), 128.6, 128.3, 127.9 (d, *J* = 6.9 Hz), 126.6 (d, *J* = 2.4 Hz), 124.3, 123.9, 119.5, 110.2, 42.8 (d, *J* = 14.5 Hz), 32.9 (d, *J* = 24.5 Hz) ppm.

^{31}P NMR (243 MHz, $CDCl_3$): δ 0.7 ppm.

HRMS (ESI+, m/z) calc. for $C_{27}H_{23}NO_2P$ (oxidized product): $[M+H]^+$ = 424.1461, found $[M+H]^+$ = 424.1462.

CSP-HPLC: (254 nm, Chiralcel OD-H, *n*-heptane:*i*PrOH = 99:1, 40 °C, 0.5 mL/min), t_R = 17.6 min (minor), t_R = 18.6 min (major).

<Chromatogram>

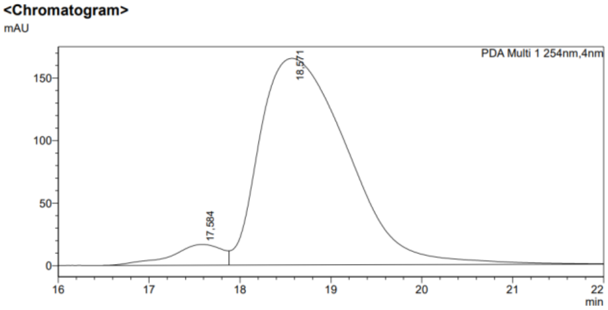

<Peak Table>

PDA Ch1 254nm

Peak#	Ret. Time	Area%
1	17,584	5,539
2	18,571	94,461
Total		100,000

<Chromatogram>

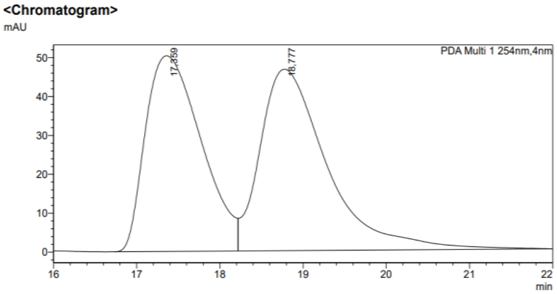

<Peak Table>

PDA Ch1 254nm

Peak#	Ret. Time	Area%
1	17,359	47,269
2	18,777	52,731
Total		100,000

UV Spectrum

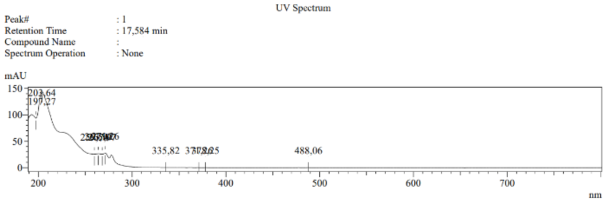

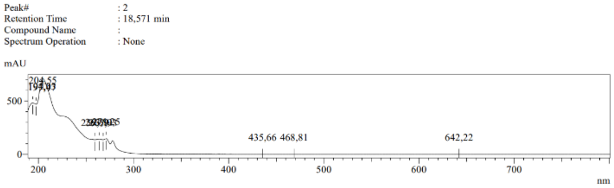

6. (*Z*)/(*E*)-Isomerization experiments

(Z)/(E)-isomerization was followed by NMR spectroscopy. For this purpose the reaction was performed in dry, deoxygenated toluene-d8 (1 mL) in the glovebox at RT in an oven-dried 4 mL vial equipped with a stirring bar. Aliquots were taken from the reaction mixture, filtered through a plug of alumina and collected in an oven-dried NMR tube. The samples were measured right after the preparation (about 10-15min).

Final conclusion of the following studies is that no (Z)/(E)-isomerization occurs under reaction conditions when all the reagents are present.

We looked at possible isomerization of substrate (*Z*)-**2b** in the course of the reaction. We explored whether (*Z*)-**2d** undergoes isomerization in the presence of one of the reaction components (Figure S3 and S3-1): *t*PentOK (2 mol%), *t*PentOK (2 mol%) and Ph_2PH (1.0 equiv) and in the presence of the (R_C,S_P)-Mn(I) (2 mol%) and *t*PentOK (4 mol%). Isomerization was not observed in either of these conditions, after 1 h. Lastly, we explored whether isomerization occurs under the manganese(I)-catalyzed hydrophosphination reaction conditions, namely, (R_C,S_P)-Mn(I) (2 mol%), *t*PentOK (4 mol%), Ph_2PH (1.0 equiv) and the substrate (*Z*)-**2d** (1.0 equiv). Also here, isomerization of (*Z*)-**2d** was not observed.

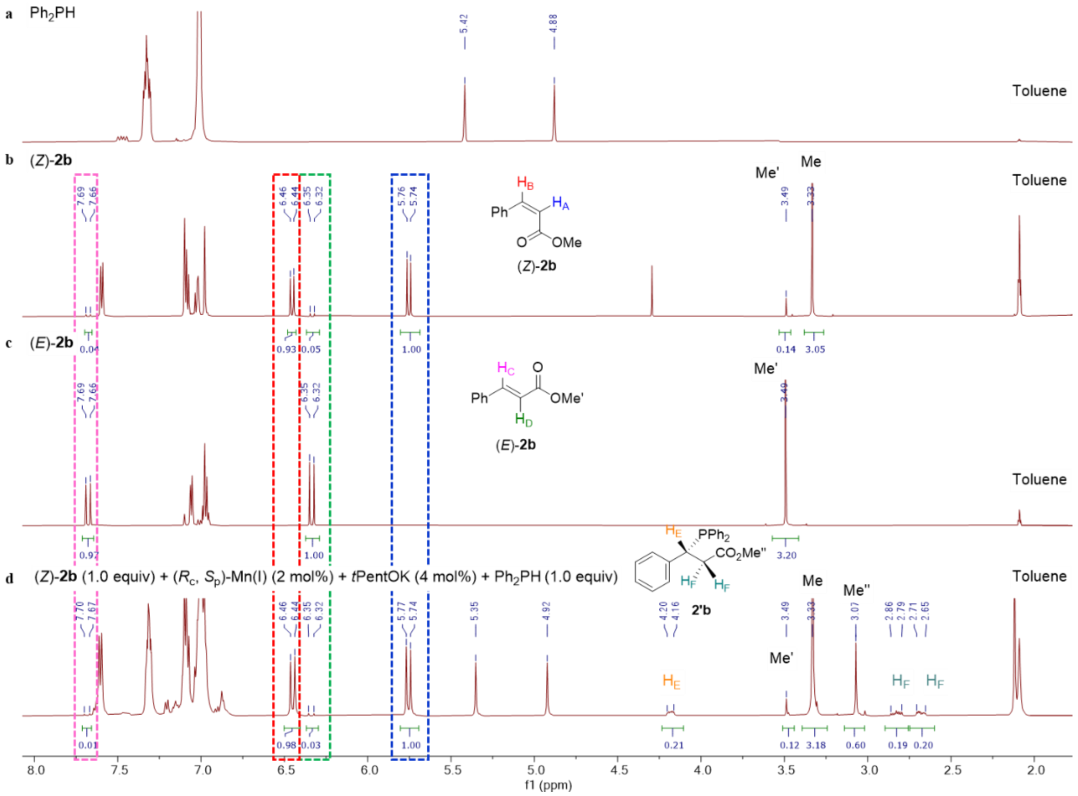

Figure S3. ¹H NMR studies on the isomerization of substrate (*Z*)-2b. ¹H NMR spectrum in dry, deoxygenated toluene-d8 (1 mL) of **a.** Ph_2PH. **b.** (*Z*)-**2b**.* **c.** (*Z*)-**2b**. **d.** a mixture of (*Z*)-**2b** (0.1 mmol, 1.0 equiv), (R_C,S_P)-Mn(I) (2

mol%), *t*PentOK (0.042 M, 4 mol%), Ph_2PH (0.1 mmol, 1.0 equiv) under nitrogen atmosphere at RT. ^1H NMR taken after 1 h and filtration through a plug of alumina with EtOAc (1 mL). *Compound (*Z*)-**2b** was used as a mixture ((*Z*)/(*E*) = 95:5) stereoisomers.

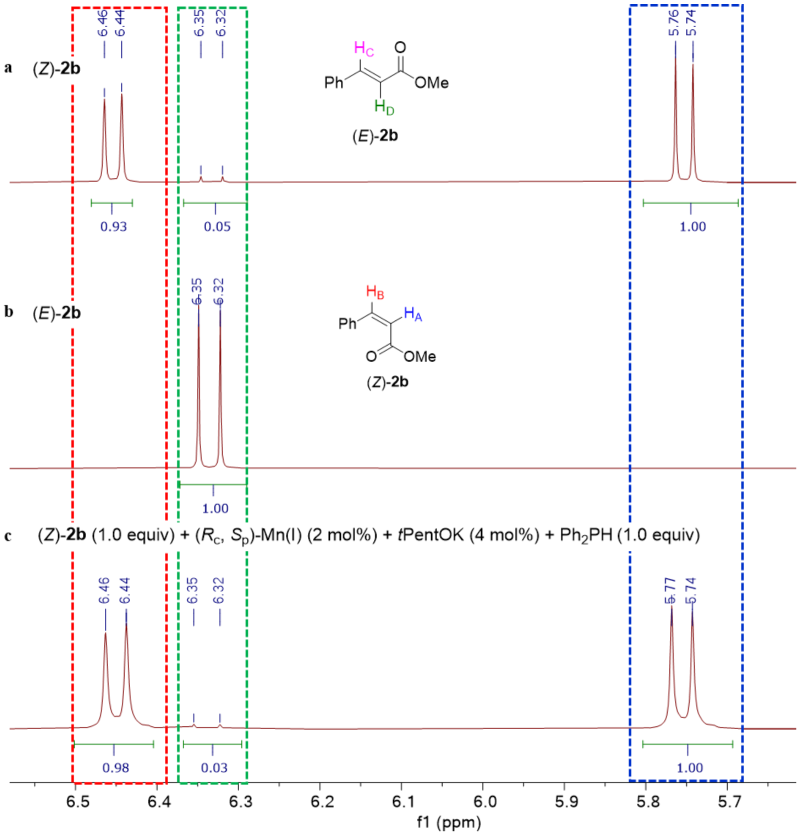

Figure S3-1. Zoomed in ^{1}H NMRs depicted in Figure S3. ^1H NMR spectrum in dry, deoxygenated toluene-d8 (1 mL) of **a.** (*Z*)-**2b**. **b.** (*Z*)-**2b**.* **c.** a mixture of (*Z*)-**2b** (0.1 mmol, 1.0 equiv), (R_C,S_P)-Mn(I) (2 mol%), *t*PentOK (0.042 M, 4 mol%), Ph_2PH (0.1 mmol, 1.0 equiv) under nitrogen atmosphere at RT. ^1H NMR taken after 1 h and filtration through a plug of alumina with EtOAc (1 mL). *Compound (*Z*)-**2b** was used as a mixture ((*Z*)/(*E*) = 95:5) stereoisomers.

7. Comparing the reactivities of *α,β*-unsaturated ketones, esters and amides in the base catalyzed and manganese(I)-catalyzed asymmetric hydrophosphination reactions

To rationalize the reactivity of ketones, esters and amides, the base and manganese(I)-catalyzed reactions were studied with ***1b****,* ***1d****,* ***2b*** *and* ***3b****. Specifically, the base catalyzed reactions were performed using substrates (E)-****1b****, (Z)-****1b****, (E)-****1d****, (E)-****2b****, (Z)-****2b****, (E)-****3b*** *and (Z)-****3b****, tPentOK (0.042 M, 3 mol%), in dry, deoxygenated toluene (1 mL) under nitrogen atmosphere at RT. The reaction mixture was quenched by HCl solution (1M), followed by addition of an aqueous solution of* H_2O_2 *(30%). The manganese(I)-catalyzed reactions were performed using substrates (E)-****1b****, (Z)-****1b****, (E)-****1d****, (E)-****2b****, (Z)-****2b****, (E)-****3b*** *and (Z)-****3b****,, the* (R_C,S_P)*-Mn(I) (2 mol%), tPentOK (0.042 M, 4 mol%), in dry, deoxygenated toluene (1 mL) under nitrogen atmosphere at RT. The reaction mixture was quenched by HCl solution (1M), followed by addition of an aqueous solution of* H_2O_2 *(30%). In both cases, the reaction conversion was analyzed by GS after 5 min. The reactions were carried out in duplo and the results presented an error of <5%. The results are presented in* ***Figure S4****.*

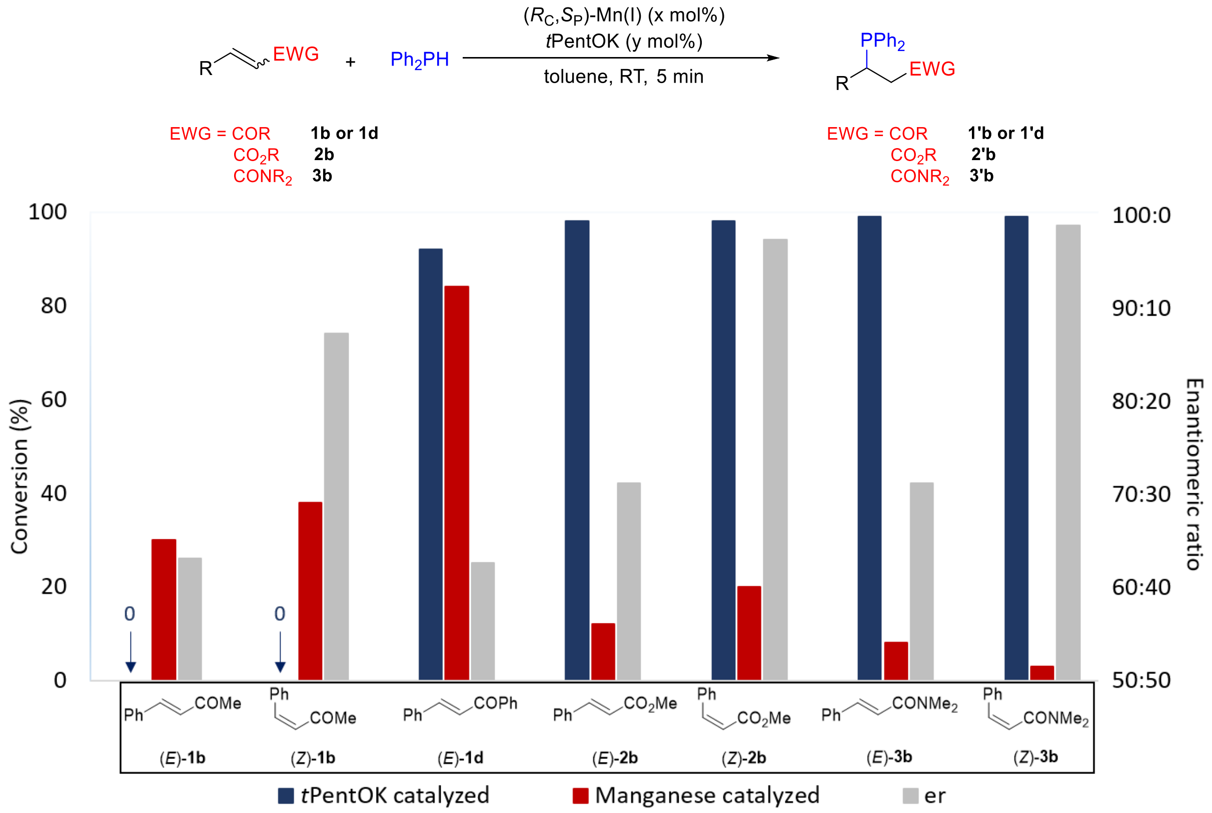

Figure S4. **Study of *α,β*-unsaturated ketones, esters and amides reactivity**. Reactivity of the substrates (*E*)-**1b**, (*Z*)-**1b**, (*E*)-**1d**, (*E*)-**2b**, (*Z*)-**2b**, (*E*)-**3b** and (*Z*)-**3b** for the base and Mn(I)-catalysed hydrophosphination**.** Blue represents the conversion of the base catalyzed reaction, red and grey the conversion and the enantiomeric ratios respectively of the products resulting from the Mn(I)-catalyzed rections.

We found out that the base catalyzed reactions are leading to full conversion, except for the ketones (*E*)-**1b** and (*Z*)-**1b** as a concomitant enolization is taking place; to overcome the enolization process, the aromatic substituted ketone (*E*)-**1d** was used and we could obtain full conversion in this case. The Mn(I)- catalyzed reactions showed a similar reactivity when the *E* and the *Z* substrates were considered within the same class of Michael acceptor. With these results we were not able to find a direct correlation between the speed of the base catalyzed reactions and the er values obtained for the Mn(I)-catalyzed reactions.

8. Synthesis of starting materials: general procedures and characterization of internal and terminal *α,β*-unsaturated nitriles, esters and amides

Method 1: To a 10 ml Schlenk tube equipped with a magnetic stirring bar, the manganese (6.0 mmol, 1.5 equiv), $Pd(OAc)_2$ (0.04 mmol) and the alkyne (4 mmol, 1 equiv) were added. Then water (6 mL) and CH_3CN (25 mL) were added and three cycles of vacuum and hydrogen filling were done. The mixture was stirred at RT for overnight. The solution was concentrated under reduced pressure and the corresponding product ((*Z*)-**1b**) was obtained after purification by column chromatography.[8]

Method 2: To an oven-dried Schlenk containing a suspension of the Lindlar catalyst (0.1 mmol, 0.05 equiv) in dry Et_2O (0.2 mL), ethyl but-2-ynoate (2.0 mmol, 1.0 equiv) was added. The Schlenk was equipped with a balloon containing hydrogen and three cycles of vacuum and hydrogen filling were done. Then, the reaction was stirred at RT overnight. The catalyst was removed by filtration of the reaction mixture through a Celite® pad. The solution was concentrated under reduced pressure and the corresponding product ((*Z*)-**2a**) was purified by flash column chromatography.[9]

Method 3: A solution of the corresponding aldehyde (5.0 mmol, 1.0 equiv) in anhydrous THF (17 mL) was treated with diethylcyanomethylphosphonate or ethyl 2-(diethoxyphosphoryl)acetate (5.5 mmol, 1.1 equiv) and *t*BuOK (5.5 mmol, 1.1 equiv) at 0 °C and stirred for 1 h under nitrogen atmosphere. The mixture was allowed to warm up to RT and stirred overnight. Subsequently, water (20 mL) was added and the mixture was extracted with CH_2Cl_2 (3 x 10 mL). The combined organic layers were dried over anhydrous $MgSO_4$, filtered and concentrated under reduced pressure. The corresponding products ((*E*)-**2c**, (*E*)-**2d**, (*E*)-**2e**, (*E*)-**2f**, (*E*)-**2g**, (*E*)-**2h**) were obtained after purification by column chromatography.[10]

Method 4: Compounds (*E*)-**2b**, (*E*)-**2c**, (*E*)-**2d**, (*E*)-**2e**, (*E*)-**2f**, (*E*)-**3b**, (*E*)-**3c** and (*E*)-**3j** were converted to their corresponding (*Z*)-isomers by photoisomerization in the presence of $BF_3{\cdot}OEt_2$. The corresponding (*E*)-isomer (0.5 mmol, 1.0 equiv) and $BF_3{\cdot}OEt_2$ (0.5 mmol, 1.0 equiv) were dissolved in CH_2Cl_2 (17 mL) under nitrogen atmosphere. The solution was irradiated at 365 nm for 24 h. The resulting solution was quenched with water (5 mL) and the organic layer was separated, dried over anhydrous $MgSO_4$, filtered and concentrated under reduced pressure. The (*Z*)-isomers ((*Z*)-**2b**, (*Z*)-**2c**, (*Z*)-**2d**, (*Z*)-**2e**, (*Z*)-**2f**, (*Z*)-**3b**, (*Z*)-**3c** and (*Z*)-**3j**) were obtained after purification by column chromatography.[11]

Method 5: A solution of crotonoyl chloride (10 mmol, 1.0 equiv) in dry Et_2O (6 mL) was cooled to 0 °C in an ice-bath. Then, anhydrous dimethylamine (2.0 M in THF, 20 mmol, 2.0 equiv) was added over a period of 5 min and the reaction mixture was allowed to warm up to RT and stirred overnight. The solvents were evaporated under reduced pressure and the amide ((*E*)-**3a**) was obtained after purification by column chromatography.[12,13]

Method 6: Thionyl chloride (6.0 mmol, 1.2 equiv) and dry DMF (0.2 mmol, 0.04 equiv) were added to a solution of the corresponding carboxylic acid (5.0 mmol, 1.0 equiv) in Et_2O (4 mL) at 0 °C. The solution was allowed to warm up to RT and stirred for 2 h before removal of solvent under reduced pressure. The resulting residue was redissolved in Et_2O (4 mL) and cooled to 0 °C. Then, dimethylamine (8.0 mmol, 1.6 equiv) and dry triethylamine (6.5 mmol, 1.3 equiv) were added. Stirring was continued at RT for an additional 3 h. Volatiles were removed under reduced pressure and the residue was redissolved in Et_2O (14 mL). The organic phase was washed with HCl (2.0 M, 2 x 2 mL), water (2 x 3 mL) and brine (4 mL), dried over anhydrous $MgSO_4$, filtered and concentrated under reduced pressure to obtain the corresponding amides ((*E*)-**3b**, (*E*)-**3c**, (*E*)-**3d**, (*E*)-**3e**, (*E*)-**3f**, (*E*)-**3g**, (*E*)-**3h** and (*E*)-**3i**) which were used after purification by column chromatography.[14,15,16]

Method 7: Compounds (*E*)-**3d**, (*E*)-**3e** and (*E*)-**3f** were converted to their corresponding (*Z*)-isomers by photoisomerization in the presence of thioxanthone. The corresponding (*E*)-isomer (0.8 mmol, 1.0 equiv) and thioxanthone (0.05 mmol, 0.06 equiv) were dissolved in CH_3CN (4 mL) under nitrogen atmosphere. The solution was irradiated at 450 nm for 24 h. The solvents were evaporated under reduced pressure and the (*Z*)-isomers ((*Z*)-**3d**, (*Z*)-**3e** and (*Z*)-**3f**) were obtained after purification by column chromatography.[17]

Method 8: In a 50 mL stainless steel pressure vessel, a mixture of 2-aminophenol (2.0 mmol, 1.0 equiv), carboxylic acid (2.0 mmol, 1.0 equiv), and $SnCl_2$ (2.0 mmol, 1.0 equiv) in dioxane (10 mL) was placed. The mixture was stirred at 180 °C for 30 h before removal of solvent under reduced pressure. The product ((*E*)-**3j**) was obtained after purification by column chromatography.[18]

9. Characterization data of starting materials

(*Z*)-4-Phenylbut-3-en-2-one ((*Z*)-1b):

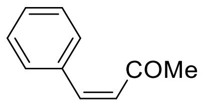

(*Z*)-**1b**

Compound (*Z*)-**1b** was prepared following **Method 1**. The product was obtained as a colorless oil after column chromatography (SiO_2, pentane:EtOAc 99:1). The NMR data are in agreement with literature precedents.[8]

^{1}H NMR (400 MHz, $CDCl_3$): 7.49–7.47 (m, 2H), 7.38–7.34 (m, 3H), 6.90 (d, J = 12.7 Hz, 1H), 6.18 (d, J = 12.7 Hz, 1H), 2.15 (s, 3H), ppm.

^{13}C NMR (101 MHz, $CDCl_3$): 201.0, 140.1, 135.4, 129.4, 129.1, 128.3, 126.1, 30.9 ppm.

Ethyl (*Z*)-but-2-enoate ((*Z*)-2a):

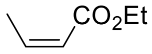

(*Z*)-**2a**

Compound (*Z*)-**2a** was prepared following **Method 2**. The product was obtained as a colorless oil after column chromatography (SiO_2, pentane:EtOAc 99:1). The NMR data are in agreement with literature precedents.[19]

^{1}H NMR (600 MHz, $CDCl_3$): δ 6.25 (dq, J = 11.4, 7.1 Hz, 1H), 5.72 (d, J = 11.4, Hz, 1H), 4.10 (q, J = 7.1 Hz, 2H), 2.07 (d, J = 7.1, Hz, 3H), 1.22 (t, J = 7.1 Hz, 3H) ppm.

^{13}C NMR (151 MHz, $CDCl_3$): δ 166.6, 144.9, 120.7, 59.7, 15.3, 14.3 ppm.

Methyl (*Z*)-3-phenylacrylate ((*Z*)-2b):

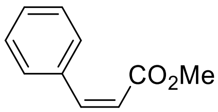

(*Z*)-**2b**

Compound (*Z*)-**2b** was prepared following **Method 4**. The product was obtained as a colorless oil after column chromatography (SiO_2, pentane:EtOAc 99:1). The NMR data are in agreement with literature precedents.[20]

^{1}H NMR (600 MHz, $CDCl_3$): δ 7.60–7.56 (m, 2H), 7.38–7.29 (m, 3H), 6.95 (d, *J* = 12.6 Hz, 1H), 5.95 (d, *J* = 12.6 Hz, 1H), 3.71 (s, 3H) ppm.

^{13}C NMR (151 MHz, $CDCl_3$): δ 166.6, 143.4, 134.7, 129.7, 129.1, 128.0, 119.3, 51.4 ppm.

Ethyl (*E*)-3-(4-methoxyphenyl)acrylate ((*E*)-2c):

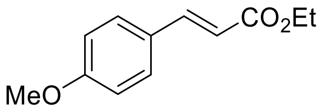

(*E*)-**2c**

Compound (*E*)-**2c** was prepared following **Method 3**. The product was obtained as a colorless oil after column chromatography (SiO_2, pentane:EtOAc 99:1). The NMR data are in agreement with literature precedents.[21]

^{1}H NMR (600 MHz, $CDCl_3$): δ 7.63 (d, *J* = 16.0 Hz, 1H), 7.47 (d, *J* = 8.8 Hz, 2H), 6.89 (d, *J* = 8.8 Hz, 2H), 6.30 (d, *J* = 16.0 Hz, 1H), 4.24 (q, *J* = 7.1 Hz, 2H), 3.83 (s, 3H), 1.32 (t, *J* = 7.1 Hz, 3H) ppm.

^{13}C NMR (151 MHz, $CDCl_3$): δ 167.3, 161.3, 144.2, 129.7, 127.2, 115.8, 114.3, 60.3, 55.4, 14.3 ppm.

Ethyl (*Z*)-3-(4-methoxyphenyl)acrylate ((*Z*)-2c):

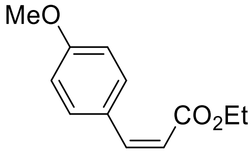

(*Z*)-**2c**

Compound (*Z*)-**2c** was prepared following **Method 4**. The product was obtained as a colorless oil after column chromatography (SiO_2, pentane:EtOAc 99:1). The NMR data are in agreement with literature precedents.[22]

^{1}H NMR (600 MHz, $CDCl_3$): δ 7.20–7.15 (m, 2H), 7.04 (d, *J* = 7.6 Hz, 1H), 6.83 (d, *J* = 12.6 Hz, 1H), 6.81–6.80 (m, 1H), 5.87 (d, *J* = 12.6 Hz, 1H), 4.10 (q, *J* = 7.1 Hz, 2H), 3.74 (s, 3H), 1.17 (t, *J* = 7.1 Hz, 3H) ppm.

^{13}C NMR (151 MHz, $CDCl_3$): δ 166.2, 159.2, 142.6, 136.1, 128.9, 122.3, 120.1, 114.9, 114.7, 60.3, 55.2, 14.1 ppm.

Ethyl (*E*)-3-(4-(trifluoromethyl)phenyl)acrylate ((*E*)-2d):

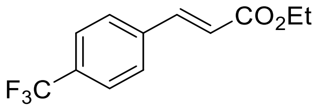

(*E*)-**2d**

Compound (*E*)-**2d** was prepared following **Method 3**. The product was obtained as a colorless oil after column chromatography (SiO_2, pentane:EtOAc 99:1). The NMR data are in agreement with literature precedents.[21]

^{1}H NMR (600 MHz, $CDCl_3$): δ 7.68 (d, *J* = 16.0 Hz, 1H), 7.65–7.60 (m, 4H), 6.50 (d, *J* = 16.0 Hz, 1H), 4.28 (q, *J* = 7.1 Hz, 2H), 1.34 (t, *J* = 7.1 Hz, 3H) ppm.

^{13}C NMR (151 MHz, $CDCl_3$): δ 166.4, 142.7, 137.8, 130.4 (q, *J* = 32.9 Hz), 128.1, 125.8 (q, *J* = 3.9 Hz), 123.9, 120.9, 60.8, 14.3 ppm.

Ethyl (*Z*)-3-(4-(trifluoromethyl)phenyl)acrylate ((*Z*)-2d):

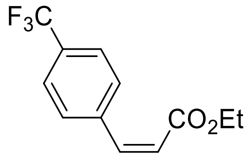

(*Z*)-**2d**

Compound (*Z*)-**2d** was prepared following **Method 4**. The product was obtained as a colorless oil after column chromatography (SiO_2, pentane:EtOAc 99:1). The NMR data are in agreement with literature precedents.[22]

^{1}H NMR (600 MHz, CDCl3): δ 7.65–7.59 (m, 4H), 6.98 (d, *J* = 12.6 Hz, 1H), 6.06 (d, *J* = 12.6 Hz, 1H), 4.17 (q, *J* = 7.1 Hz, 2H), 1.24 (t, *J* = 7.1 Hz, 3H) ppm.

^{13}C NMR (151 MHz, $CDCl_3$): δ 166.6, 141.4, 138.4, 130.5 (q, *J* = 32.5 Hz), 129.6, 124.9 (q, *J* = 3.8 Hz), 123.1, 122.1, 60.5, 14.0 ppm.

Ethyl (*E*)-3-(4-chlorophenyl)acrylate ((*E*)-2e):

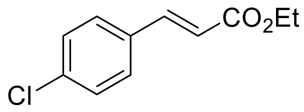

(*E*)-**2e**

Compound (*E*)-**2e** was prepared following **Method 3**. The product was obtained as a colorless oil after column chromatography (SiO_2, pentane:EtOAc 99:1). The NMR data are in agreement with literature precedents.[21]

^{1}H NMR (600 MHz, $CDCl_3$): δ 7.63 (d, *J* = 16.0 Hz, 1H), 7.45 (d, *J* = 8.6 Hz, 2H), 7.36 (d, *J* = 8.6 Hz, 2H), 6.41 (d, *J* = 16.0 Hz, 1H), 4.26 (q, *J* = 7.1 Hz, 2H), 1.33 (t, *J* = 7.1 Hz, 3H) ppm.

^{13}C NMR (151 MHz, $CDCl_3$): δ 166.7, 143.1, 136.1, 132.9, 129.2 (2 x C), 118.8, 60.6, 14.3 ppm.

Ethyl (*Z*)-3-(4-chlorophenyl)acrylate ((*Z*)-2e):

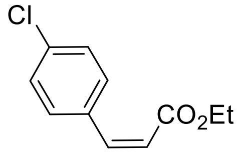

(*Z*)-**2e**

Compound (*Z*)-**2e** was prepared following **Method 4**. The product was obtained as a colorless oil after column chromatography (SiO_2, pentane:EtOAc 99:1). The NMR data are in agreement with literature precedents.[22]

^{1}H NMR (600 MHz, $CDCl_3$): δ 7.55 (d, *J* = 8.5 Hz, 2H), 7.32 (d, *J* = 8.5 Hz, 2H), 6.88 (d, *J* = 12.6 Hz, 1H), 5.96 (d, *J* = 12.6 Hz, 1H), 4.18 (q, *J* = 7.1 Hz, 2H),1.26 (t, *J* = 7.1 Hz, 3H) ppm.

^{13}C NMR (151 MHz, $CDCl_3$): δ 165.9, 141.8, 134.9, 133.2, 131.2, 128.2, 120.4, 60.4, 14.1 ppm.

Ethyl (*E*)-3-(3-chlorophenyl)acrylate ((*E*)-2f):

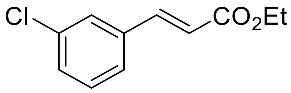

(*E*)-**2f**

Compound (*E*)-**2f** was prepared following **Method 3**. The product was obtained as a colorless oil after column chromatography (SiO_2, pentane:EtOAc 99:1). The NMR data are in agreement with literature precedents.[21]

^{1}H NMR (600 MHz, $CDCl_3$): δ 7.59 (d, *J* = 16.0 Hz, 1H), 7.48 (s, 1H), 7.39–7.25 (m, 3H), 6.41 (d, *J* = 16.0 Hz, 1H), 4.25 (q, *J* = 7.1 Hz, 2H), 1.32 (t, *J* = 7.1 Hz, 3H), ppm.

^{13}C NMR (151 MHz, $CDCl_3$): δ 166.5, 142.9, 136.2, 134.8, 130.1, 130.0, 127.7, 126.1, 119.7, 60.6, 14.2 ppm.

Ethyl (*Z*)-3-(3-chlorophenyl)acrylate ((*Z*)-2f):

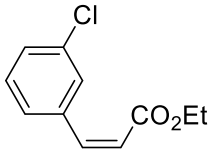

(*Z*)-**2f**

Compound (*Z*)-**2f** was prepared following **Method 4**. The product was obtained as a colorless oil after column chromatography (SiO_2, pentane:EtOAc 99:1). The NMR data are in agreement with literature precedents.[22]

^{1}H NMR (600 MHz, $CDCl_3$): δ 7.56–7.54 (m, 1H), 7.42–7.38 (m, 1H), 7.28–7.24 (m, 2H), 6.86 (d, *J* = 12.6 Hz, 1H), 5.97 (d, *J* = 12.6 Hz, 1H), 4.16 (q, *J* = 7.1 Hz, 2H), 1.23 (t, *J* = 7.1 Hz, 3H) ppm.

^{13}C NMR (151 MHz, $CDCl_3$): δ 165.8, 141.2, 136.6, 133.8, 129.5, 129.2, 128.8, 127.7, 122.3, 60.5, 14.0 ppm.

Ethyl (*E*)-3-(quinolin-2-yl)acrylate ((*E*)-2g):

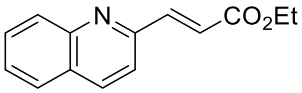

(*E*)-**2g**

Compound (*E*)-**2g** was prepared following **Method 3**. The product was obtained as a brown oil after column chromatography (SiO_2, pentane:EtOAc 9:1). The NMR data are in agreement with literature precedents.[23]

^{1}H NMR (600 MHz, $CDCl_3$): δ 8.15 (d, *J* = 8.5 Hz, 1H), 8.09 (d, *J* = 8.3 Hz, 1H), 7.88 (d, *J* = 16.0 Hz, 1H), 7.79 (d, *J* = 8.1, 1H), 7.74–7.69 (m, 1H), 7.59 (d, *J* = 8.5 Hz, 1H), 7.56–7.51 (m, 1H), 6.97 (d, *J* = 16.0 Hz, 1H), 4.29 (q, *J* = 7.1 Hz, 2H), 1.34 (t, *J* = 7.1 Hz, 3H) ppm.

^{13}C NMR (151 MHz, $CDCl_3$): δ 166.6, 153.3, 148.3, 148.1, 144.1, 136.8, 130.0, 129.9, 128.1, 127.3, 123.8, 120.2, 60.8, 14.3 ppm.

Ethyl (*E*)-3-(pyridin-2-yl)acrylate ((*E*)-2h):

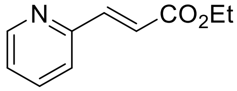

(*E*)-**2h**

Compound (*E*)-**2h** was prepared following **Method 3**. The product was obtained as a colorless oil after column chromatography (SiO_2, pentane:EtOAc 99:1). The NMR data are in agreement with literature precedents.[20]

^{1}H NMR (600 MHz, $CDCl_3$): δ 8.64 (d, *J* = 4.8 Hz, 1H), 7.73–7.69 (m, 1H), 7.68 (d, *J* = 15.7 Hz, 1H), 7.43 (d, *J* = 7.8 Hz, 1H), 7.28–7.25 (m, 1H), 6.91 (d, *J* = 15.7 Hz, 1H), 4.27 (q, *J* = 7.1 Hz, 2H), 1.33 (t, *J* = 7.1 Hz, 3H) ppm.

^{13}C NMR (151 MHz, $CDCl_3$): δ 166.7, 153.0, 150.1, 143.2, 136.7, 124.2, 124.1, 122.5, 60.6, 14.3 ppm.

(*E*)-*N*,*N*-Dimethylbut-2-enamide ((*E*)-3a):

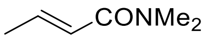

(*E*)-**3a**

Compound (*E*)-**3a** was prepared following **Method 5**. The product was obtained as a colorless oil after column chromatography (SiO_2, pentane: EtOAc 98:2). The NMR data are in agreement with literature precedents.[24]

^{1}H NMR (600 MHz, $CDCl_3$): δ 6.87 (dq, *J* = 15.0, 6.9 Hz, 1H), 6.27 (dq, *J* = 15.0, 1.7 Hz, 1H), 3.06 (s, 3H), 2.99 (s, 3H), 1.87 (dd, *J* = 6.9, 1.7 Hz, 3H) ppm.

^{13}C NMR (151 MHz, $CDCl_3$): δ 166.8, 141.1, 121.8, 37.3, 35.6, 18.0 ppm.

(*E*)-*N*,*N*-Dimethylcinnamamide ((*E*)-3b):

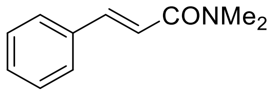

(*E*)-**3b**

Compound (*E*)-**3b** was prepared following **Method 6**. The product was obtained as a white solid and used after purification with column chromatography Et_2O:EtOAc (2:1). The NMR data are in agreement with literature precedents.[25]

^{1}H NMR (600 MHz, $CDCl_3$): δ 7.67 (d, *J* = 15.4 Hz, 1H), 7.54–7.52 (m, 2H), 7.39–7.32 (m, 3H), 6.89 (d, *J* = 15.4 Hz, 1H), 3.17 (s, 3H), 3.07 (s, 3H) ppm.

^{13}C NMR (151 MHz, $CDCl_3$): δ 166.6, 142.2, 135.3, 129.4, 128.7, 127.7, 117.4, 37.3, 35.8 ppm.

(*Z*)-*N*,*N*-Dimethyl-3-phenylacrylamide ((*Z*)-3b):

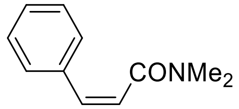

(*Z*)-**3b**

Compound (*Z*)-**3b** was prepared following **Method 4**. The product was obtained as a colorless oil after column chromatography (SiO_2, pentane:EtOAc 99:1). The NMR data are in agreement with literature precedents.[26]

^{1}H NMR (600 MHz, $CDCl_3$): δ 7.41-7.38 (m, 3H), 7.31-7.28 (m, 2H), 7.18 (d, *J* = 12.3 Hz, 1H), 6.33 (d, *J* = 12.3 Hz, 1H), 3.14 (s, 3H), 2.76 (s, 3H) ppm.

^{13}C NMR (151 MHz, $CDCl_3$): δ 171.9, 141.2, 134.6, 130.0, 129.3, 127.6, 116.5, 39.4, 37.6 ppm.

(*E*)-3-(4-Methoxyphenyl)-*N,N*-dimethylacrylamide ((*E*)-3c):

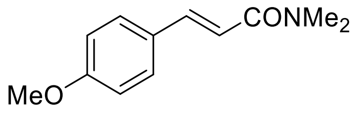

(*E*)-**3c**

Compound (*E*)-**3c** was prepared following **Method 6**. The product was obtained as a white solid and used without further purification. The NMR data are in agreement with literature precedents.[25]

^{1}H NMR (600 MHz, $CDCl_3$): δ 7.63 (d, *J* = 15.4 Hz, 1H), 7.48 (d, *J* = 8.7 Hz, 2H), 6.89 (d, *J* = 8.7 Hz, 2H), 6.76 (d, *J* = 15.4 Hz, 1H), 3.83 (s, 3H), 3.16 (s, 3H), 3.06 (s, 3H) ppm.

^{13}C NMR (151 MHz, $CDCl_3$): δ 167.0, 160.8, 142.0, 129.3, 128.1, 114.9, 114.2, 55.3, 37.3, 35.9 ppm.

(*Z*)-3-(4-Methoxyphenyl)-*N,N*-dimethylacrylamide ((*Z*)-3c):

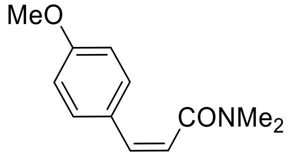

(*Z*)-**3c**

Compound (*Z*)-**3c** was prepared following **Method 4.** The product was obtained as a colorless oil after column chromatography (SiO_2, pentane:EtOAc 99:1). The NMR data are in agreement with literature precedents.[27]

^{1}H NMR (600 MHz, $CDCl_3$): δ 7.26 (d, *J* = 8.8 Hz, 2H), 6.80 (d, *J* = 8.8 Hz, 2H), 6.53 (d, *J* = 12.5 Hz, 1H), 5.88 (d, *J* = 12.5 Hz, 1H), 3.76 (s, 3H), 2.95 (s, 3H), 2.82 (s, 3H) ppm.

^{13}C NMR (151 MHz, $CDCl_3$): δ 169.2, 159.6, 132.9, 129.6, 128.2, 121.1, 113.8, 55.2, 37.4, 34.3 ppm.

(*E*)-3-(4-Bromophenyl)-*N*,*N*-dimethylacrylamide ((*E*)-3d):

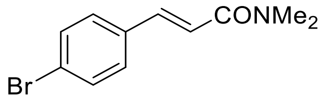

(*E*)-**3d**

Compound (*E*)-**3d** was prepared following **Method 6**. The product was obtained as a white solid and used after purification with column chromatography Et_2O:EtOAc (2:1). The NMR data are in agreement with literature precedents.[28]

^{1}H NMR (400 MHz, $CDCl_3$): δ 7.60 (d, *J* = 15.5 Hz, 1H), 7.50 (d, *J* = 8.5 Hz, 2H), 7.38 (d, *J* = 8.5 Hz, 2H), 6.87 (d, *J* = 15.5 Hz, 1H), 3.17 (s, 3H), 3.07 (s, 3H) ppm.

^{13}C NMR (101 MHz, $CDCl_3$): δ 166.4, 141.1, 134.3, 132.0, 129.2, 118.1, 37.2, 36.0 ppm.

(*Z*)-3-(4-Bromophenyl)-*N*,*N*-dimethylacrylamid ((*Z*)-3d):

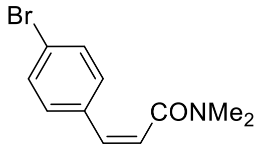

(*Z*)-**3d**

Compound (*Z*)-**3d** was prepared following **Method 7**. The product was obtained as a colourless oil and used after purification with column chromatography Et_2O:EtOAc (9:1). The NMR data are in agreement with literature precedents.[29]

^{1}H NMR (400 MHz, $CDCl_3$): δ 7.42 (d, *J* = 8.5 Hz, 2H), 7.21 (d, *J* = 8.5 Hz, 2H), 6.55 (d, *J* = 12.6 Hz, 1H), 6.06 (d, *J* = 12.6 Hz, 1H), 2.96 (s, 3H), 2.83 (s, 3H) ppm.

^{13}C NMR (101 MHz, $CDCl_3$): δ 169.0, 135.6, 133.3, 128.6, 128.4, 128.1, 123.4, 37.5, 34.4 ppm.

(*E*)-3-(3-Bromophenyl)-*N*,*N*-dimethylacrylamide ((*E*)-3e)

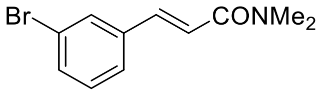

(*E*)-**3e**

Compound (*E*)-**3e** was prepared following **Method 6**. The product was obtained as a white solid and used after purification with column chromatography Et_2O:EtOAc (2:1). The NMR data are in agreement with literature precedents.[28]

^{1}H NMR (400 MHz, $CDCl_3$): δ 7.71 (s, 1H), 7.62 (d, *J* = 15.5 Hz, 1H), 7.52–7.45 (m, 2H), 7.30–7.22 (m, 1H), 6.92 (d, *J* = 15.5 Hz, 1H), 3.21 (s, 3H), 3.11 (s, 3H) ppm.

^{13}C NMR (101 MHz, $CDCl_3$): δ 166.2, 140.7, 137.5, 132.3, 130.3, 130.1, 126.8, 122.9, 118.9, 37.5, 36.0 ppm.

(*Z*)-3-(3-Bromophenyl)-*N*,*N*-dimethylacrylamide ((*Z*)-3e)

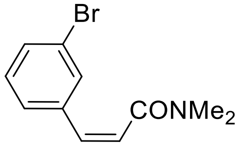

(*Z*)-**3e**

Compound (*Z*)-**3e** was prepared following **Method 7**. The product was obtained as a colourless oil and used after purification with column chromatography Et_2O:EtOAc (9:1). The NMR data are in agreement with literature precedents.[29]

^{1}H NMR (400 MHz, $CDCl_3$): δ 7.46 (s, 1H), 7.39 (d, *J* = 7.9 Hz, 1H), 7.28 (t, *J* = 7.4 Hz, 1H), 7.17 (t, *J* = 7.9 Hz, 1H), 6.55 (d, *J* = 12.6 Hz, 1H), 6.08 (d, *J* = 12.6 Hz, 1H), 2.97 (s, 3H), 2.83 (s, 3H) ppm.

^{13}C NMR (101 MHz, $CDCl_3$): δ 168.3, 137.6, 131.7, 131.3, 131.2, 130.1, 126.6, 124.9, 122.6, 37.5, 34.4 ppm.

***(E)*-3-(2-Bromophenyl)-*N,N*-dimethylacrylamide ((*E*)-3f)**:

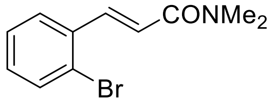

(*E*)-**3f**

Compound (*E*)-**3f** was prepared following **Method 6**. The product was obtained as a white solid and used after purification with column chromatography Et_2O:EtOAc (2:1). The NMR data are in agreement with literature precedents.[28]

^{1}H NMR (400 MHz, $CDCl_3$): δ 7.94 (d, *J* = 15.4 Hz, 1H), 7.60–7.55 (m, 2H), 7.31–7.28 (m, 1H), 7.20–7.15 (m, 1H), 6.80 (d, *J* = 15.4 Hz, 1H), 3.16 (s, 3H), 3.06 (s, 3H) ppm.

^{13}C NMR (101 MHz, $CDCl_3$): δ 166.2, 140.7, 135.7, 133.3, 130.4, 127.7, 127.5, 124.9, 120.8, 37.5, 35.9 ppm.

(*Z*)-3-(2-Bromophenyl)-*N,N*-dimethylacrylamide ((*Z*)-3f):

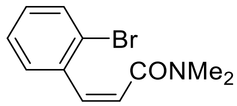

(*Z*)-**3f**

Compound (*E*)-**3f** was prepared following **Method 7**. The product was obtained as a colourless oil and used after purification with column chromatography Et_2O:EtOAc (9:1). The NMR data are in agreement with literature precedents.[29]

^{1}H NMR (400 MHz, $CDCl_3$): δ 7.56 (d, *J* = 8.0 Hz, 1H), 7.47–7.37 (m, 1H), 7.30–7.20 (m, 1H), 7.16–7.12 (m, 1H), 6.86 (d, *J* = 12.3 Hz, 1H), 6.15 (d, *J* = 12.3 Hz, 1H), 2.86 (s, 3H), 2.71 (s, 3H) ppm.

^{13}C NMR (101 MHz, $CDCl_3$): δ 168.1, 136.0, 132.9, 132.5, 129.8, 129.7, 127.5, 125.1, 123.3, 37.4, 34.4 ppm.

(*E*)-*N,N*-Dimethyl-3-(pyridin-4-yl)acrylamide ((*E*)-3g):

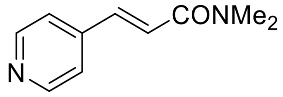

(*E*)-**3g**

Compound (*E*)-**3g** was prepared following **Method 6**. The product was obtained as a pale pink solid and used after purification with column chromatography Et_2O:EtOAc (9:1). The NMR data are in agreement with literature precedents.[25]

^{1}H NMR (600 MHz, $CDCl_3$): δ 8.61 (d, *J* = 5.9 Hz, 2H), 7.56 (d, *J* = 15.5 Hz, 1H), 7.36 (d, *J* = 5.9 Hz, 2H), 7.05 (d, *J* = 15.5 Hz, 1H), 3.18 (s, 3H), 3.07 (s, 3H), ppm.

^{13}C NMR (151 MHz, $CDCl_3$): δ 165.6, 150.4, 142.6, 139.4, 122.0, 121.7, 37.4, 36.0ppm.

(*E*)-*N,N*-Dimethyl-3-(pyridin-3-yl)acrylamide ((*E*)-3h):

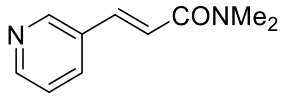

(*E*)-**3h**

Compound (*E*)-**3h** was prepared following **Method 6**. The product was obtained as a white solid and used after purification with column chromatography Et_2O:EtOAc (9:1). The NMR data are in agreement with literature precedents.[25]

^{1}H NMR (600 MHz, $CDCl_3$): δ 8.74 (s, 1H), 8.51 (d, *J* = 4.7 Hz, 1H), 7.76 (d, *J* = 7.9 Hz, 1H), 7.59 (d, *J* = 15.5 Hz, 1H), 7.25 (dd, *J* = 7.9, 4.7 Hz, 1H), 6.92 (d, *J* = 15.5 Hz, 1H), 3.13 (s, 3H), 3.02 (s, 3H) ppm.

^{13}C NMR (151 MHz, $CDCl_3$): δ 165.9, 150.2, 149.2, 138.6, 134.2, 131.0, 123.6, 119.5, 37.4, 35.9 ppm.

(*E*)-*N*,*N*-Dimethyl-3-(pyridin-2-yl)acrylamide ((*E*)-3i):

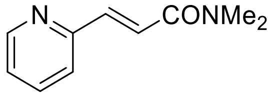

(*E*)-**3i**

Compound (*E*)-**3i** was prepared following **Method 6**. The product was obtained as a yellow solid and used after purification with column chromatography Et_2O:EtOAc (9:1). The NMR data are in agreement with literature precedents.[25]

^{1}H NMR (400 MHz, $CDCl_3$): δ 8.60 (d, *J* = 4.5 Hz, 1H), 7.56–7.46 (m, 1H), 7.48 (d, *J* = 15.0 Hz, 1H), 7.37 (d, *J* = 15.0 Hz, 1H), 7.21 (d, *J* = 7.8 Hz, 1H), 7.10–7.06 (m, 1H), 3.04 (s, 3H), 2.91 (s, 3H) ppm.

^{13}C NMR (151 MHz, $CDCl_3$): δ 166.4, 155.5, 149.9, 140.7, 136.8, 124.8, 123.8, 121.5, 37.5, 35.9 ppm.

(*E*)-2-Styrylbenzo[*d*]oxazole ((*E*)-3j):

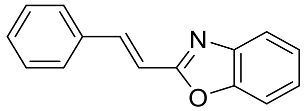

(*E*)-**3j**

Compound (*E*)-**3j** was prepared following **Method 8**. The product was obtained as a white solid after column chromatography (SiO_2, pentane:EtOAc 10:1). The NMR data are in agreement with literature precedents.[18]

^{1}H NMR (400 MHz, $CDCl_3$): δ 7.78 (d, *J* = 16.6 Hz, 1H), 7.74–7.69 (m, 1H), 7.60–7.58 (m, 2H), 7.54–7.50 (m, 1H), 7.44–7.30 (m, 5H), 7.07 (d, *J* =16.6 Hz, 1H) ppm.

^{13}C NMR (101 MHz, $CDCl_3$): δ 162.8, 150.4, 142.2, 139.5, 135.1, 129.8, 129.0, 127.6, 125.2, 124.5, 119.9, 113.9, 110.3 ppm.

(*Z*)-2-Styrylbenzo[*d*]oxazole ((*Z*)-3j):

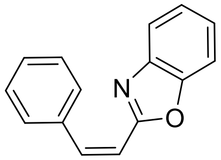

(*Z*)-**3j**

Compound (*Z*)-**3j** was prepared following **Method 4**. The product was obtained as a colorless oil after column chromatography (SiO_2, pentane:EtOAc 99:1). The NMR data are in agreement with literature precedents, where the (*E*)-isomer is reported, except for the coupling constants.[30]

^{1}H NMR (600 MHz, $CDCl_3$): δ 7.76–7.68 (m, 3H), 7.48–7.37 (m, 4H), 7.37–7.29 (m, 2H), 7.15–7.08 (m, 1H), 6.51 (d, *J* = 12.3 Hz, 1H) ppm.

^{13}C NMR (151 MHz, $CDCl_3$): δ 150.6, 139.9, 135.3, 130.0, 129.0, 128.9, 128.3, 128.1, 125.3, 124.5, 120.1, 114.7, 110.5 ppm.

10.NMR spectra

1’a

^{1}H NMR (400 MHz, $CDCl_3$)

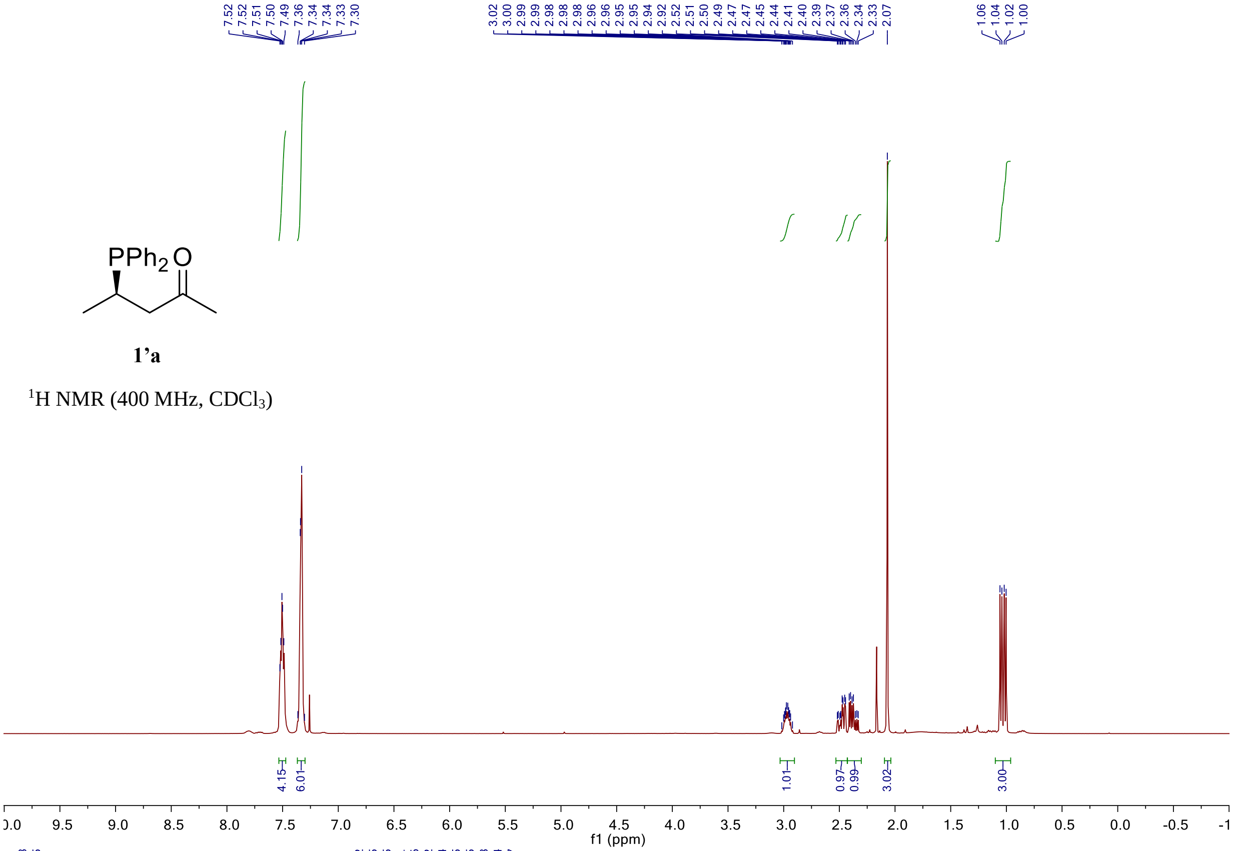

1’a

^{13}C NMR (151 MHz, $CDCl_3$)

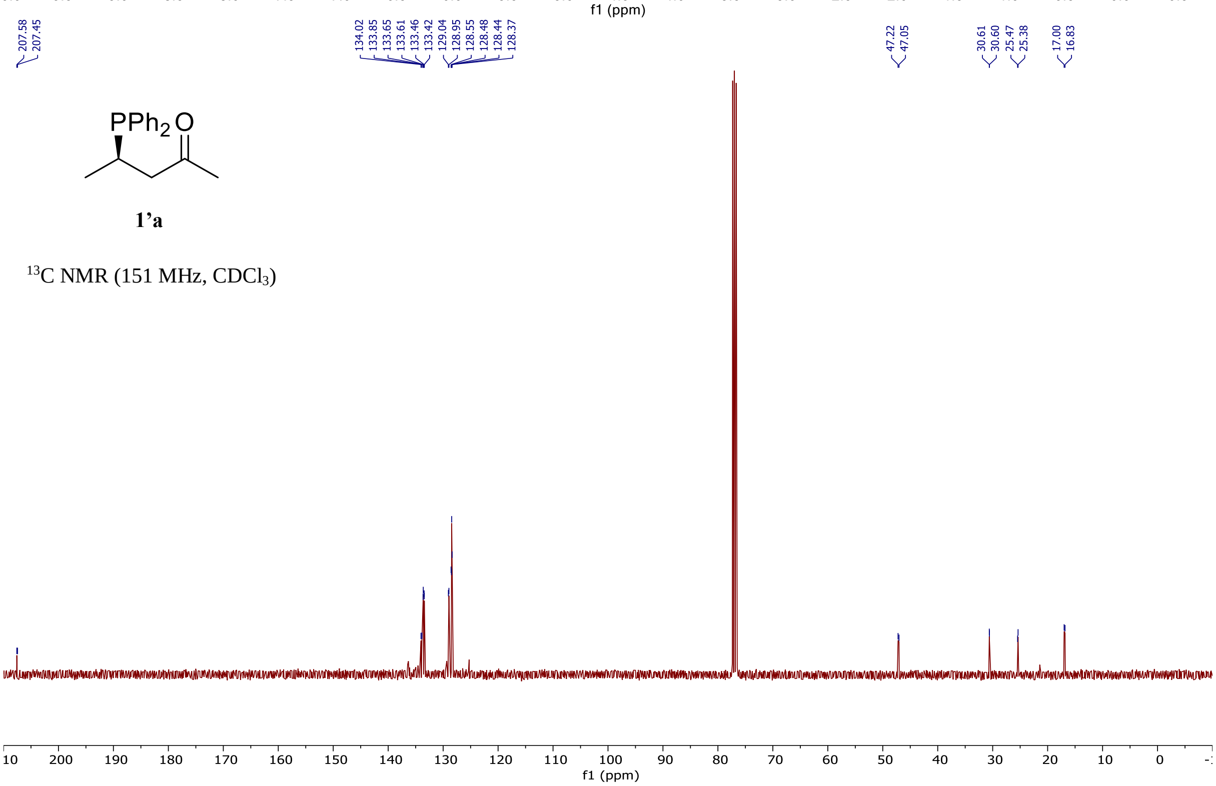

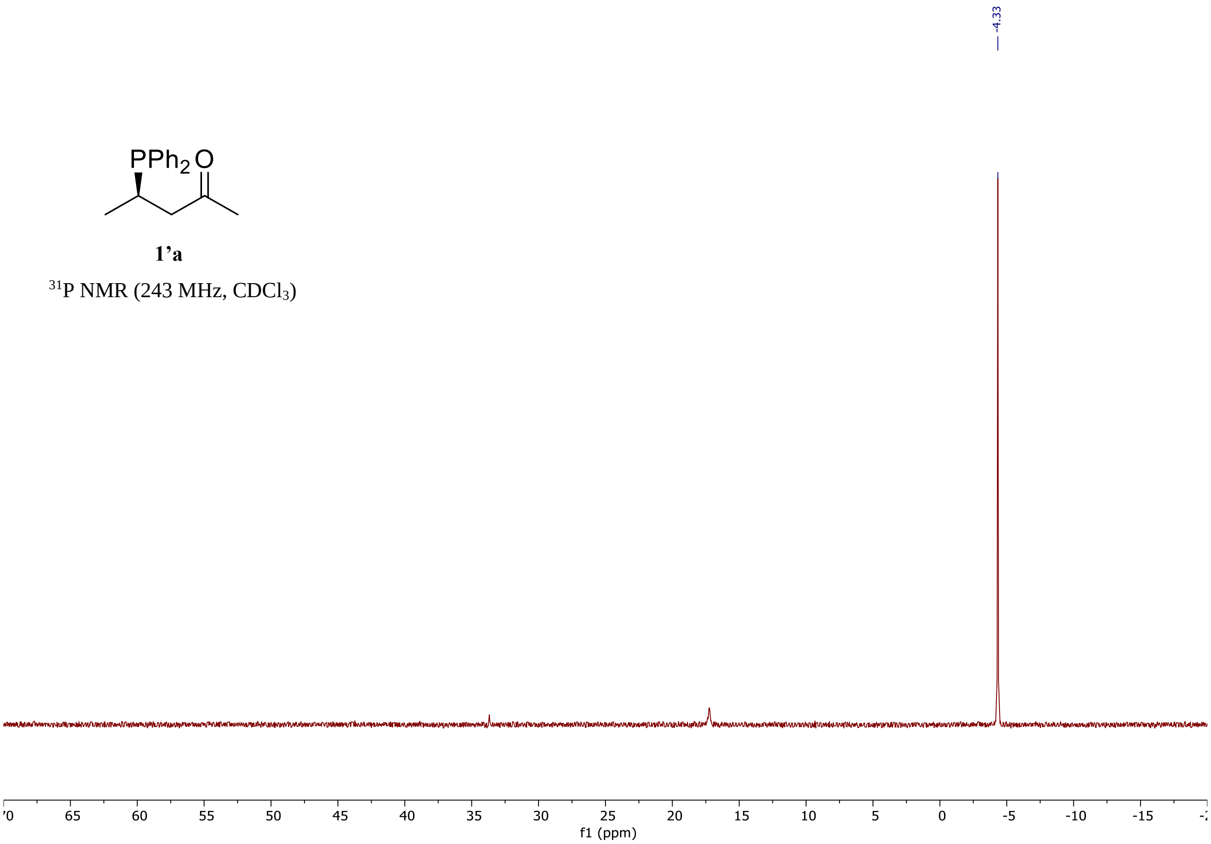

1’a

^{31}P NMR (243 MHz, $CDCl_3$)

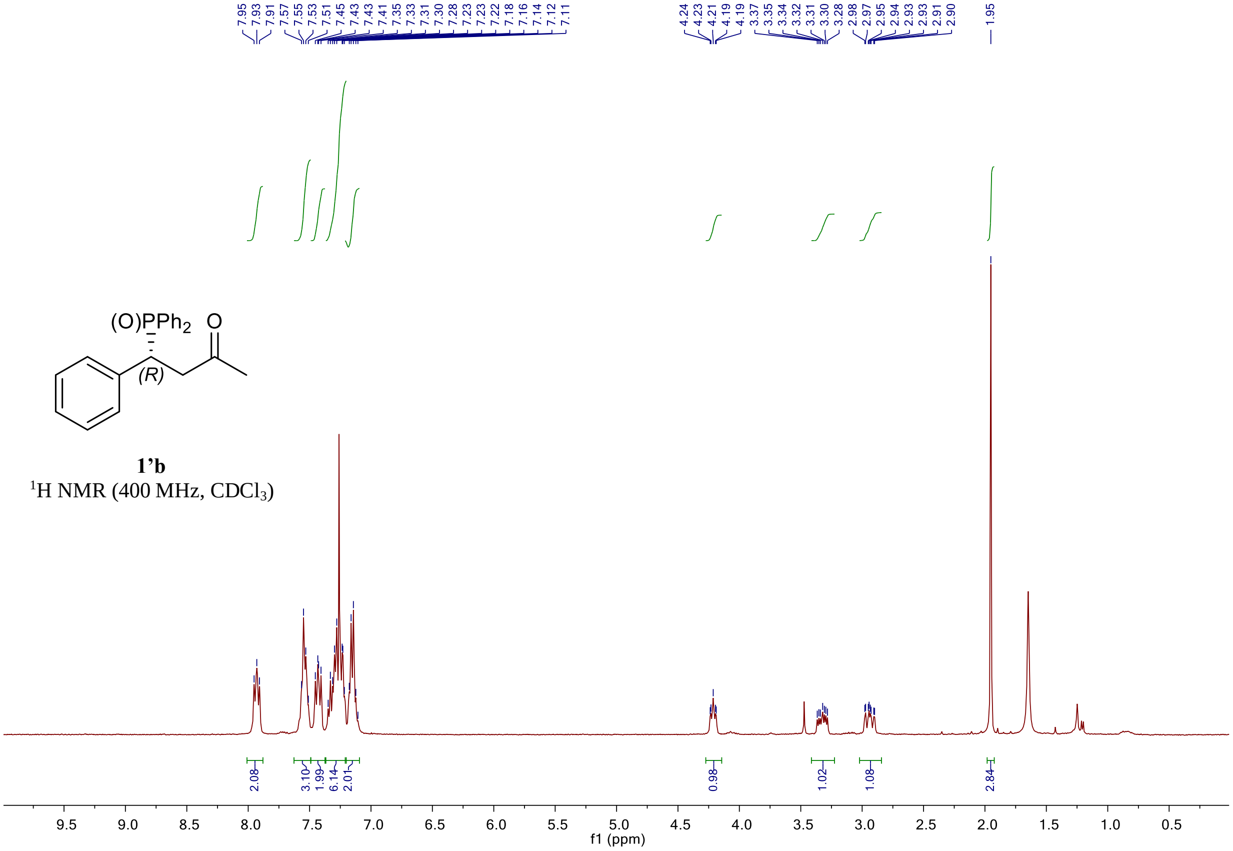

1’b

^{1}H NMR (400 MHz, $CDCl_3$)

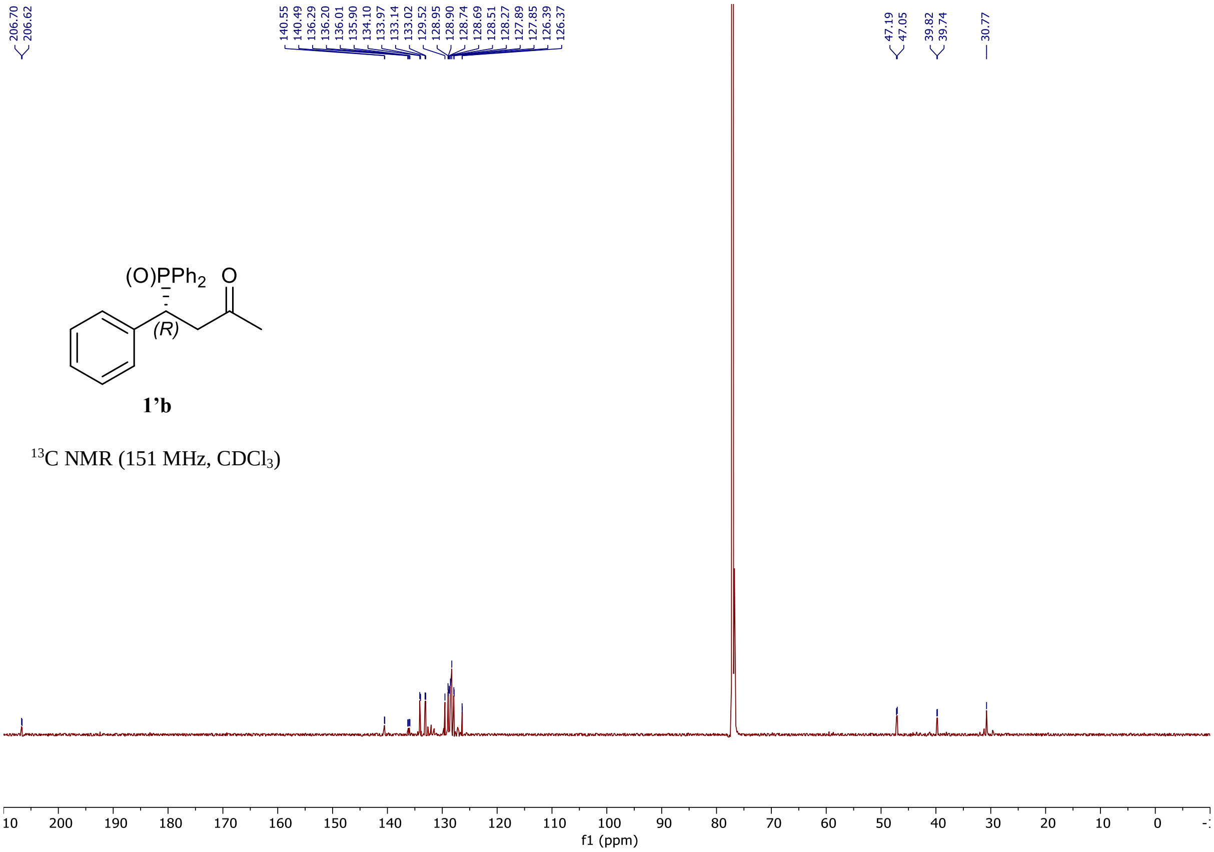

1'b

^{13}C NMR (151 MHz, $CDCl_3$)

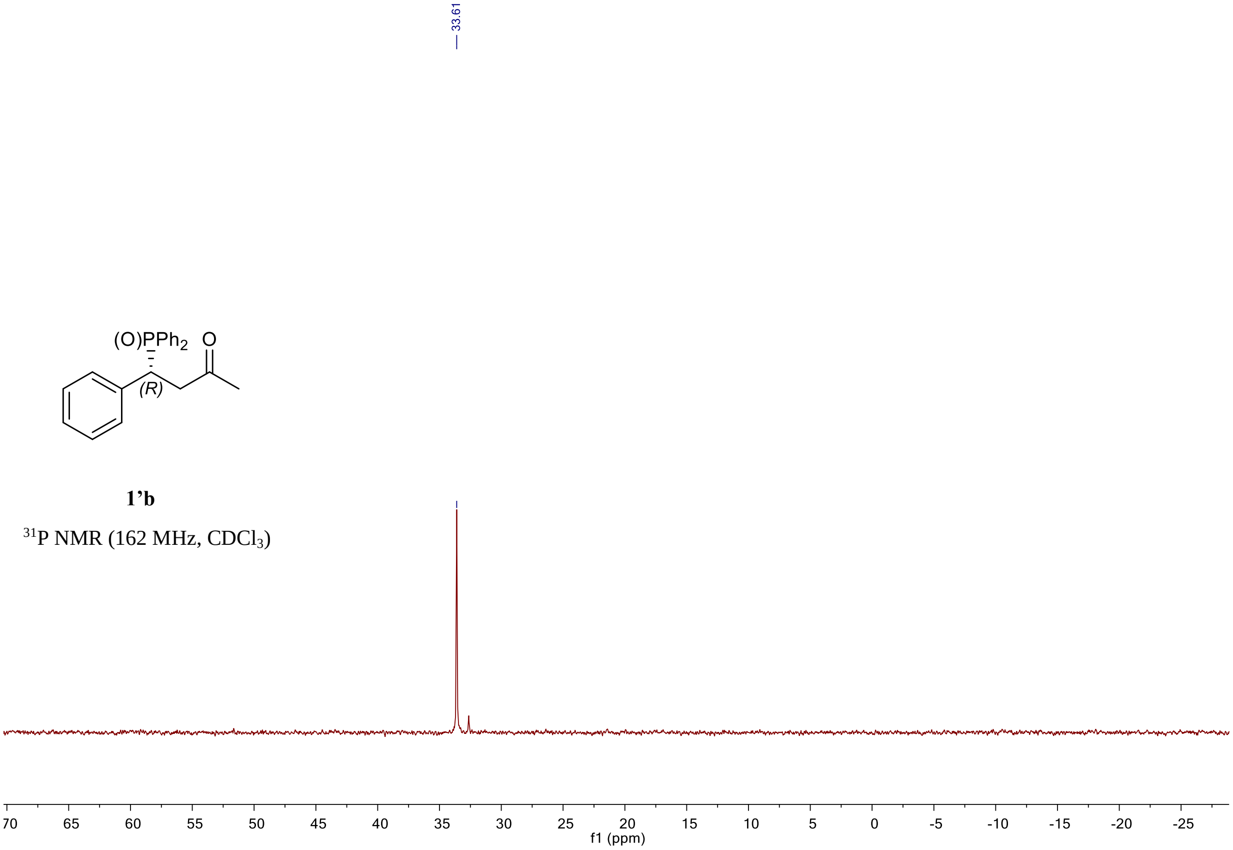

1'b

^{31}P NMR (162 MHz, $CDCl_3$)

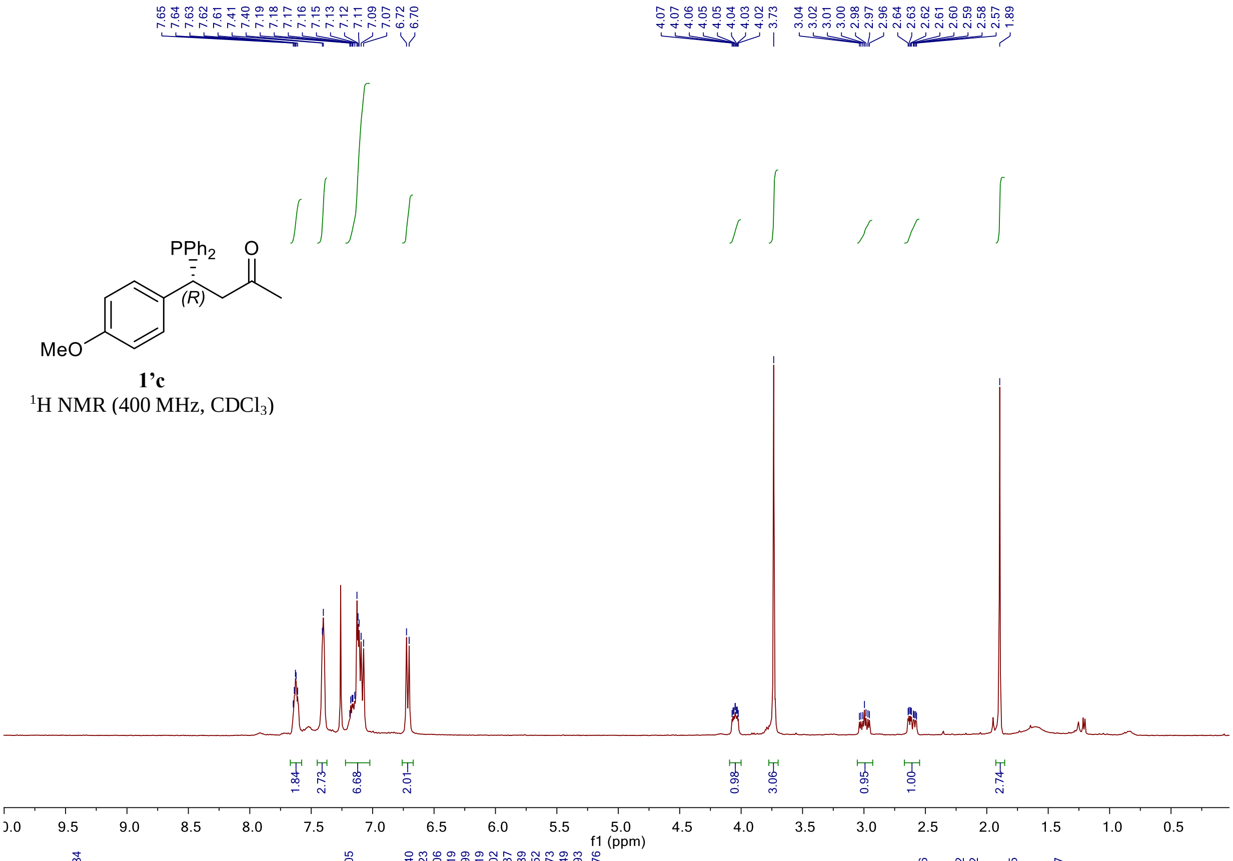

1’c

^{1}H NMR (400 MHz, $CDCl_3$)

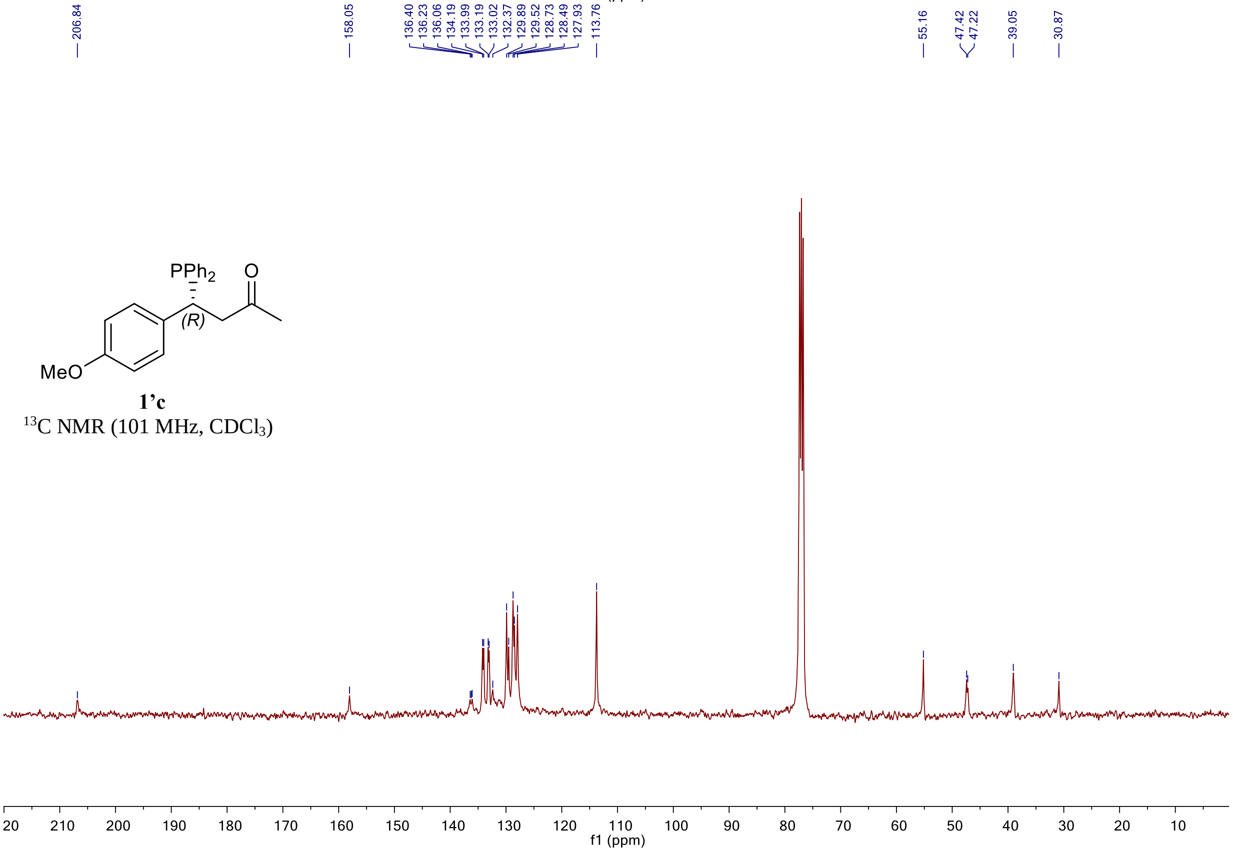

1’c

^{13}C NMR (101 MHz, $CDCl_3$)

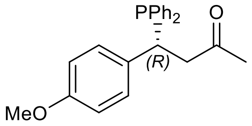

1’c

^{31}P NMR (162 MHz, $CDCl_3$)

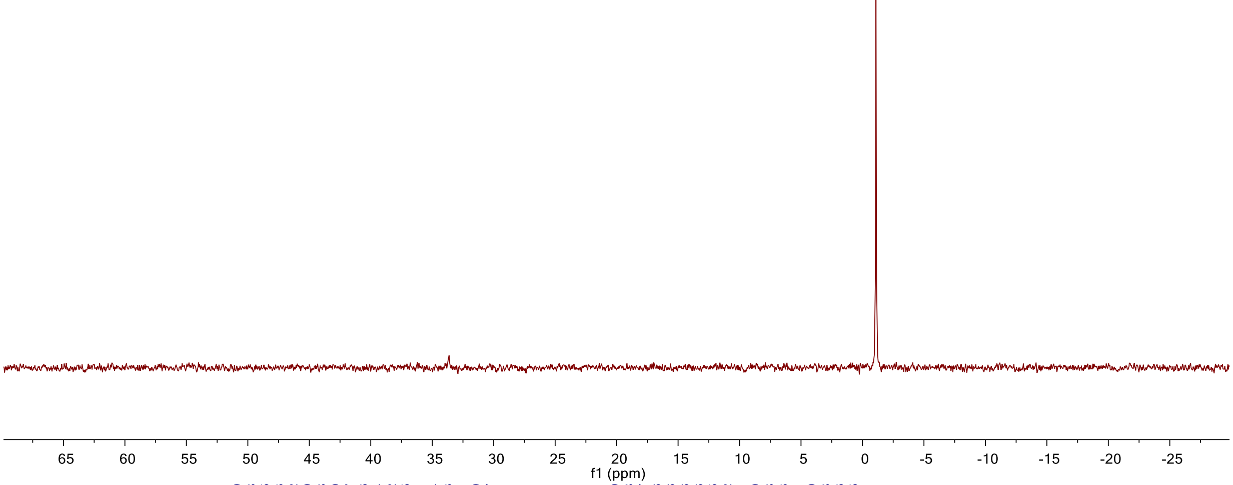

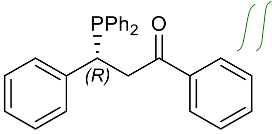

1’d

^{1}H NMR (400 MHz, $CDCl_3$)

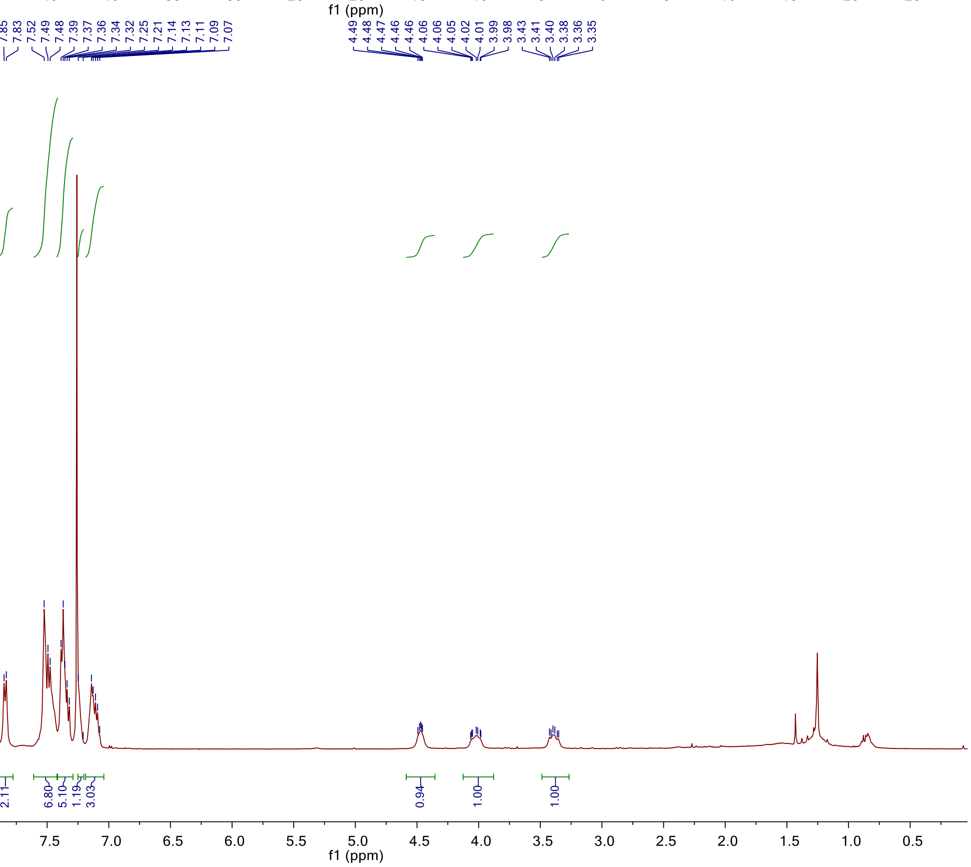

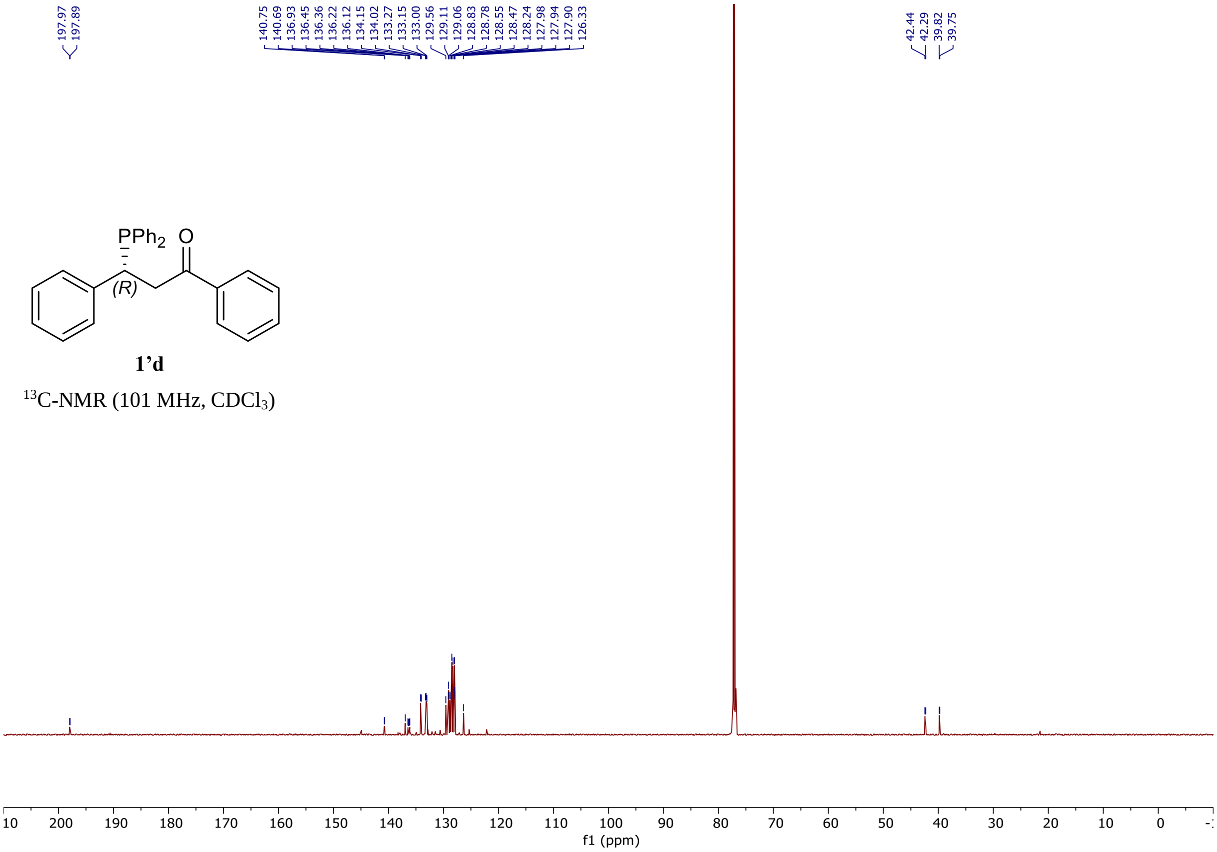

1'd

^{13}C-NMR (101 MHz, $CDCl_3$)

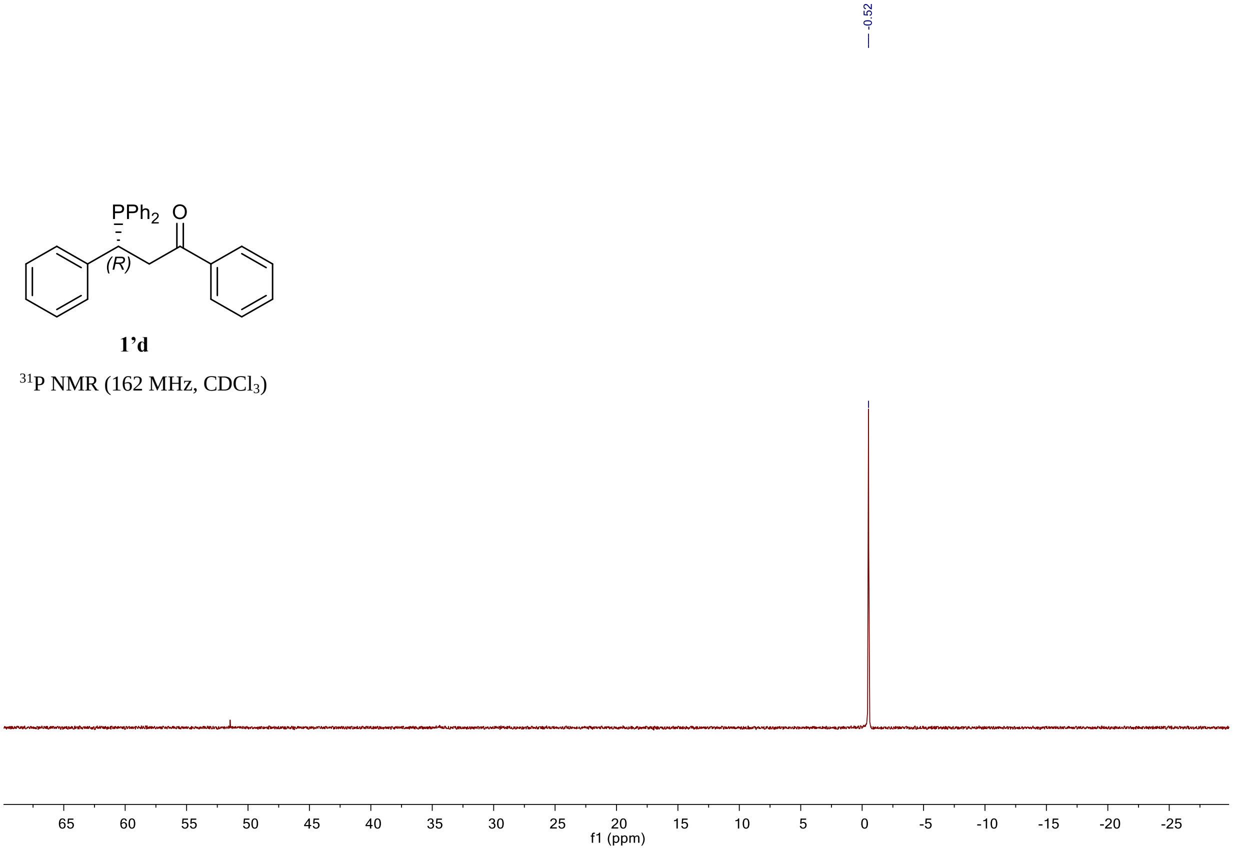

1'd

^{31}P NMR (162 MHz, $CDCl_3$)

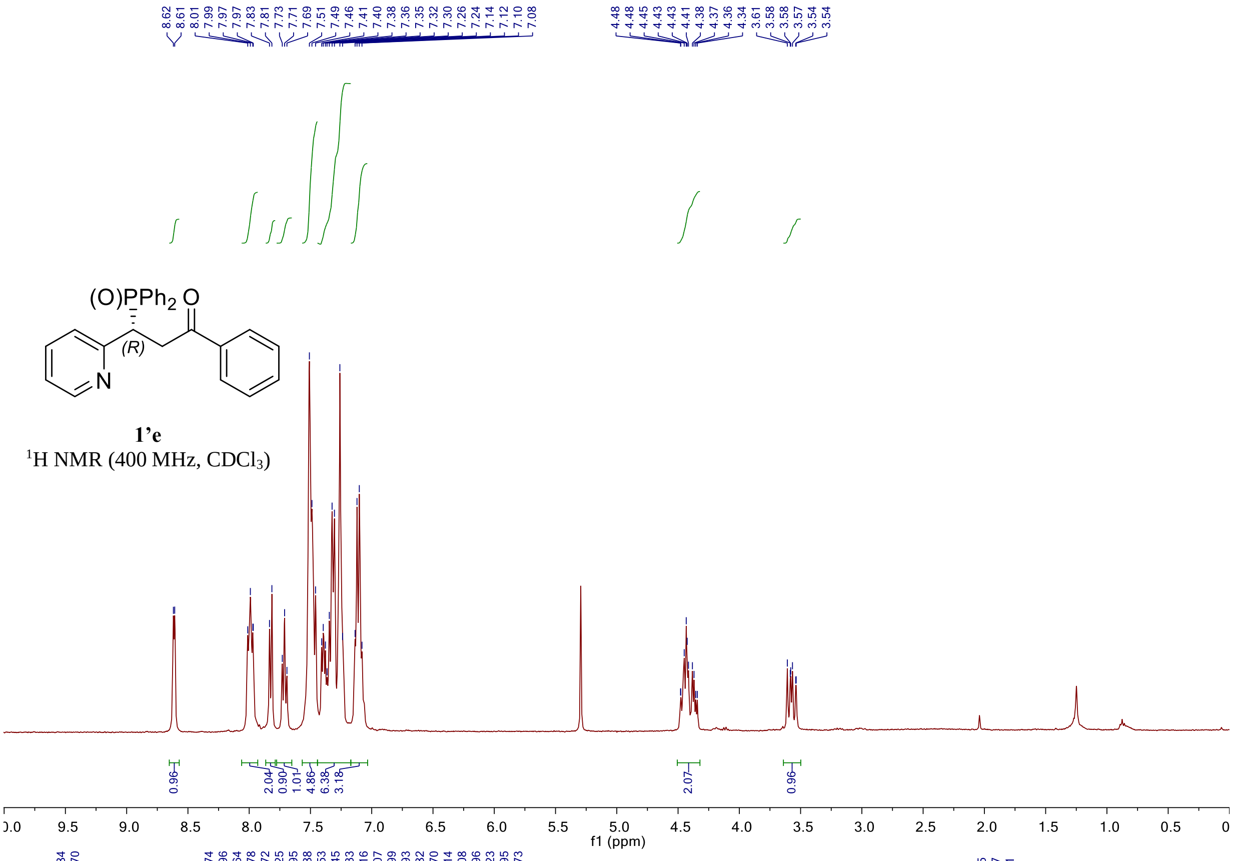

1’e
^{1}H NMR (400 MHz, $CDCl_3$)

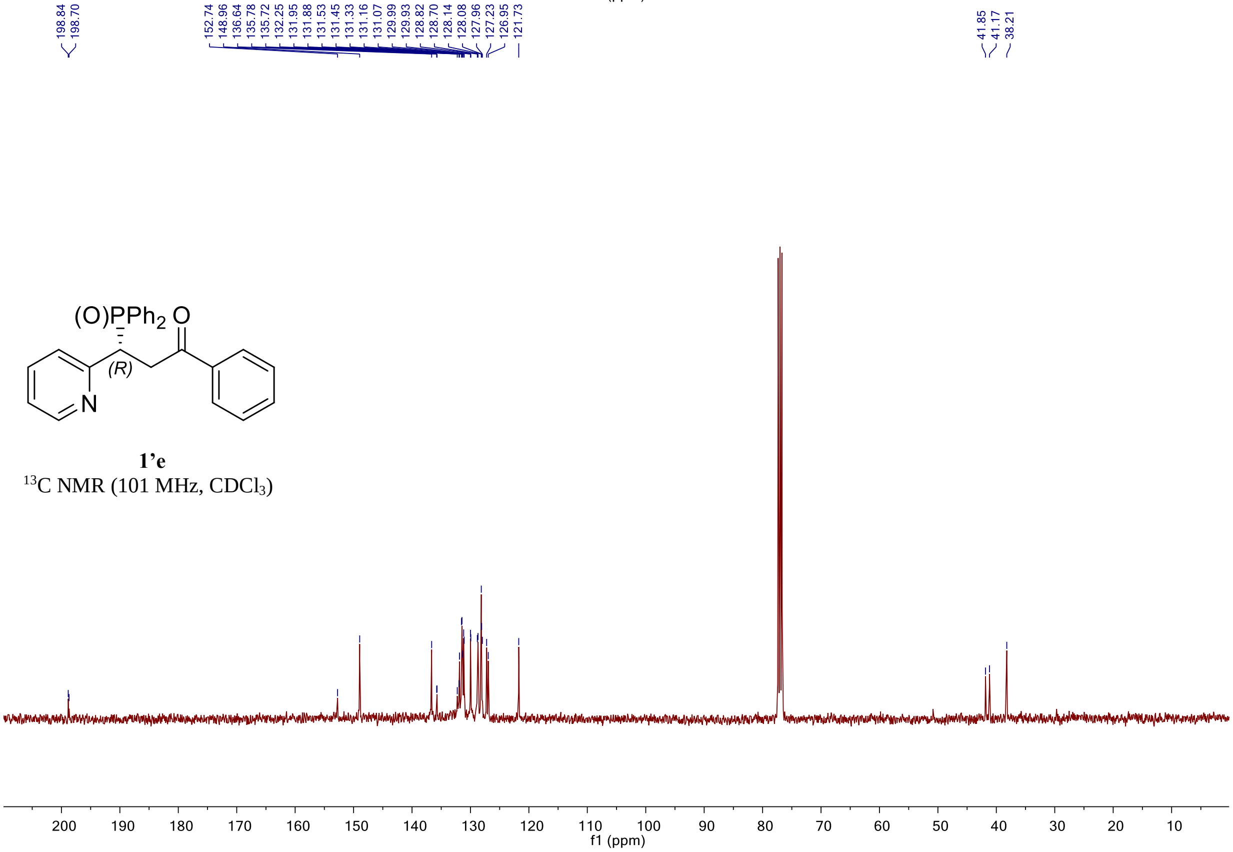

1’e
^{13}C NMR (101 MHz, $CDCl_3$)

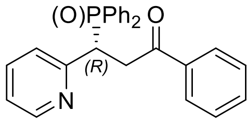

1’e

^{31}P NMR (162 MHz, $CDCl_3$)

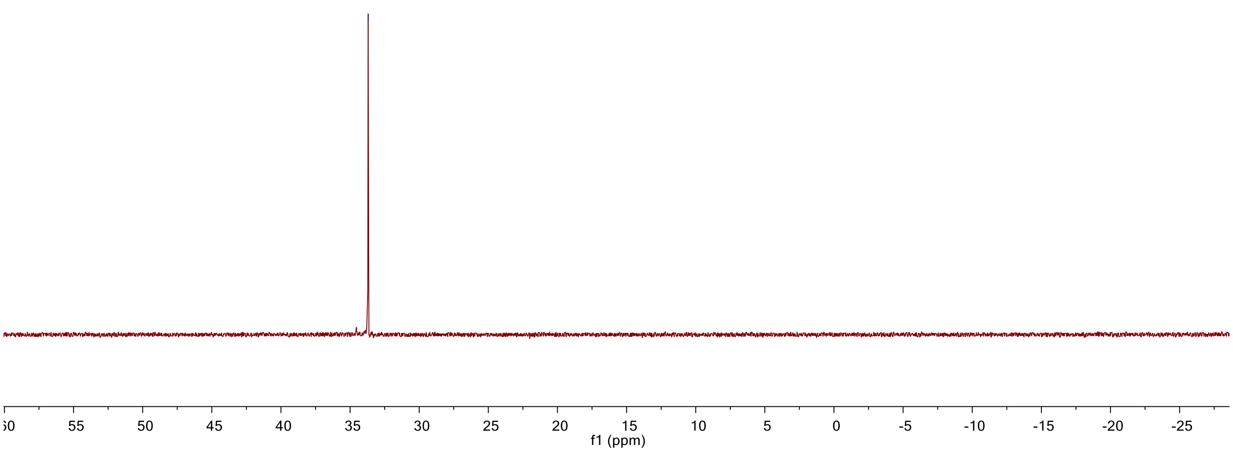

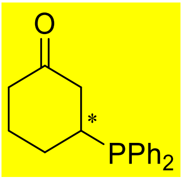

1’f

^{1}H NMR (400 MHz, $CDCl_3$)

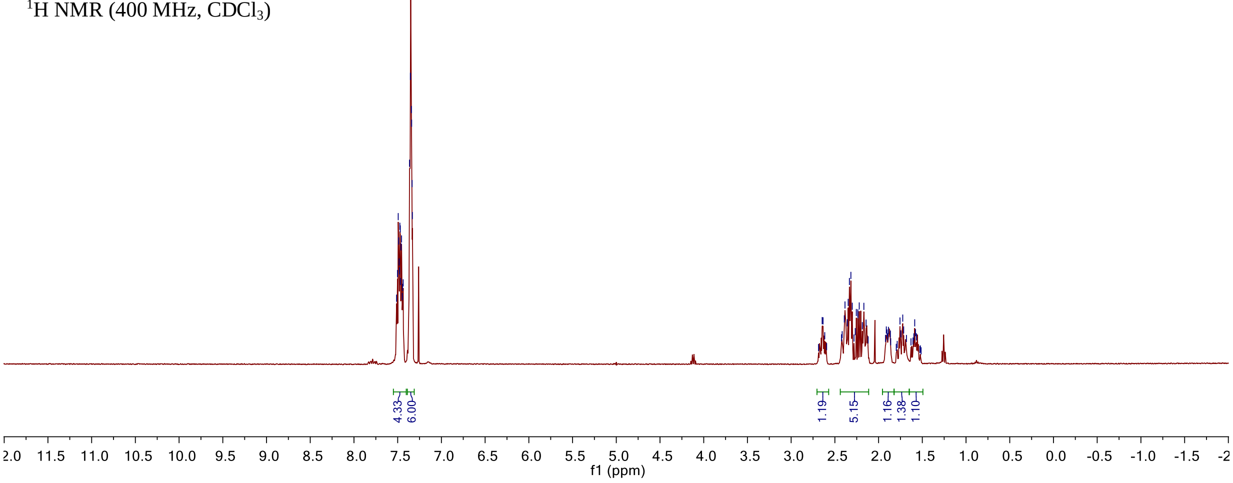

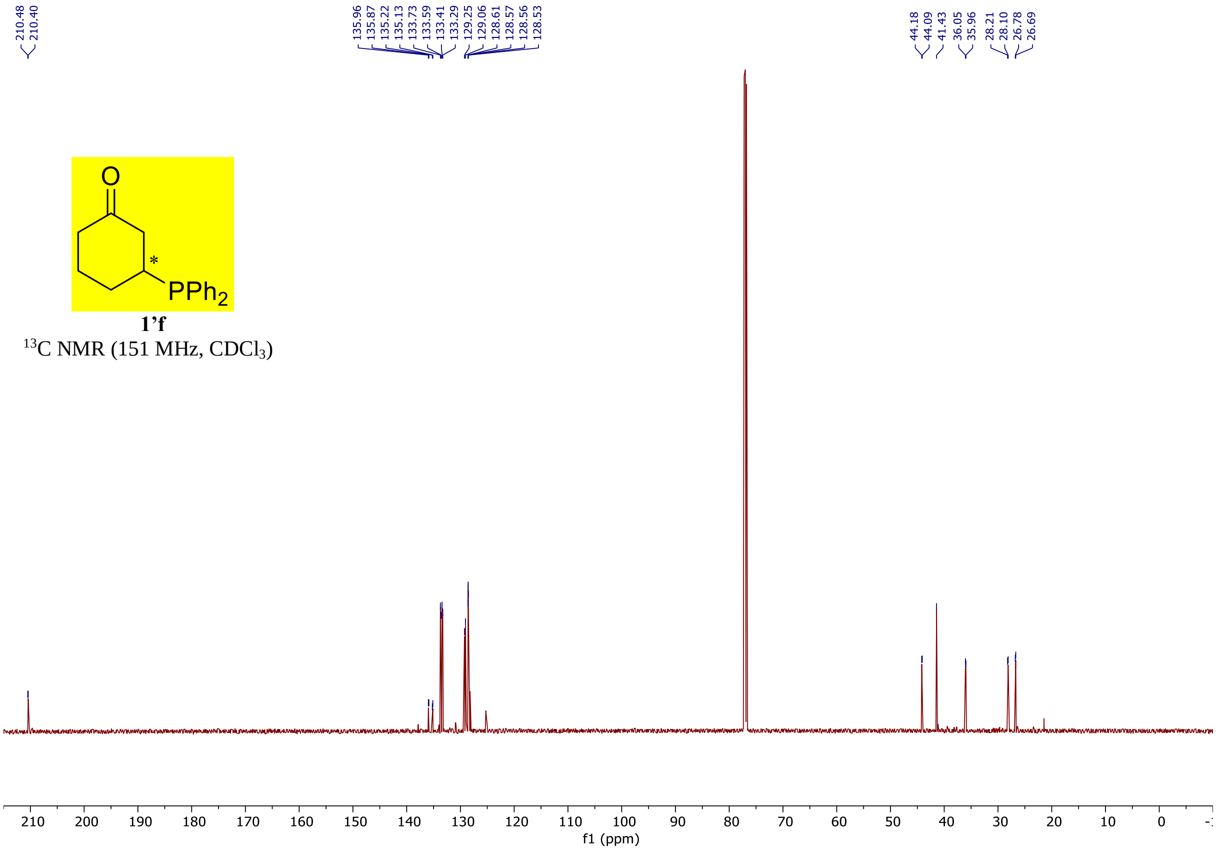

1’f
^{13}C NMR (151 MHz, $CDCl_3$)

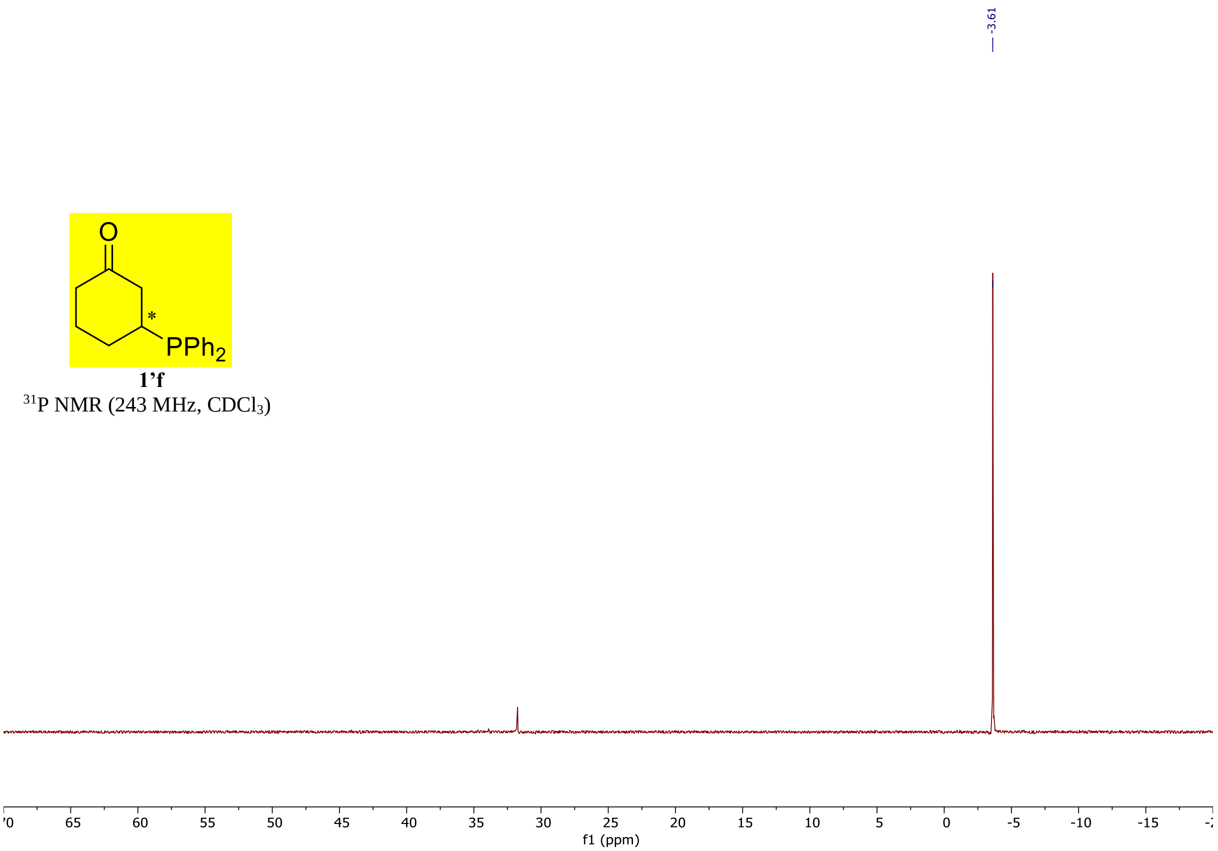

1’f
^{31}P NMR (243 MHz, $CDCl_3$)

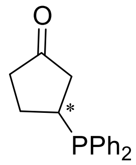

1'g

^{1}H NMR (600 MHz, $CDCl_3$)

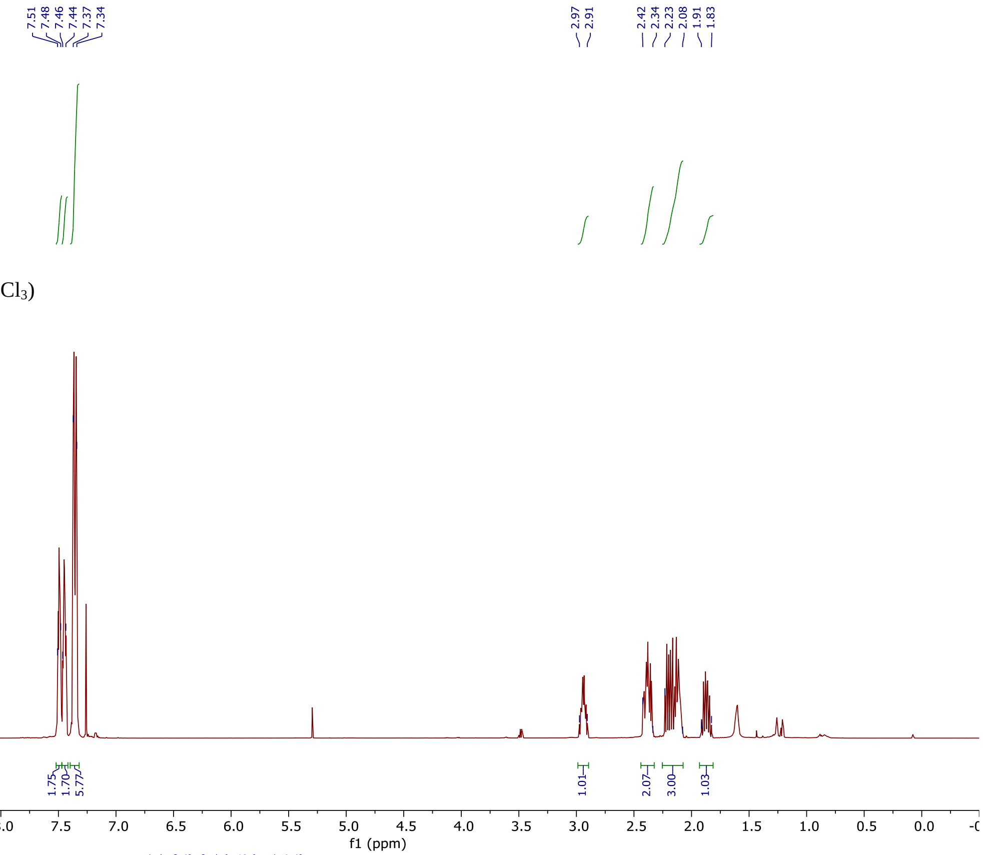

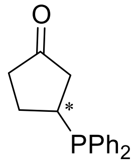

1'g

^{13}C NMR (151 MHz, $CDCl_3$)

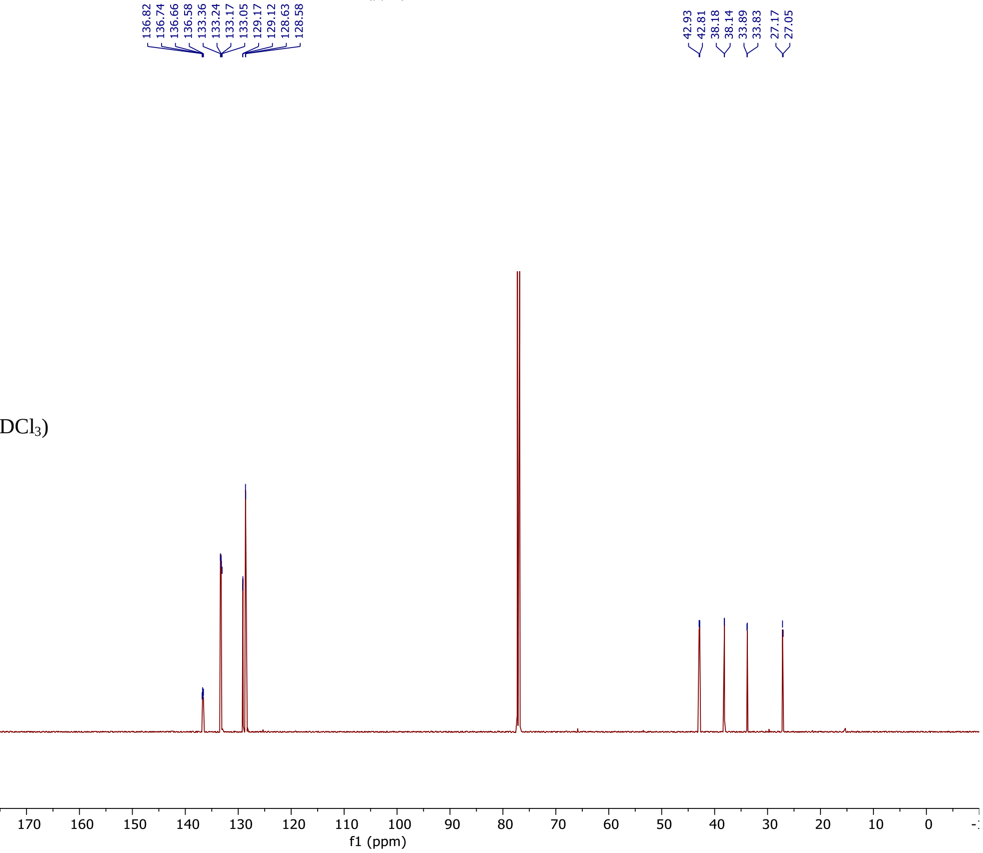

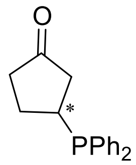

1'g

^{31}P NMR (243 MHz, $CDCl_3$)

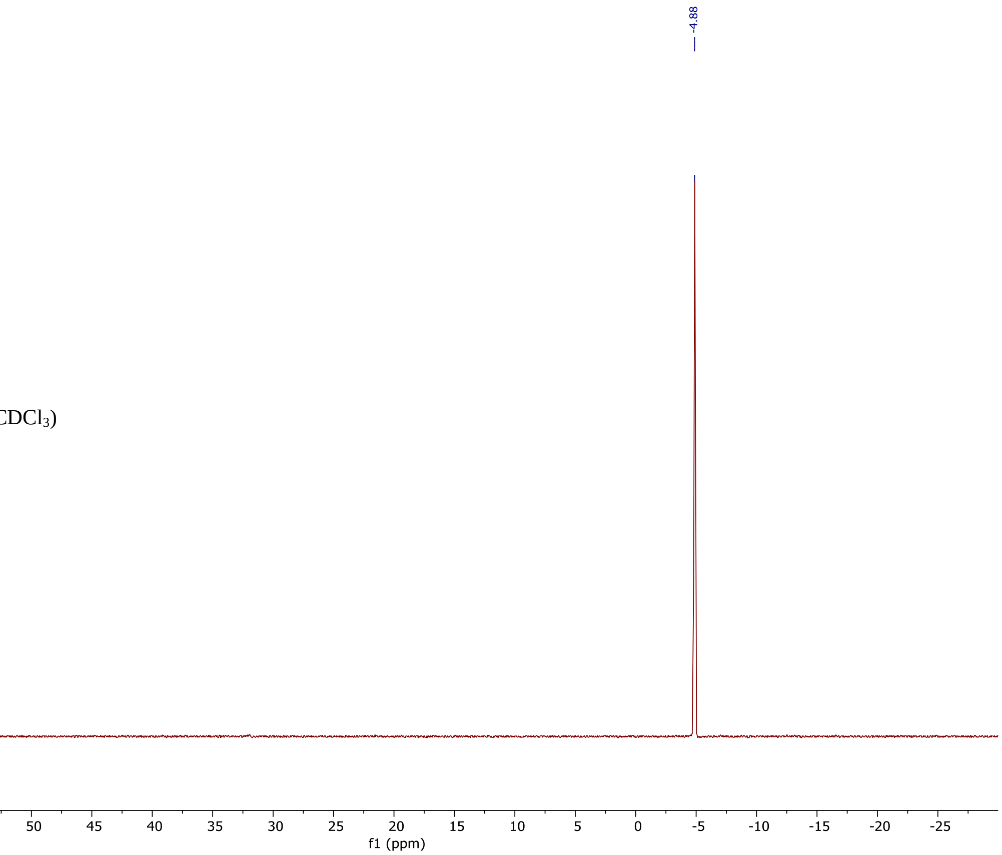

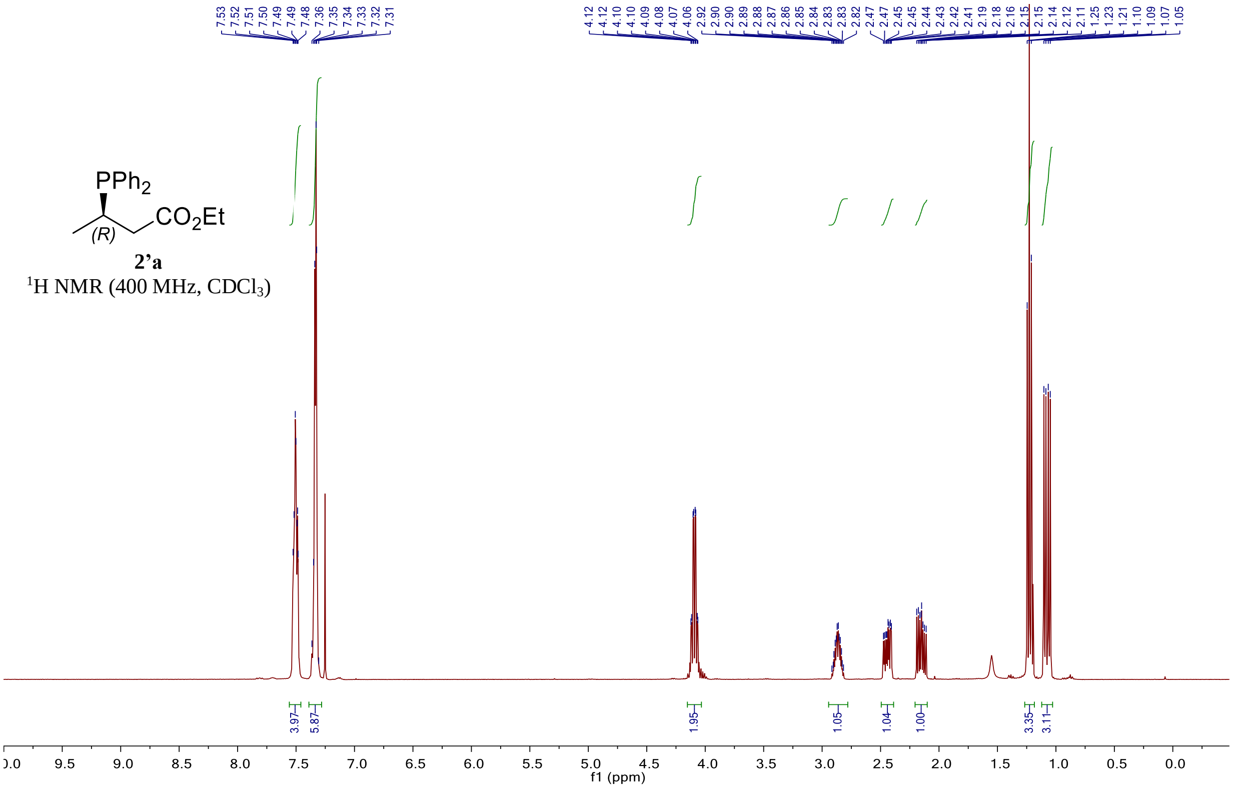

2'a

^{1}H NMR (400 MHz, $CDCl_3$)

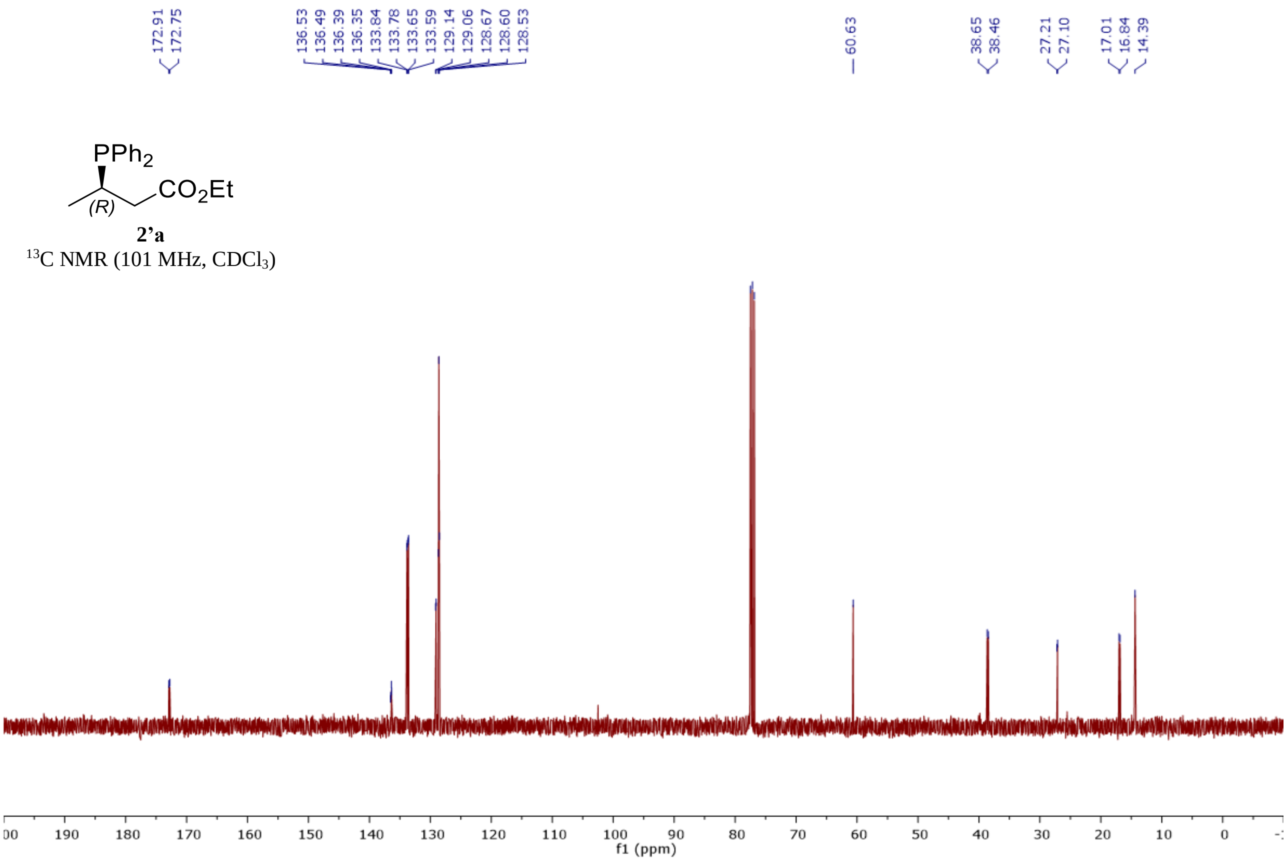

2'a
^{13}C NMR (101 MHz, $CDCl_3$)

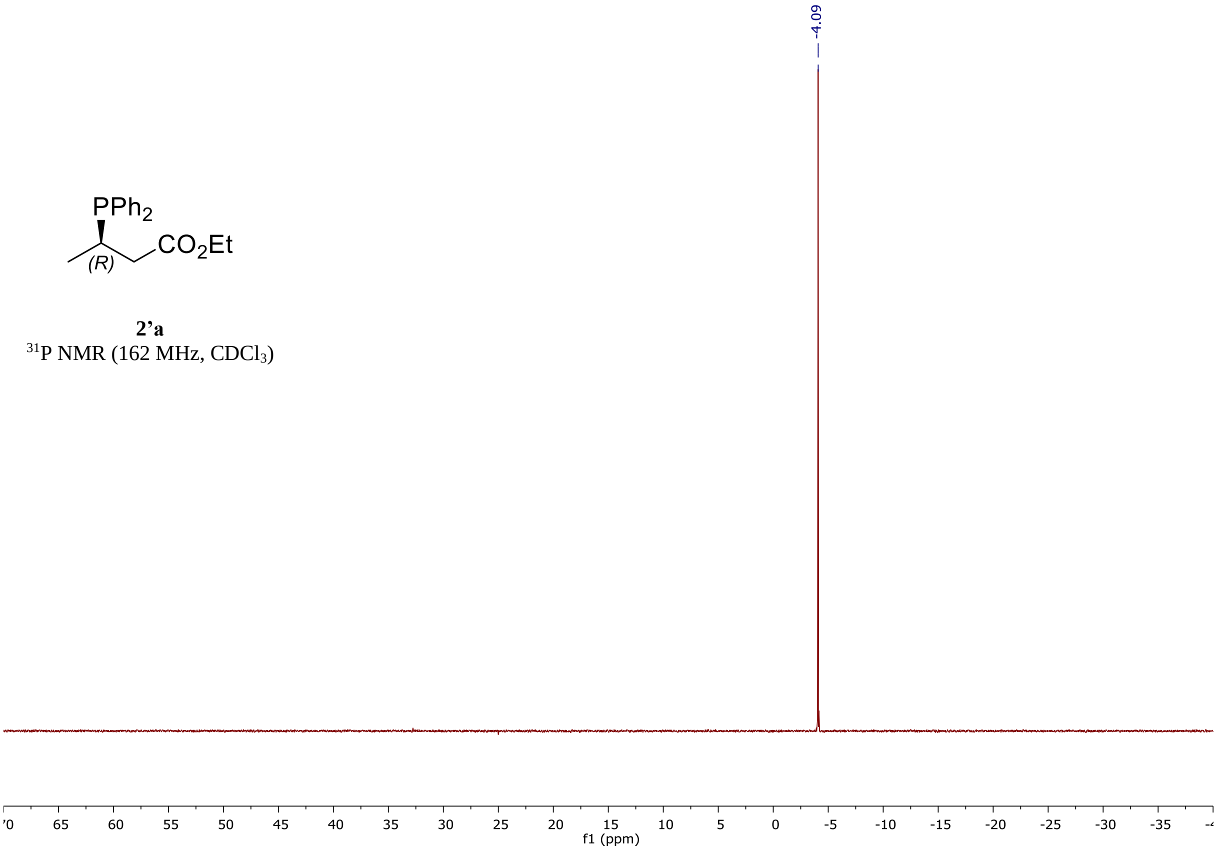

2'a
^{31}P NMR (162 MHz, $CDCl_3$)

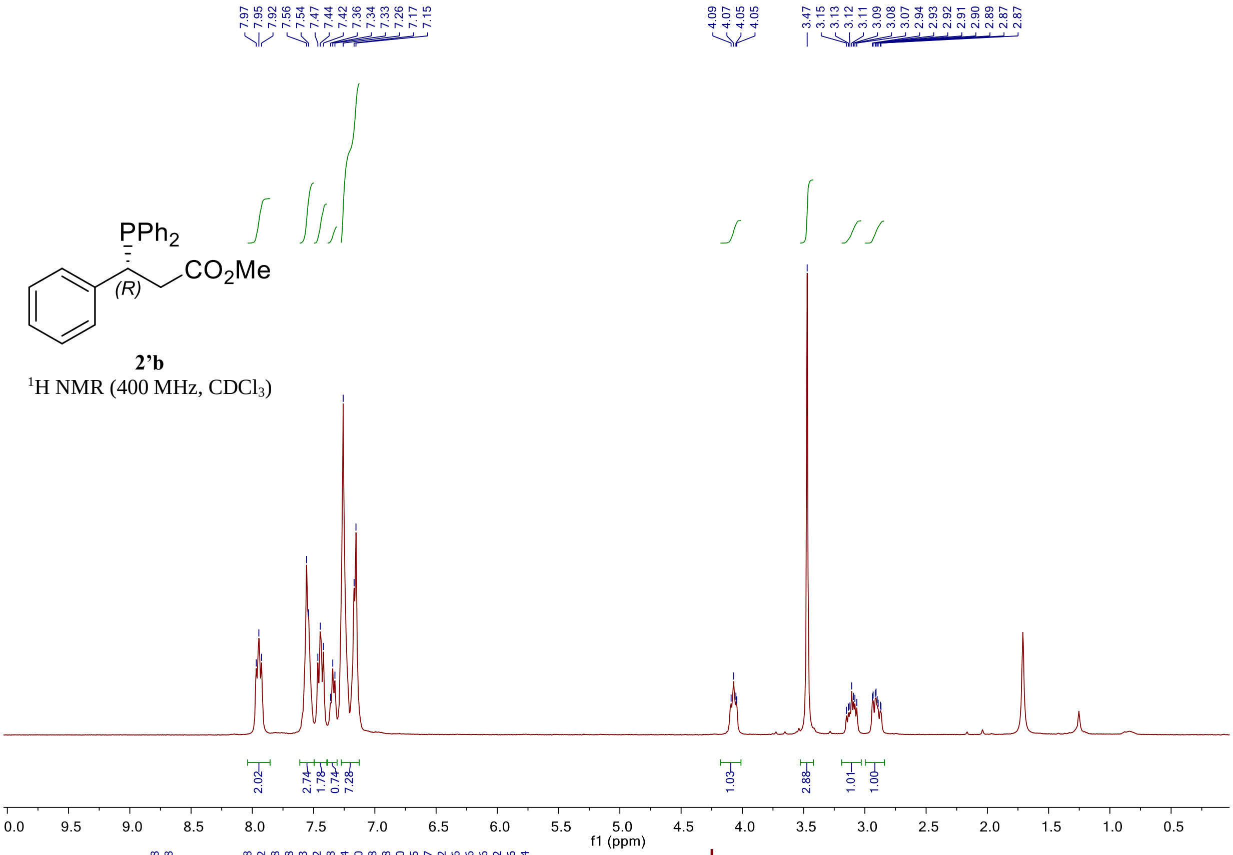

2'b

^{1}H NMR (400 MHz, $CDCl_3$)

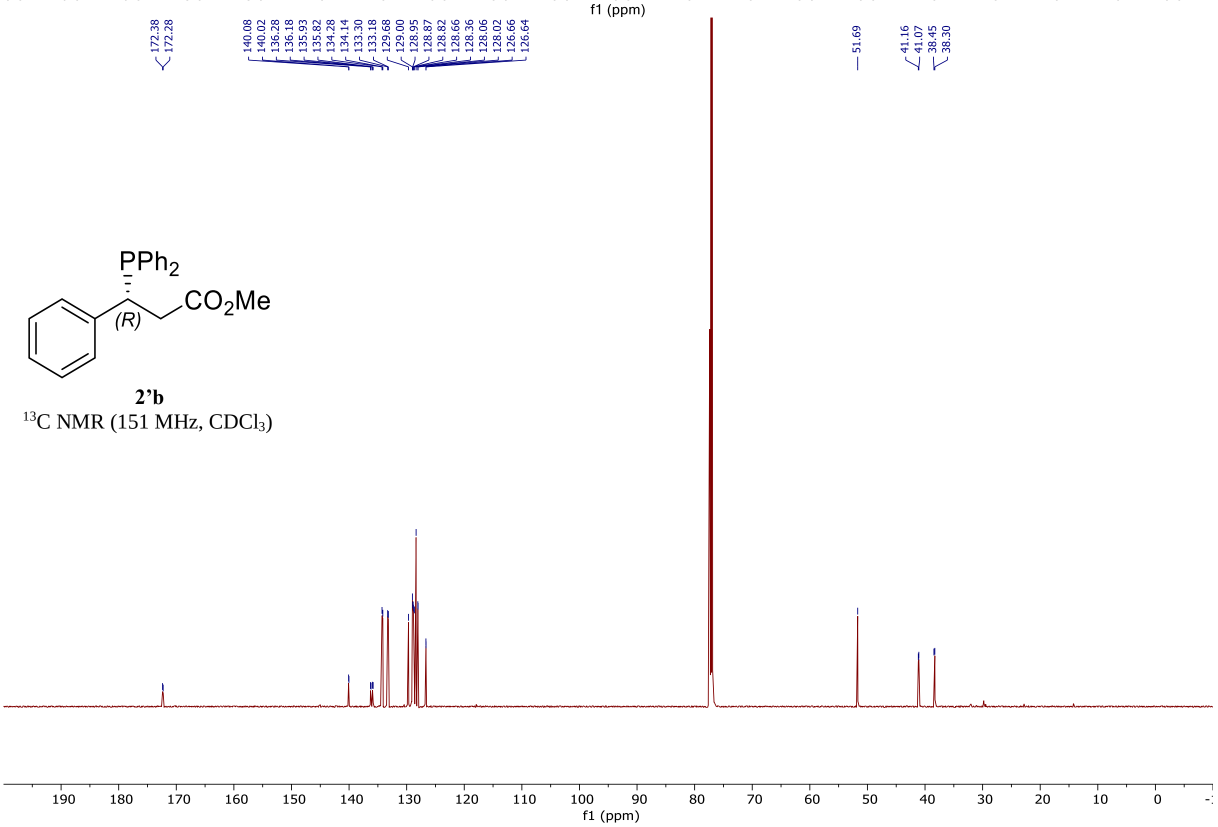

2'b

^{13}C NMR (151 MHz, $CDCl_3$)

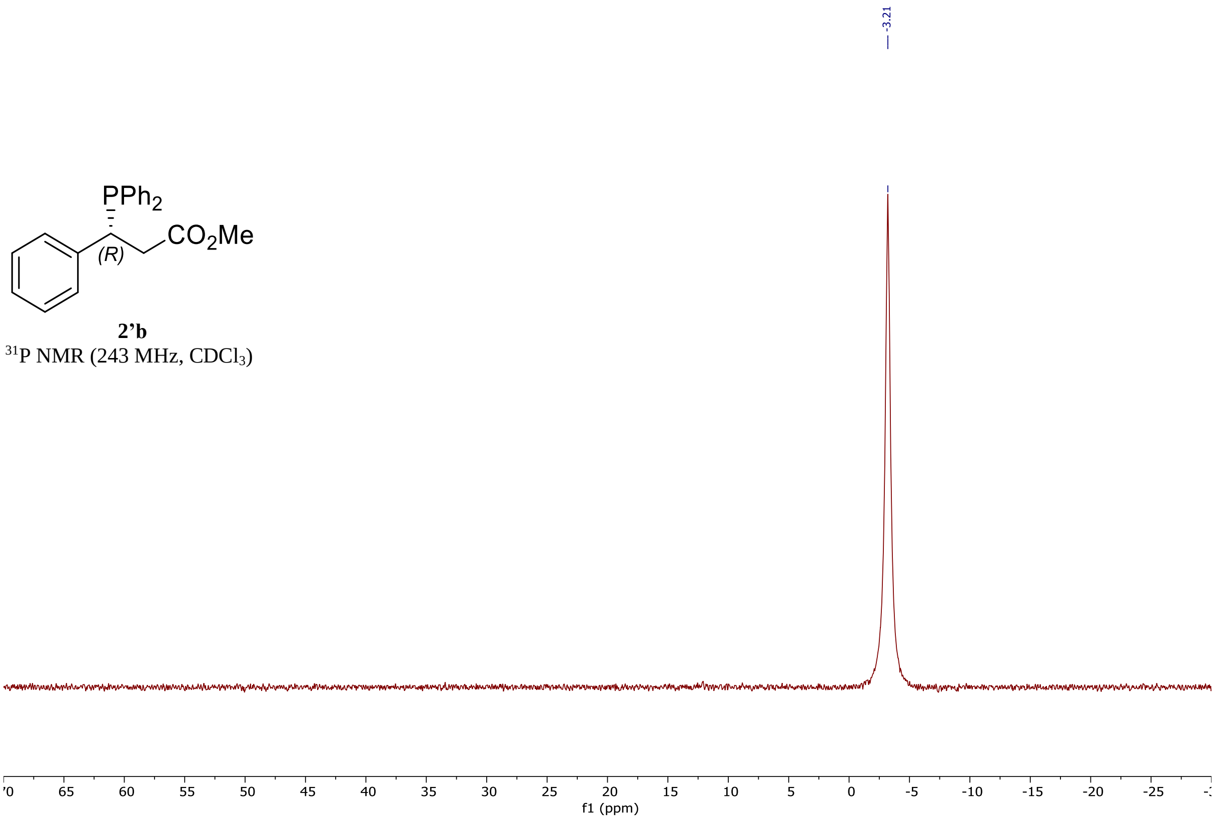

2’b

^{31}P NMR (243 MHz, $CDCl_3$)

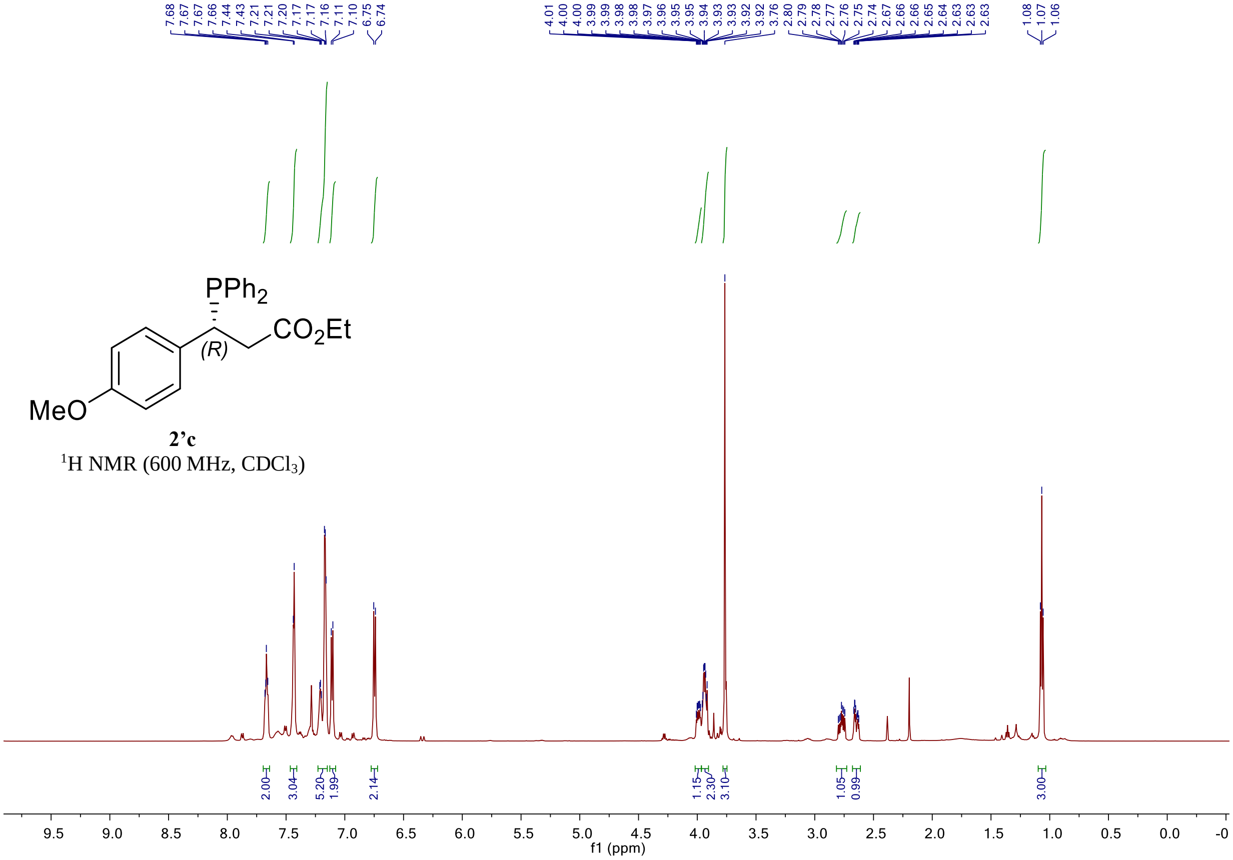

2’c

^{1}H NMR (600 MHz, $CDCl_3$)

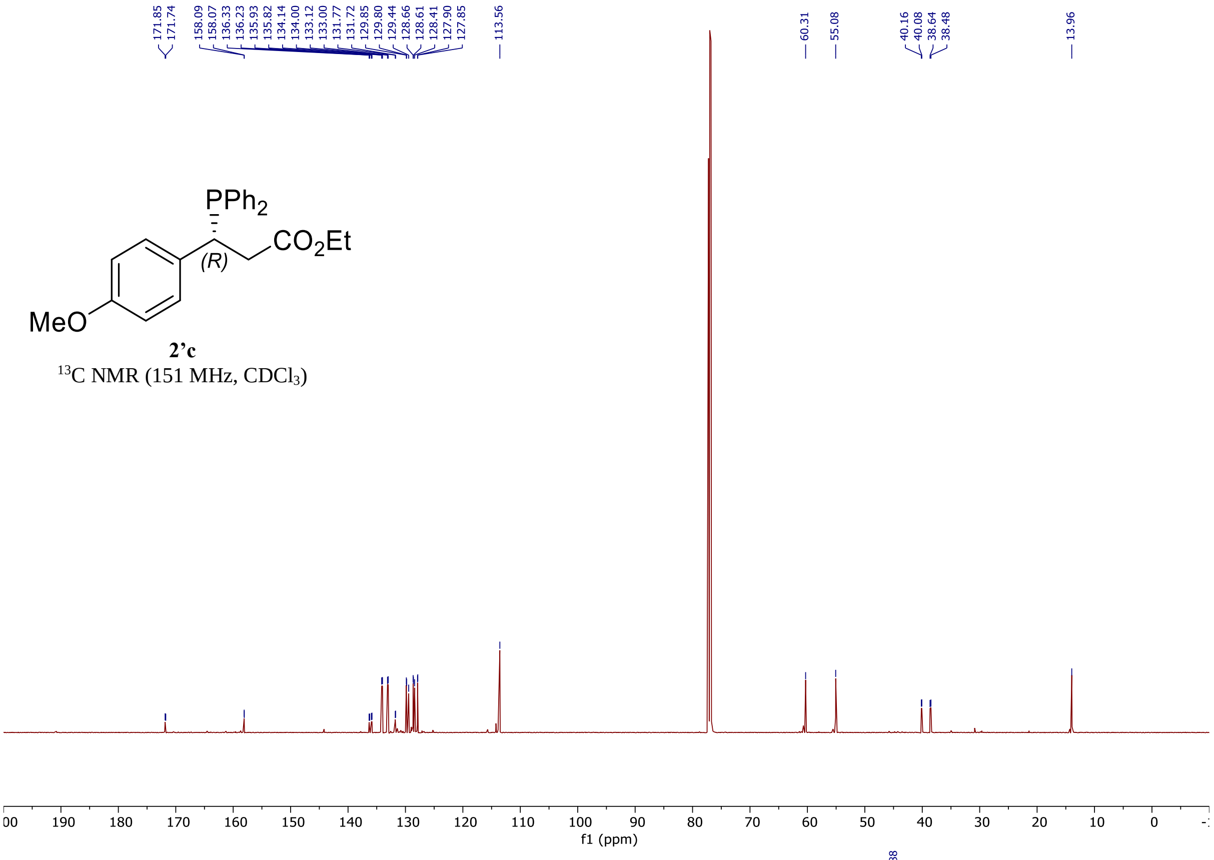

2'c

^{13}C NMR (151 MHz, $CDCl_3$)

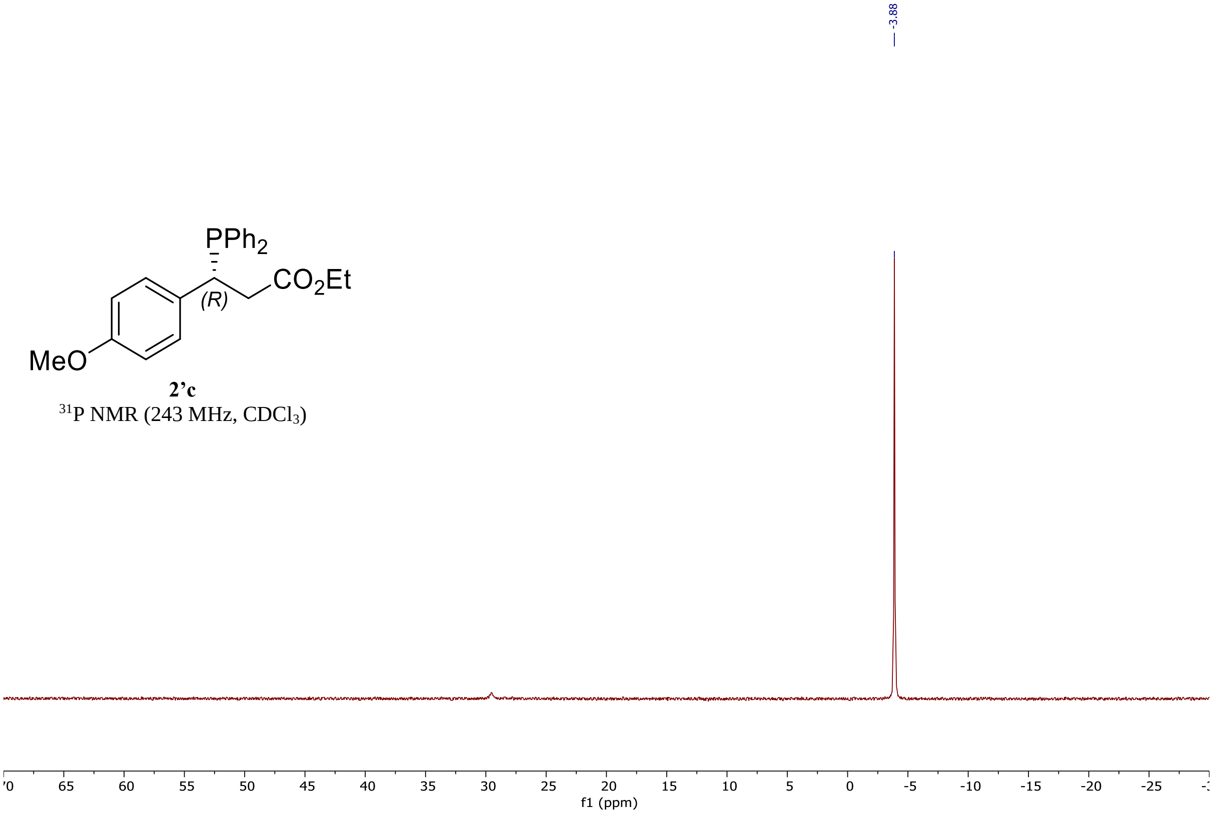

2'c

^{31}P NMR (243 MHz, $CDCl_3$)

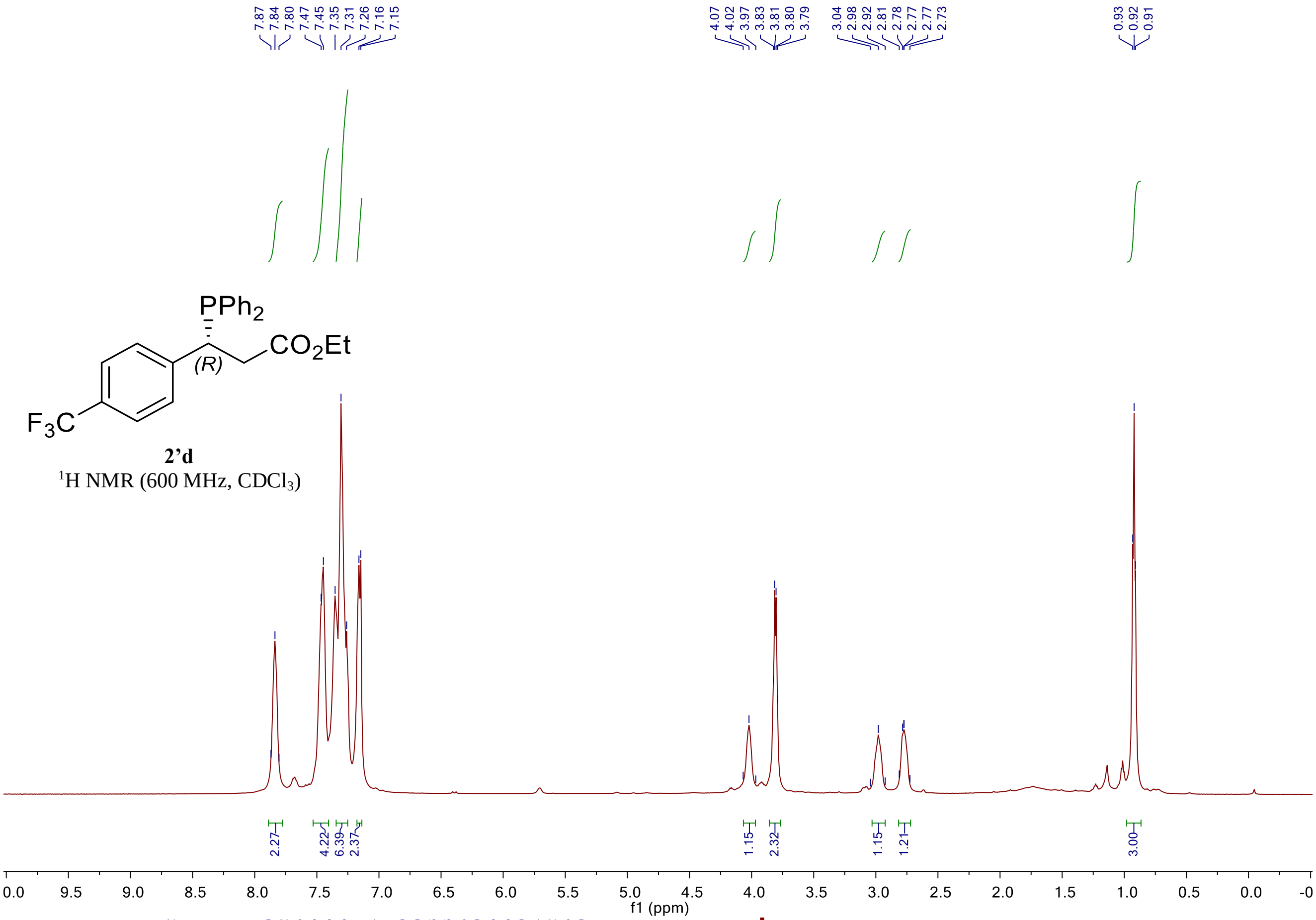

2’d

^{1}H NMR (600 MHz, $CDCl_3$)

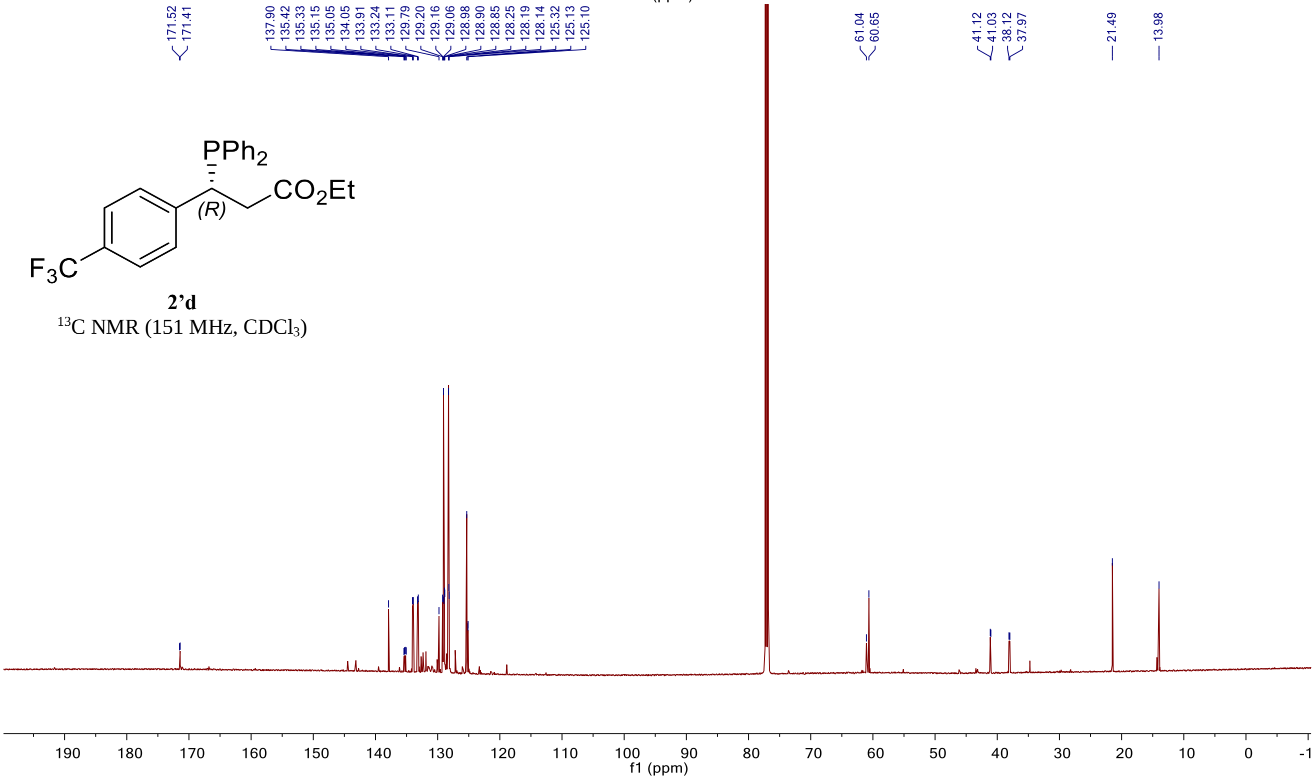

2’d

^{13}C NMR (151 MHz, $CDCl_3$)

Supplementary Material

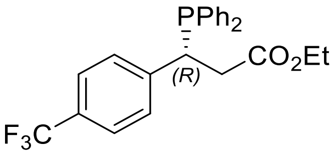

2’d

^{31}P NMR (243 MHz, $CDCl_3$)

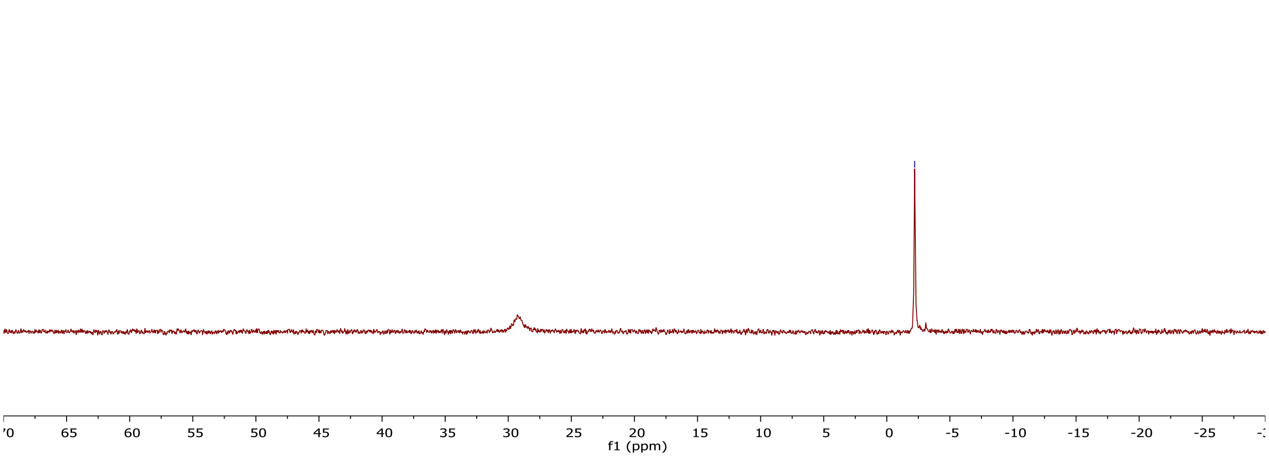

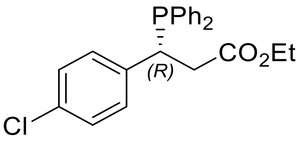

2’e

^{1}H NMR (400 MHz, $CDCl_3$)

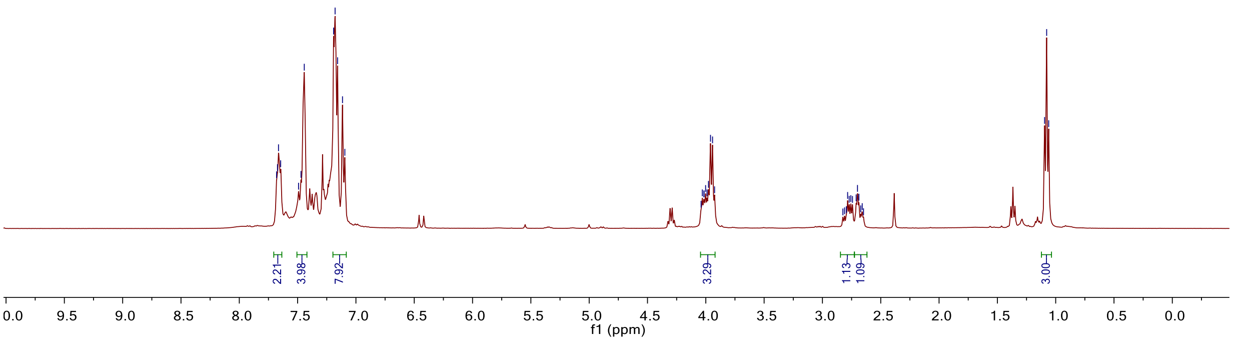

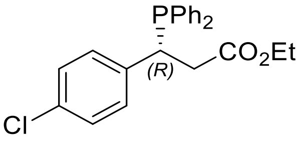

2'e

^{13}C NMR (151 MHz, CDCl$_3$

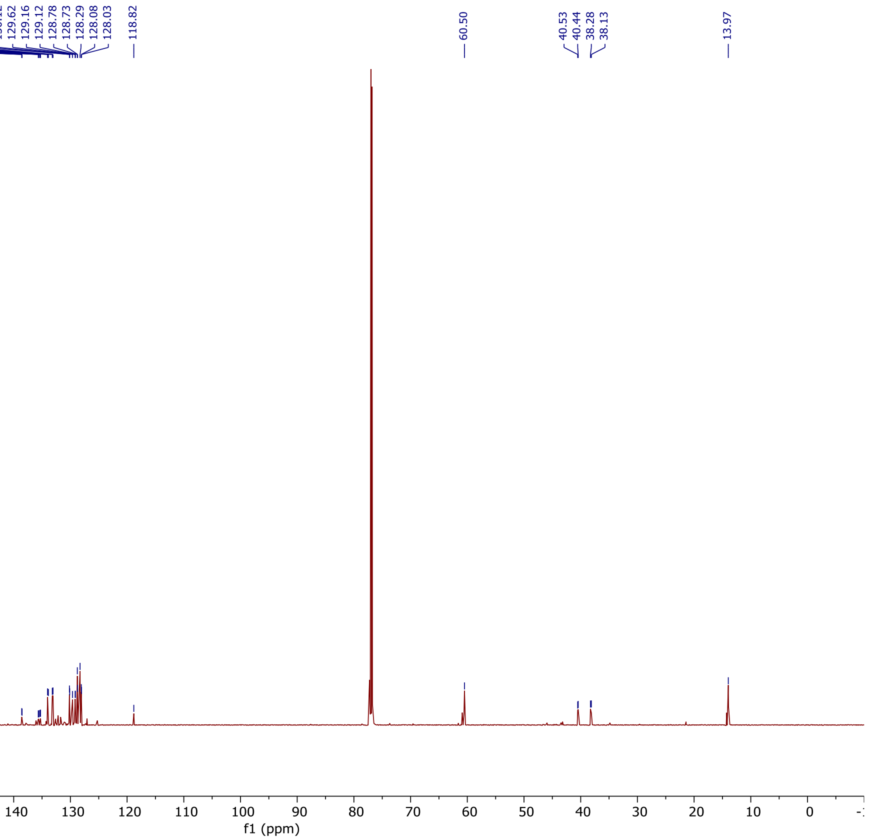

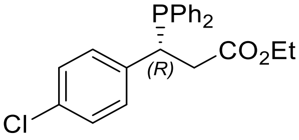

2'e

^{31}P NMR (243 MHz, CDCl$_3$)

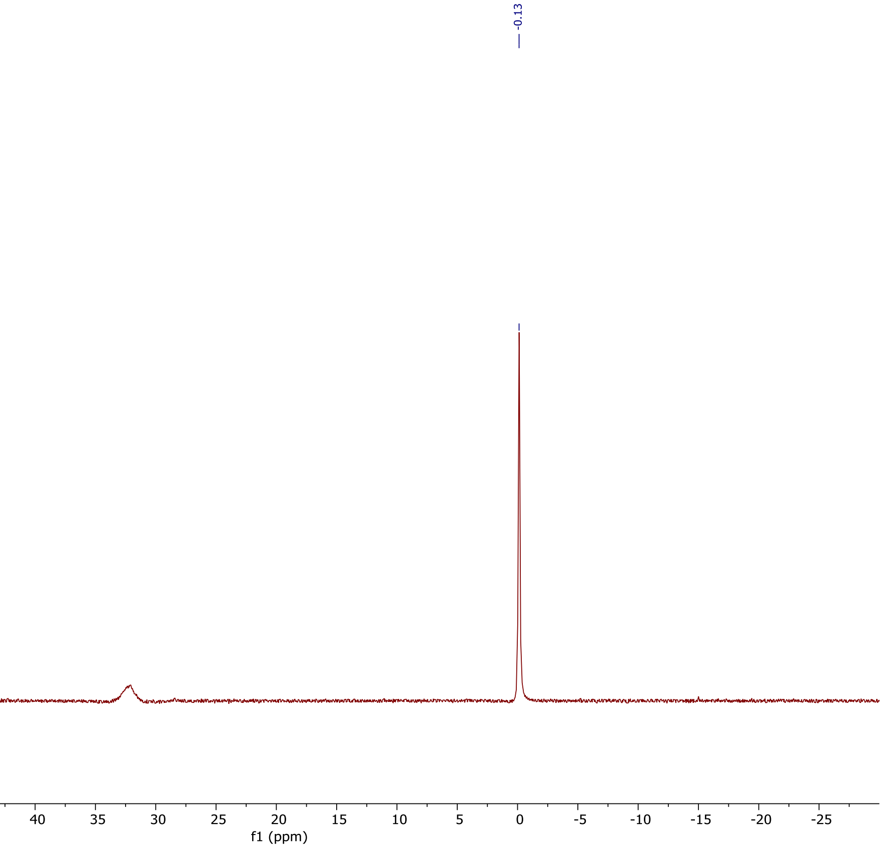

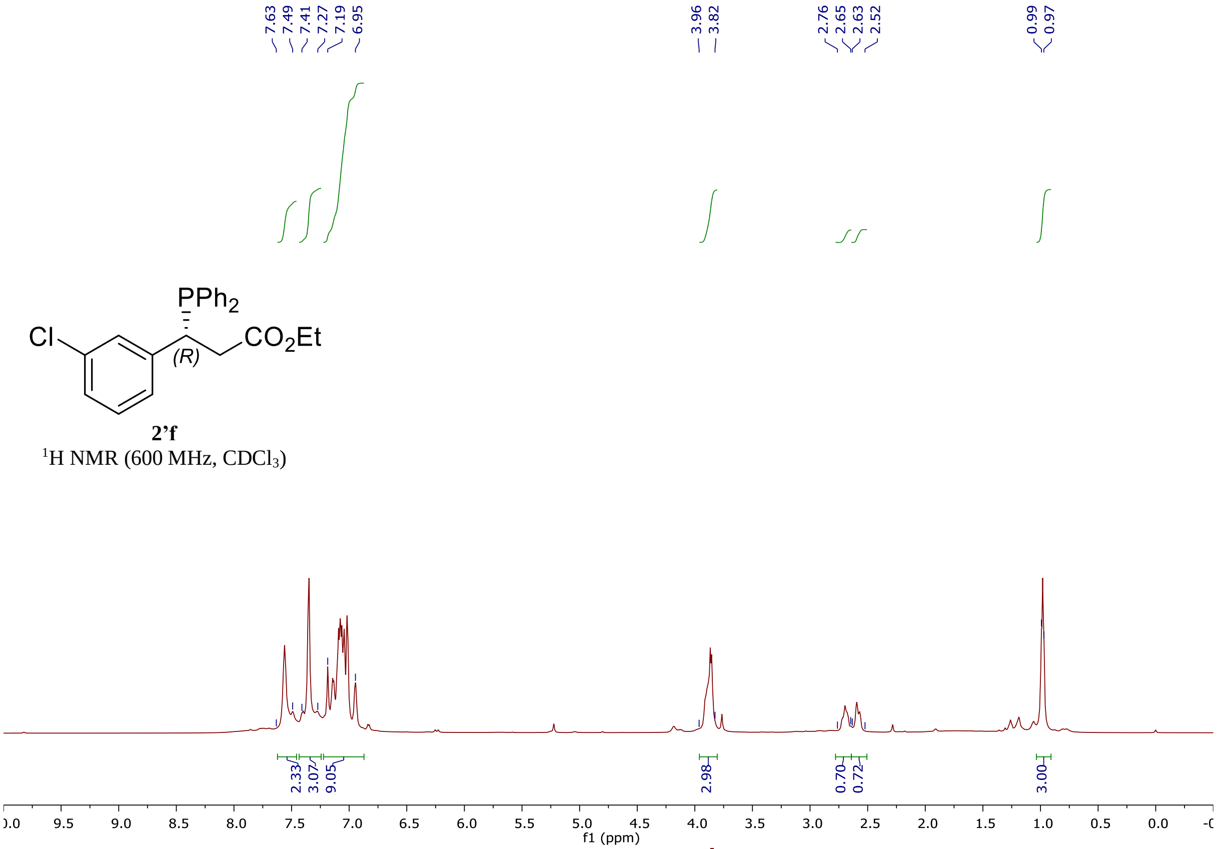

2'f

^{1}H NMR (600 MHz, $CDCl_3$)

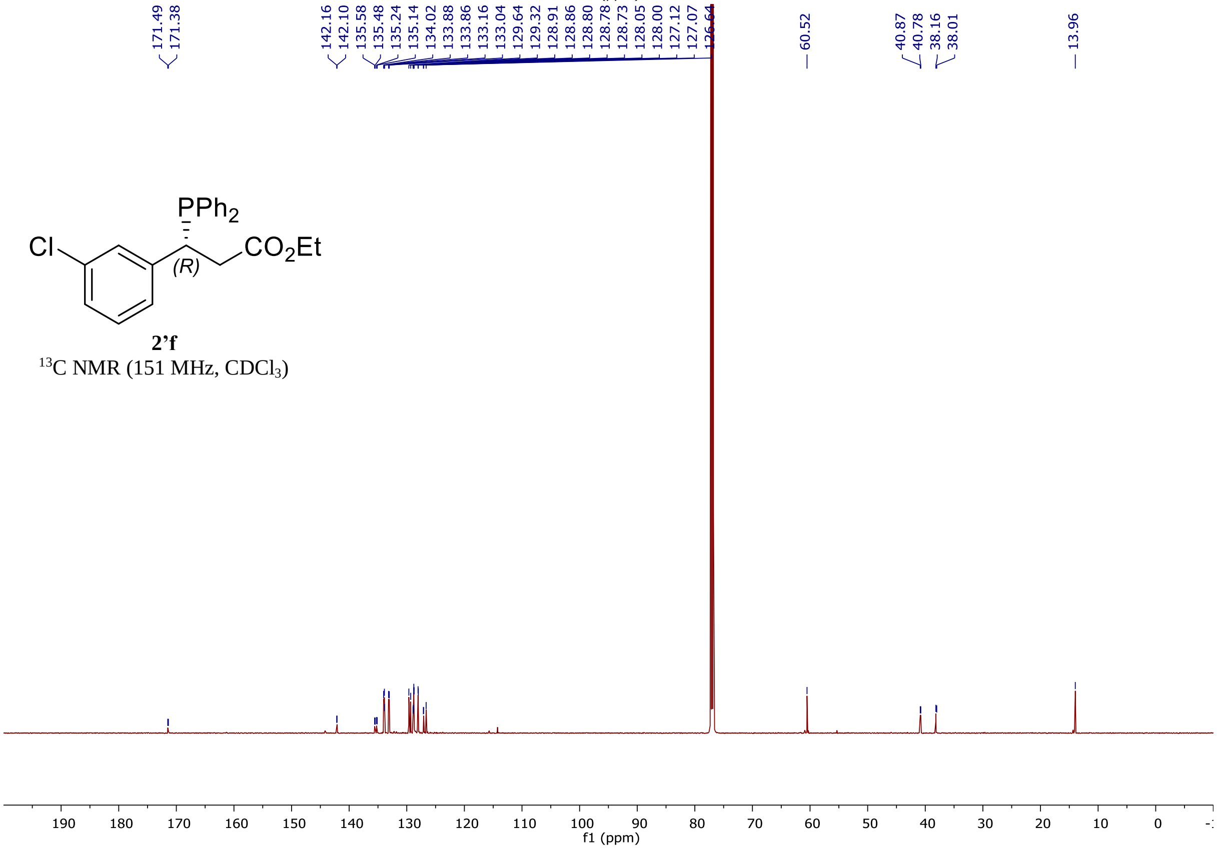

2'f

^{13}C NMR (151 MHz, $CDCl_3$)

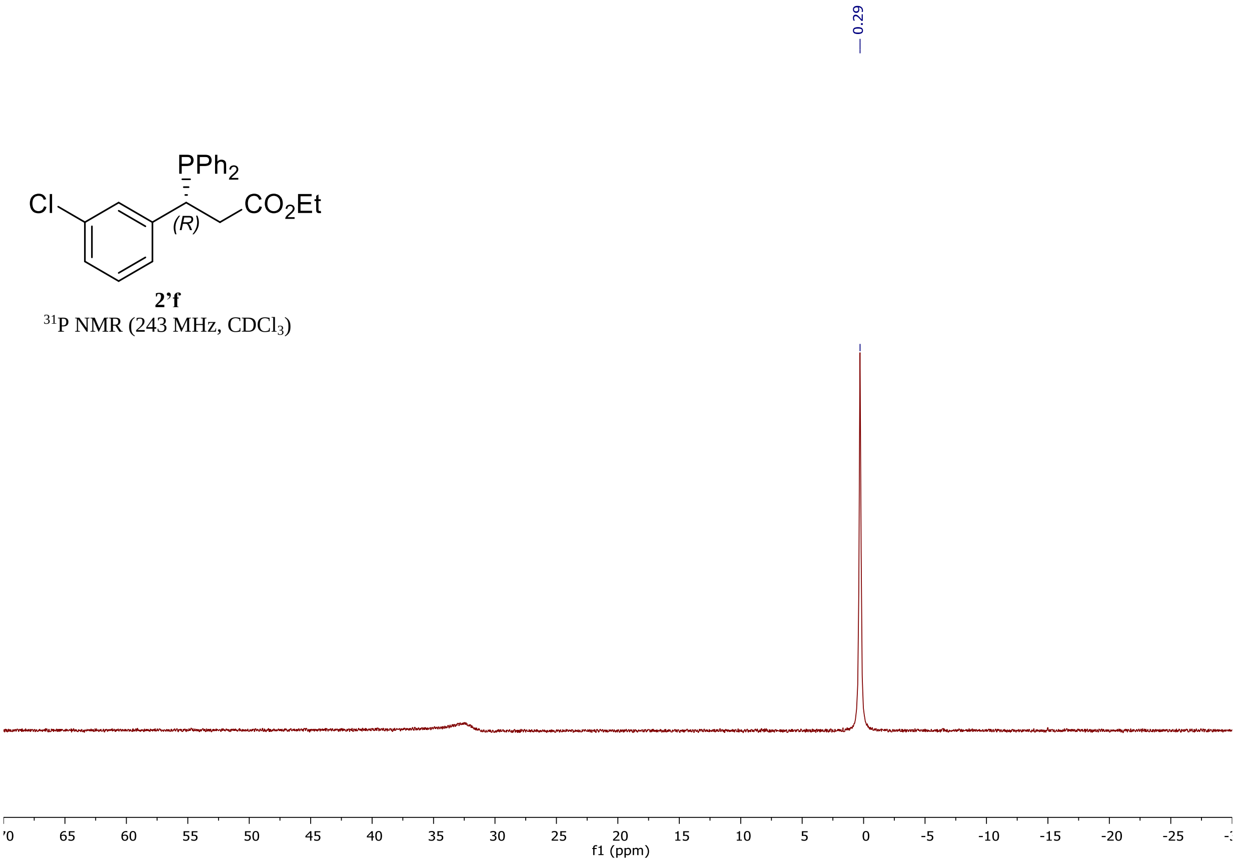

2'f

^{31}P NMR (243 MHz, $CDCl_3$)

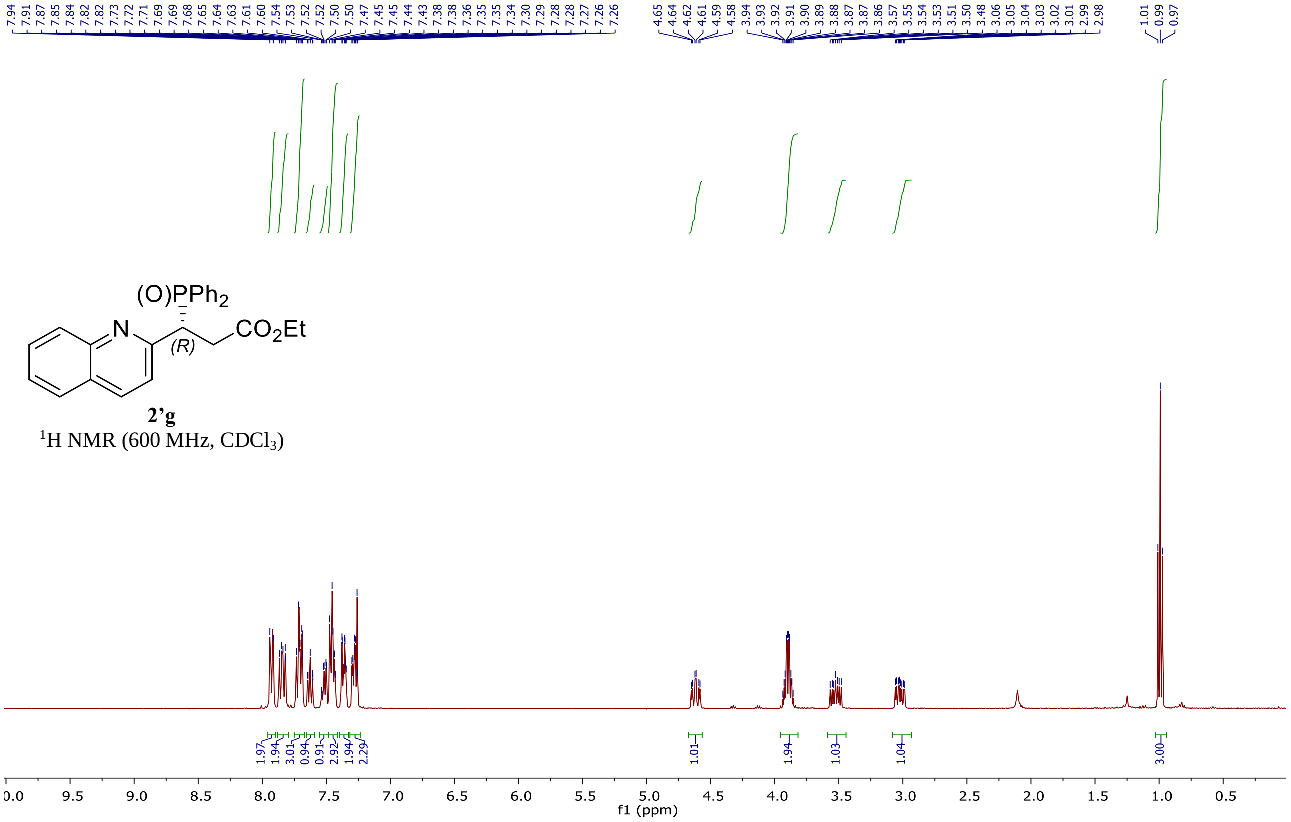

2'g

^{1}H NMR (600 MHz, $CDCl_3$)

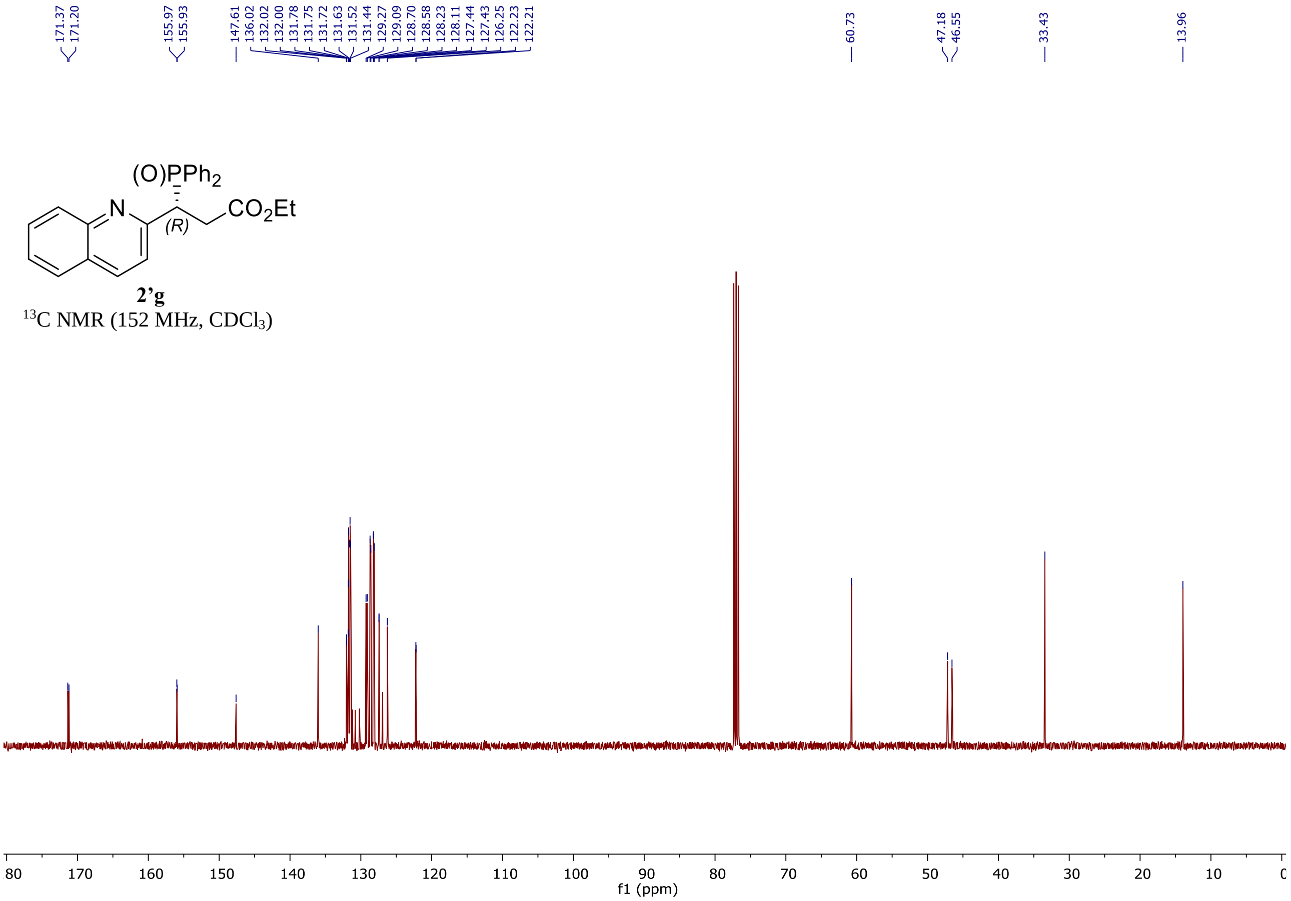

2'g

^{13}C NMR (152 MHz, $CDCl_3$)

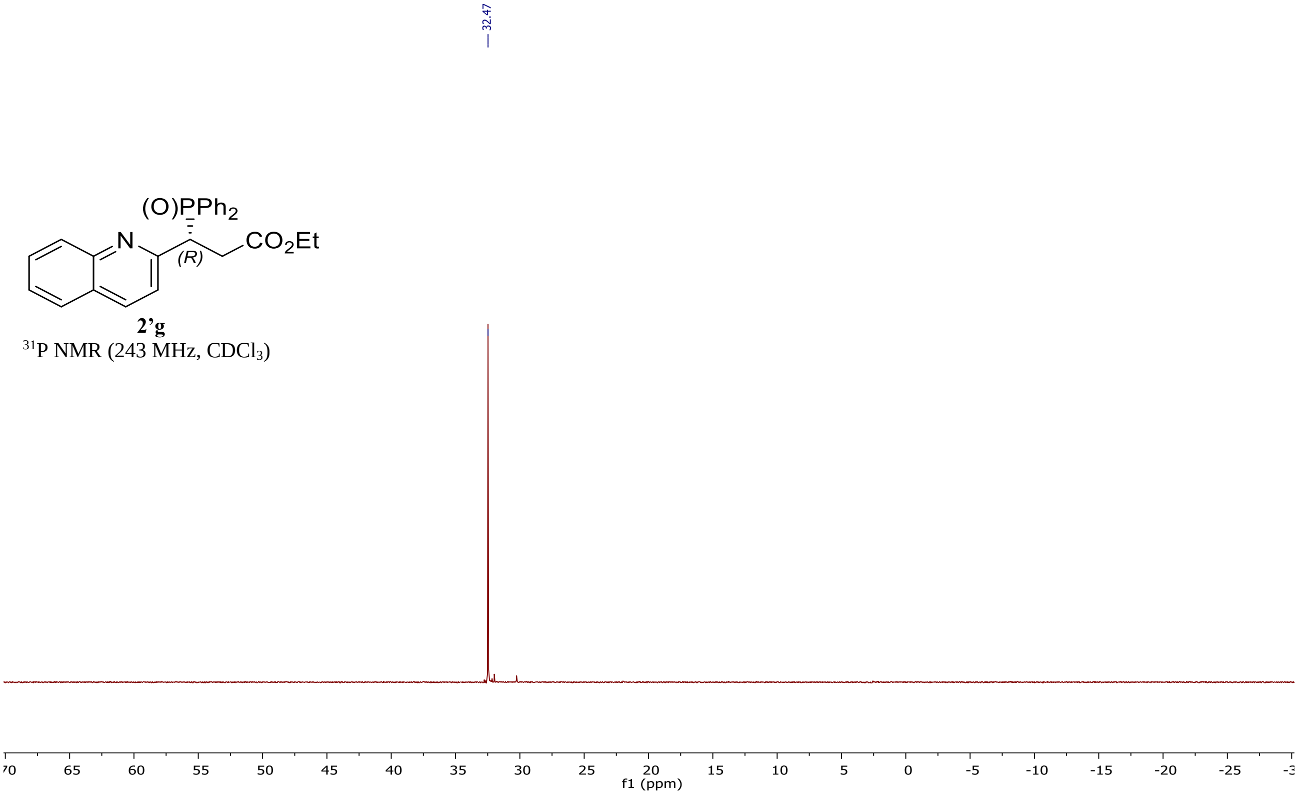

2'g

^{31}P NMR (243 MHz, $CDCl_3$)

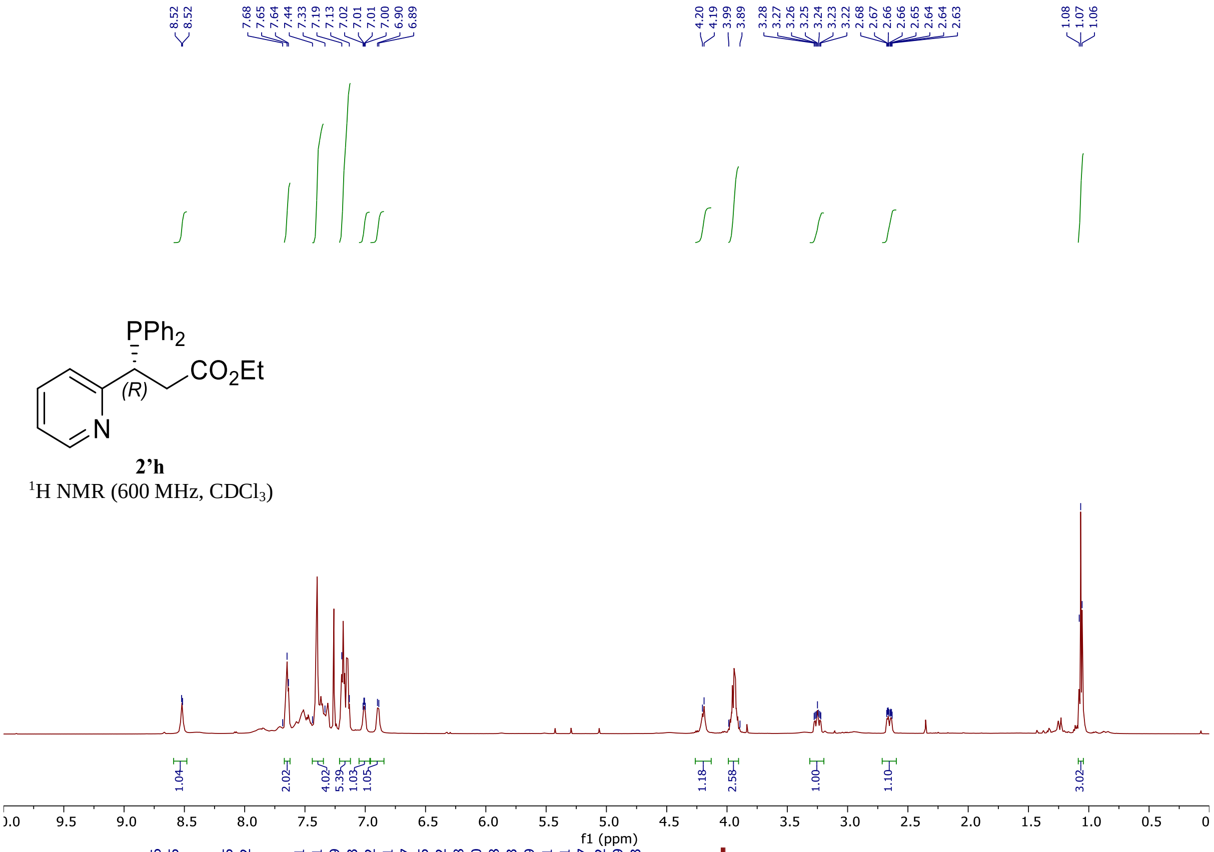

2'h

^{1}H NMR (600 MHz, $CDCl_3$)

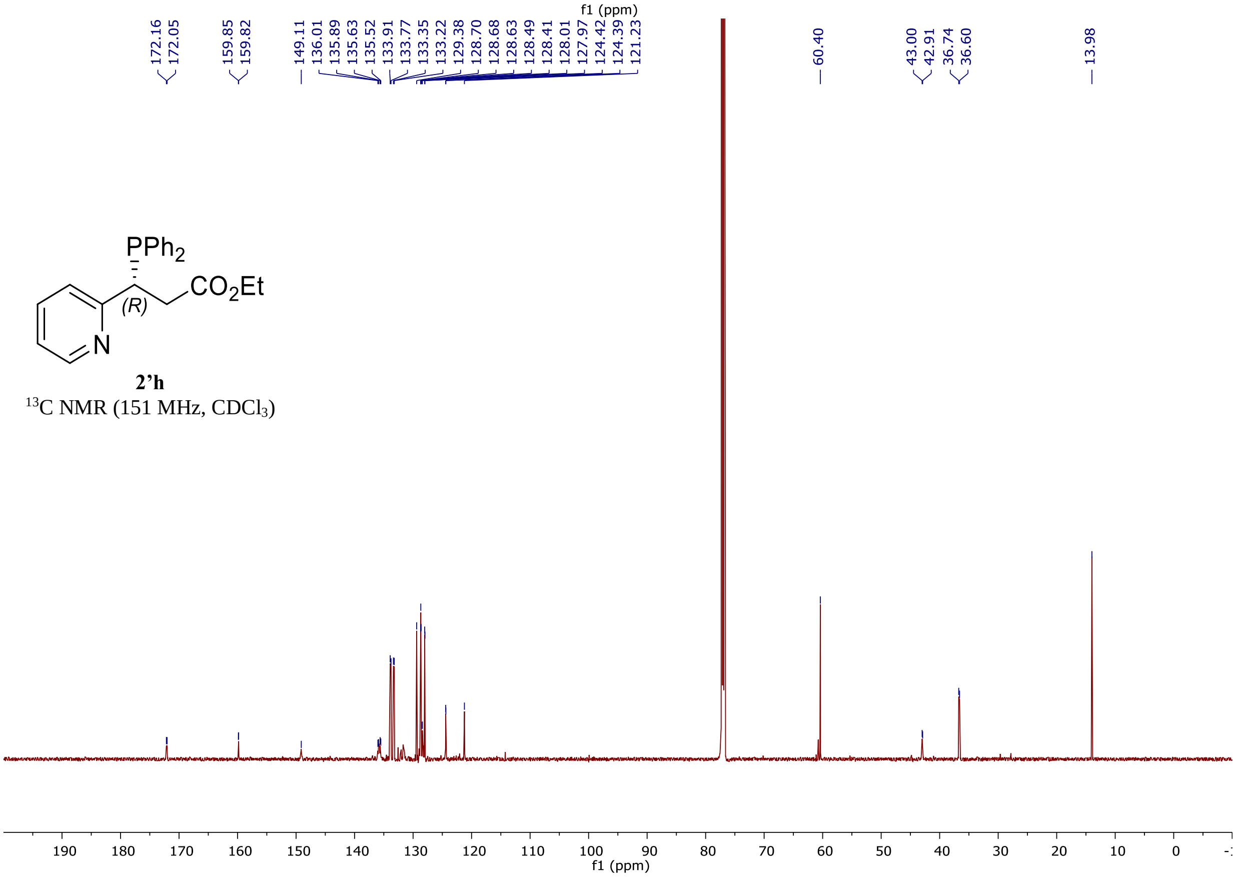

2'h

^{13}C NMR (151 MHz, $CDCl_3$)

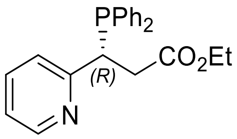

2’h

^{31}P NMR (243 MHz, $CDCl_3$)

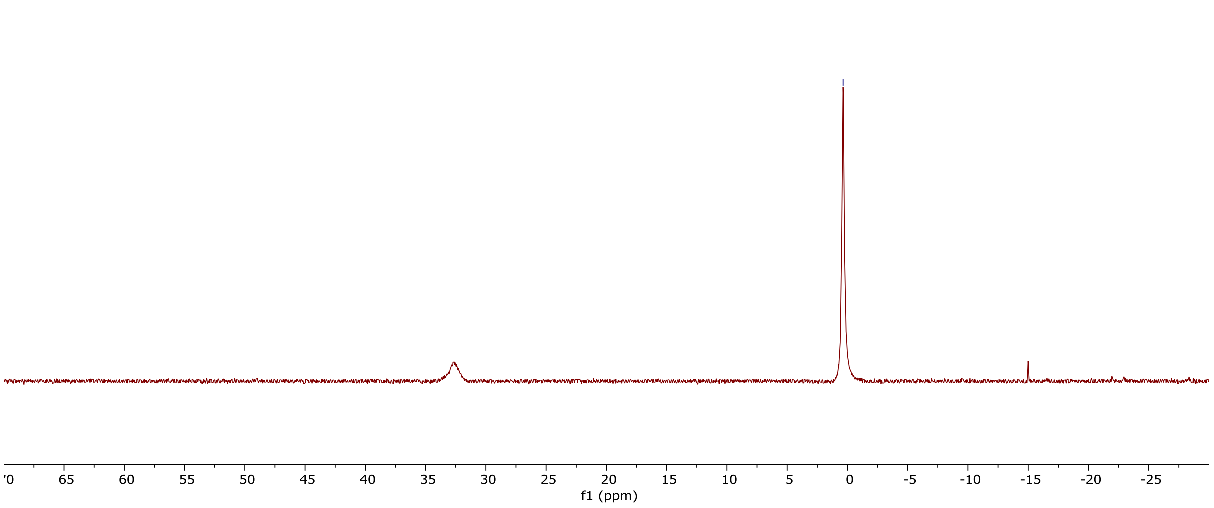

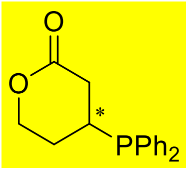

2’i

^{1}H NMR (600 MHz, $CDCl_3$)

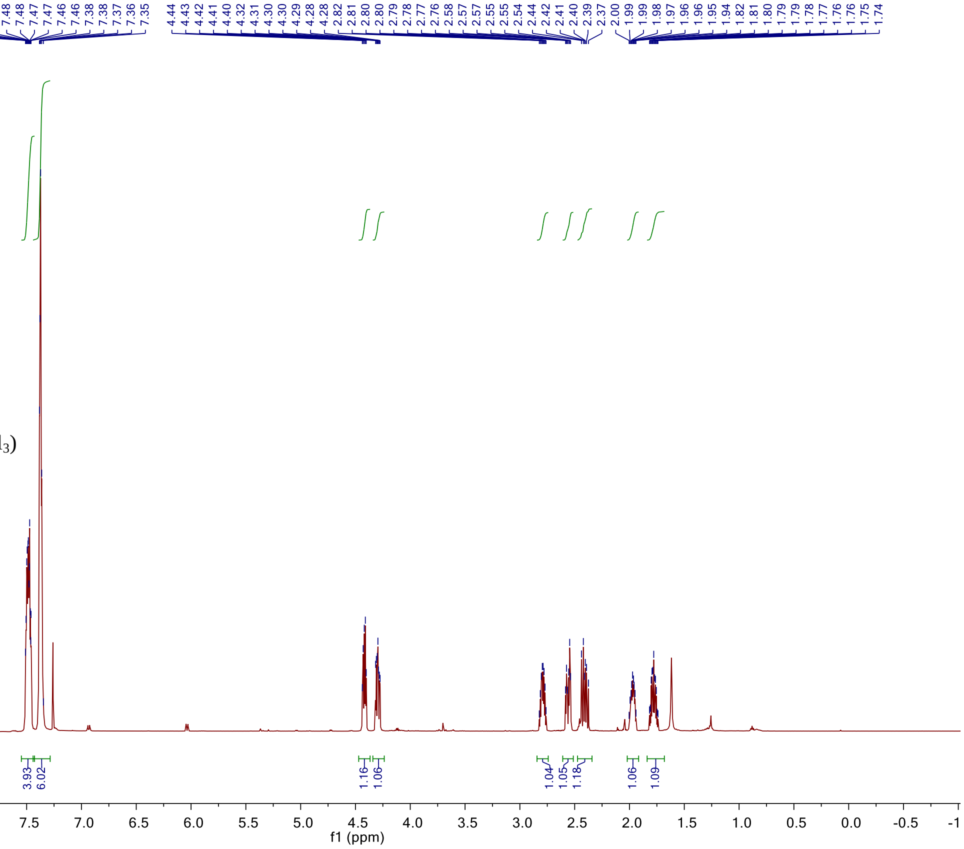

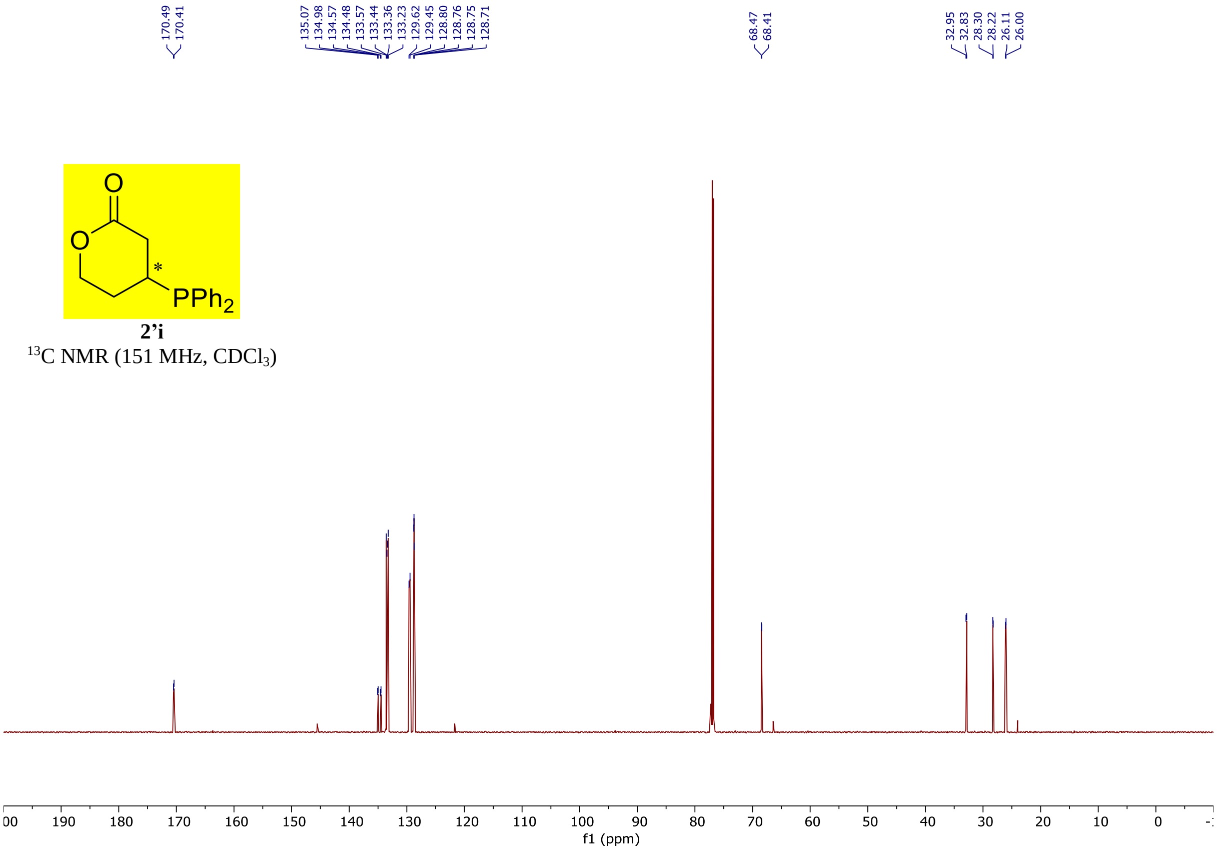

2'i
^{13}C NMR (151 MHz, $CDCl_3$)

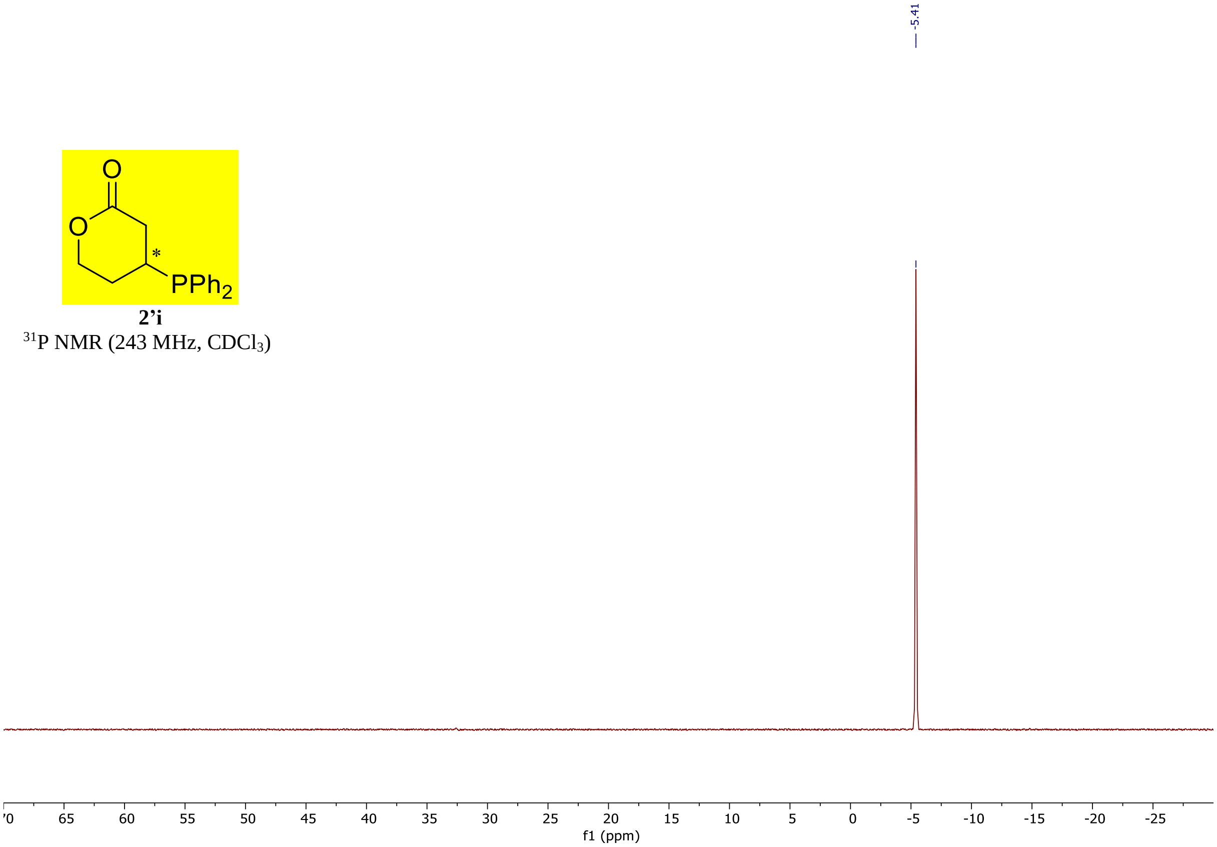

2'i
^{31}P NMR (243 MHz, $CDCl_3$)

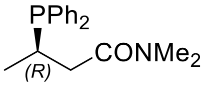

3’a

^{1}H NMR (600 MHz, $CDCl_3$)

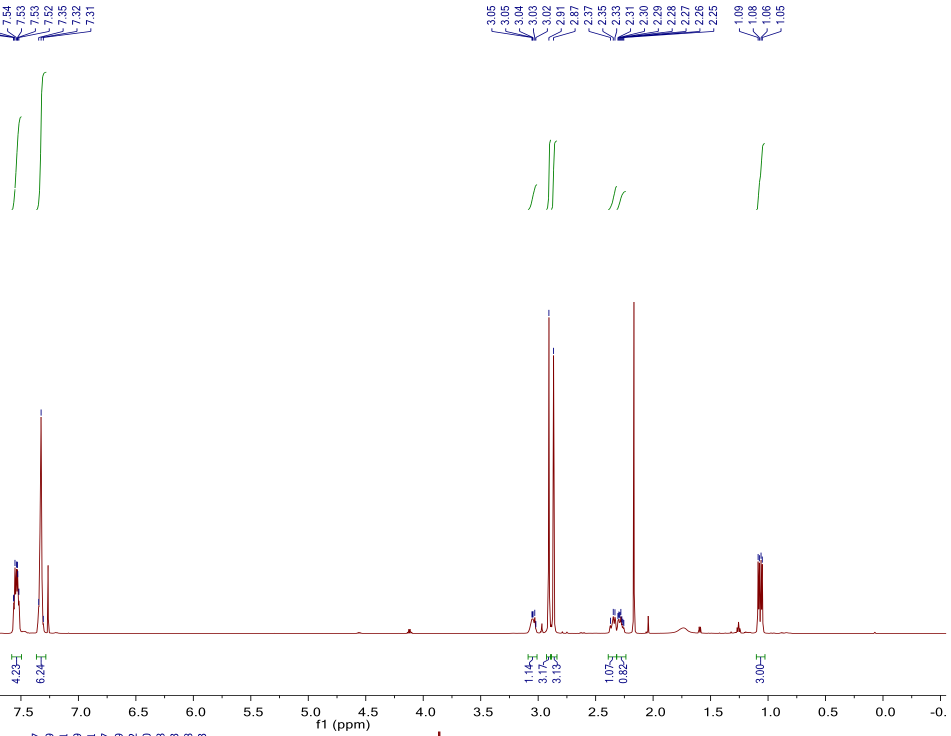

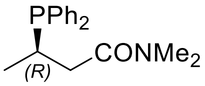

3’a

^{13}C NMR (151 MHz, $CDCl_3$)

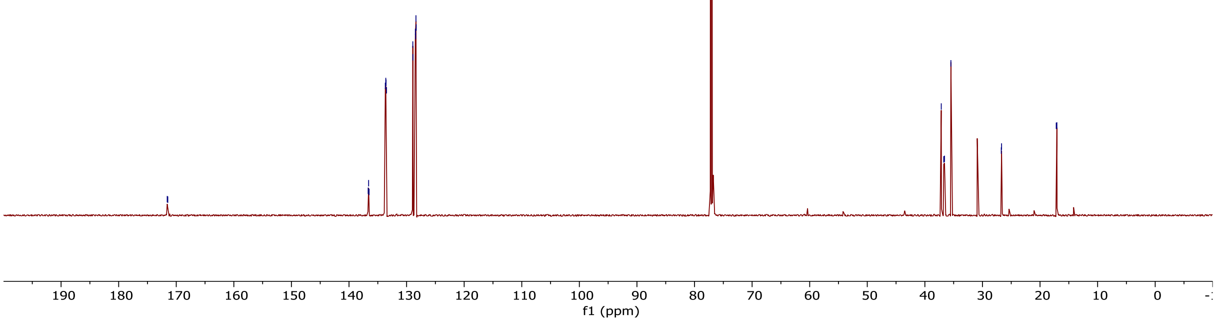

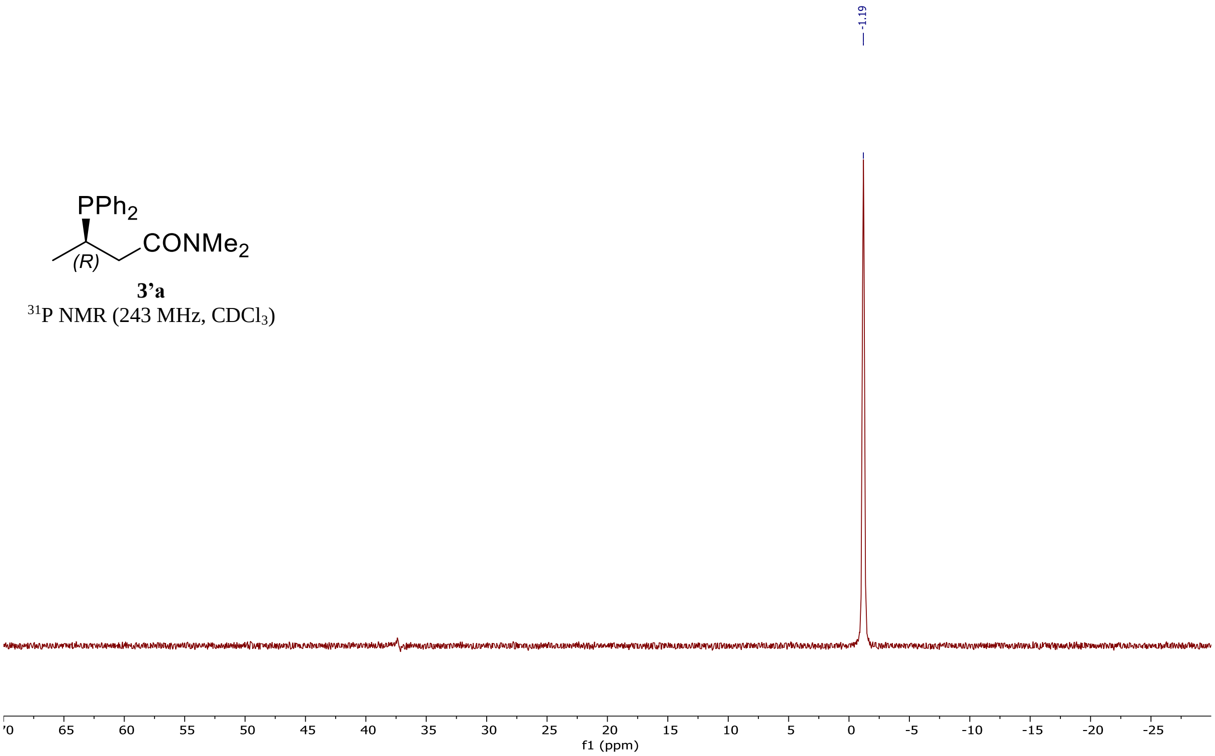

3'a

^{31}P NMR (243 MHz, $CDCl_3$)

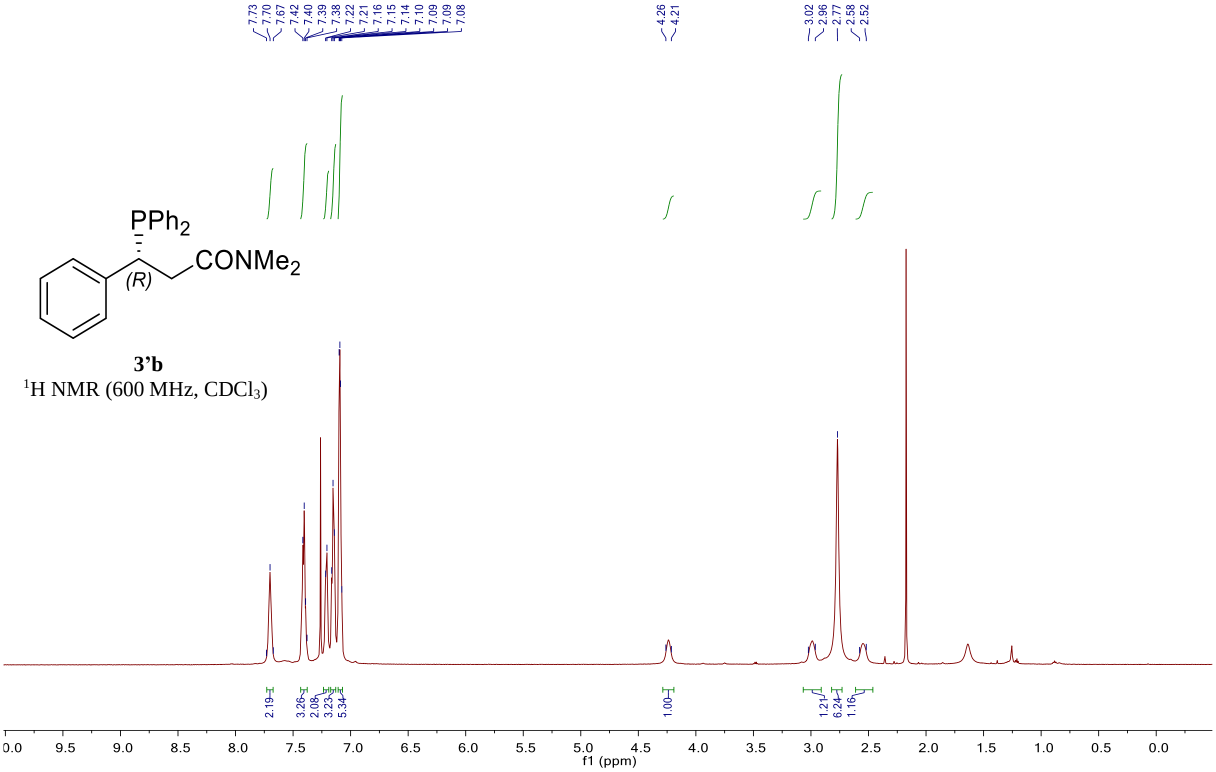

3'b

^{1}H NMR (600 MHz, $CDCl_3$)

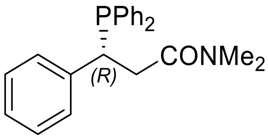

3'b
^{13}C NMR (151 MHz, $CDCl_3$)

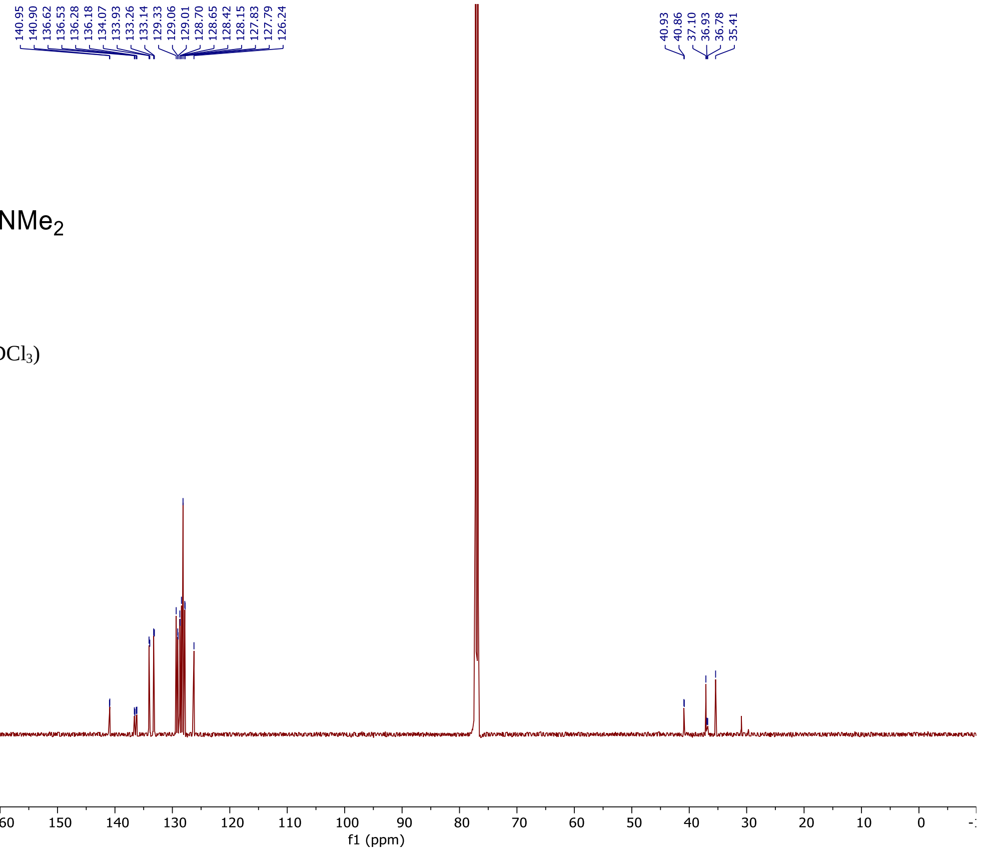

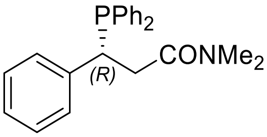

3'b
^{31}P NMR (243 MHz, $CDCl_3$)

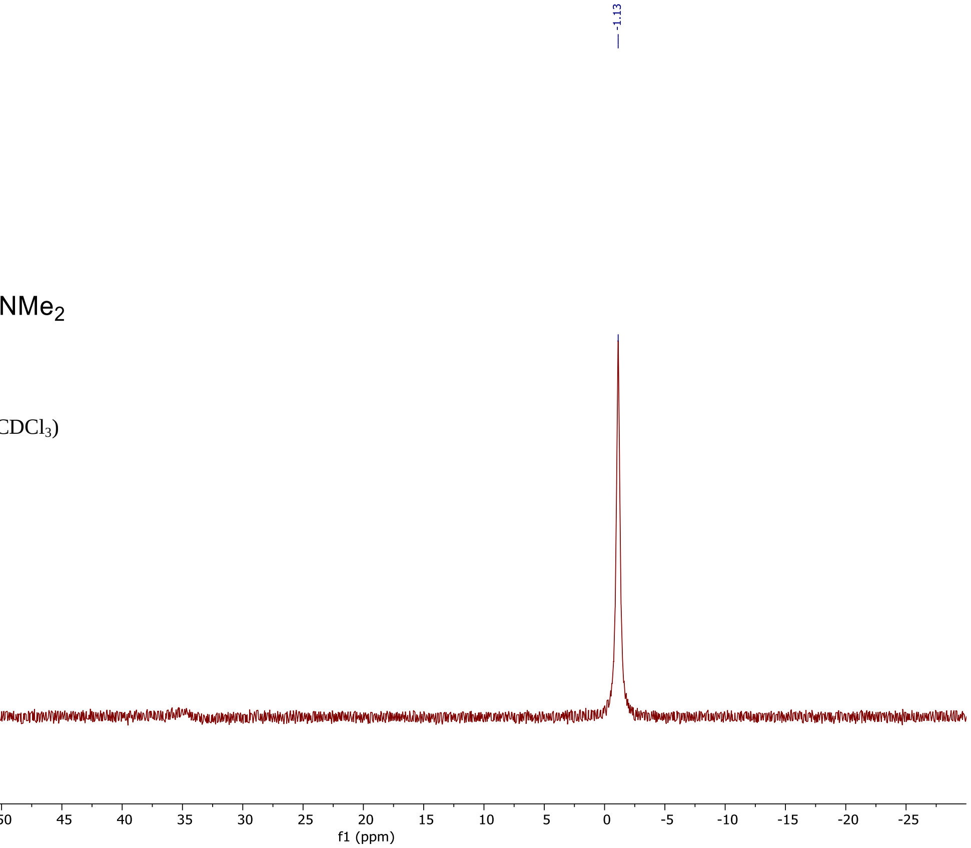

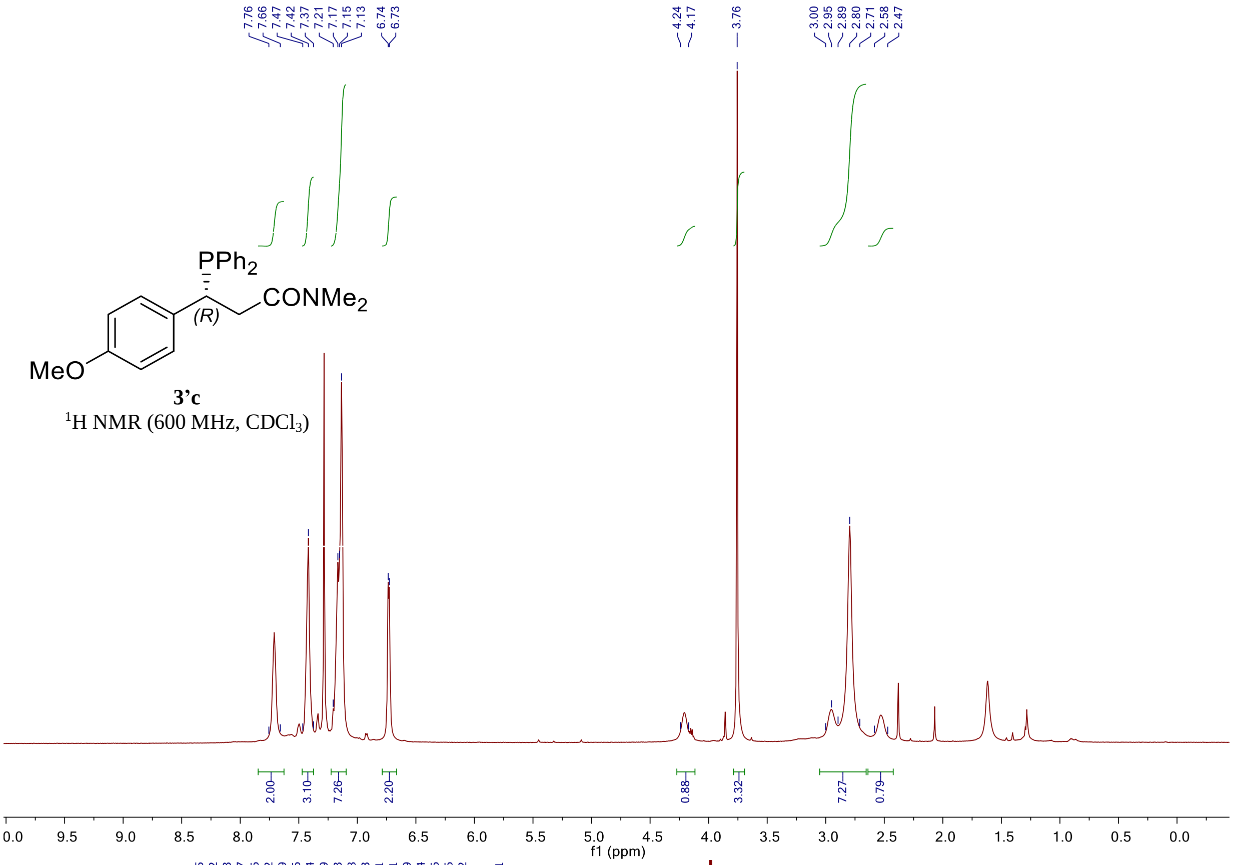

3’c

^{1}H NMR (600 MHz, $CDCl_3$)

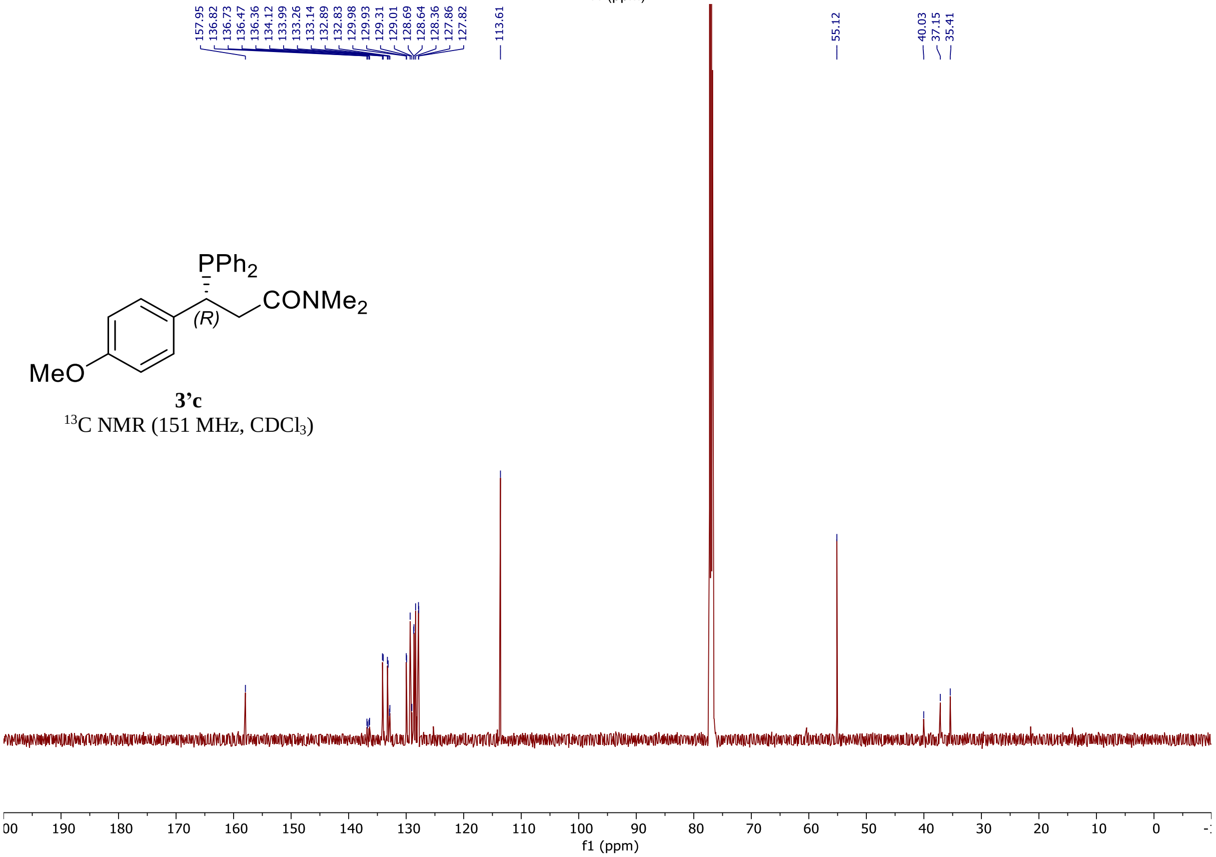

3’c

^{13}C NMR (151 MHz, $CDCl_3$)

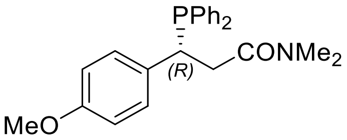

3’c

^{31}P NMR (243 MHz, $CDCl_3$)

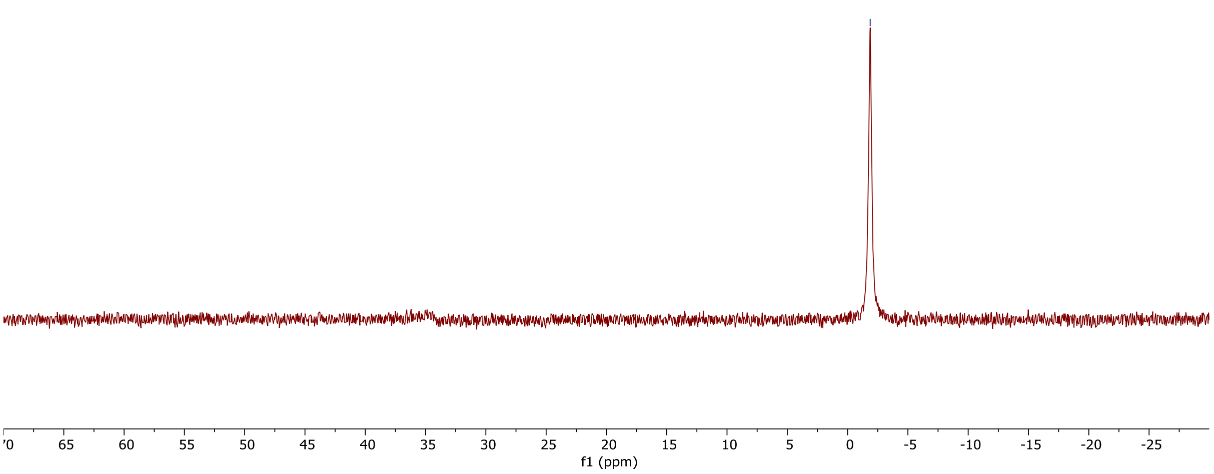

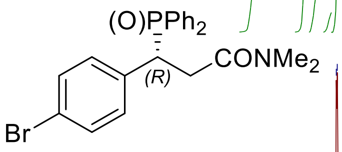

3’d

^{1}H NMR (400 MHz, $CDCl_3$)

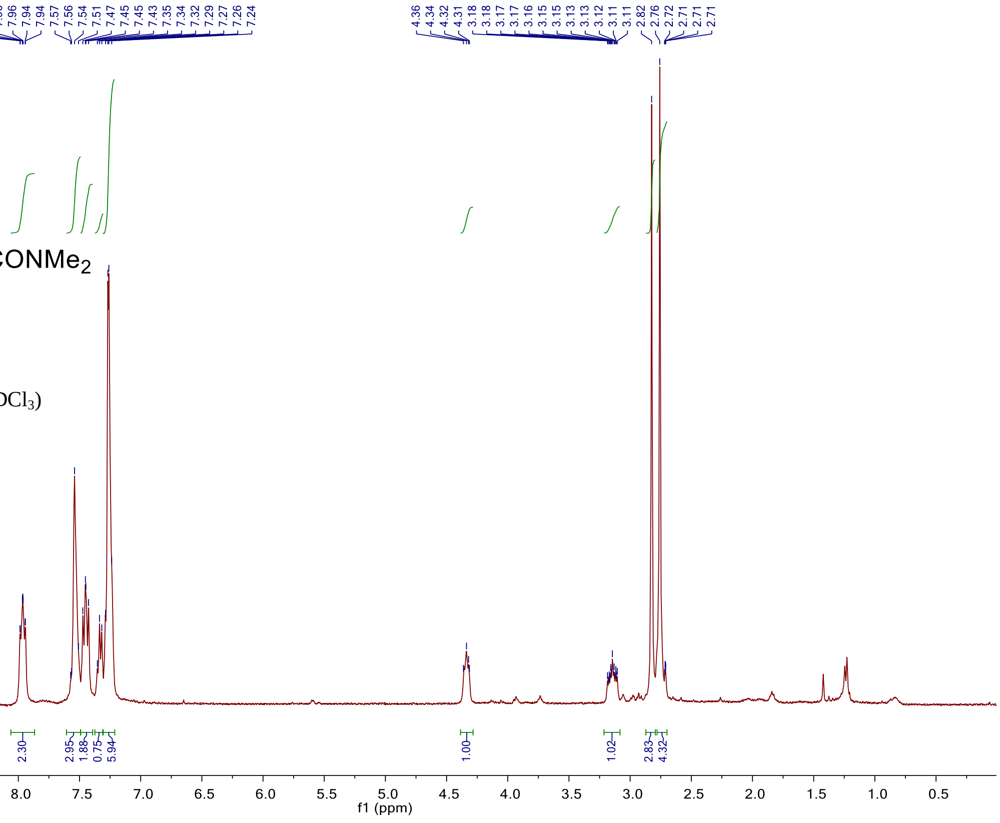

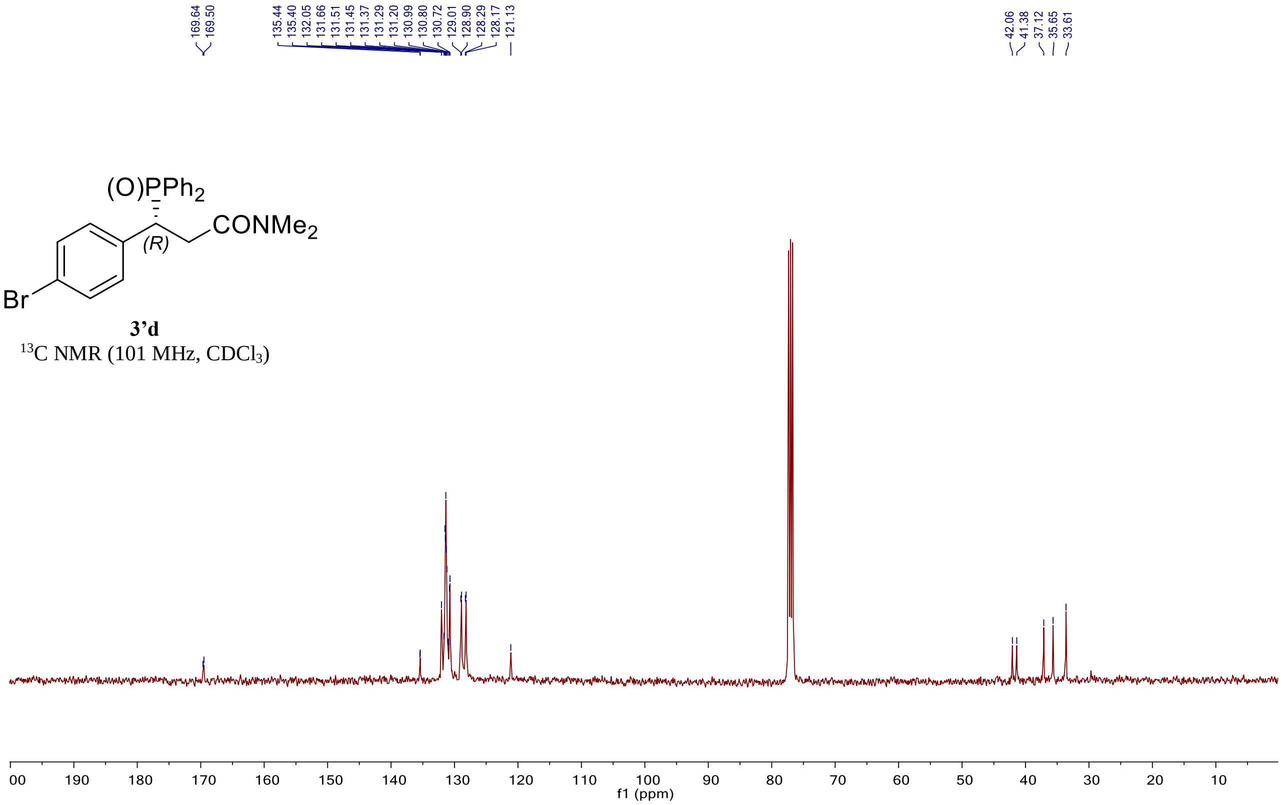

3’d

^{13}C NMR (101 MHz, $CDCl_3$)

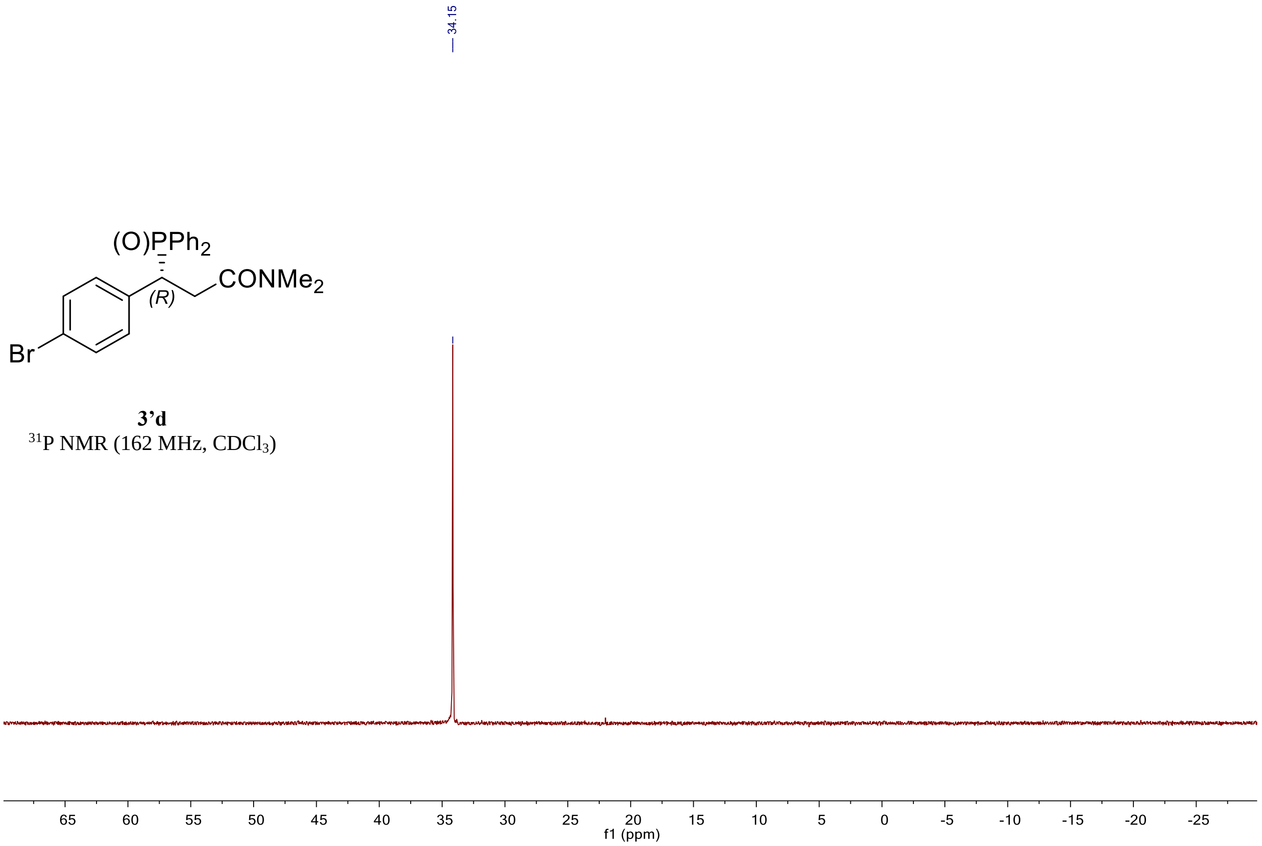

3’d
^{31}P NMR (162 MHz, CDCl$_3$)

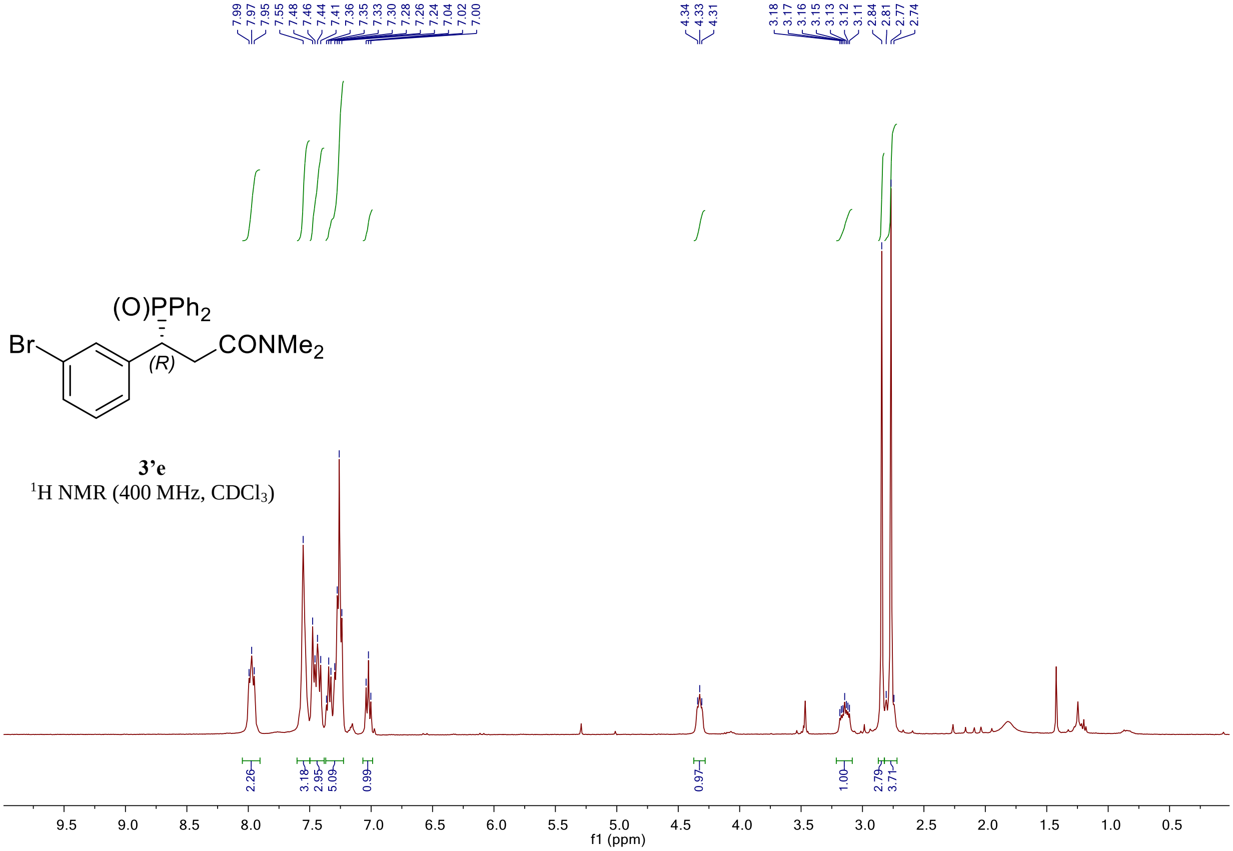

3’e
^{1}H NMR (400 MHz, CDCl$_3$)

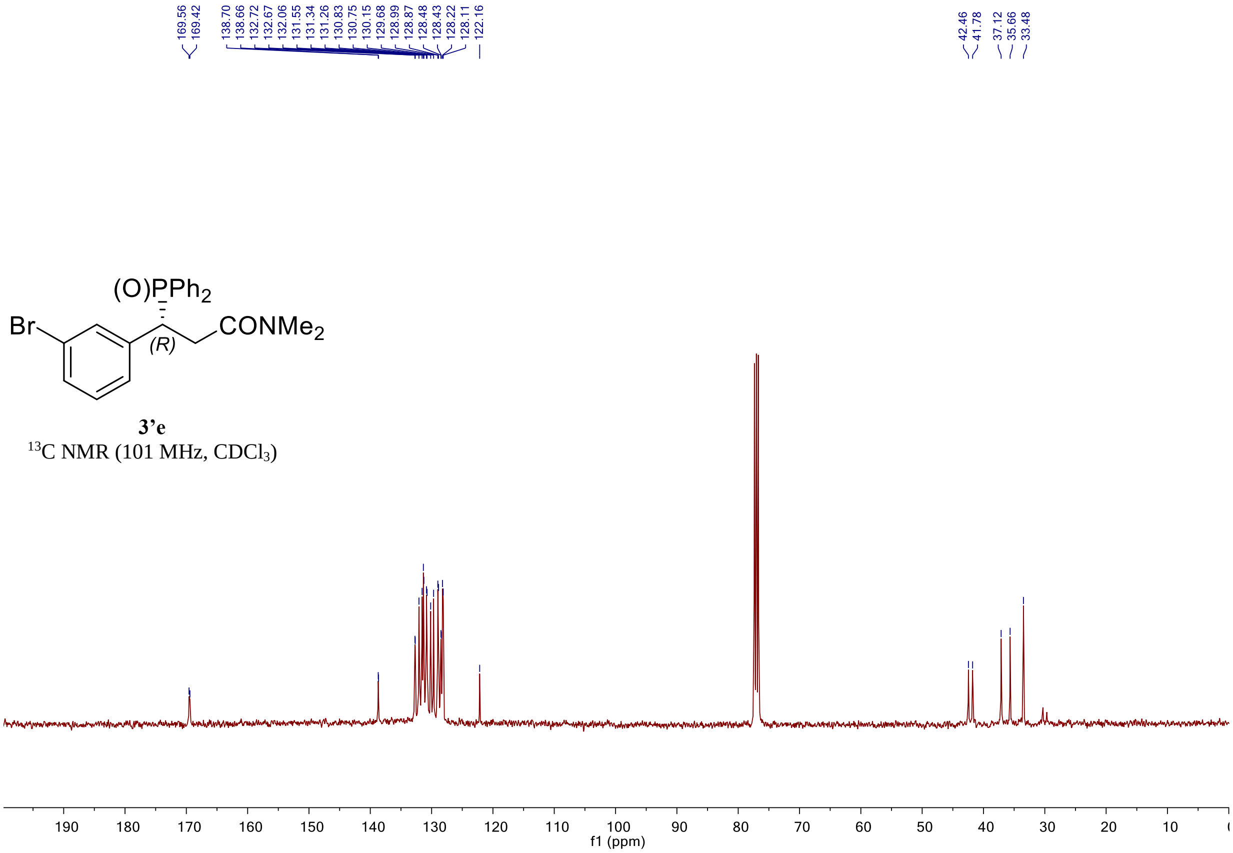

3'e

^{13}C NMR (101 MHz, $CDCl_3$)

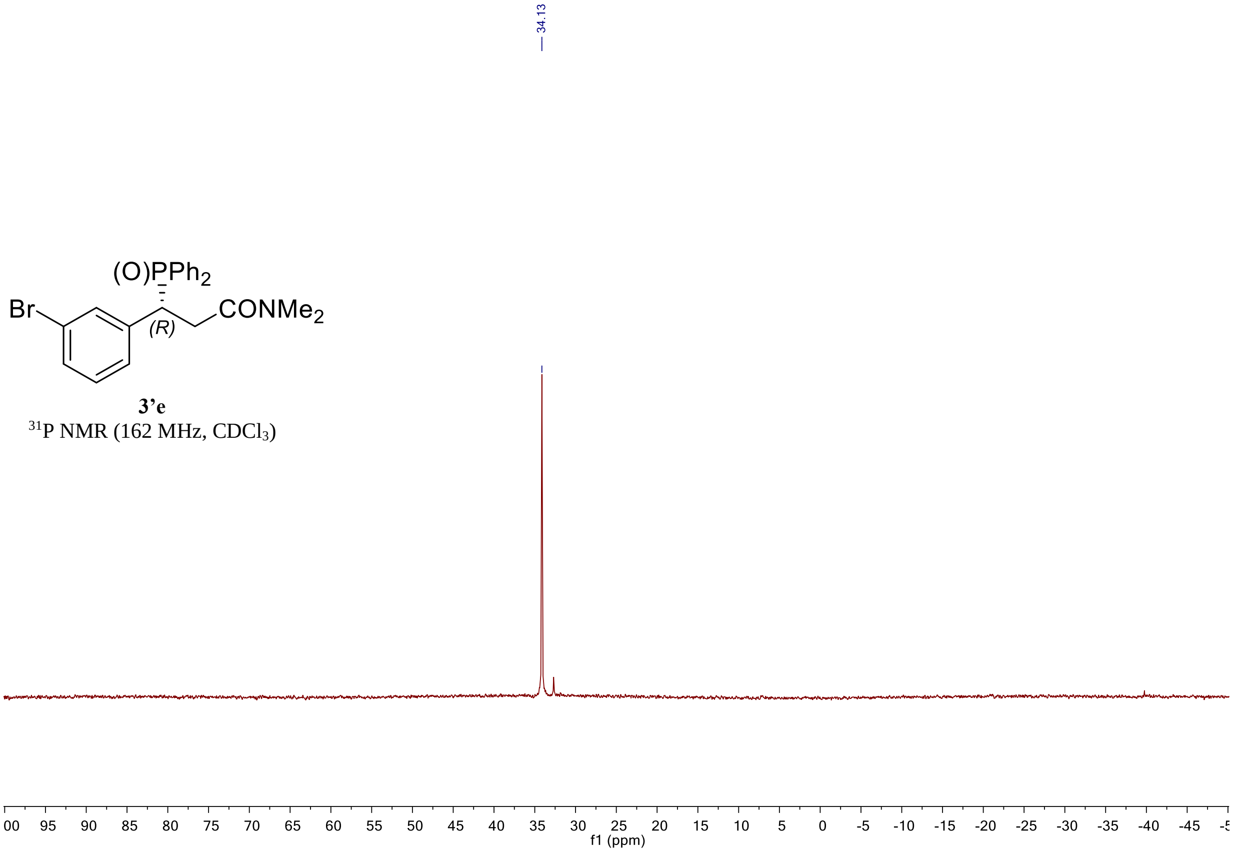

3'e

^{31}P NMR (162 MHz, $CDCl_3$)

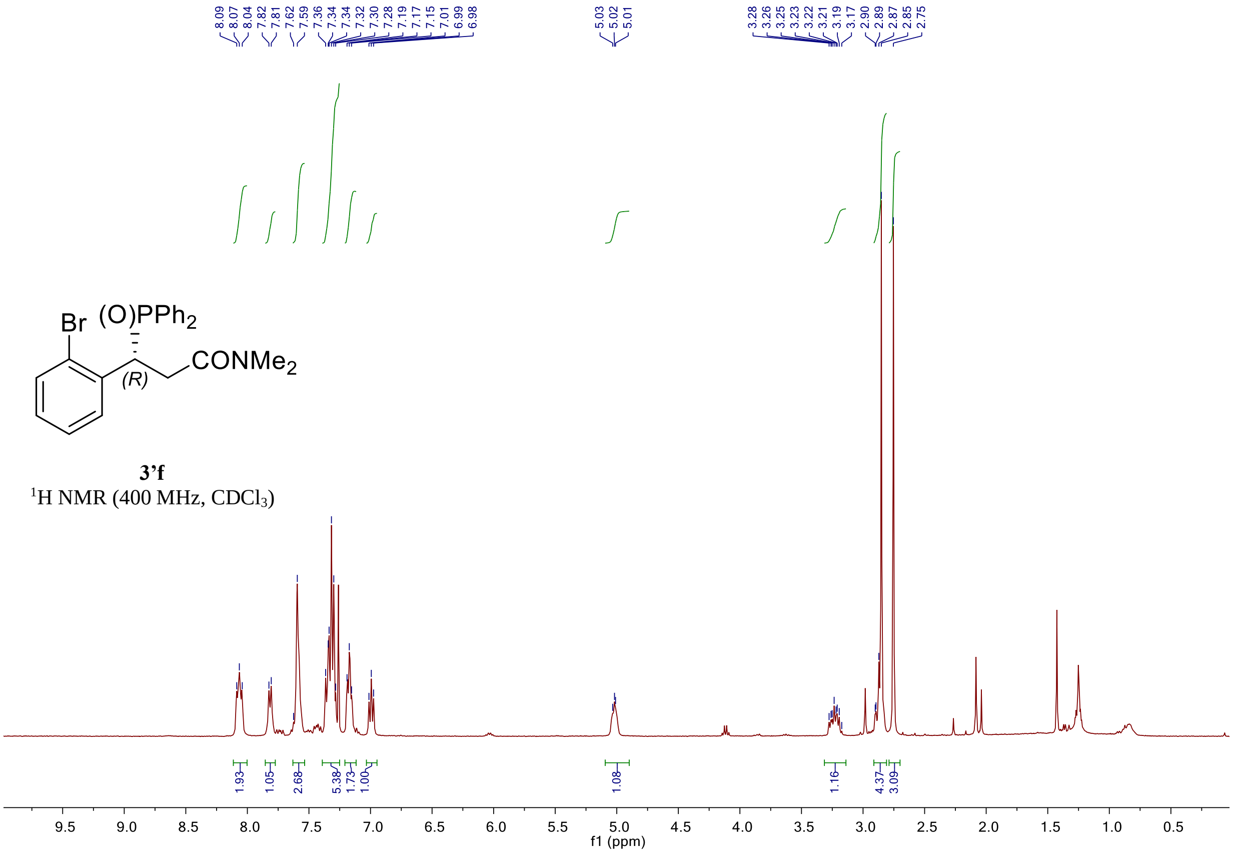

3’f
^{1}H NMR (400 MHz, $CDCl_3$)

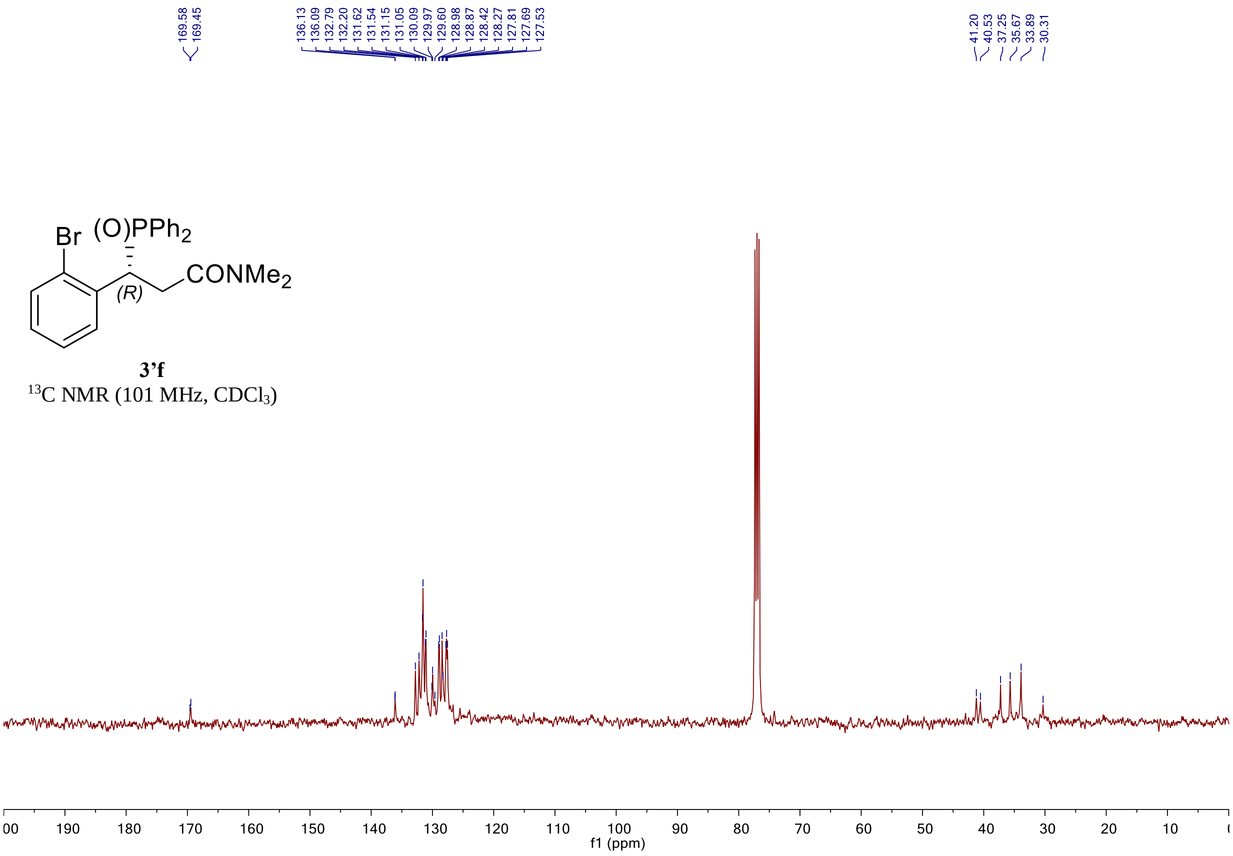

3’f
^{13}C NMR (101 MHz, $CDCl_3$)

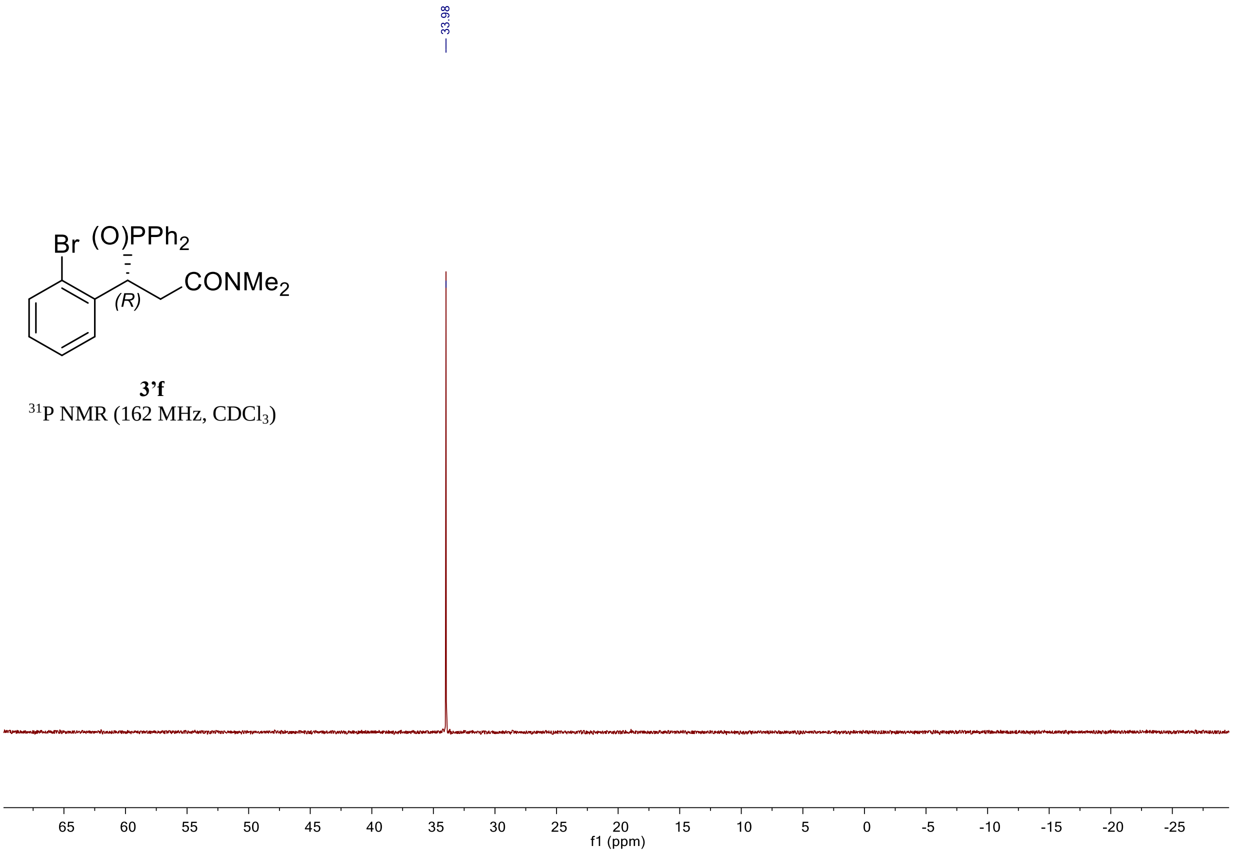

3’f

^{31}P NMR (162 MHz, $CDCl_3$)

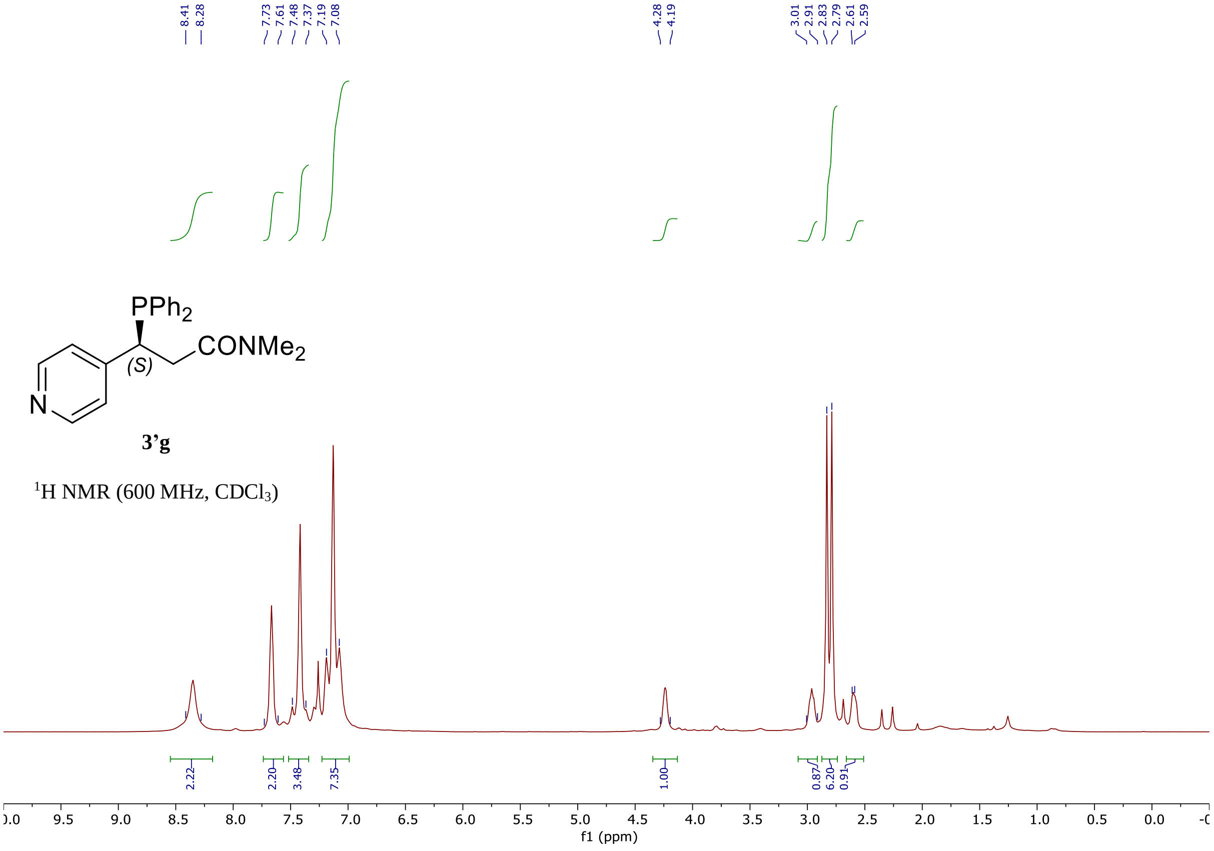

3’g

^{1}H NMR (600 MHz, $CDCl_3$)

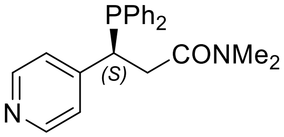

3’g

^{13}C NMR (151 MHz, $CDCl_3$)

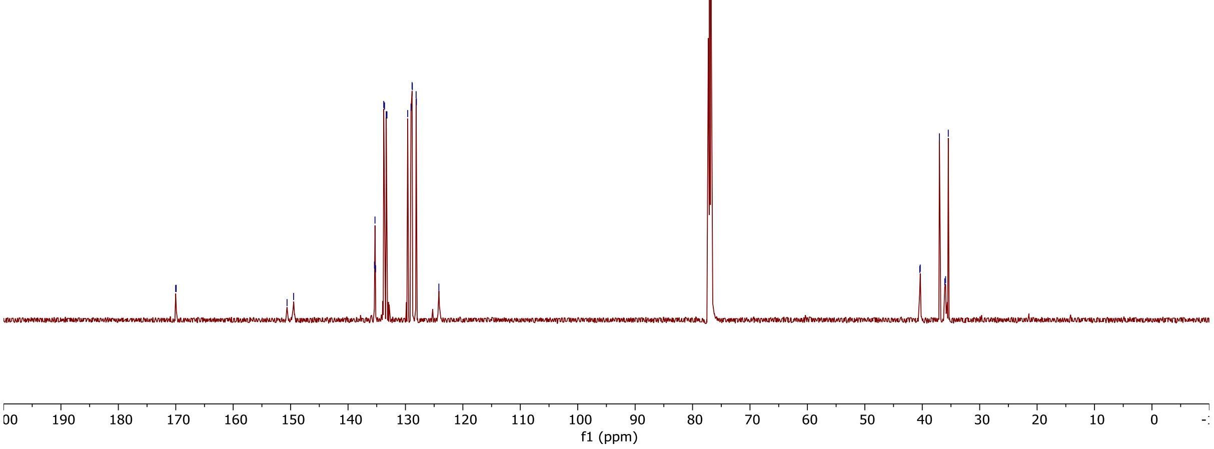

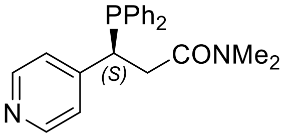

3’g

^{31}P NMR (243 MHz, $CDCl_3$)

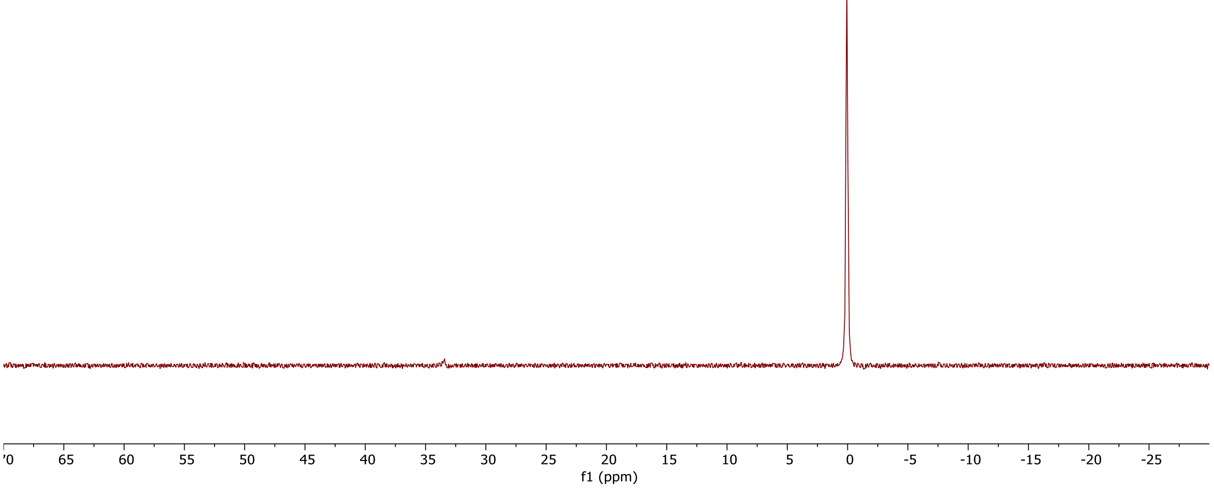

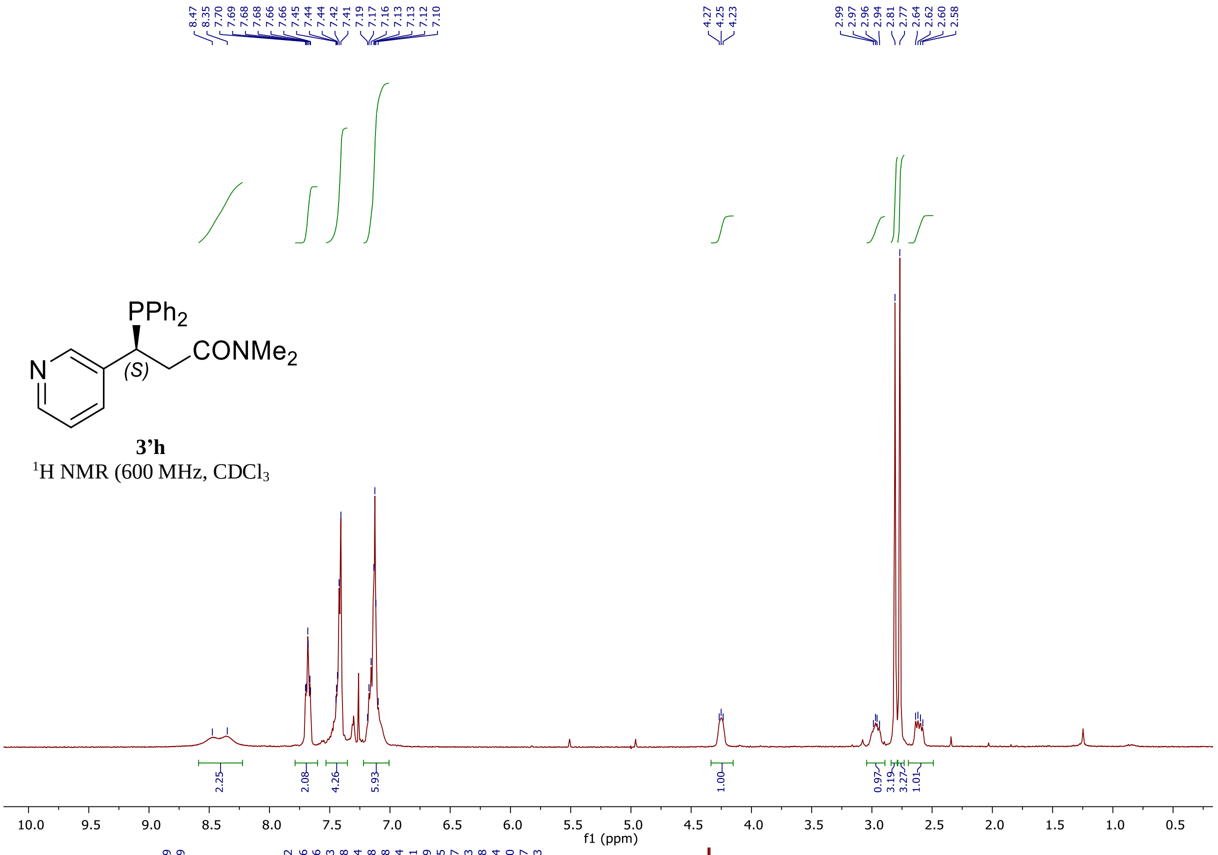

3'h
^{1}H NMR (600 MHz, $CDCl_3$

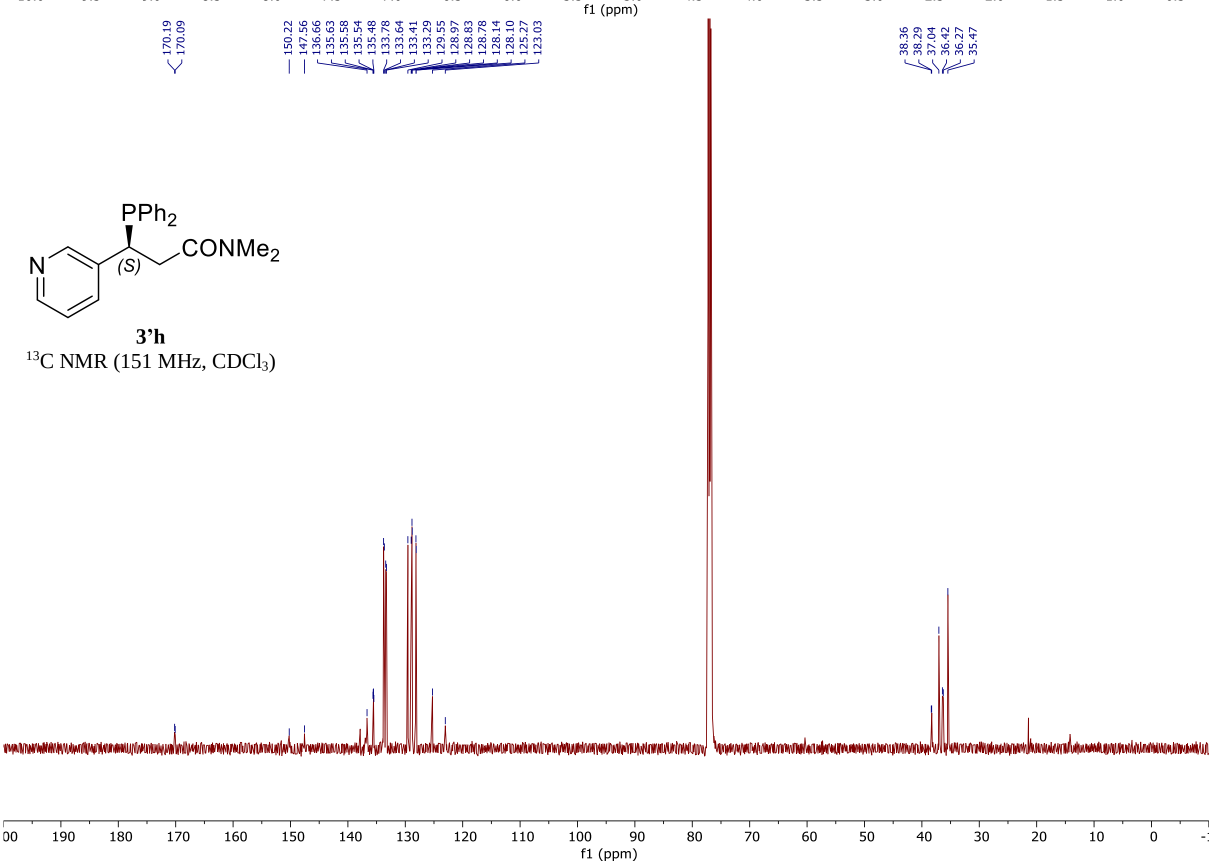

3'h
^{13}C NMR (151 MHz, $CDCl_3$)

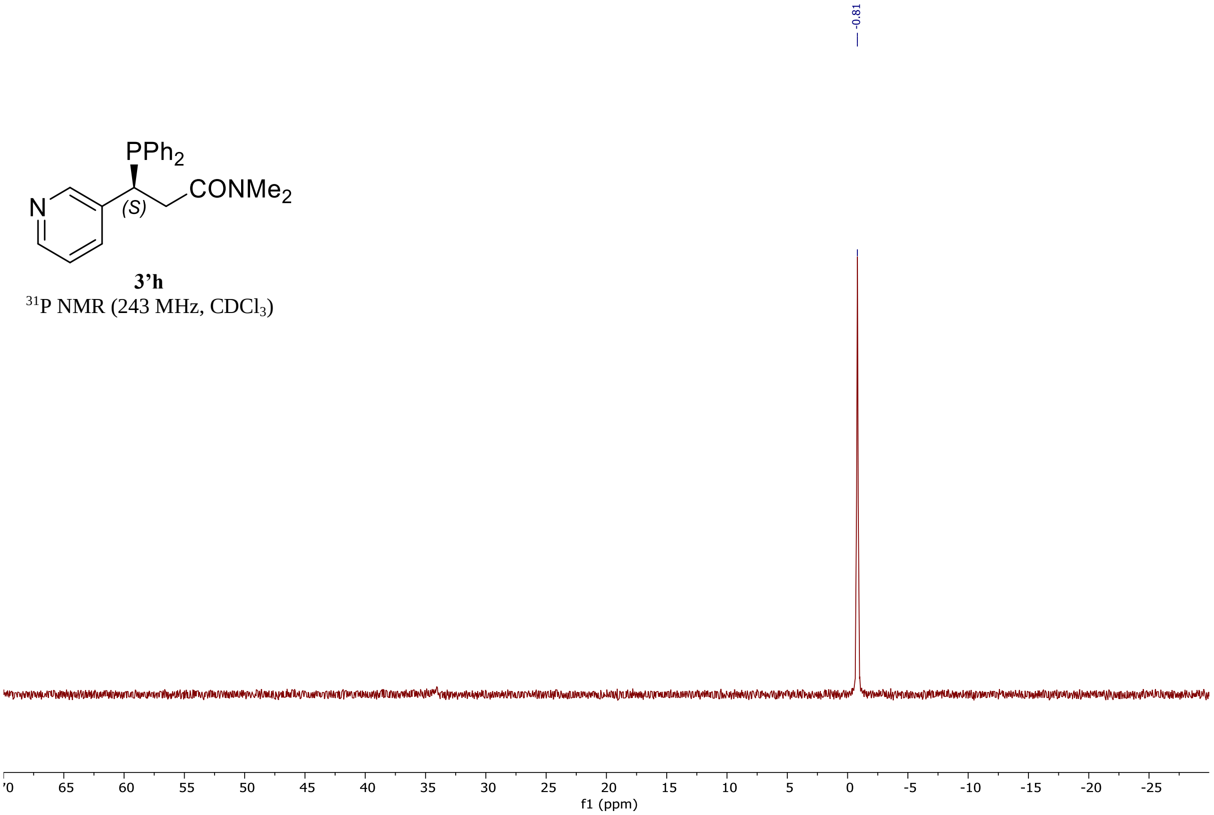

3’h

^{31}P NMR (243 MHz, $CDCl_3$)

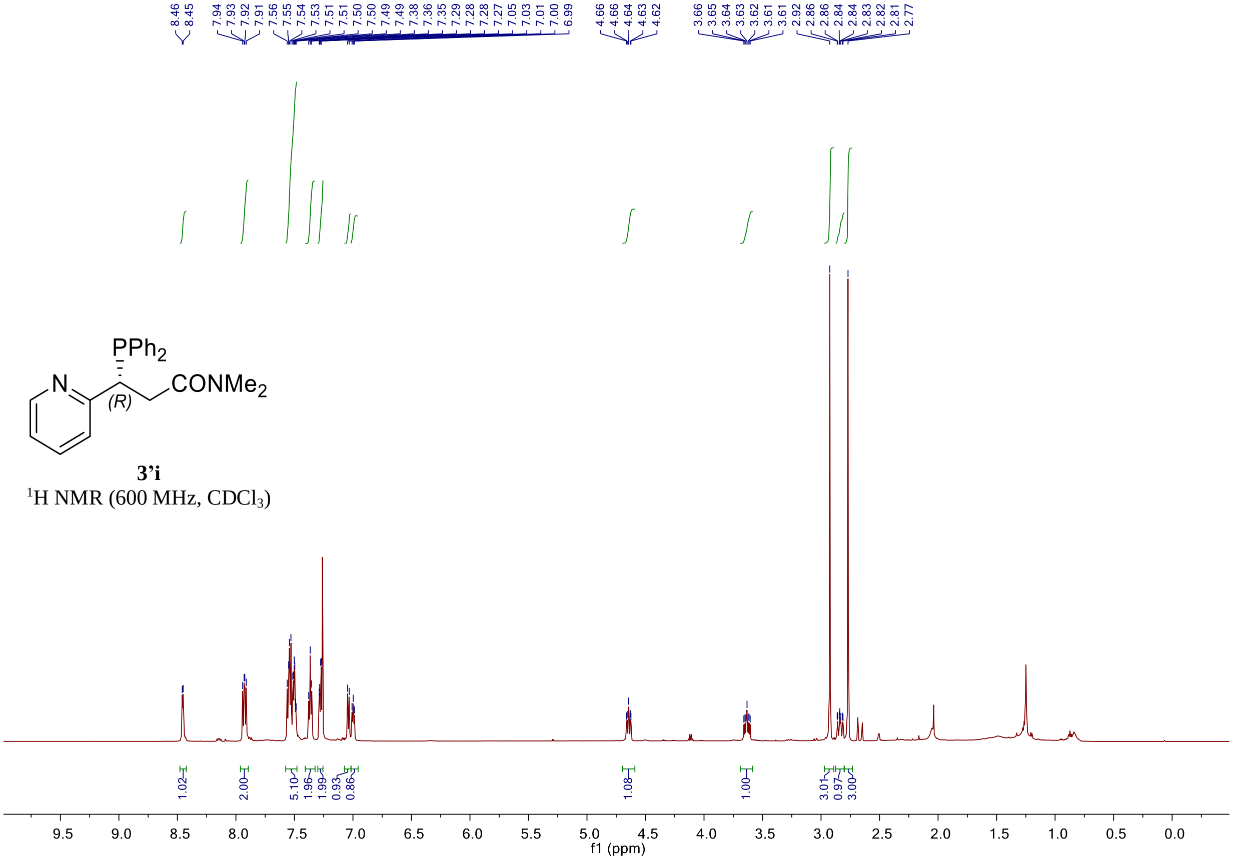

3’i

^{1}H NMR (600 MHz, $CDCl_3$)

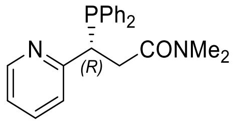

3'i
^{13}C NMR (151 MHz, $CDCl_3$)

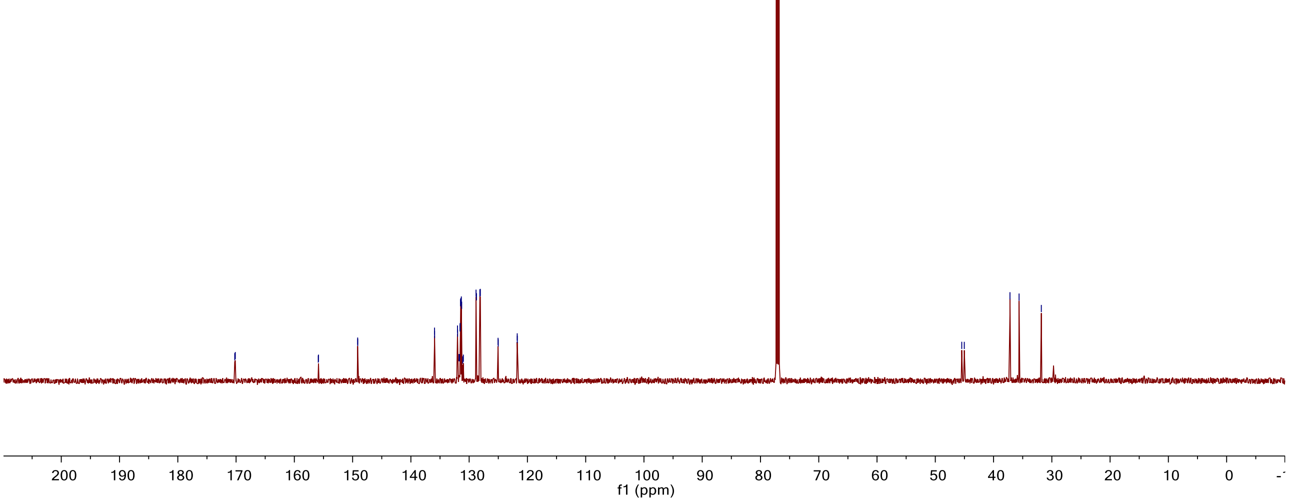

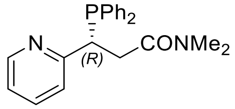

3'i
^{31}P NMR (243 MHz, $CDCl_3$)

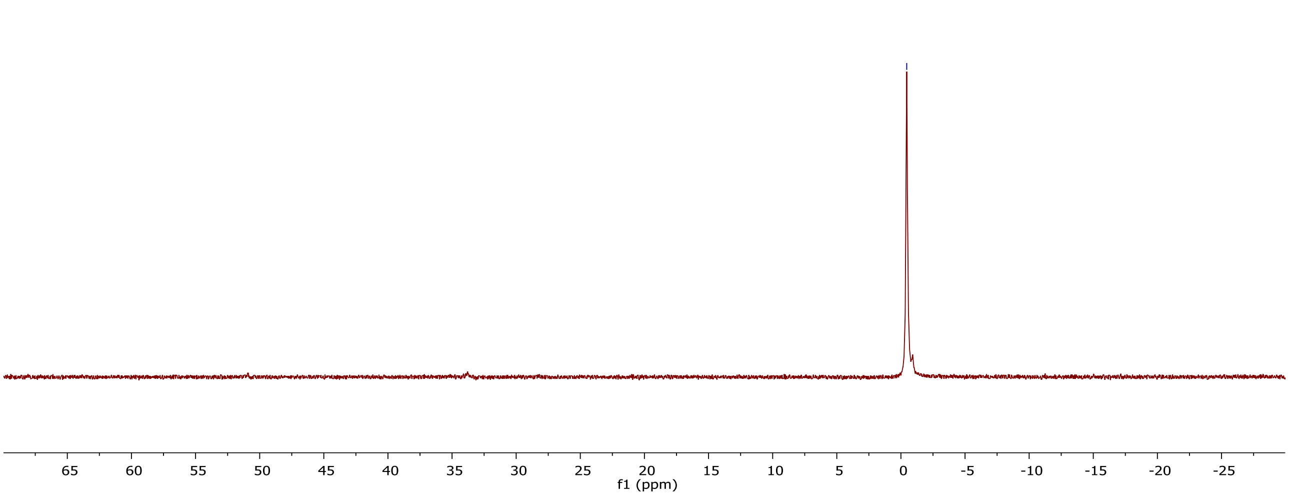

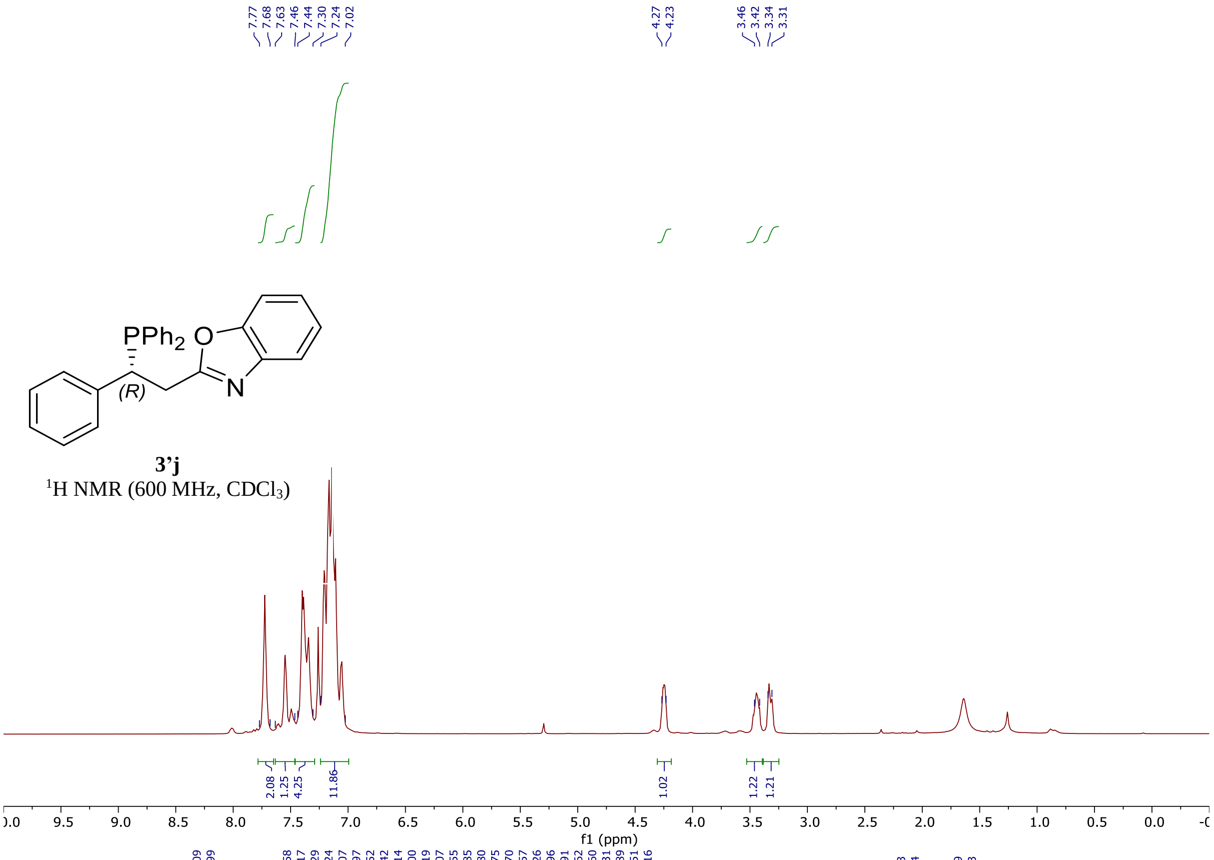

3'j

^{1}H NMR (600 MHz, $CDCl_3$)

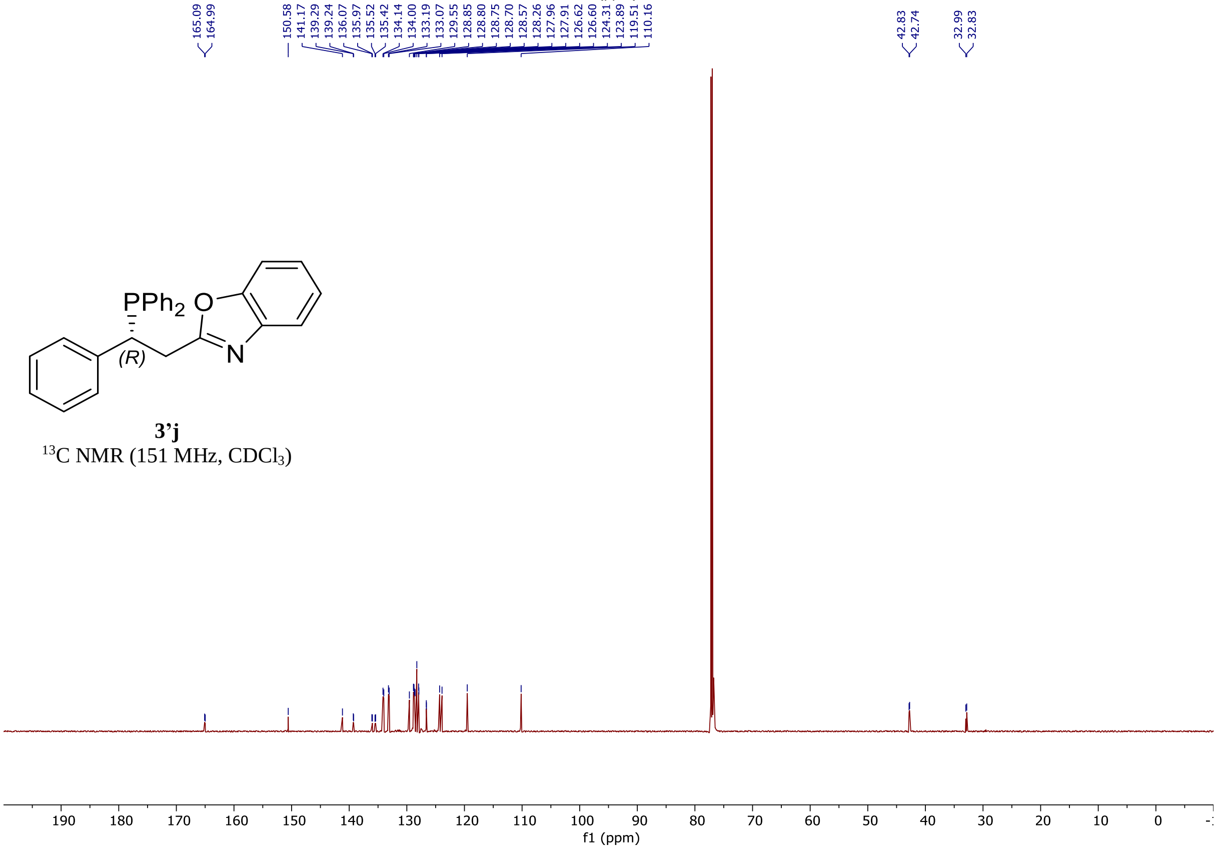

3'j

^{13}C NMR (151 MHz, $CDCl_3$)

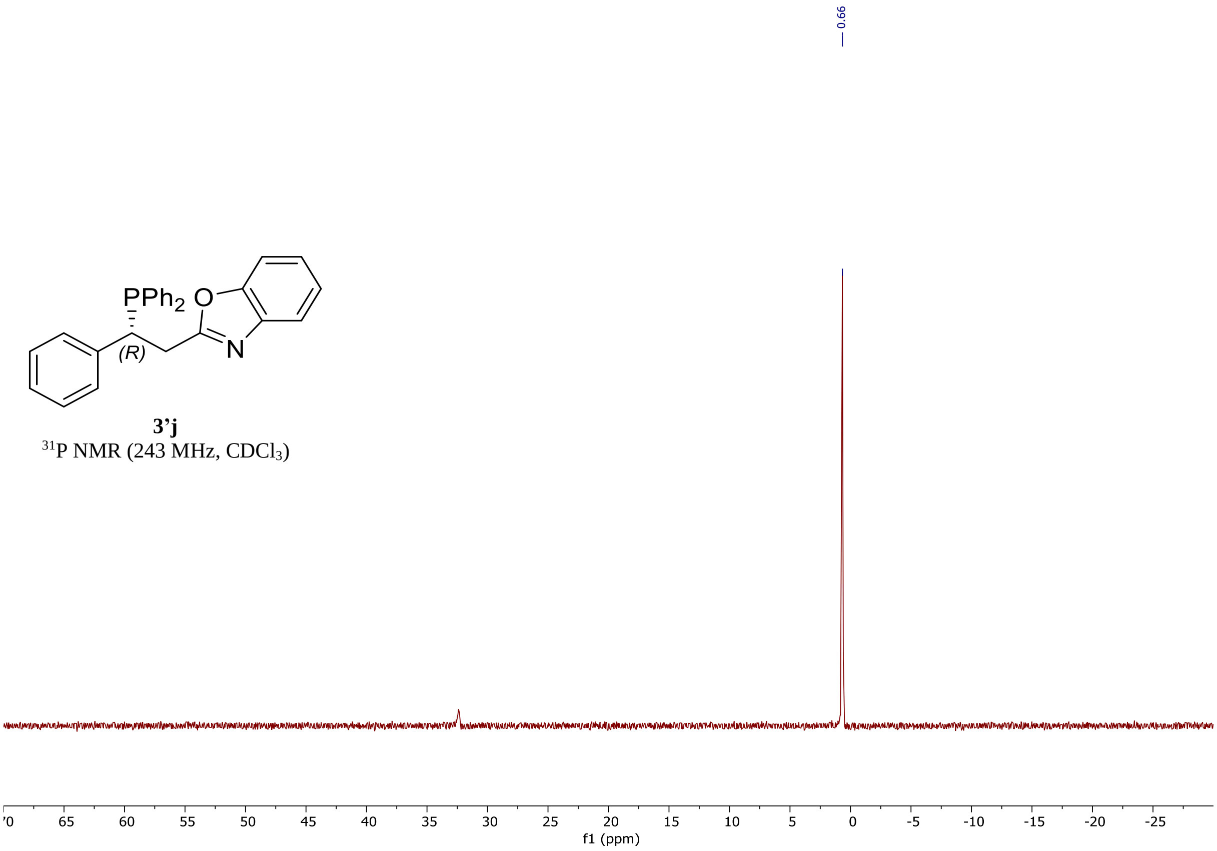

3'j
^{31}P NMR (243 MHz, $CDCl_3$)

11.X-Ray crystallography data

CDDC numbers for the crystallized compounds

Deposition Number 2085289 (**2'e**) contains the supplementary crystallographic data for compound **2'e** in this paper.

Deposition Number 2085288 (**3'b**) contains the supplementary crystallographic data for compound **3'b** in this paper.

A suitable crystal of the oxidized compound **2'e** was mounted on top of a cryoloop and transferred into the cold (100 K) nitrogen stream of a Bruker D8 Venture diffractometer. Data collection and reduction was done using the Bruker software suite APEX3.[31] The final unit cell was obtained from the xyz centroids of 5216 reflections after integration. A multiscan absorption correction was applied, based on the intensities of symmetry-related reflections measured at different angular settings (*SADABS*).[31] The structure was solved by intrinsic phasing methods using *SHELXT*,[32] and refinement of the structure was performed using *SHELXL*.[33] The hydrogen atoms were generated by geometrical considerations, constrained to idealized geometries and allowed to ride on their carrier atoms with an isotropic displacement parameter related to the equivalent displacement parameter of their carrier atoms. Crystal data and details on data collection and refinement are presented in Table S1.

Table S1. Crystallographic data of the oxidized compound **2'e**.

chem formula	$C_{23}H_{22}ClO_3P$
M_r	412.82
cryst syst	orthorhombic
color, habit	colorless, needle
size (mm)	0.305 x 0.076 x 0.061
space group	$P2_12_12_1$
a (Å)	5.6437(2)
b (Å)	17.0149(5)
c (Å)	21.1112(7)
V (Å^3)	2027.24(12)
Z	4
ρ_{calc}, g.cm^{-3}	1.353
Radiation [Å]	Cu K_α 1.54178
μ(Cu K_α), mm^{-1}	2.588
F(000)	864
temp (K)	100(2)
θ range (°)	3.336 – 68.336

data collected (h,k,l)	-6:6; -20:19; -25:25
no. of rflns collected	17304
no. of indpndt reflns	3709
observed reflns $F_o \geq 2.0\ \sigma\ (F_o)$	3436
R(F) (%)	3.15
wR(F^2) (%)	7.64
GooF	1.031
weighting a,b	0.0379, 0.000
params refined	254
min, max resid dens	-0.181, 0.276
Flack x	0.029(10)

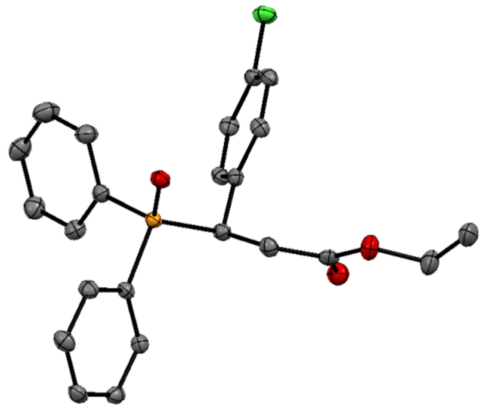

Figure S5. Molecular structure of the oxidized compound **2’e**. Hydrogen atoms are omitted for clarity.

A suitable crystal of the oxidized compound **3’b** was mounted on top of a cryoloop and transferred into the cold (100 K) nitrogen stream of a Bruker D8 Venture diffractometer. Data collection and reduction was done using the Bruker software suite APEX3.[31] The final unit cell was obtained from the xyz centroids of 1386 reflections after integration. A multiscan absorption correction was applied, based on the intensities of symmetry-related reflections measured at different angular settings (*SADABS*).[31] The structures were solved by intrinsic phasing methods using *SHELXT*,[32] and refinement of the structure was performed using *SHELXL*.[33] The hydrogen atoms were generated by geometrical considerations, constrained to idealized geometries and allowed to ride on their carrier atoms with an isotropic displacement parameter related to the equivalent displacement parameter of their carrier atoms. Crystal data and details on data collection and refinement are presented in Table S2.

Table S2. Crystallographic data of the oxidized compound **3’b**.

chem formula	$C_{23}H_{24}NO_2P$
M_r	377.40
cryst syst	orthorhombic
color, habit	colorless, needle
size (mm)	0.289 x 0.165 x 0.082
space group	$P2_12_12_1$
a (Å)	5.7707(5)
b (Å)	16.7521(18)
c (Å)	19.7777(18)
V (Å^3)	1911.9(3)
Z	4
ρ_{calc}, g.cm^{-3}	1.311
Radiation [Å]	Mo K_α 0.71073
μ(Mo K_α), mm^{-1}	0.162
F(000)	800
temp (K)	100(2)
θ range (°)	3.187 – 26.372
data collected (h,k,l)	-6:6; -20:20; -23:23
no. of rflns collected	13847
no. of indpndt reflns	3473
observed reflns $F_o \geq 2.0\ \sigma\ (F_o)$	2918
R(F) (%)	4.12
wR(F^2) (%)	9.10
GooF	1.031
weighting a,b	0.0344, 0.1911
params refined	246
min, max resid dens	-0.253, 0.198
Flack x	-0.03(9)

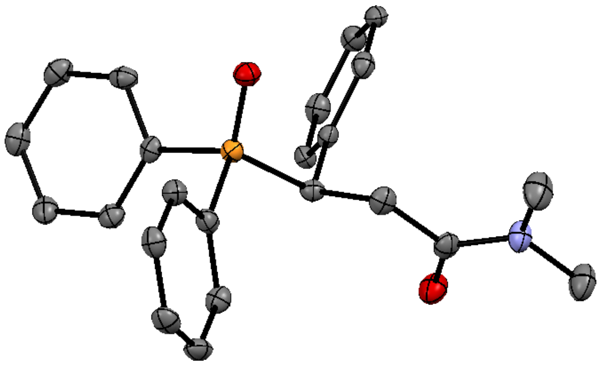

Figure. S6. Molecular structure of the oxidized compound **3'b**. Hydrogen atoms are omitted for clarity.

12.References

[1] M. B. Widegren, G. J. Harkness, A. M. Z. Slawin, D. B. Cordes, M. L. Clarke. A Highly Active Manganese Catalyst for Enantioselective Ketone and Ester Hydrogenation. *Angew. Chem., Int. Ed.* **56**, 5825–5828 (2017).
[2] K. Issleib, K. Jasche [3-Oxo-alkyl]-diorgano-phosphine R"-CO-CH_2-CHR'-PR_2 *Chem. Ber*. **3352**, 3343–3352 (1967).
[3] B. Demerseman, *et al.* Chelating and Hemilabile Properties of β- and γ-Keto Phosphines: (η^6 -Arene)ruthenium(II) Derivatives from γ-Keto Phosphines, Synthesis and Reactivity of Bis(η^2 -keto phosphine-P,O)ruthenium(II) Complexes. *Organometallics* **13**, 2269–2283 (1994).
[4] B. Demerseman, *et al.* Chelating and Hemilabile Properties of β- and γ-Keto Phosphines: (η^6 -Arene)ruthenium(II) Derivatives from γ-Keto Phosphines, Synthesis and Reactivity of Bis(η^2 -keto phosphine-P,O)ruthenium(II) Complexes. *Organometallics* **13**, 2269–2283 (1994).
[5] S. Sabater, J. A. Mata, E. Peris, Synthesis of heterodimetallic iridium-palladium complexes containing two axes of chirality: Study of sequential catalytic properties. *Eur. J. Inorg. Chem.* 4764–4769 (2013).
[6] S. Sabater, J. A. Mata, E. Peris, Synthesis of heterodimetallic iridium-palladium complexes containing two axes of chirality: Study of sequential catalytic properties. *Eur. J. Inorg. Chem.* 4764–4769 (2013).
[7] R. J. Chew, *et al.*, An Approach to the Efficient Syntheses of Chiral Phosphino- Carboxylic Acid Esters. *Adv. Synth. Catal.* **357**, 3297–3302 (2015).
[8] C.-Q. Zhao, Y.-G. Chen, H. Qiu, L. Wei, P. Fang, T.-S. Mei, Water as a Hydrogenating Agent: Stereodivergent Pd-Catalyzed Semihydrogenation of Alkynes. *Org. Lett.* **21**, 1412−1416, (2019).
[9] M. J. Taschner, T. Rosen, C. H. Heathcock, Ethyl isocrotonate. *Org. Synth.* **64**, 108–112 (2003).
[10] P. Franzmann, S. Trosien, M. Schubert, S. R. Waldvogel, Modular Approach to 9-Monosubstituted Fluorene Derivatives Using MoV Reagents. *Org. Lett.* **18**, 1182–1185 (2016).
[11] F. D. Lewis, J. D. Oxman, L. L.Gibson, H. L. Hampsch, S. Quillen, Lewis Acid Catalysis of Photochemical Reactions. 4. Selective Isomerization of Cinnamic Esters. *J. Am. Chem. Soc.* **108**, 3005–3015 (1986).
[12] K. Biswas, S. Woodward, Stereoselective 1,4-addition of Grignard reagents to *α,β*-enamides using a combined chiral auxiliary-catalyst approach. *Tetrahedron Asymmetry* **19**, 1702–1708 (2008).
[13] M. Hirose, M. Okaniwa, Y. Hayashi, T. Takagi, Preparation of heterocyclic compounds as Raf inhibitors for treatment of cancer. PCT Int. Appl.WO 2009028629 (2009).
[14] C. L. Wang, Y. X. Huang, S. R. Sheng, W. Yang, M. Z. Cai, Solid-phase synthesis of (*E*)-3-substituted acrylonitriles from polystyrene-supported α-selenoacetonitrile. *Synth. Commun.* **39**, 1282–1289 (2009).
[15] K. Nishikawa, *et al.*, Substituent effects of cis-cinnamic acid analogues as plant growh inhibitors. *Phytochemistry* (2013).
[16] S. Chandrasekhar, G. Pavan Kumar Reddy, C. Nagesh, C. Raji Reddy, A novel one-pot conversion of amines to homologated esters in poly(ethylene glycol). *Tetrahedron Lett.* **48**, 1269–1271 (2007).
[17] Molloy, J. J.; Schäfer, M.; Wienhold, M.; Morack, T.; Daniliuc, C. G.; Gilmour, R. Boron-Enabled Geometric Isomerization of Alkenes via Selective Energy-Transfer Catalysis. *Science* 369, 302−306 (2020).
[18] C. S. Cho, D. T. Kim, J. Q. Zhang, S.-L. Ho, T.-J. Kim, S. C. Shim, Tin(II) Chloride-Mediated Synthesis of 2-Substituted Benzoxazoles. J. *Heterocycl. Chem*. **39**, 421, (2002).
[19] D. Lanari, M. Alonzi, F. Ferlin, S. Santoro, L. Vaccaro, A Catalytic Peterson-like Synthesis of Alkenyl Nitriles. *Org. Lett.* **18**, 2680–2683 (2016).
[20] K. Zhan, Y. Li, Visible-light photocatalytic E to Z isomerization of activated olefins and its application for the syntheses of coumarins. *Catalysts* **7**, 337-347 (2017).
[21] Y. Chen, L. Huang, M. A. Ranade, X. P. Zhang, Iron(III) and ruthenium(II) porphyrin complex-catalyzed selective olefination of aldehydes with ethyl diazoacetate. *J. Org. Chem.* **68**, 3714–3717 (2003).
[22] D. H. Lee, A.Taher, S. Hossain, M. J. Jin, An efficient and general method for the Heck and Buchwald-Hartwig coupling reactions of aryl chlorides. *Org. Lett.* **13**, 5540–5543 (2011).
[23] J. Wu, X. Cui, L. Chen, G. Jiang, Y. Wu, Palladium-Catalyzed Alkenylation of Quinoline-N-oxides via C-H Activation under External-Oxidant-Free Conditions. *J. Am. Chem. Soc.* **39**, 13888–13889 (2009).
[24] M. Rodríguez-Fernández, X. Yan, J. F. Collados, P. B. White, S. R. Harutyunyan, Lewis Acid Enabled Copper-Catalyzed Asymmetric Synthesis of Chiral β-Substituted Amides. *J. Am. Chem. Soc.* **139**, 14224–14231 (2017).
[25] M. Majewski, G. B. Mpango, M. T. Thomas, A. Wu, V. Snieckus, Metalated Unsaturated Amides. Regio- and Stereoselective γ-Alkylation. *J. Org. Chem.* **46**, 2029–2045 (1981).

[26] Y. Lee *et al.*, (*Z*)-Selective Partial Hydrogenation of Internal Alkynes by Using Palladium Nanoparticles Supported on Nitrogen-Doped Carbon Nanofiber. *ChemCatChem* **4**, 778–781 (2012).
[27] S. Kojima, H. Inai, T. Hidaka, T. Fukuzaki, K. Ohkata, Z-selective synthesis of *α,β*-unsaturated amides with triphenylsilylacetamides. *J. Org. Chem.* **67**, 4093–4099 (2002).
[28] Lai M., Wu Z., Su F., Yu Y., Jing Y., Kong J., Wang Z., Wang S., Zhao M. Synthesis of Cinnamides via Amidation Reaction of Cinnamic Acids with Tetraalkylthiuram Disulfides under Simple Condition, *Eur. J. Org. Chem*. 198–208, (2020).
[29] Duan L., Jiang K., Zhu H., Yin B. $CuCl_2$-Catalyzed Highly Stereoselective and Chemoselective Reduction of Alkynyl Amides into *α,β*-Unsaturated Amides Using Silanes as Hydrogen Donors. *Org. Biomol. Chem*. **2021**, *19*, 365.
[30] R. P. Jumde, F. Lanza, M. J. Veenstra, S. R. Harutyunyan, Catalytic asymmetric addition of Grignard reagents to alkenyl-substituted aromatic N-heterocycles *Science* **352**, 433-437 (2016).
[31] Bruker *APEX3, SAINT and SADABS.* Bruker AXS Inc., Madison, Wisconsin, USA (2016).
[32] Sheldrick, G. M. *Acta Cryst. A*, **71**, 3-8 (2015).
